# Rh-catalyzed decarbonylation of conjugated ynones *via* carbon–alkyne bond activation: reaction scope and mechanistic exploration *via* DFT calculations†
†Electronic supplementary information (ESI) available. See DOI: 10.1039/c5sc00584a



**DOI:** 10.1039/c5sc00584a

**Published:** 2015-03-31

**Authors:** Alpay Dermenci, Rachel E. Whittaker, Yang Gao, Faben A. Cruz, Zhi-Xiang Yu, Guangbin Dong

**Affiliations:** a The University of Texas at Austin , Department of Chemistry , Austin , TX 78712 , USA; b Beijing National Laboratory of Molecular Sciences (BNLMS) , Key Laboratory of Bioorganic Chemistry and Molecular Engineering , College of Chemistry , Peking University , Beijing , 100871 , China; c Key Laboratory of Pesticide & Chemical Biology , Ministry of Education , College of Chemistry , Central China Normal University , Hubei , Wuhan 430079 , China

## Abstract

In this full article, detailed development of a catalytic decarbonylation of conjugated monoynones to synthesize disubstituted alkynes is described. The reaction scope and limitation has been thoroughly investigated, and a broad range of functional groups including heterocycles were compatible under the catalytic conditions. Mechanistic exploration *via* DFT calculations has also been executed. Through the computational study, a proposed catalytic mechanism has been carefully evaluated. These efforts are expected to serve as an important exploratory study for developing catalytic alkyne-transfer reactions *via* carbon–alkyne bond activation.

## Abstract 2

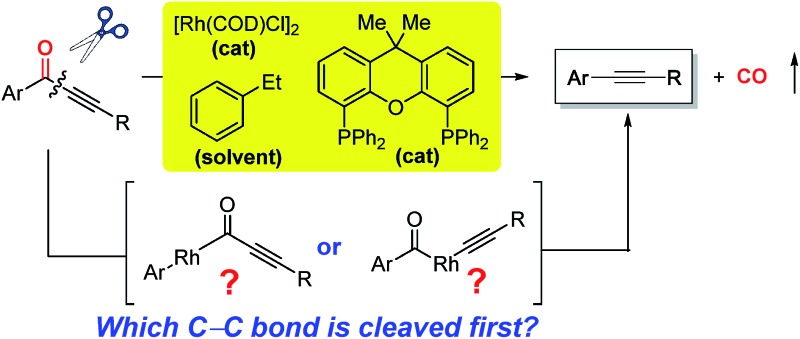
We report a catalytic C–C bond activation of unstrained conjugated monoynones *via* decarbonylation to synthesize disubstituted alkynes.

## Introduction

**Scheme 1:**
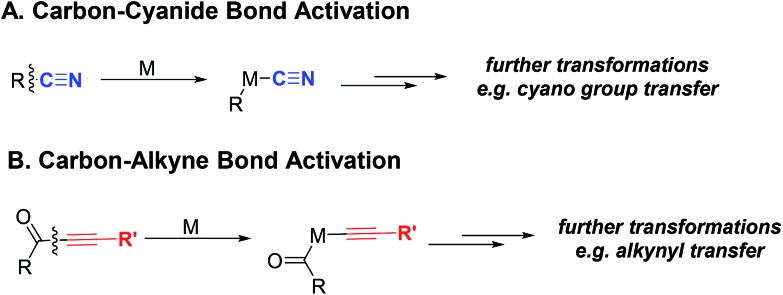
C–C activation of nitriles and ynones.

Transition metal-mediated carbon–carbon σ bond (C–C) activation offers a distinct strategy to construct or assemble organic molecules from unexpected, yet readily available starting materials.^1,2^ Despite a number of C–C activation modes reported to date, limited catalytic approaches are available without relying on release of ring strain or use of an auxiliary directing group.^3^ One important example that avoids these requirements is the catalytic activation of C–CN bonds, which has found broad usage in organic synthesis enabling “CN transfer” transformations (Scheme 1A).^4^ Given that the cyano group can be readily converted to other functional groups, such as amides or amines, the C–CN activation approach can potentially be employed to streamline synthesis of nitrogen-containing molecules.^5^

**Scheme 2:**
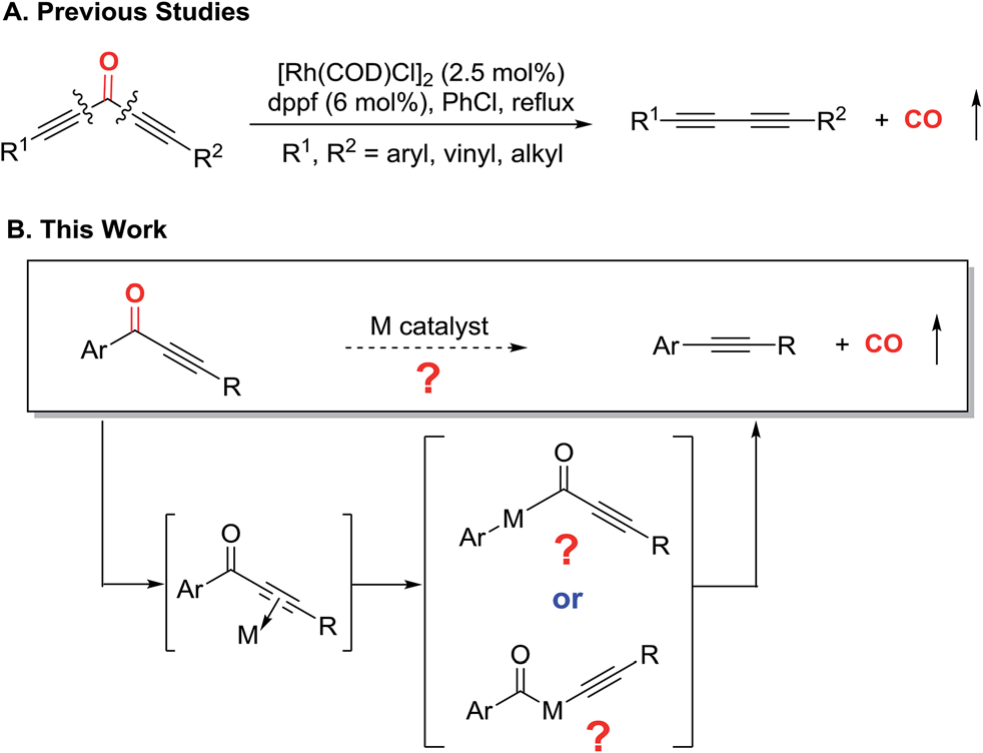
Catalytic decarbonylation of conjugated diynones and monoynones *via* C–C activation.

Considering that alkynes also have sp hybridized carbons like the cyano group, it would be impactful if the analogous activation of the carbon–alkyne bond could be realized (Scheme 1B). Alkynes have rich chemical reactivity and can serve as a latent functional group for alkenes, alkanes, ketones, diones, vicinal carbenes, *etc.*^6^ Thus, transformations coupled with carbon–alkyne bond activation should be synthetically useful. However, in contrast to the C–CN bond, the carbon–alkyne bond is much less polarized. Consequently, only a few isolated cases on carbon–alkyne bond activation, *i.e.* oxidative addition of a transition metal into a carbon–alkyne bond, have been reported. One seminal example is C–C cleavage followed by decarbonylation of conjugated diynones with stoichiometric Wilkinson's complex by Müller in 1969;^7^ later, oxidative addition of rhodium(i) into a quinoline-derived acyl–alkyne bond was disclosed by Suggs in 1981.^8^ Another example is photochemical cleavage of the aryl–alkyl bond in diarylalkynes with platinum(0) complexes.^9^ To the best of our knowledge, it was not until our recent report that the catalytic transformation involving carbon–alkyne bond activation was realized.^10^ Our laboratory has been particularly interested in developing catalytic transformations involving C–C activation of ketone compounds.^11^ In the previous communication, we described an initial effort on catalytic decarbonylation of diynones to synthesize various 1,3-diynes (Scheme 2A).^10^ Under the optimized conditions, both symmetrical and unsymmetrical diynones are suitable substrates, and a number of functional groups are tolerated. This C–C activation approach is complementary to transition metal-catalyzed cross couplings (*e.g.* compatibility with aryl bromides and iodides), and has been further applied to natural product derivatization.

With these preliminary results in hand, two key questions remained to be addressed: (1) are both alkyne moieties required to maintain the catalytic activity for cleaving the carbon–alkyne bond; (2) if not (i.e. if only one alkynyl group is sufficient), in the absence of any auxiliary directing group, which C–C bond gets cleaved first for monoynones (Scheme 2B)? Stimulated by these questions, we first describe a detailed development of a catalytic system that is effective for decarbonylation of conjugated monoynones, then disclose the reaction scope and limitation, and finally report our mechanistic exploration *via* DFT calculations. Through the computational efforts, we obtained a better understanding about the reaction mechanism, particularly about the rate-limiting step and which C–C bond is first activated. These efforts are expected to serve as an important exploratory study for developing catalytic alkyne-transfer reactions *via* carbon–alkyne bond activation.

## Results and discussion

### 1. Reaction optimization

In 1969, Müller reported a single example that bisphenylynone **1a** reacted with one equivalent of Wilkinson's complex [RhCl(PPh_3_)_3_] in refluxing xylenes giving 8% yield of diphenylacetylene **2a**.^7^ Although occurring with low efficiency, this seminal observation offered an opportunity to apply our knowledge of diynone activation into developing a catalytic decarbonylation of monoynones. However, under our previously optimized conditions (*vide supra*, Scheme 2A), 2.5 mol% [Rh(COD)Cl]_2_ and 6 mol% dppf in refluxing chlorobenzene did not provide any decarbonylation product **2a**. This initial result was a clear indication of the difference in reactivity between diynones and monoynones for decarbonylation. The significantly reduced reactivity of monoynones, compared to diynones, can be possibly explained by the following: (1) the C–C bonds α to the carbonyl of monoynones are more sterically demanding; and (2) the carbonyl group is also less electrophilic (reduced LUMO coefficient) than the one of diynones (both factors would hinder oxidative addition). Clearly, to develop a catalytic decarbonylation of monoynones, a more active catalyst system needed to be discovered.

The optimization studies began with ynone **1a** as the model substrate (Table 1). Solvents with higher boiling points than chlorobenzene were examined first. When the reaction was run with 5 mol% [Rh(COD)Cl]_2_ and 12 mol% dppf in refluxing xylenes (150–157 °C), we were pleased to find that the desired decarbonylation product **2a** was obtained in 24% yield (34% conversion of starting material, entry 1). With all other variables held constant, we surveyed a number of bidentate ligands with various bite angles, which were previously found to be important for decarbonylating diynones.^10,13^ Ligands, such as dppm, dppe, and dppp, with bite angles less than dppf (96°) showed trace or decreased yields (entries 2–4). On the other hand, bidentate ligands with larger bite angles or bulky monodentate ligands provided increased yields: while dppb slightly improved the yield (29%, entry 5), *t*-BuXphos and Xantphos^13*b*^ gave improved yields (43% and 85%, entries 7 and 8, respectively). Unexpectedly, DPEphos gave a lower yield (11%, entry 6). Satisfied with Xantphos as the ligand, other reaction parameters were then explored. The commercially available xylenes contain a mixture of *m*-, *o*-, and *p*-isomers, as well as a small amount of ethylbenzene. Surprisingly, all *m*-, *o*-, and *p*-xylenes showed lower yields (29–63%, entries 9–11) than mixed xylenes; in contrast, ethylbenzene gave the highest yield (**91%**, entry 12). In addition, a series of Lewis acids, ruthenium co-catalysts and rhodium precatalysts were also examined, albeit with no improvement observed (for details, see ESI, Table S1†).

**Table 1:** Selected optimization studies^*a*^

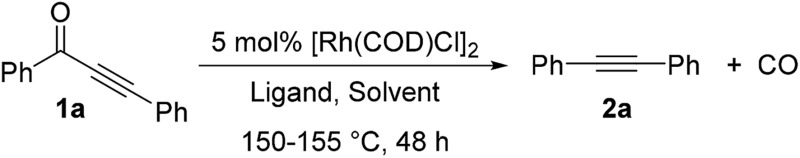				
Entry	Ligand (12 mol%)	Solvent	Bite angle^*b*^ (°)	Yield^*c*^
1	dppf	Xylenes	96	24% (34%)
2	dppm	Xylenes	72	<5%
3	dppe	Xylenes	85	<5%
4	dppp	Xylenes	91	13%
5	dppb	Xylenes	98	29%
6	DPEphos	Xylenes	104	11%
7	*t*-BuXphos	Xylenes	—	43%
8	Xantphos	Xylenes	111	85%
9	Xantphos	*m*-Xylene	111	63% (71%)
10	Xantphos	*o*-Xylene	111	29% (35%)
11	Xantphos	*p*-Xylene	111	62%
**12**	**Xantphos**	Ethylbenzene	**111**	**91%**

^*a*^Conditions: ynone **1a** (0.20 mmol), [Rh] : ligand = 1 : 1.2, solvent (0.1 M).

^*b*^See ref. 12 for bite-angle values.

^*c*^Isolated yields; number in parenthesis is percent conversion of starting material.

### 2. Substrate scope and limitation

With a standard set of conditions in hand, the substrate scope of the reaction was explored. Keeping the alkyne moiety of the substrate fixed, a range of aryl substituted ynones were investigated under the decarbonylation conditions (Table 2). In general, good to high yields can be afforded with substrates (**2a–2k**) containing either electron-donating or withdrawing aryl groups, showing no obvious electronic bias. Interestingly, the 4-nitrophenyl substrate (**1h**) gave a higher yield (77%, **2h**) when using dppf as the ligand and xylenes as the solvent, compared to 40% yield (85% conversion of starting material) under the standard conditions. Functional groups, such as –F, –CN, –Cl, and –CO_2_Me, were also found compatible. Substrates containing heterocyclic groups, such as furans (**1l**) and pyridines (**1m** and **1n**), also underwent decarbonylation smoothly, particularly the 3-pyridyl group which showed superior reactivity (**1n**).

**Table 2:** Substrate scope based on ketone substitution^*a*^

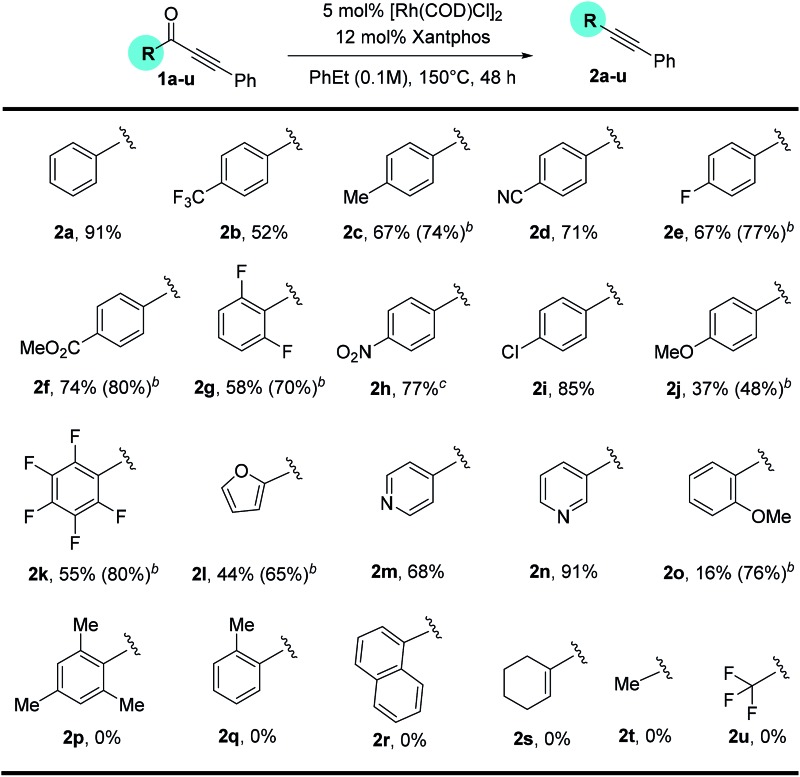

^*a*^Reactions were run on a 0.20 mmol scale; all yields are isolated yields.

^*b*^Number in parenthesis is percent conversion of starting material.

^*c*^dppf and xylenes were used.

An important observation is that this reaction is highly sensitive to the sterics around the carbonyl group. Substrates having substituents at the *ortho*-position (**2o–2r**) showed a dramatic decrease in yield, potentially hindering the substrate binding to the metal center. In addition, replacement of the aryl group with an alkenyl or alkyl substituent (**1s–1u**) resulted in no conversion to products (recovery of most of the starting materials).

**Scheme 3:**
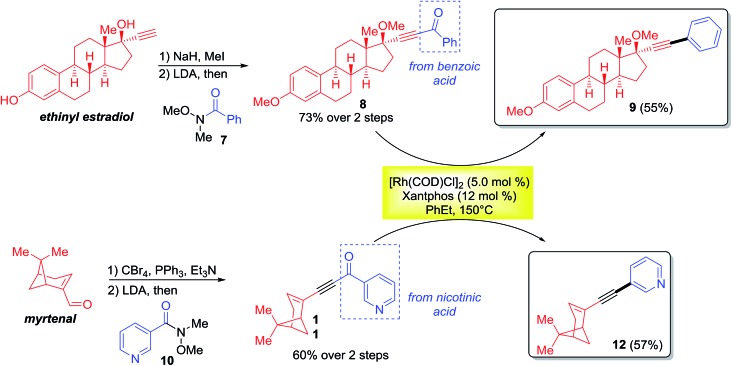
Applications in natural-product derivatization.

Substitution on the alkyne end of the substrates was also explored with the ketone end held constant as a phenyl group (Table 3). In general, both electron-donating and withdrawing aryl substituents were tolerated, giving synthetically useful yields (**4a–4d**, 60–73%). However, substrates containing a *para*-halogen substituent provided much lower yields (**4e–4g**), though the exact reason is unclear (*vide infra*, enhanced yields in Table 4). Furan (**3h**) and thiophene (**3i**) substrates also furnished the desired products, albeit in low yields. Certain alkyl substituents at the alkyne end were also tolerated (**3j**) and *vide infra*, ethynyl estradiol-derived ynone (Scheme 3). However, *t*-Bu, trifluoromethyl, trimethylsilyl or linear alkyl substituents proved unreactive under the standard conditions. Under forcing conditions, *i.e.* in refluxing mesitylene (168–170 °C with all other parameters remaining the same), linear alkyl substrates (**3l** and **3o**) gave exclusive formation of the cycloisomerized furan products, which is likely though an alkyne–allene isomerization pathway (for details, see ESI, Scheme S1†). Moreover, while cyclohexenyl ynone **3n** showed no reaction under the standard conditions, in refluxing mesitylene the decarbonylation product **4n** was able to form in 14% yield.^14^

**Table 3:** Substrate scope based on phenyl ynones^*a*^

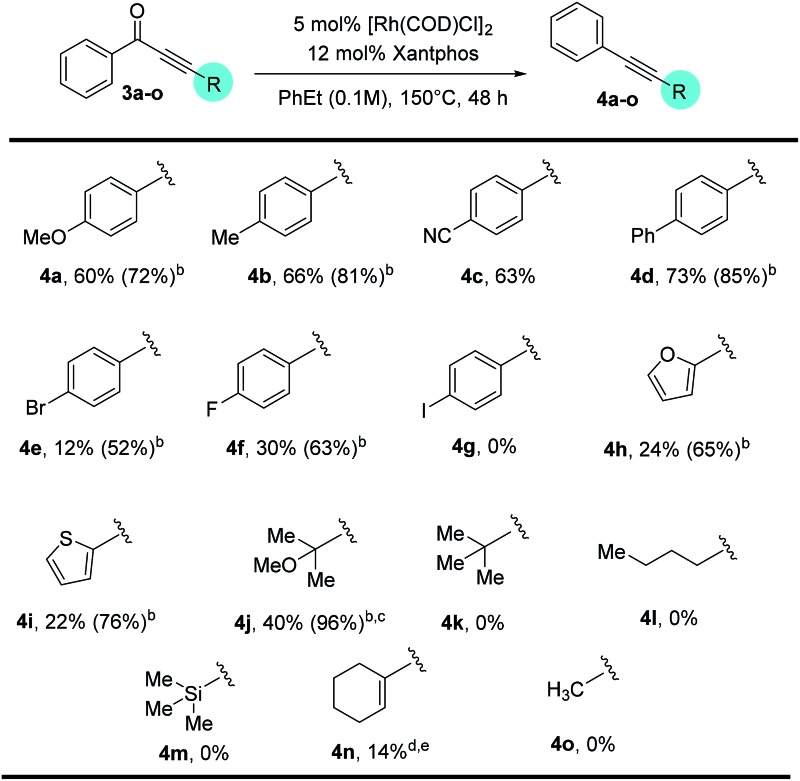

^*a*^Reactions were run on a 0.2 mmol scale; unless otherwise mentioned, all yields are isolated yields.

^*b*^Number in parenthesis is percent conversion of starting material.

^*c*^Product **4j** is slightly volatile.

^*d*^The reaction was run in mesitylene at 170 °C.

^*e*^The yield is based on ^1^H NMR using C_2_H_2_Cl_4_ as the internal standard.

**Table 4:** Substrate scope based on 3-pyridyl ynones^*a*^

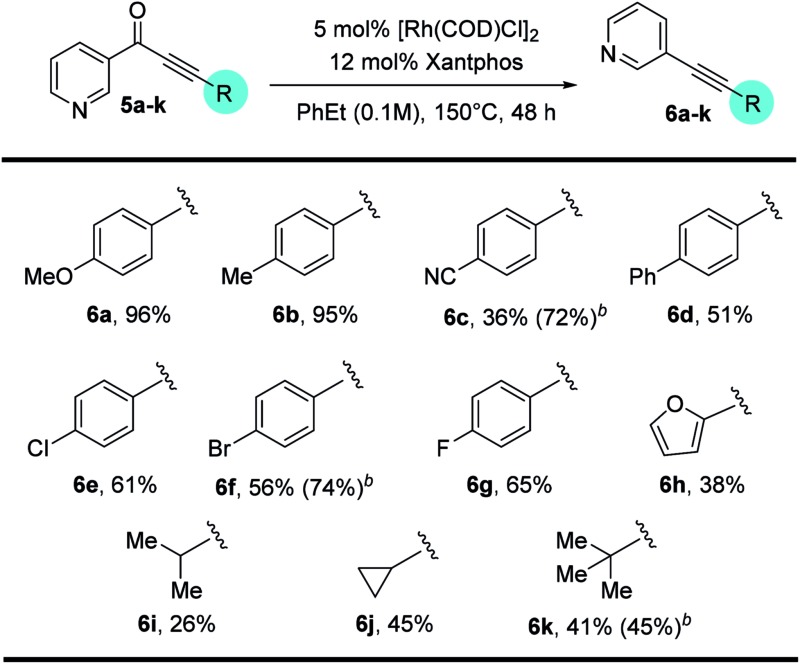

^*a*^Reactions were run on a 0.2 mmol scale; all yields are isolated yields.

^*b*^Number in parenthesis is percent conversion of starting material.

In contrast, when 3-pyridyl was used as the acyl substituent, the reactivity of the ynone substrates was greatly increased (Table 4). We were pleased to observe that a range of pyridine-containing disubstituted alkynes were isolated with enhanced conversions, and many functional groups were tolerated. Notably, the yields for the substrates containing halogen and heterocycles were significantly improved (12% for **4e***vs.* 56% for **6f**, and 24% for **4h***vs.* 38% for **6h**). In addition, the scope for the alkenyl and alkyl substituted substrates were expanded. Though straight alkyl ynones remain problematic giving allene isomerization, to our delight, branched alkyl substrates (**5i–k**) were found to be reactive and afforded products (**6i–k**) in modest to good yield (26–45%).

The monoynone decarbonylation reaction has been further investigated in the derivatization of natural products (Scheme 3). For example, the ethynyl estradiol and myrtenal derived monoynones (**8** and **11**) smoothly gave the corresponding decarbonylated products **9** and **12** in 55% and 57% yields, respectively. Note that the aryl groups coupled with the natural products ultimately come from the corresponding carboxylic acids.

**Scheme 4:**
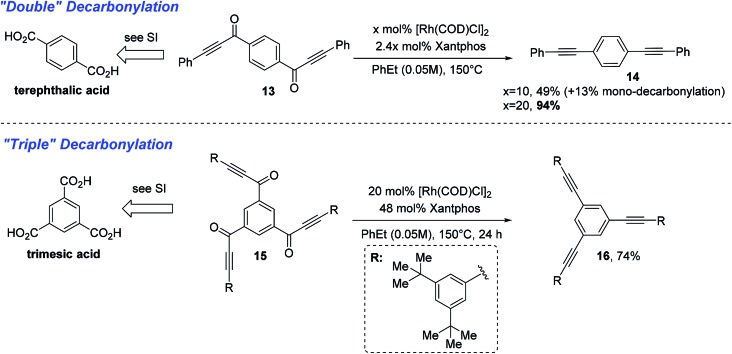
Multiple decarbonylations.

With a thorough exploration of the reaction scope and a better understanding of substrate reactivity, we finally examined substrates that can undergo multiple decarbonylations. When terephthalic acid-derived di-ynone **13** was subjected to the standard conditions, the doubly decarbonylated product (**14**) was obtained albeit in low yield along with severe decomposition to unidentified oligomers (Scheme 4). After further examining the reaction conditions, we found that use of lower concentrations can dramatically minimize the product decomposition to unidentified oligomers. Finally, with an increase of the catalyst loading at 0.05 M, the double decarbonylation product can be obtained in 94% yield.^15^ Additionally, when trimesic acid-derived tri-ynone **15** was subjected to the above-optimized conditions, the tri-yne product **16** was isolated in 74% yield.

### 3. Mechanistic studies *via* DFT calculation

**Fig. 1:**
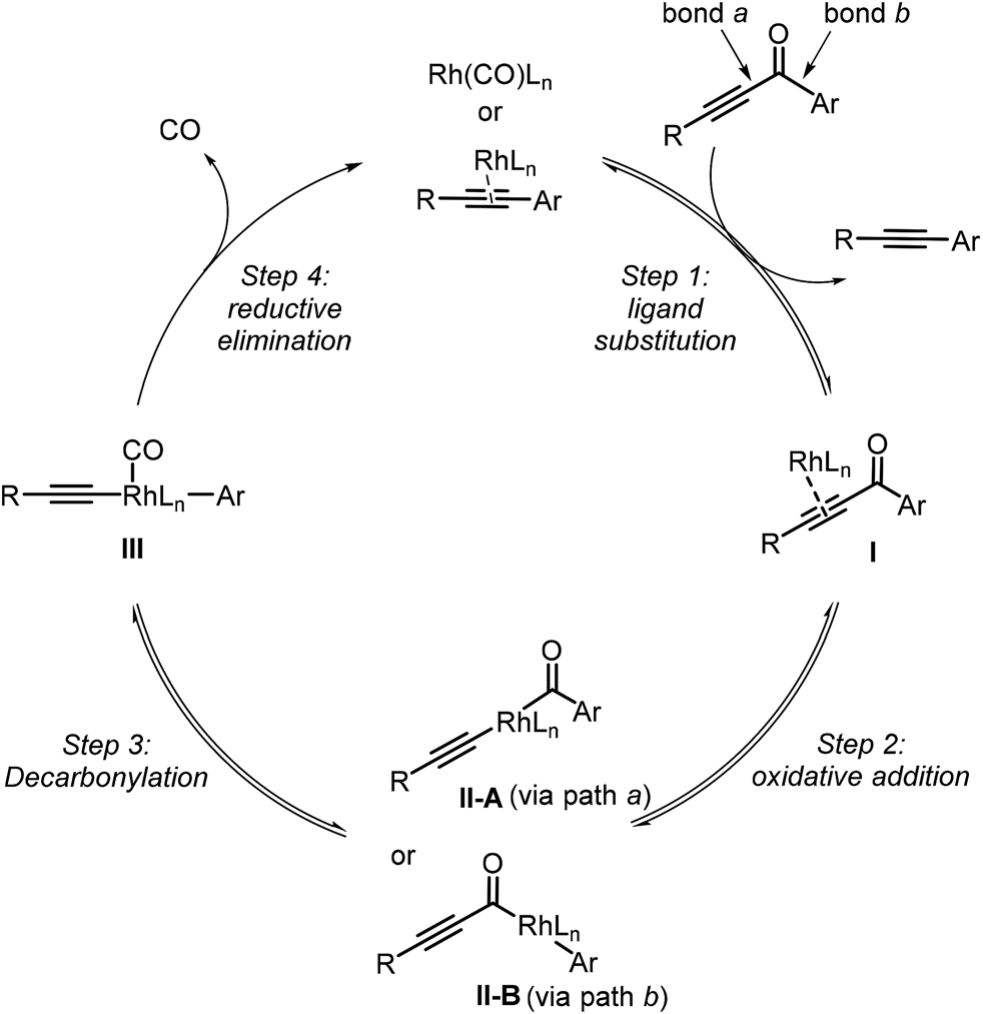
The proposed mechanism of the Rh-catalyzed decarbonylation of monoyones.

Our proposed mechanism of the Rh-catalyzed decarbonylation involves four steps: ligand substitution, oxidative addition, decarbonylation, and reductive elimination (Fig. 1). The initial step involves substrate coordination to the Rh(i) through the alkynyl group, giving complex **I** (step 1). The second step is oxidative addition, leading to Rh(iii) complex **IIA** (rhodium is inserted into *bond a* between the alkynyl and carbonyl groups of the substrate, pathway a) or **IIB** (rhodium is inserted into *bond b* between the aryl and carbonyl groups of the substrate, pathway b). Decarbonylation transforms **IIA** or **IIB** into intermediate **III**, which then undergoes reductive elimination to give the final product (step 4). Herein, we report density functional theory (DFT) calculations, to support this proposal and gain a better understanding of the mechanistic details.

**Fig. 2:**
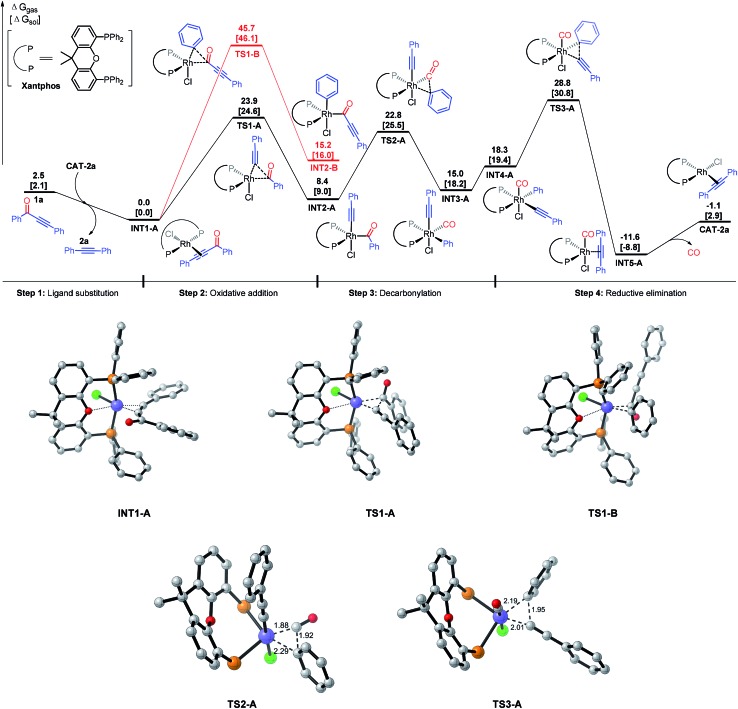
Comparison of the energy profiles (Δ*G* in kcal mol^–1^) of path a (black) and path b (red) for decarbonylation of ynone **1a**, and DFT optimized structures of transition states and key intermediates (distances in Å, hydrogen atoms and phenyl groups of Xantphos were omitted for clarity).

DFT calculations were based on the model reaction of ynone **1a** to alkyne **2a**. The full model of the best ligand, Xantphos, was used for the DFT studies. The energy profiles of paths a and b were shown in Fig. 2. The discussed energies here are the relative free energies in the gas phase, considering that the conclusions extracted from the gas phase and solvent are the same (see the DFT computed values in the parentheses in Fig. 2 for the relative free energies of the reaction in ethylbenzene solvent).

First, we discuss the energy surface of pathway a (Fig. 2). The catalytic cycle starts from ligand exchange reaction between **CAT-P** and substrate **1a**, giving catalyst–substrate complex **INT1** and releasing the decarbonylation product **2a**. Substrate **1a** could coordinate to the Rh center through either the alkyne group or the carbonyl group. DFT calculations indicate that the alkyne-coordinated complex is more stable than the carbonyl-coordinated complex by 7.1 kcal mol^–1^ and therefore formation of **INT1** is preferred. **INT1** then undergoes oxidative addition into *bond a* (pathway a) *via***TS1-A**, requiring an activation free energy of 23.9 kcal mol^–1^. This step is endergonic by 8.4 kcal mol^–1^ and generates **INT2-A**. A reversible decarbonylation *via***TS2-A** subsequently transforms **INT2-A** to **INT3-A**, requiring an activation free energy of 14.4 kcal mol^–1^. The decarbonylation step is endergonic by 6.6 kcal mol^–1^. Subsequently, ligand reorganization converts **INT3-A** to **INT4-A**, which undergoes reductive elimination to give to **INT5** (*via***TS3-A**).^16^ The final reductive elimination step has an activation free energy of 10.5 kcal mol^–1^ and is irreversible (it is exergonic by 29.9 kcal mol^–1^). Our calculations indicated that in pathway a, the rate-determining step of the catalytic cycle is the reductive elimination step and the overall activation free energy of the catalytic cycle is 28.8 kcal mol^–1^ in gas phase. Using ethylbenzene as the solvent, the computed overall activation free energy is 30.8 kcal mol^–1^.^17^ The calculation results here reasonably explain why experimentally the decarbonylation reaction had to be carried out at 150 °C.

An alternative pathway is rhodium insertion (from **INT1**) into *bond b* (**INT2-B**, between the carbonyl and aryl groups (pathway b)), which is disfavored by more than 20 kcal mol^–1^ compared to the insertion into *bond a* in pathway a. The computed activation energy barrier for this step is 45.7 kcal mol^–1^, which is much higher than the total activation energy in pathway a. Consequently, pathway b can be excluded from further consideration. To rationalize the above observation, we propose that the regioselectivity of the C–C bond cleavage can be controlled by a *trans* effect (TE), also known as *trans* influence when considering the ground state of the complex.^18^ The intermediate (**INT-2A** or **INT-2B**) after the oxidative addition step should contain three X-ligands: the acyl, phenyl, and acetylide. Acyl and phenyl are very strong TE σ-donor ligands, while acetylide ligand is a weak TE ligand (weaker than phosphine). Cleavage *b* bond will generate two strong TE ligands: the acyl and phenyl ligands. In this case, the chloride ligand (a moderately strong TE ligand) has to be in a *trans* position to either the acyl ligand or phenyl ligand, which is not favored based on the TE.^18^ In contrast, cleavage of the *a* bond will generate one strong TE ligand, the acyl ligand, and one weak TE ligand, the acetylide ligand. In this case, the strong TE ligand (*i.e.* the acyl group) can be arranged to a position that is *trans* to the oxygen of the Xantphos ligand to reduce the TE, while the weak TE ligand (*i.e.* the acetylide group) can be *trans* to the chloride (the geometry rearrangement is illustrated in **TS1-A**).

**Fig. 3:**
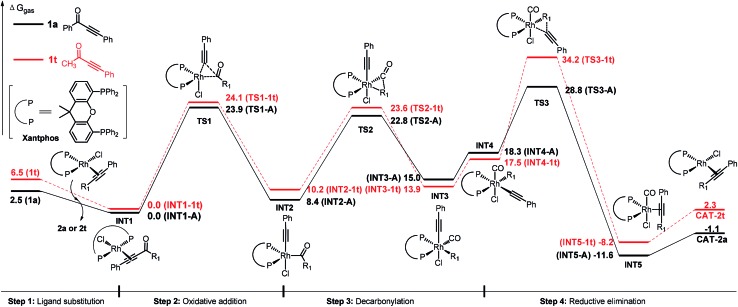
Comparison of the energy profiles (Δ*G* of gas phase in kcal mol^–1^) of **1a** (black) and **1t** (red).

Experimentally, we found that replacement of the aryl group with an alkyl substituent (such as methyl group, **2t**) resulted in no conversion to product (Table 2). DFT studies on the substituent effect between phenyl substrate **1a** and methyl substrate **1t** have been performed (Fig. 3). The rate-determining step of **1t** is also the reductive elimination step, but the overall activation free energy for the decarbonylation is 34.2 kcal mol^–1^, which is 5.4 kcal mol^–1^ higher than that of **1a**. Due to this reason, the reaction of **1t** did not occur under the experimental conditions that are suitable for **1a**.

**Fig. 4:**
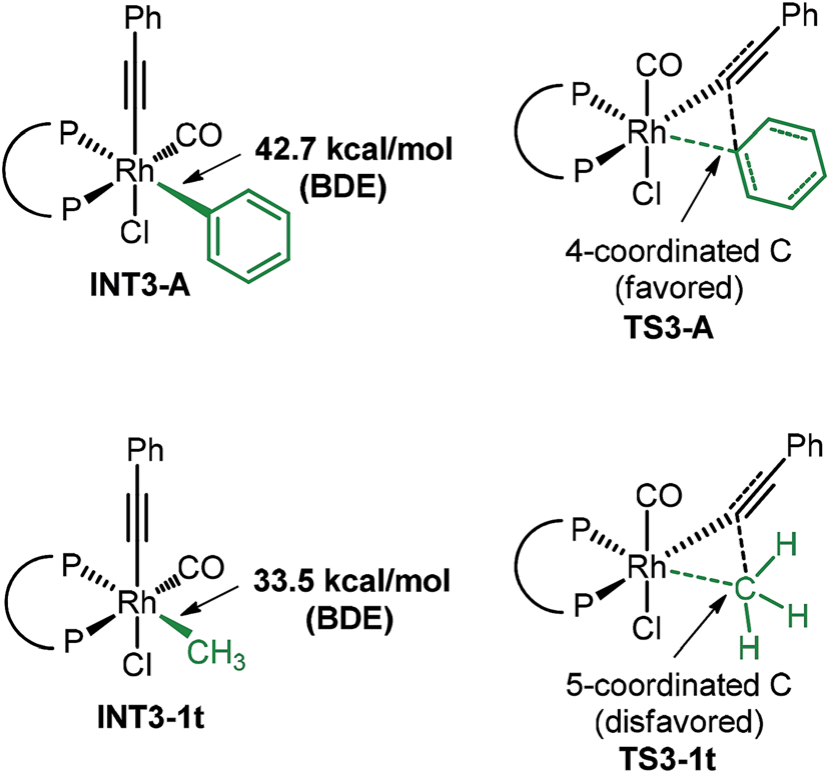
The computed BDEs of **INT3-A** and **INT3-1t** and the comparison of two reductive elimination transition states. The ligand here is Xantphos.

The higher activation free energy of **1t** compared to that of **1a** is mainly caused by the more difficult reductive elimination step in the former case. In **1t**, the reductive elimination has an energy barrier of 16.7 kcal mol^–1^, which is 6.2 kcal mol^–1^ higher than that of **1a** (10.5 kcal mol^–1^). This result is consistent with our previously observed faster reductive elimination with a C(sp^2^) group than a C(sp^3^) group through DFT calculations.^19^ What is the intrinsic reason for this difference? Here is our proposed explanation. Although the Rh–phenyl bond in **INT3-A** has a higher energy than the Rh–methyl bond in **INT3-1t** (our calculated results, Fig. 4), in the transition state of the reductive elimination step the migrating carbon in the phenyl group is four-coordinated and the charge in this phenyl group can be well distributed into the aromatic ring (**TS3-A**). In contrast, the migrating carbon in the methyl group (**TS3-1t**) is energetically disfavored (five-coordinated), and this requires additional energy compared to the four-coordinated phenyl group in **TS3-A**.

Our DFT calculations also found that the dppp ligand is not effective for the present decarbonylation reaction. This is mainly due to a disfavored reductive elimination step (even though the C–C cleavage step is not difficult). For the computed energy surface, see the ESI.†

## Conclusion

In conclusion, we have reported a detailed experimental and computational study of the Rh-catalyzed decarbonylation of conjugated monoynones, which should serve as an initiative towards developing catalytic alkyne transfer reactions *via* carbon–alkyne bond activation (a long-term goal). We have discovered an active catalytic system that is suitable for a range of substrates and functional groups stemming from readily available carboxylic acids. From the experimental study, the scope and limitation of this transformation have been thoroughly explored. From the computational study, a proposed catalytic mechanism has been carefully evaluated. Employing the theoretic models, we now have a better understanding about how the C–C bond in monoynones is activated, ruling out the pathway involving initial cleavage of the aryl–carbonyl bond and favoring cleavage of the alkynyl–carbonyl bond. In addition, the calculation results support that reductive elimination is the rate-determining step for the catalytic cycle. Furthermore, we obtained key information about why the aryl ketones are more reactive than the corresponding alkyl ketones.

With all the mechanistic information of the ynone decarbonylation in hand, further investigations to discover the alkyne-transfer transformations (analogous to the CN transfer reactions^6^) are currently underway in our laboratories.

## Computational details

All calculations were performed with the Gaussian 09 program.^20^ Density functional theory calculations using the B3LYP method were used to locate all the minima and transition points involved.^21^ The 6-31G(d) basis set^22^ was applied for all elements except for Rh, for which the LANL2DZ basis set and pseudopotential^23^ were used. The key word “5D” in Gaussian 09 program was used. Frequency calculations at the same level had been performed to confirm each stationary point to be either a minimum or a transition structure and to evaluate its zero-point energy and the thermal corrections at 298 K. Both single-point energies and solvation energies based on the geometry structures obtained at the B3LYP level were obtained by M06L method^24^ using a higher level basis set, LANL2TZ(f) basis set and pseudopotential^25^ for Rh and 6-311+G(d,p) basis set for all the other atoms in order to take the dispersion energies into consideration. Solvation energies in ethylbenzene were evaluated by a self-consistent reaction field (SCRF) using the SMD model with radii and non-electrostatic terms. Bond dissociation energies (BDE) are discussed as bond-homolysis into two radicals in B3LYP/6-31G(d) level in gas phase. In the paper and the ESI,† all discussed energies are Gibbs free energies in gas phase (Δ*G*_gas_) at 298 K unless specified. We found that the conclusions in both the gas phase and ethylbenzene are the same. Computed structures are illustrated using CYLVIEW drawings.^26^

## Abbreviations

COD: Cyclooctadiene
COE: Cyclooctene
dppf: 1,1′-Bis(diphenylphosphino)ferrocene
dppp: 1,3-Bis(diphenylphosphino)propane
dppe: 1,2-Bis(diphenylphosphino)ethane
dppb: 1,3-Bis(diphenylphosphino)butane
Xantphos: 4,5-Bis(diphenylphosphino)-9,9-dimethylxanthene
DPEphos: (Oxydi-2,1-phenylene)bis(diphenylphosphine)
dppm: 1,1-Bis(diphenylphosphino)methane
*t*-BuXphos: 2-Di-*tert*-butylphosphino-2′,4′,6′-triisopropylbiphenyl

## Supplementary Material

Supplementary information

Supplementary information
