# Supplementary figures and images for: Rh-catalyzed decarbonylation of conjugated ynones via carbon–alkyne bond activation: reaction scope and mechanistic exploration via DFT calculations
Source: Chem Sci. 2015 Mar 31;6(5):3201–10. doi: 10.1039/c5sc00584a (PMC4517480; doi:10.1039/c5sc00584a)

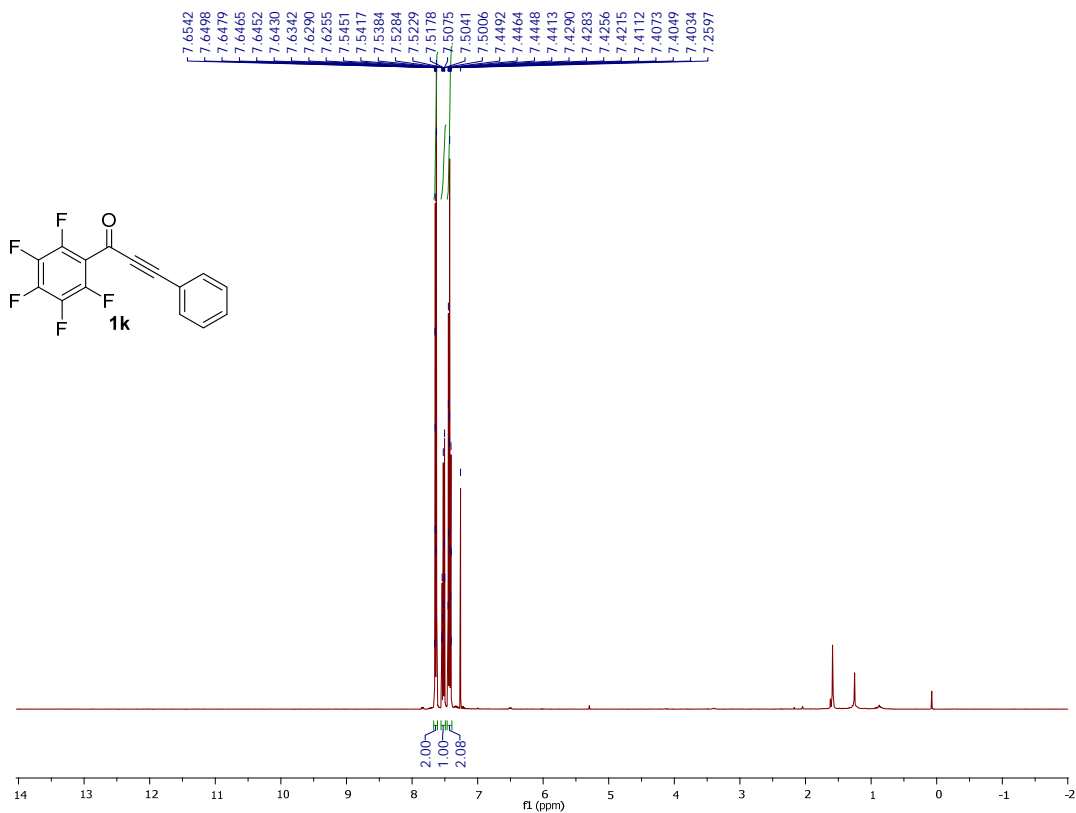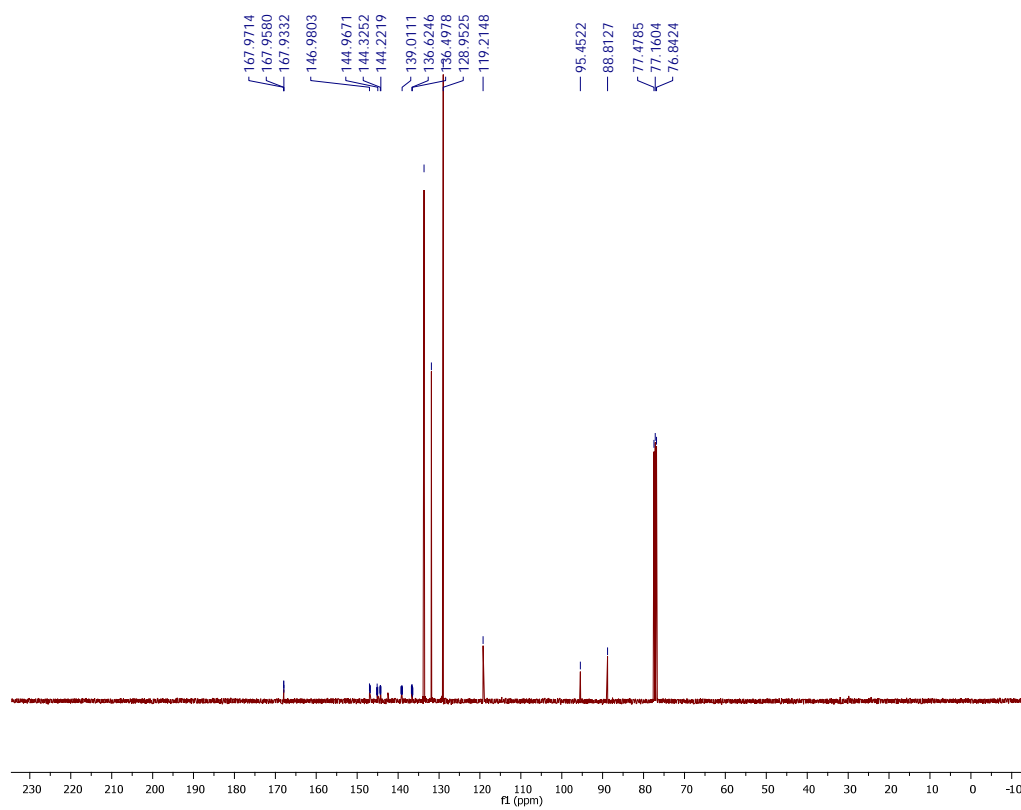

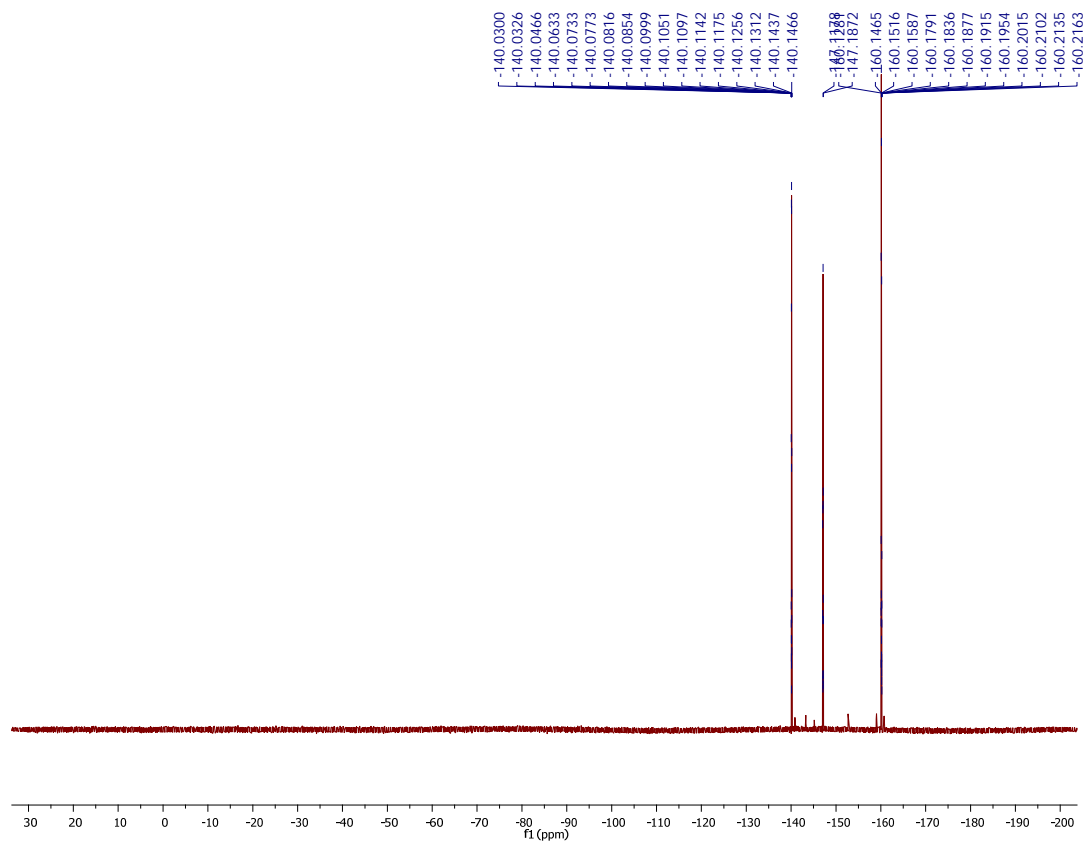

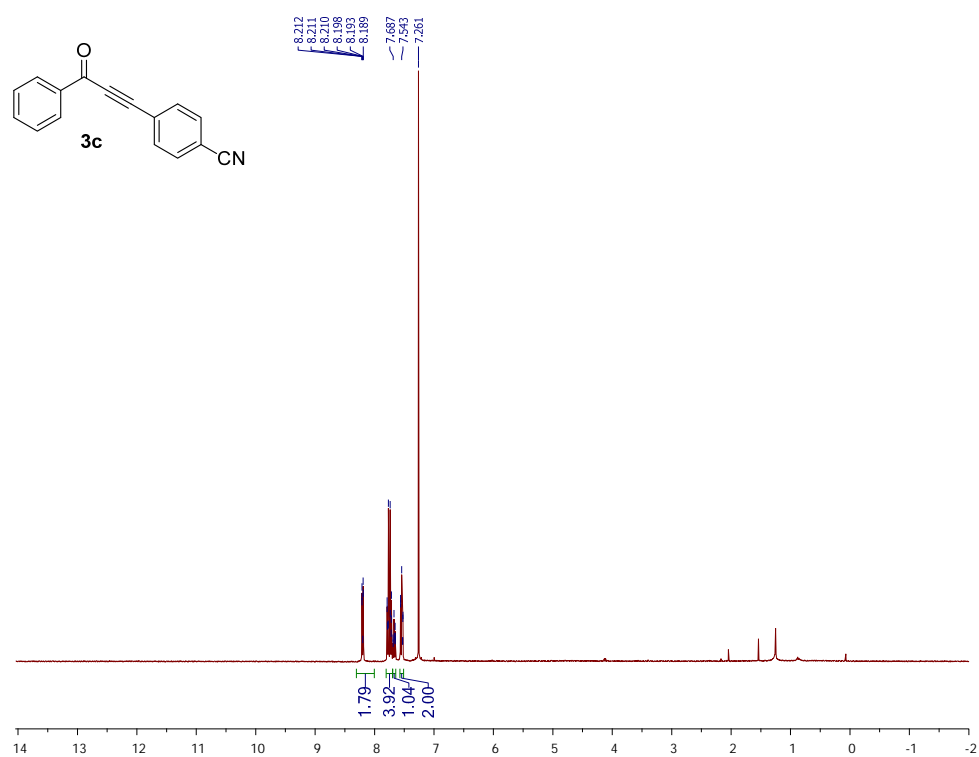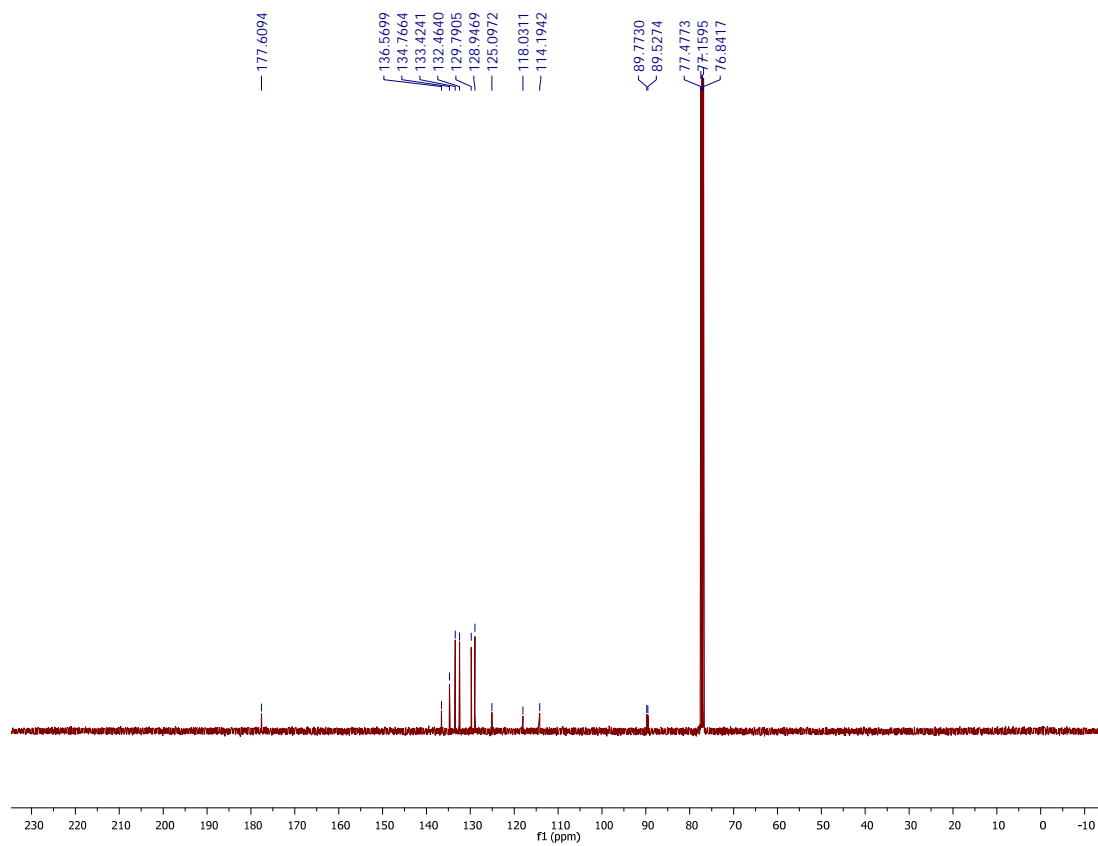

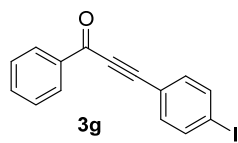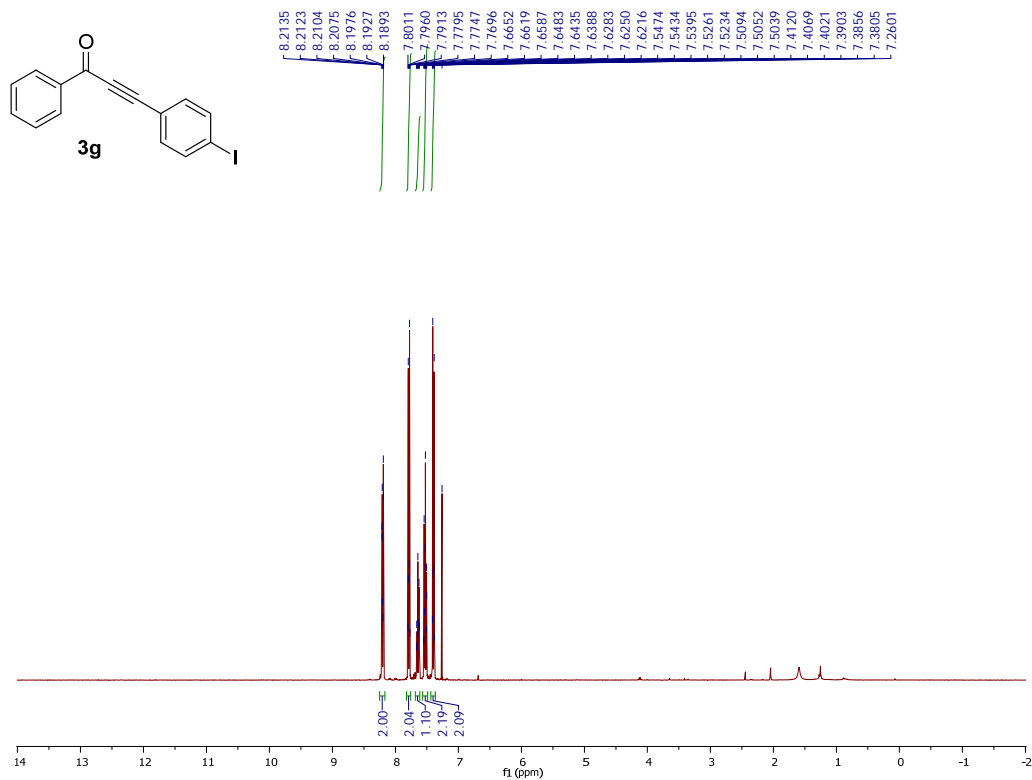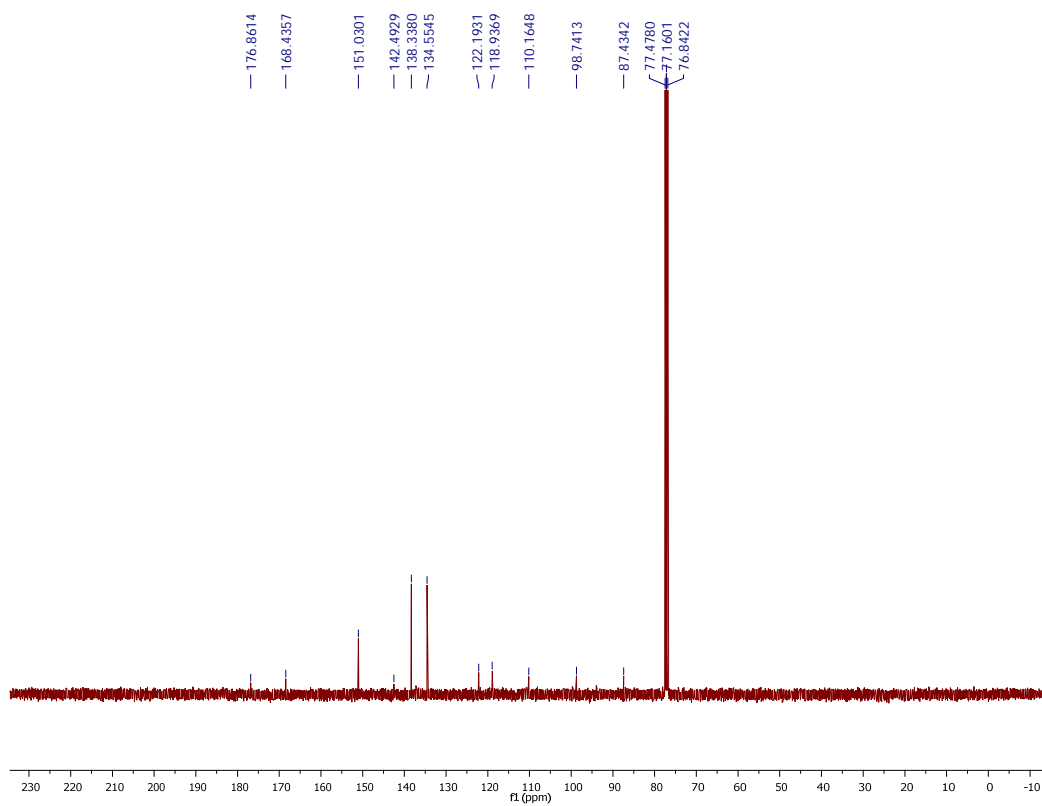

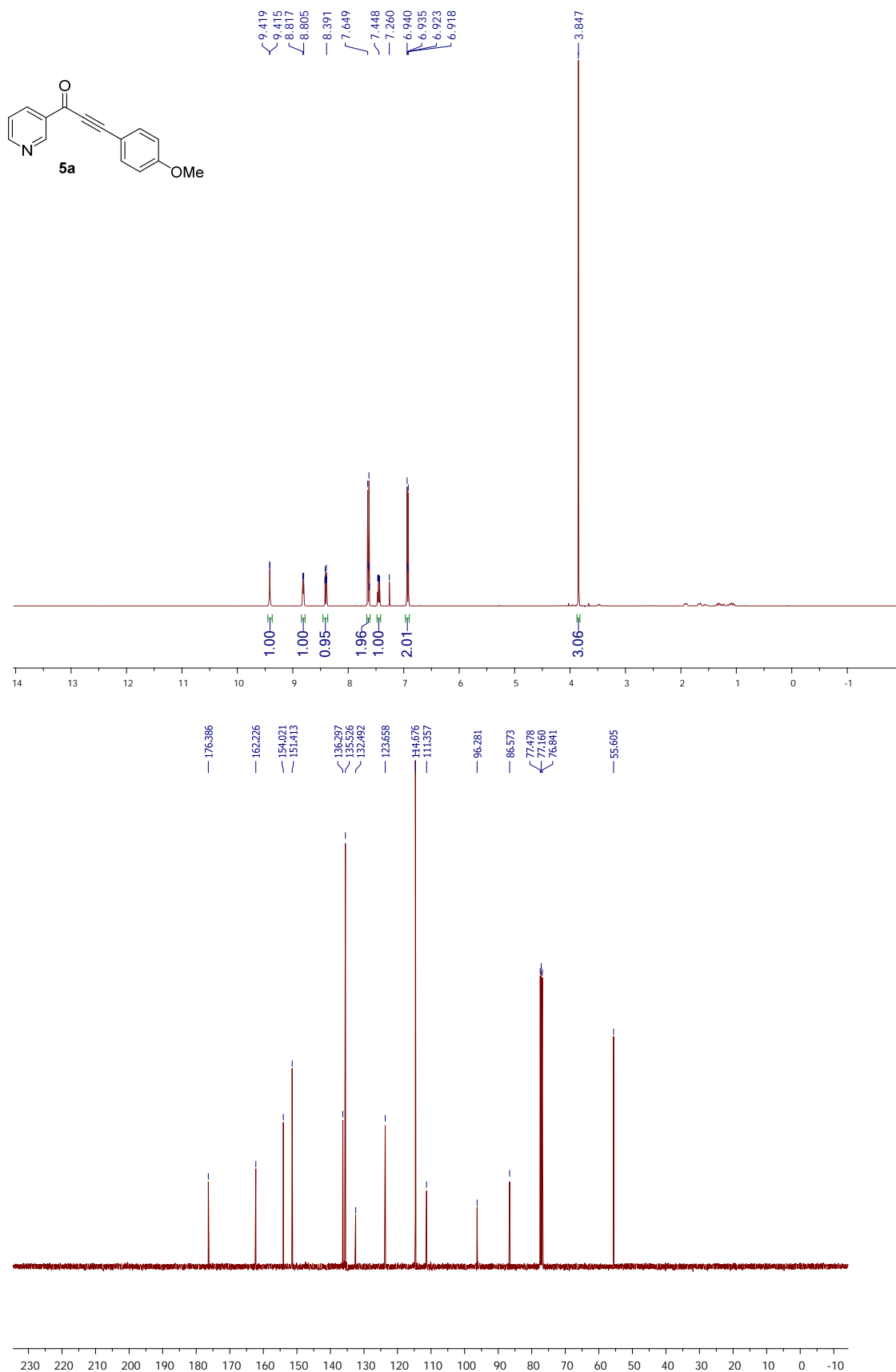

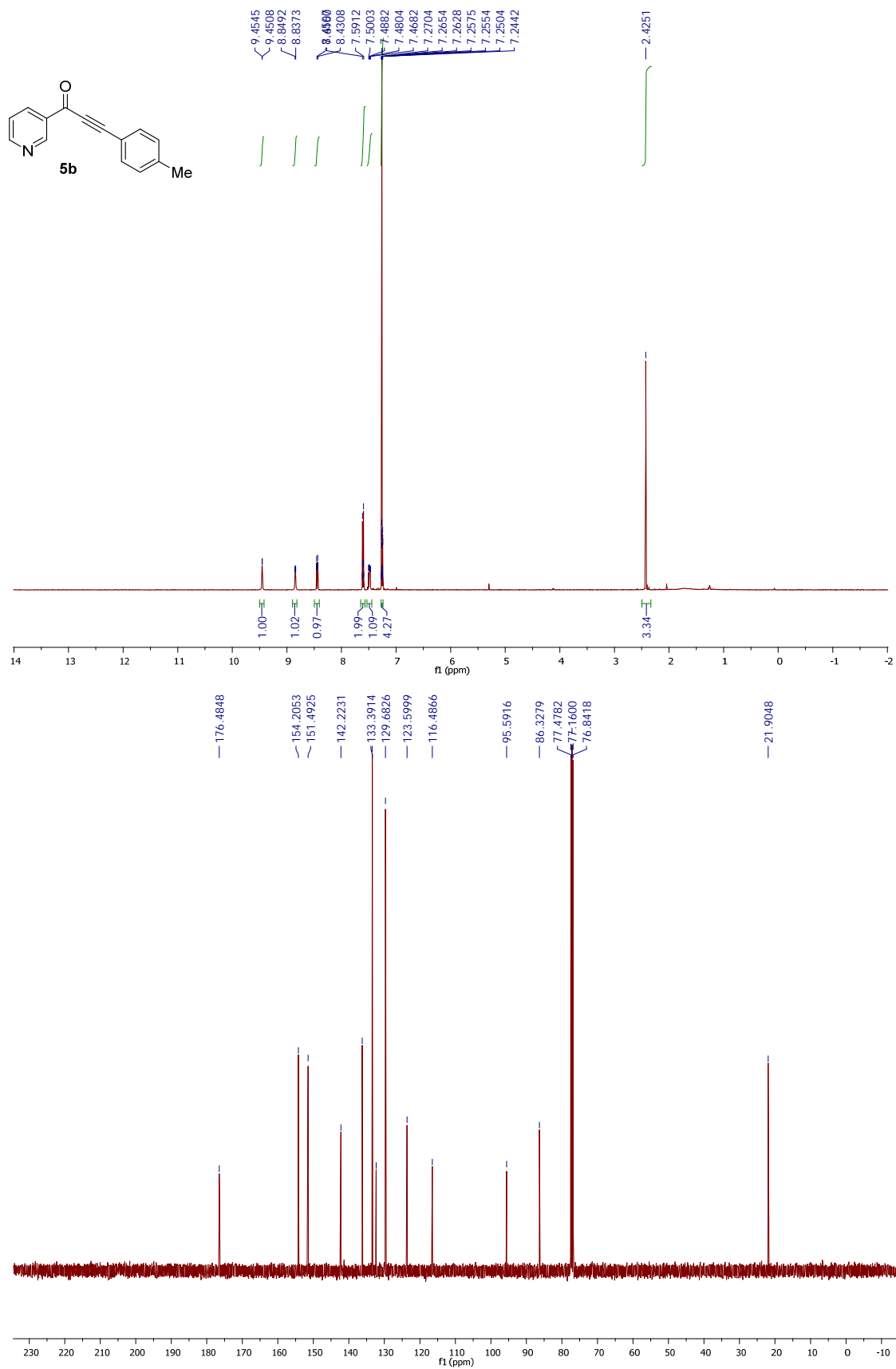

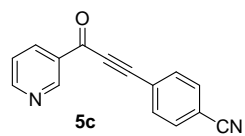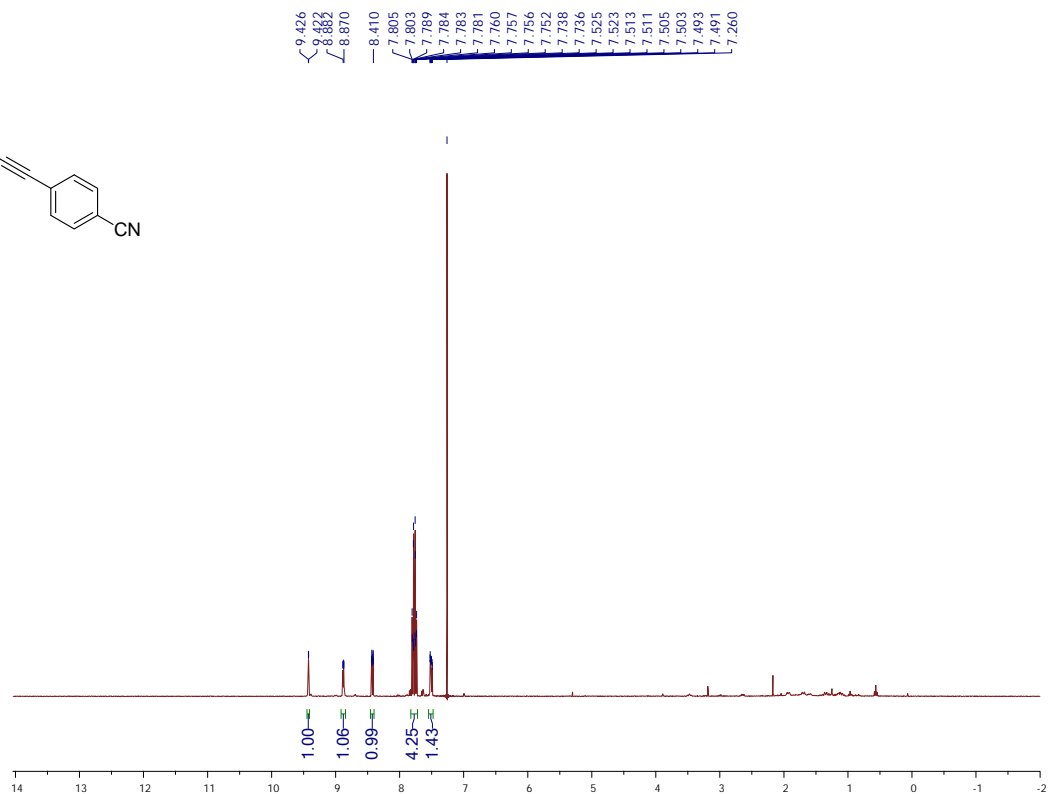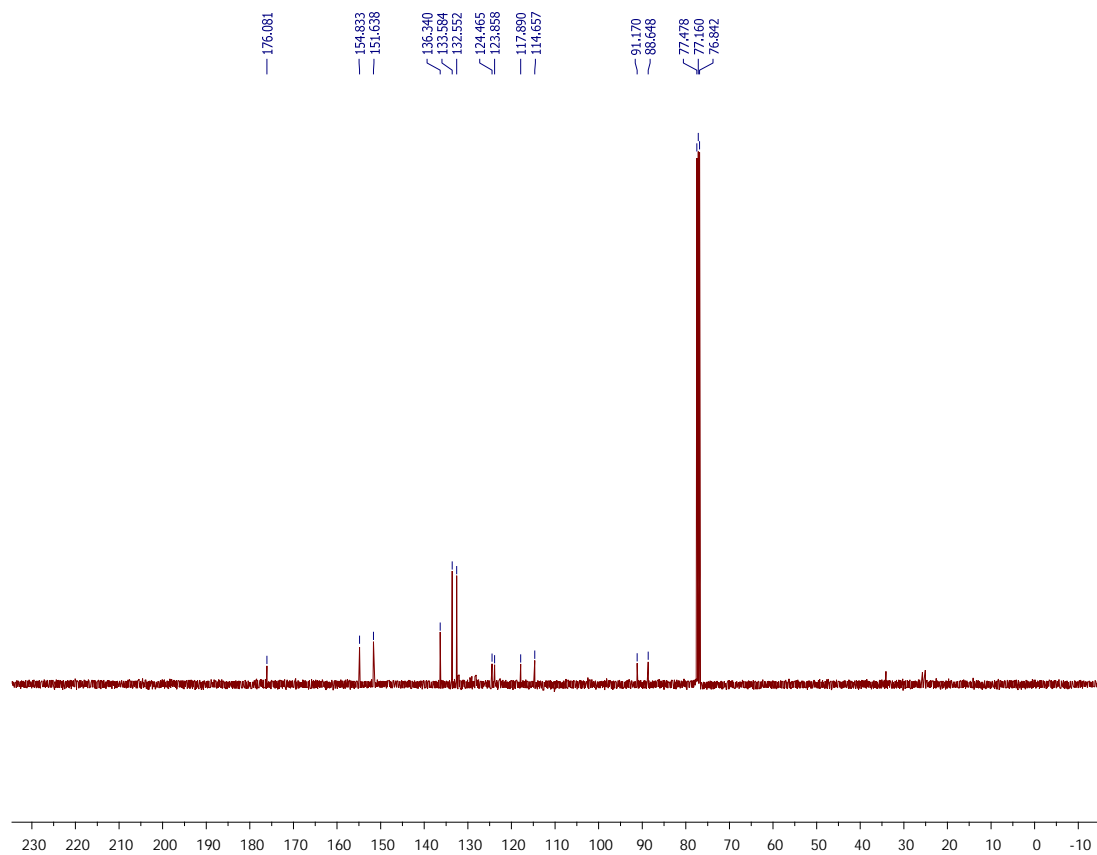

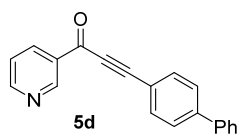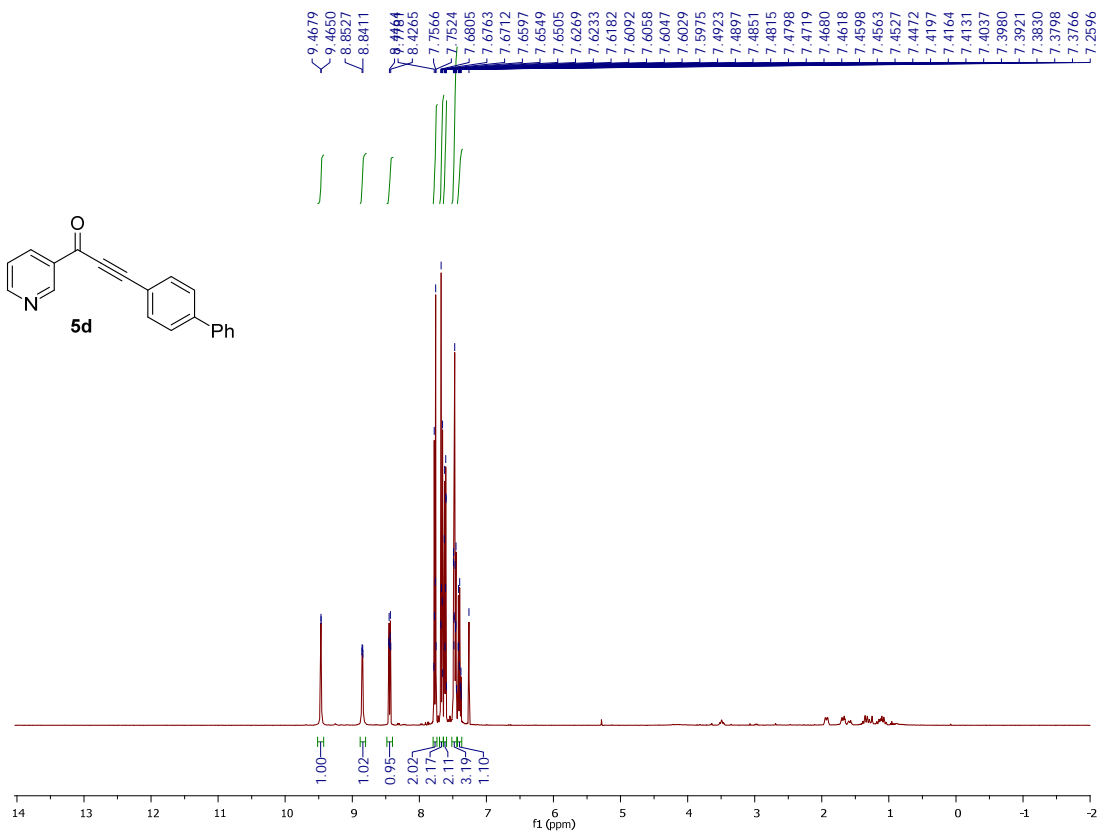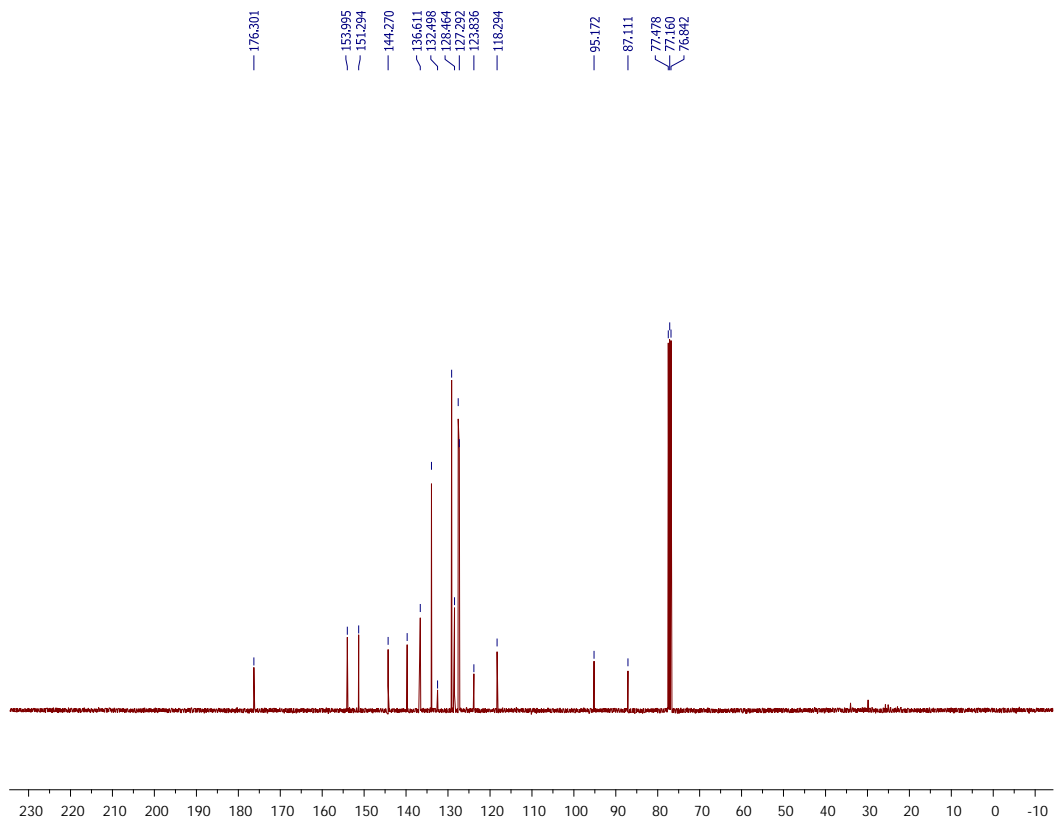

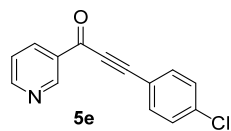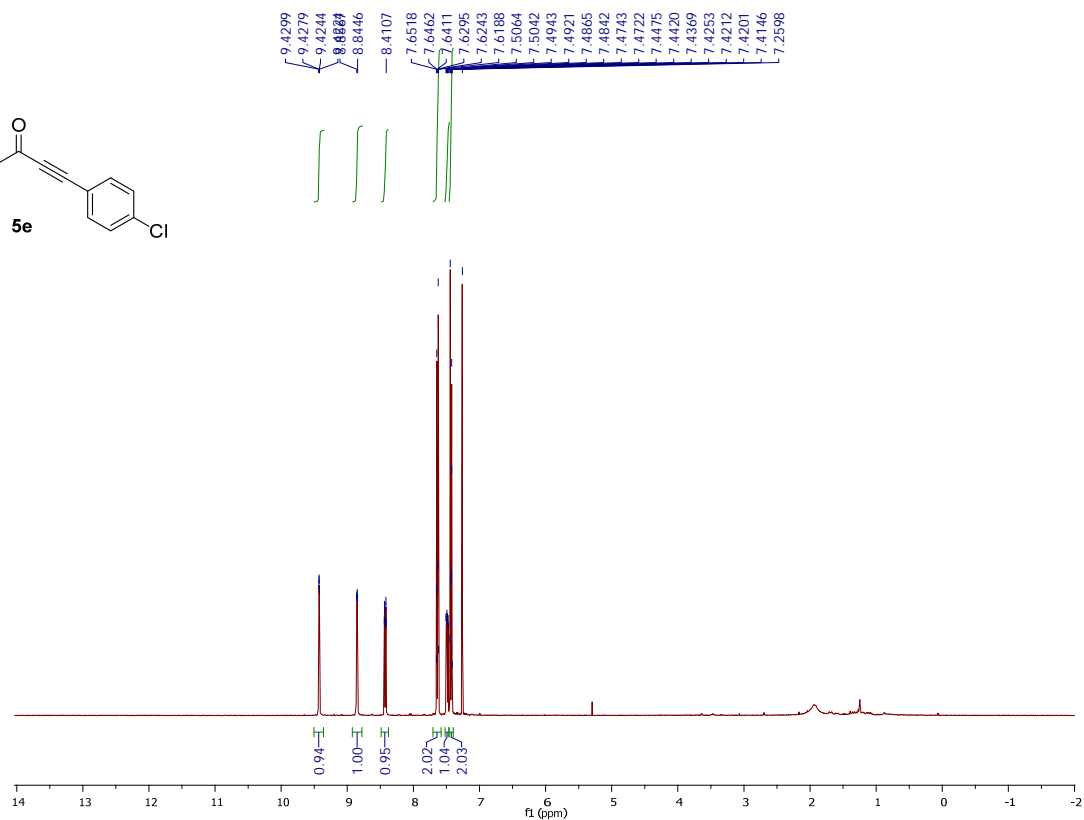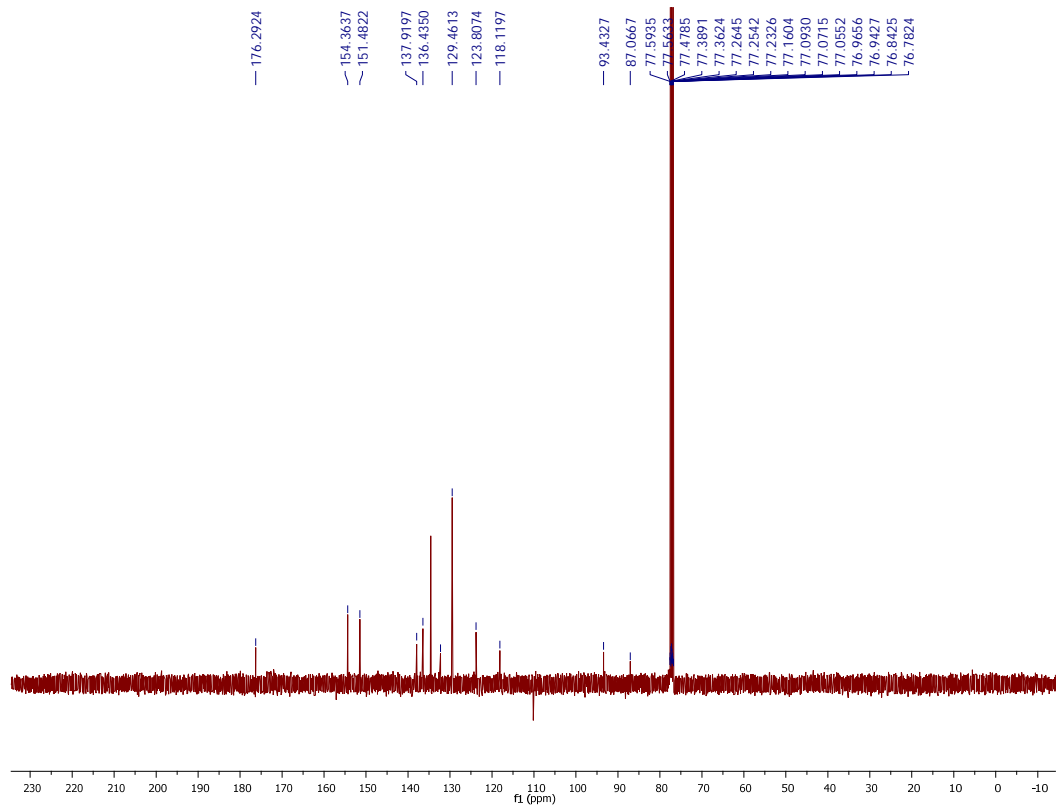

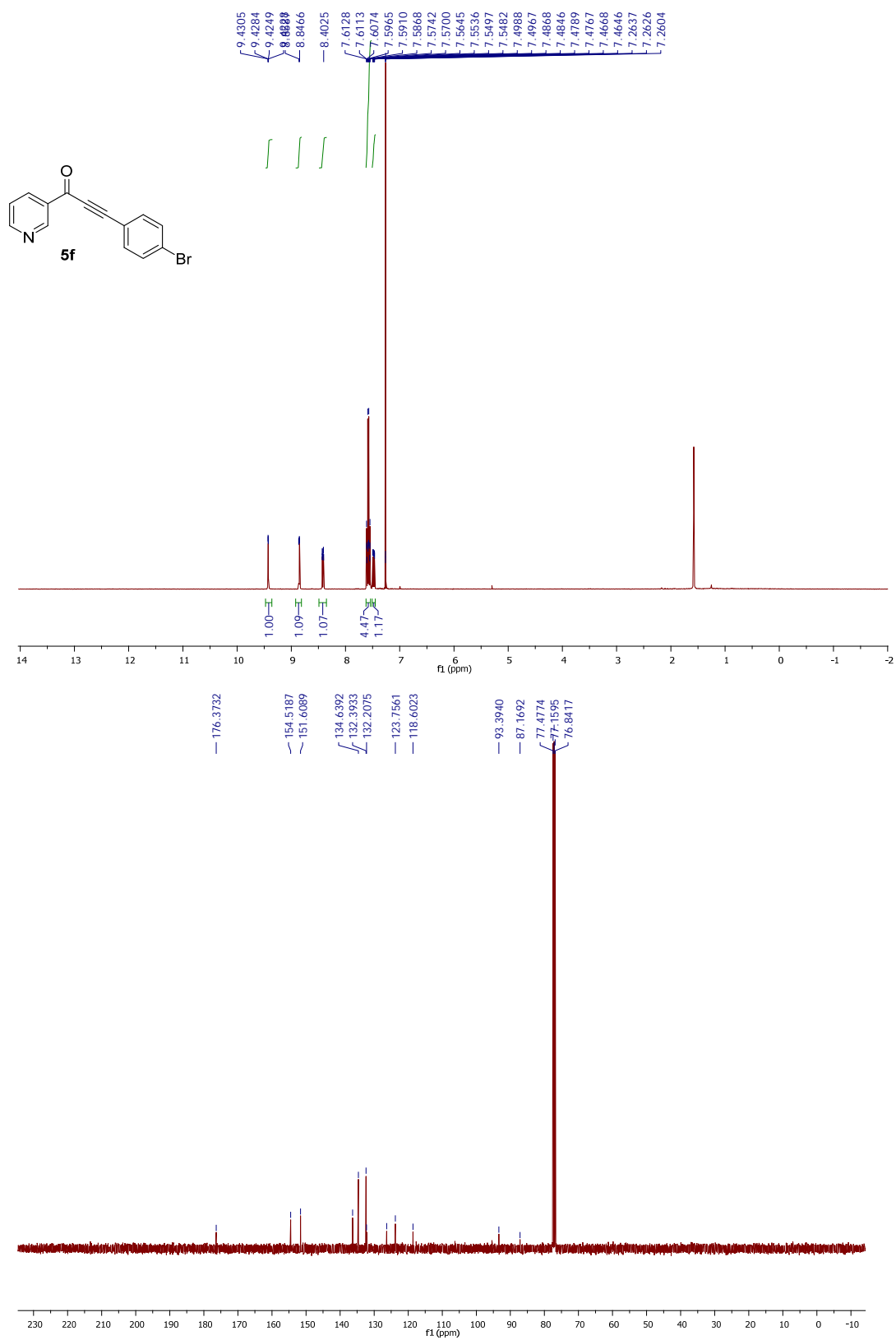

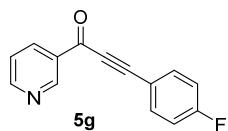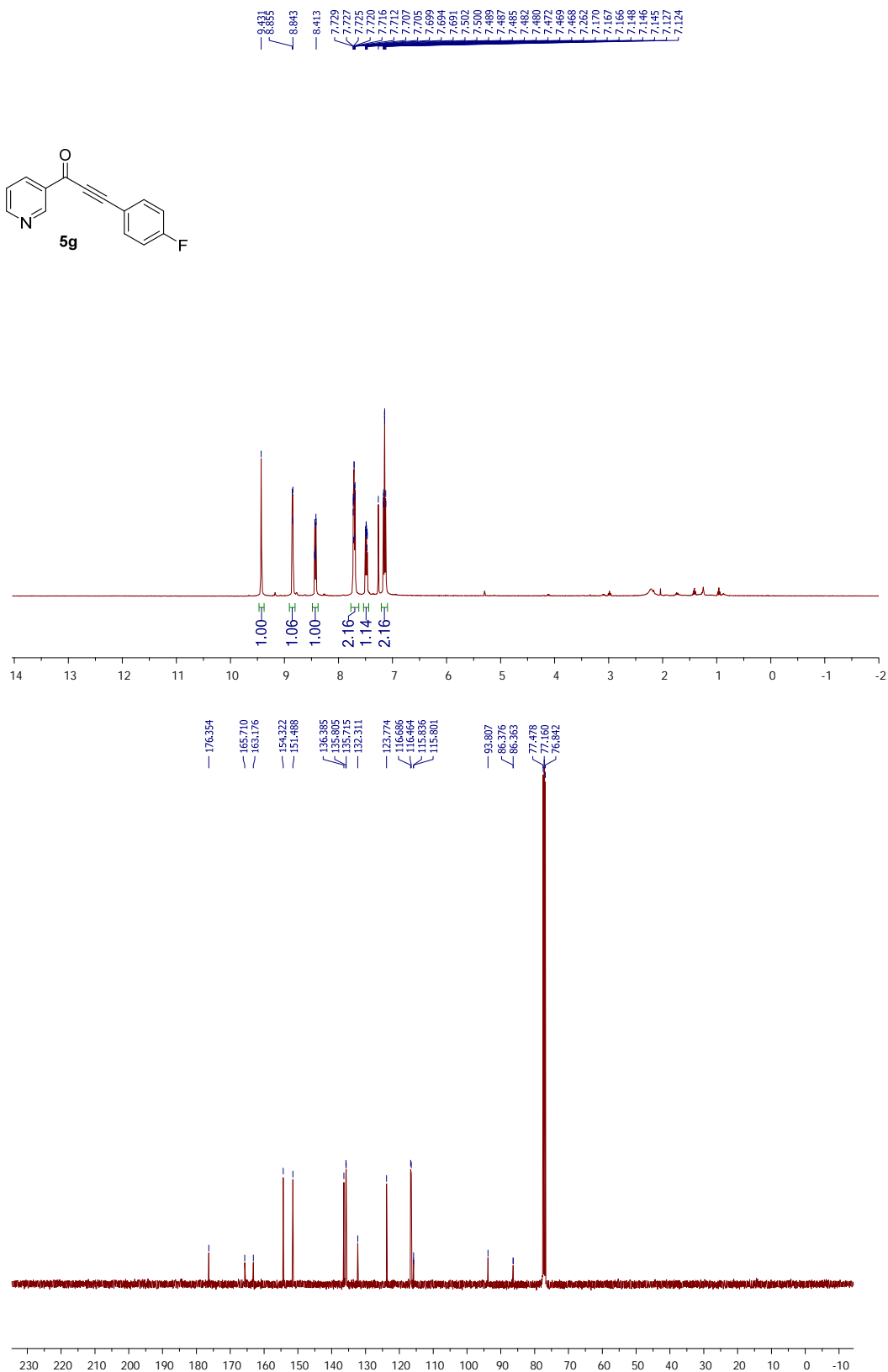

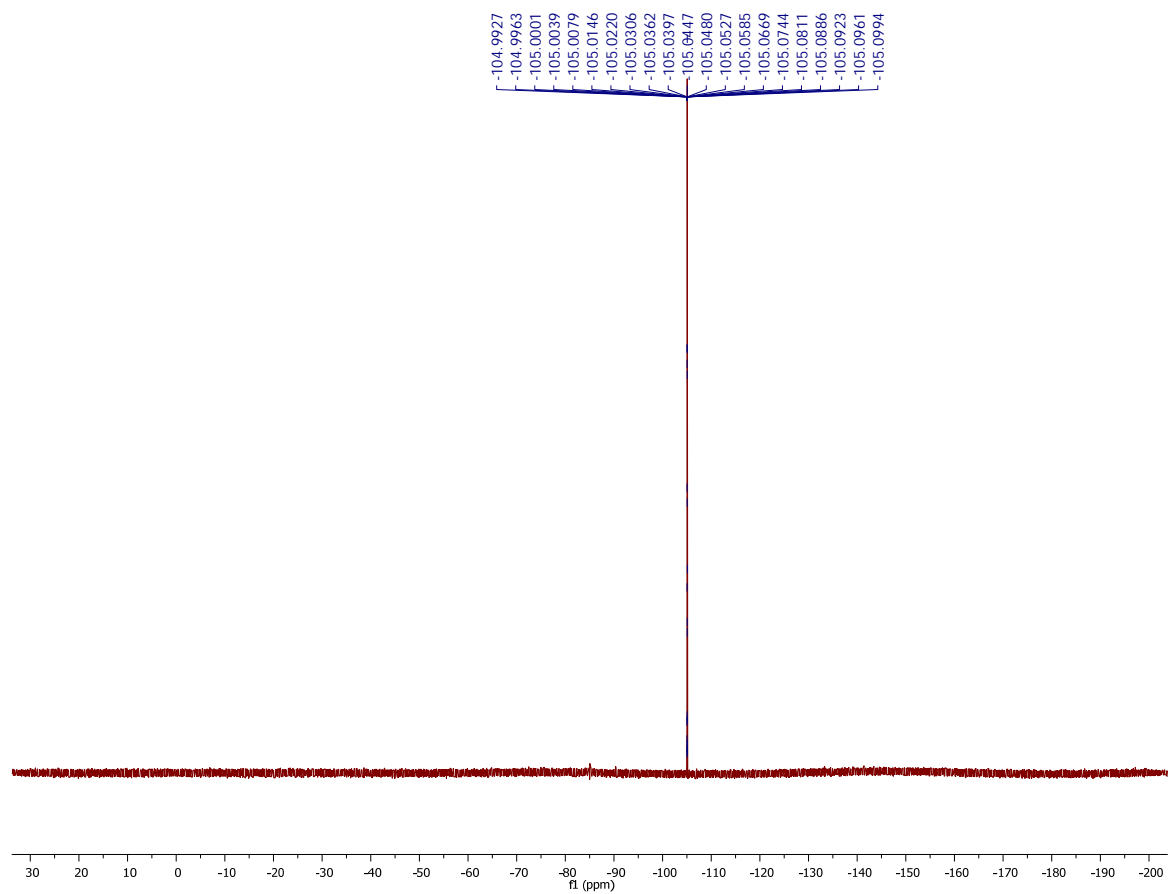

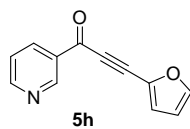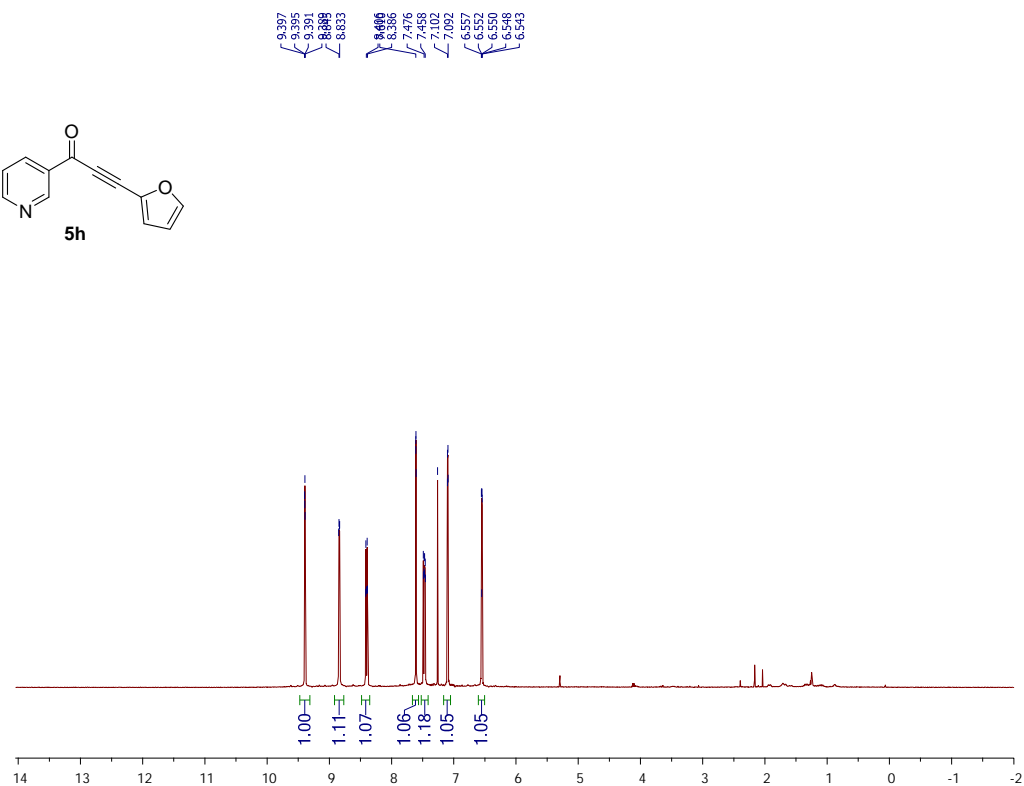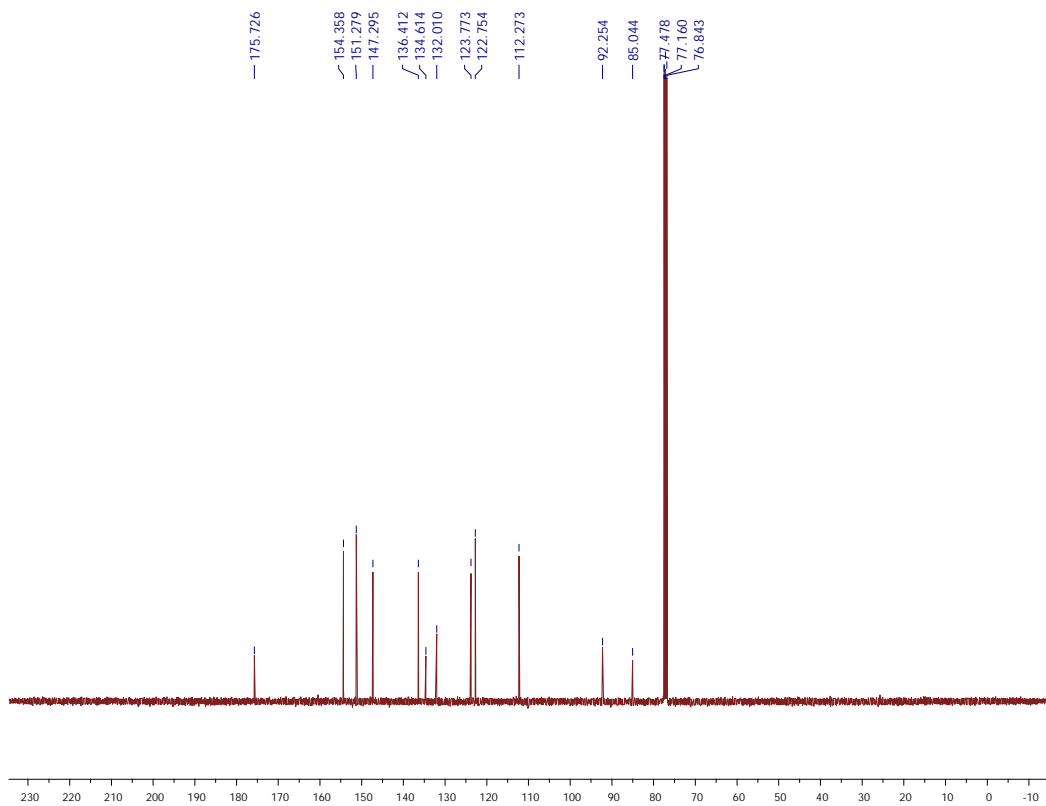

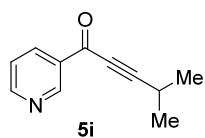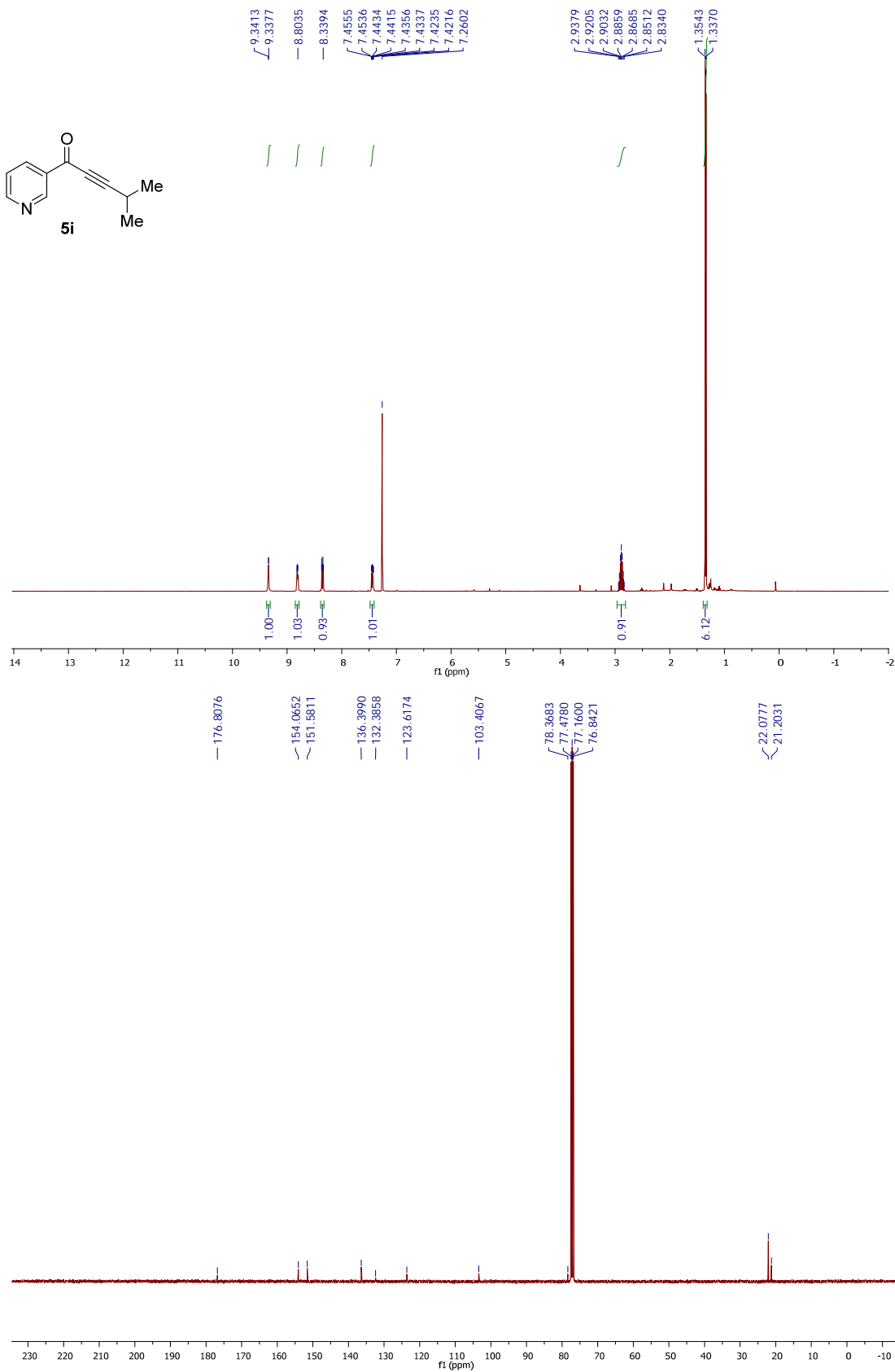

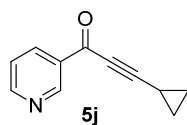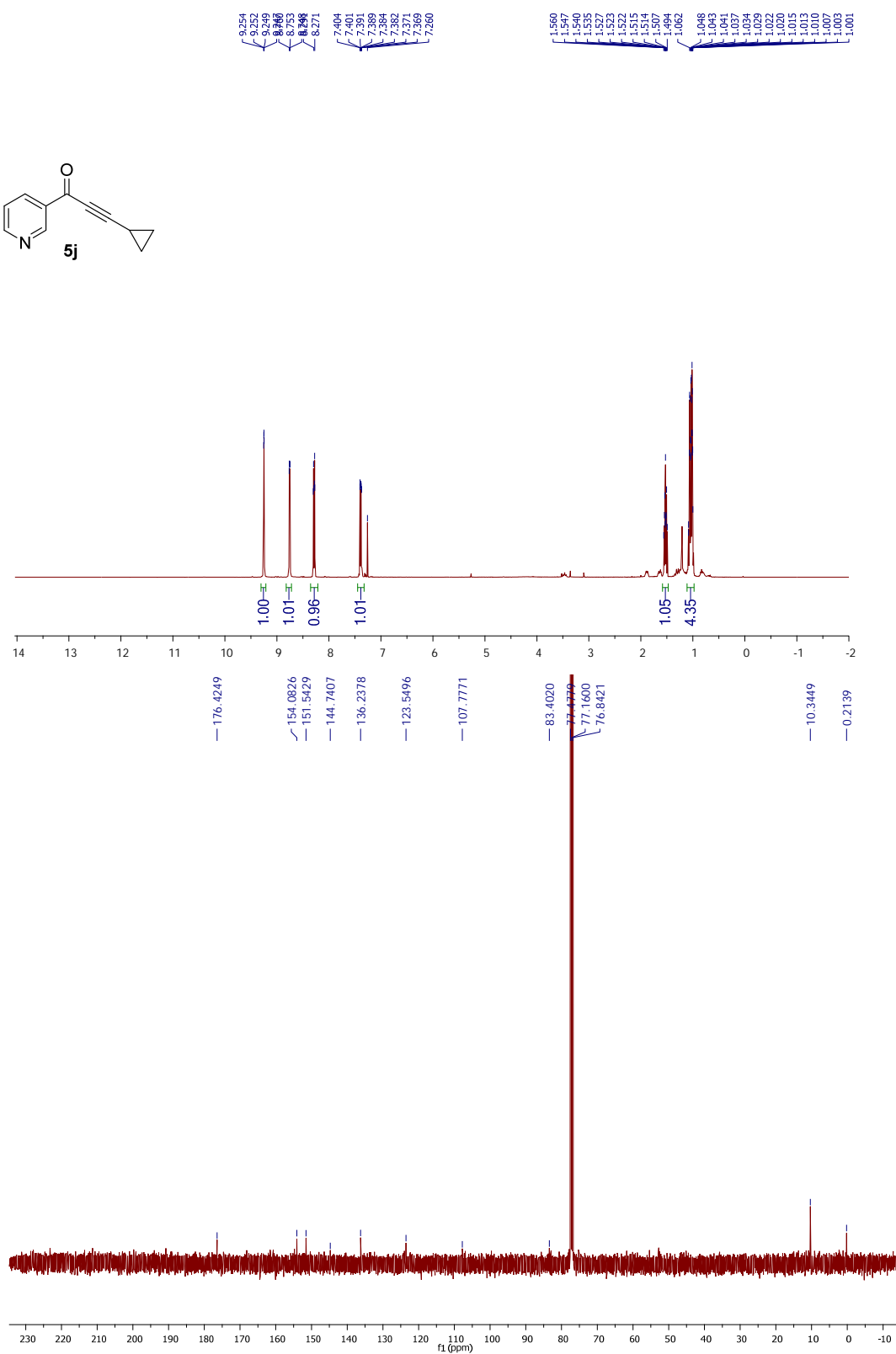

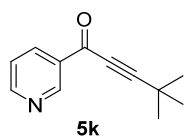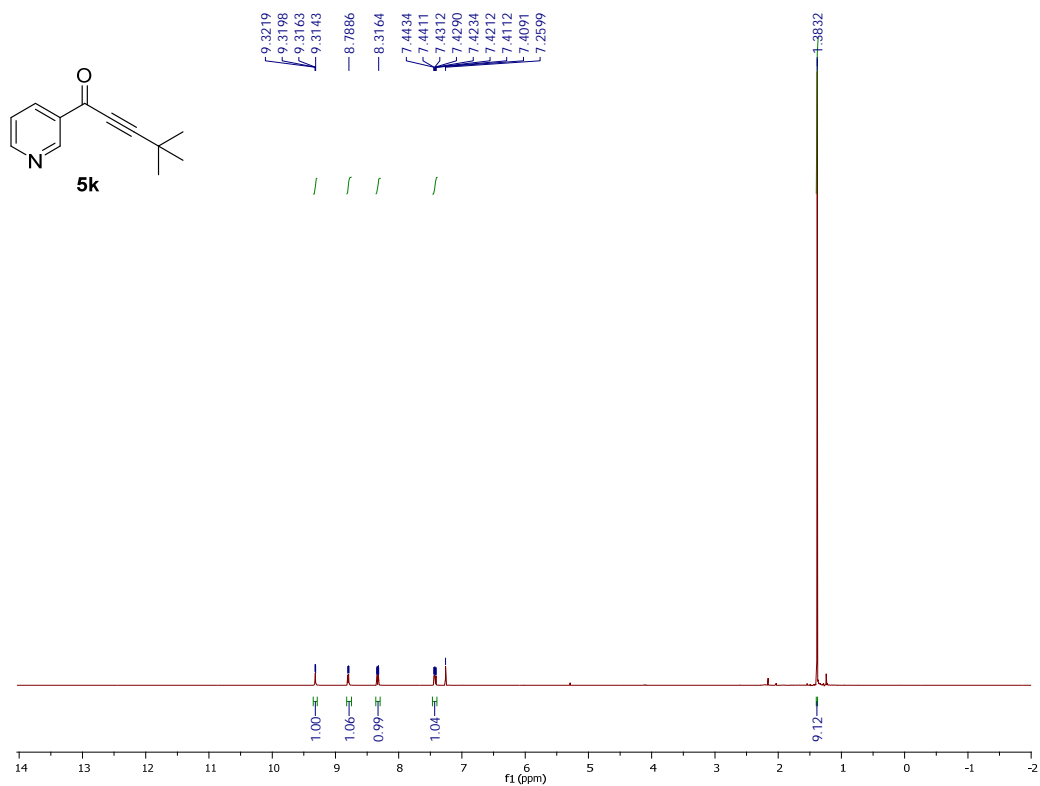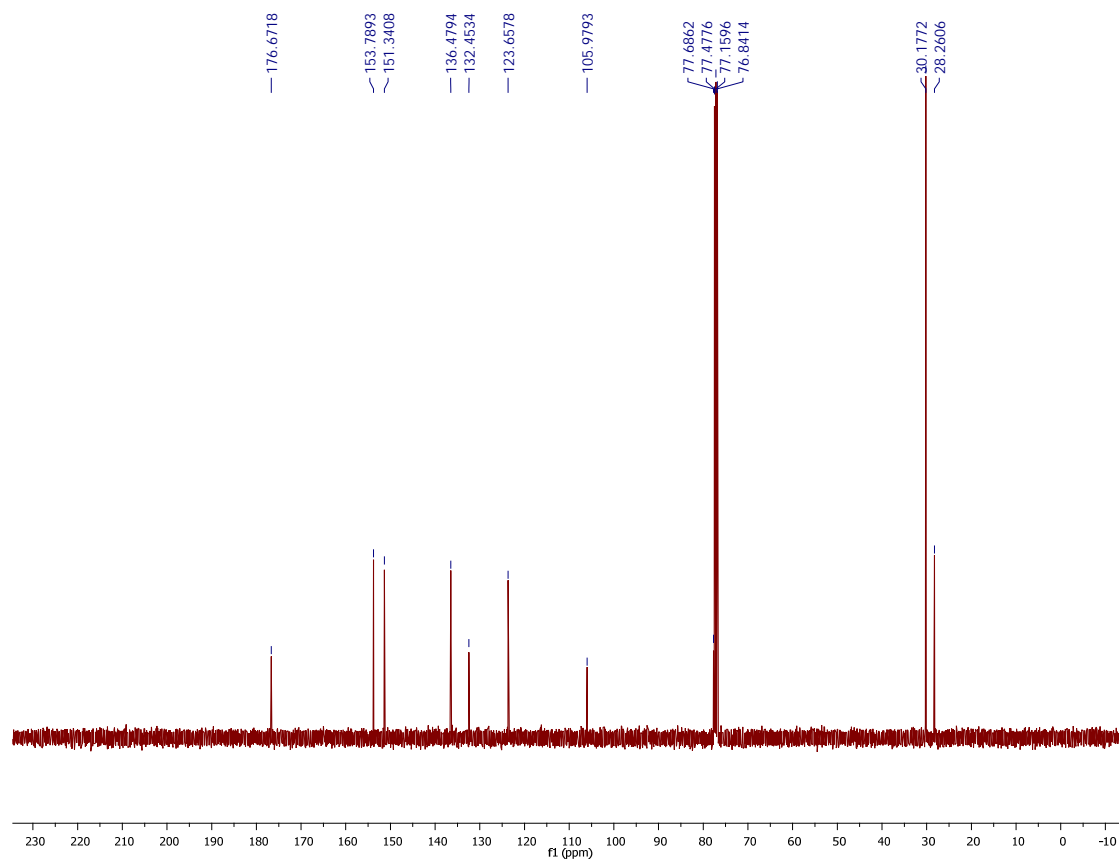

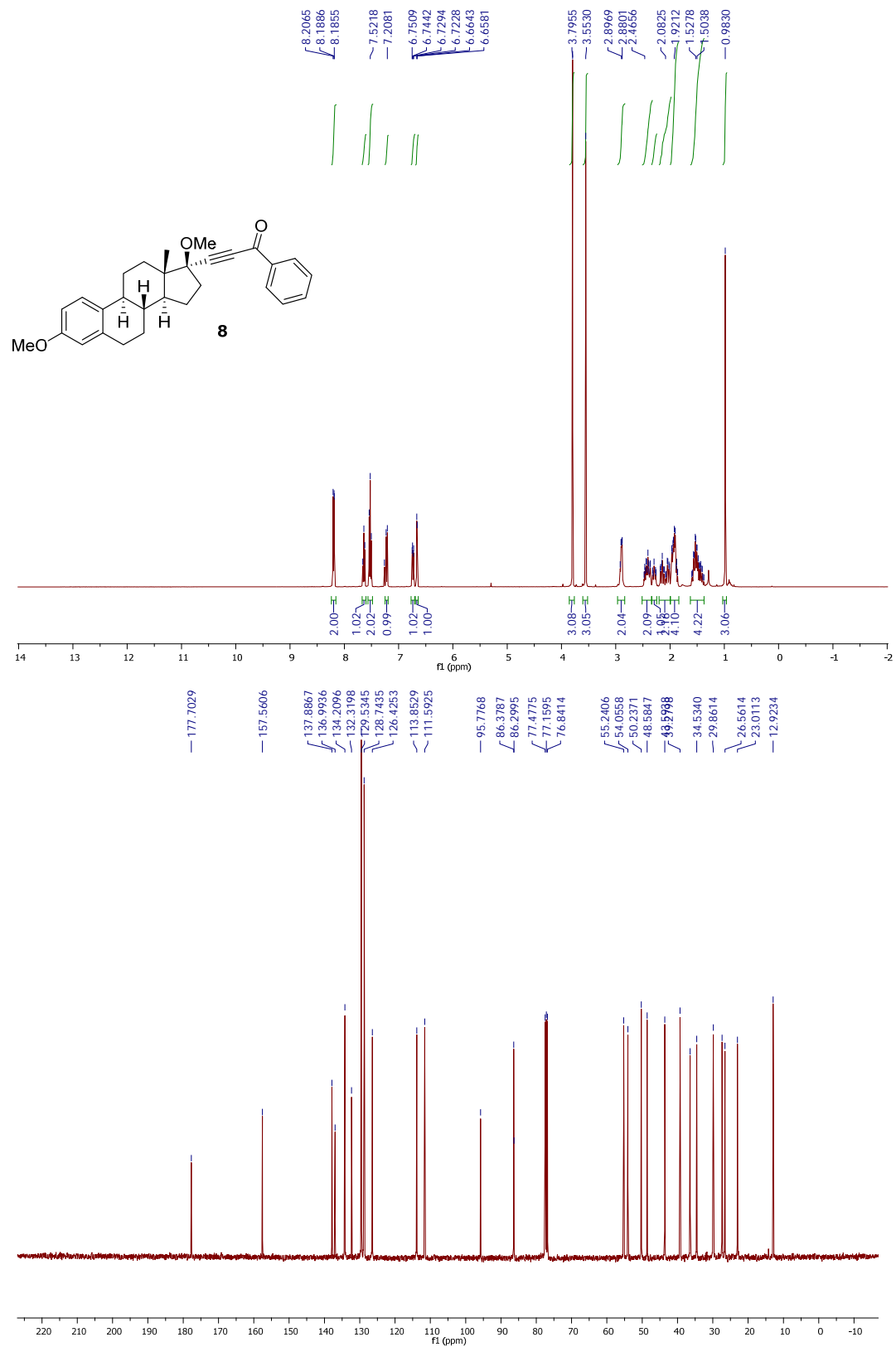

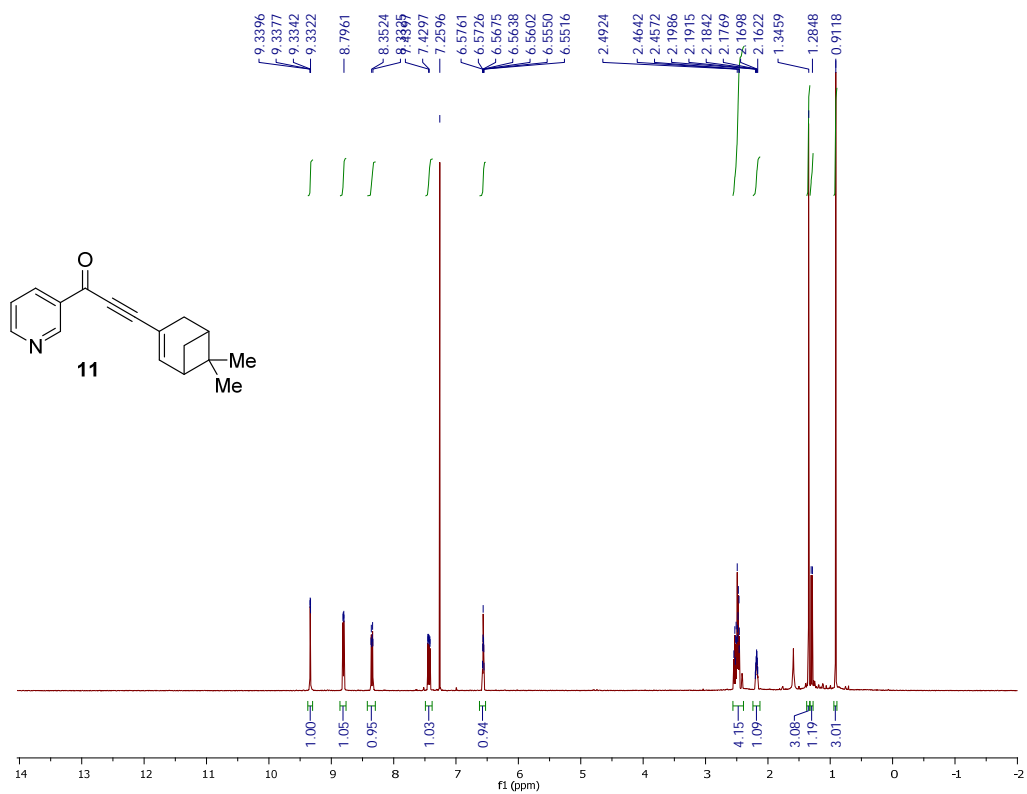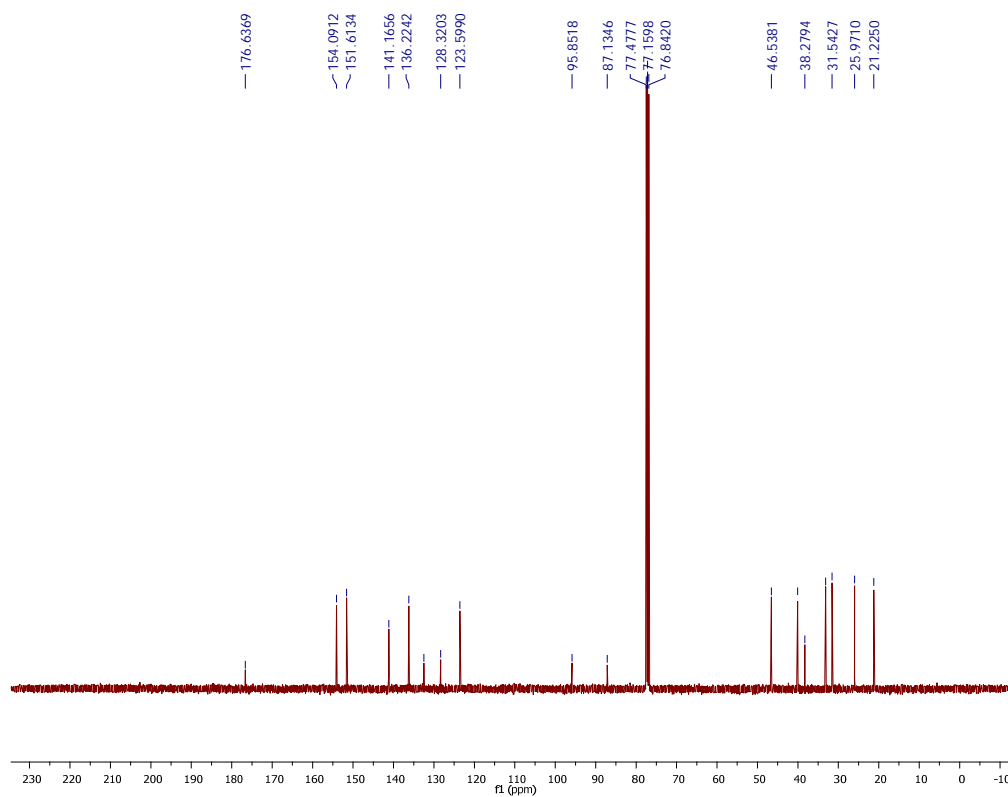

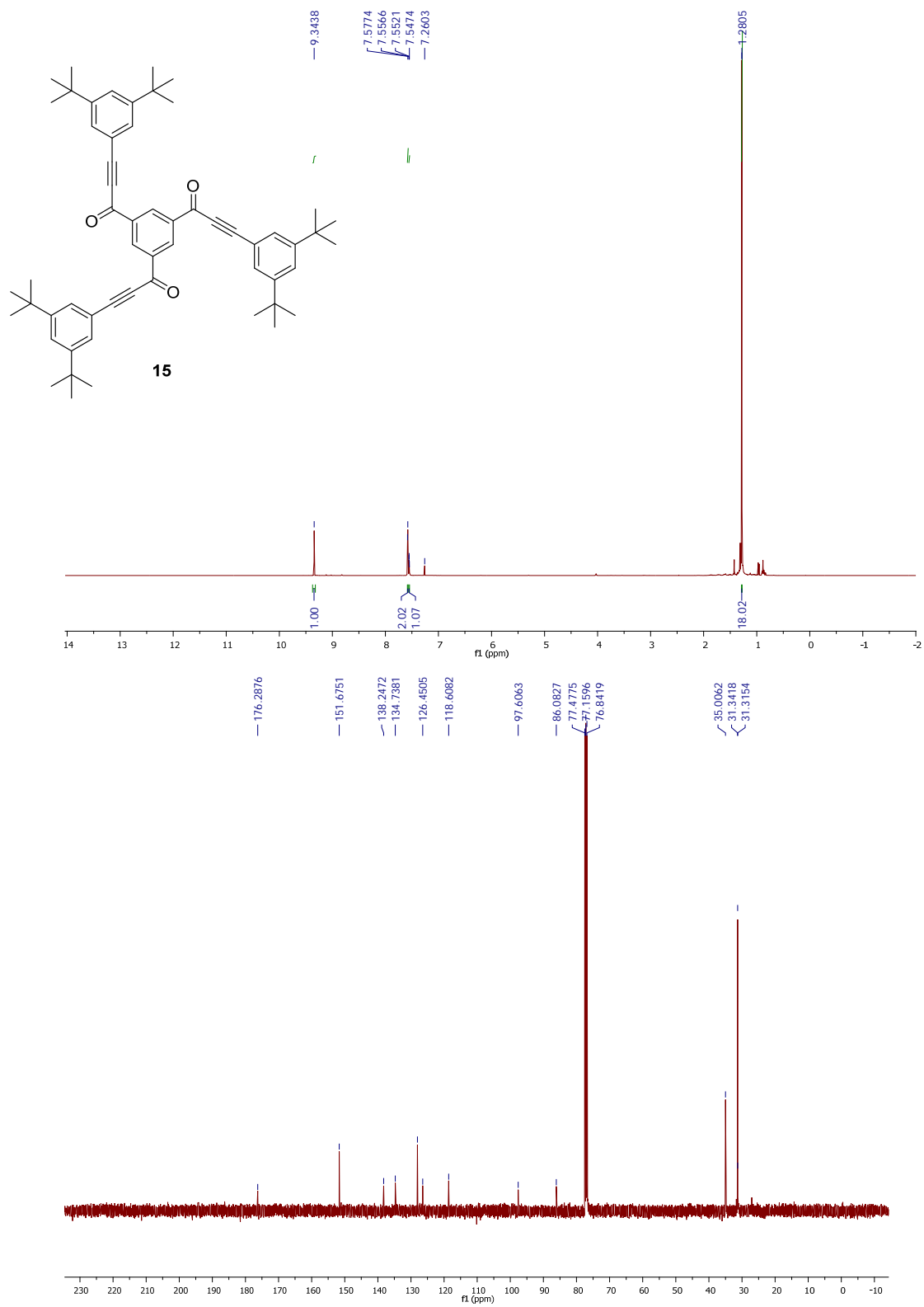

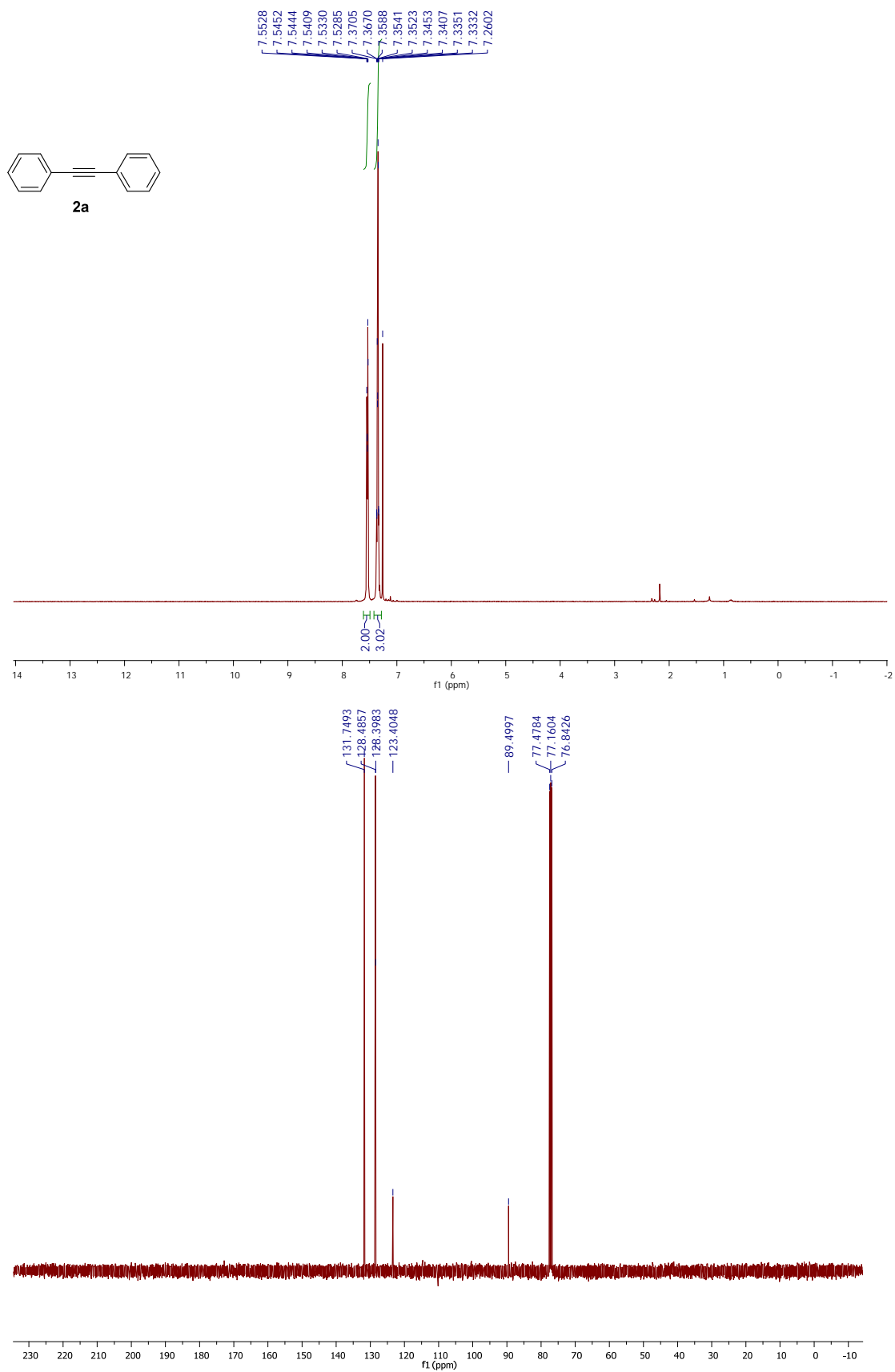

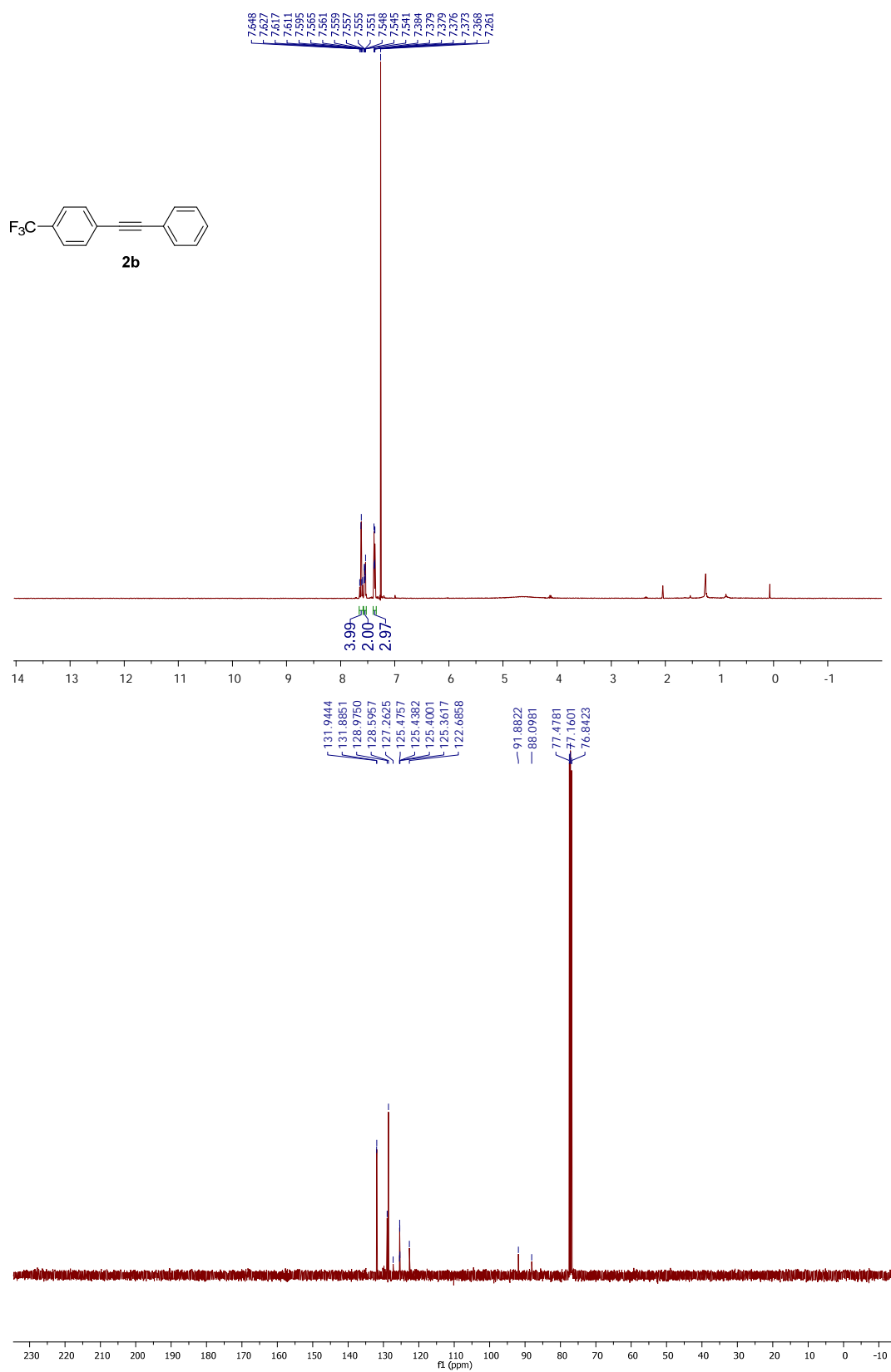

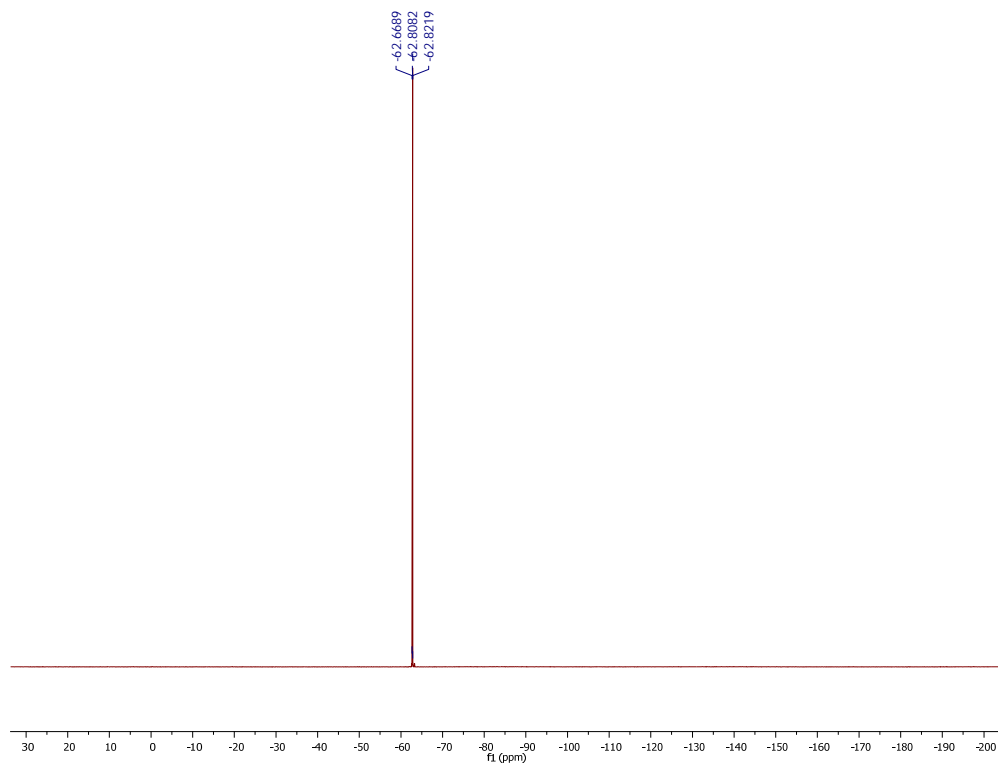

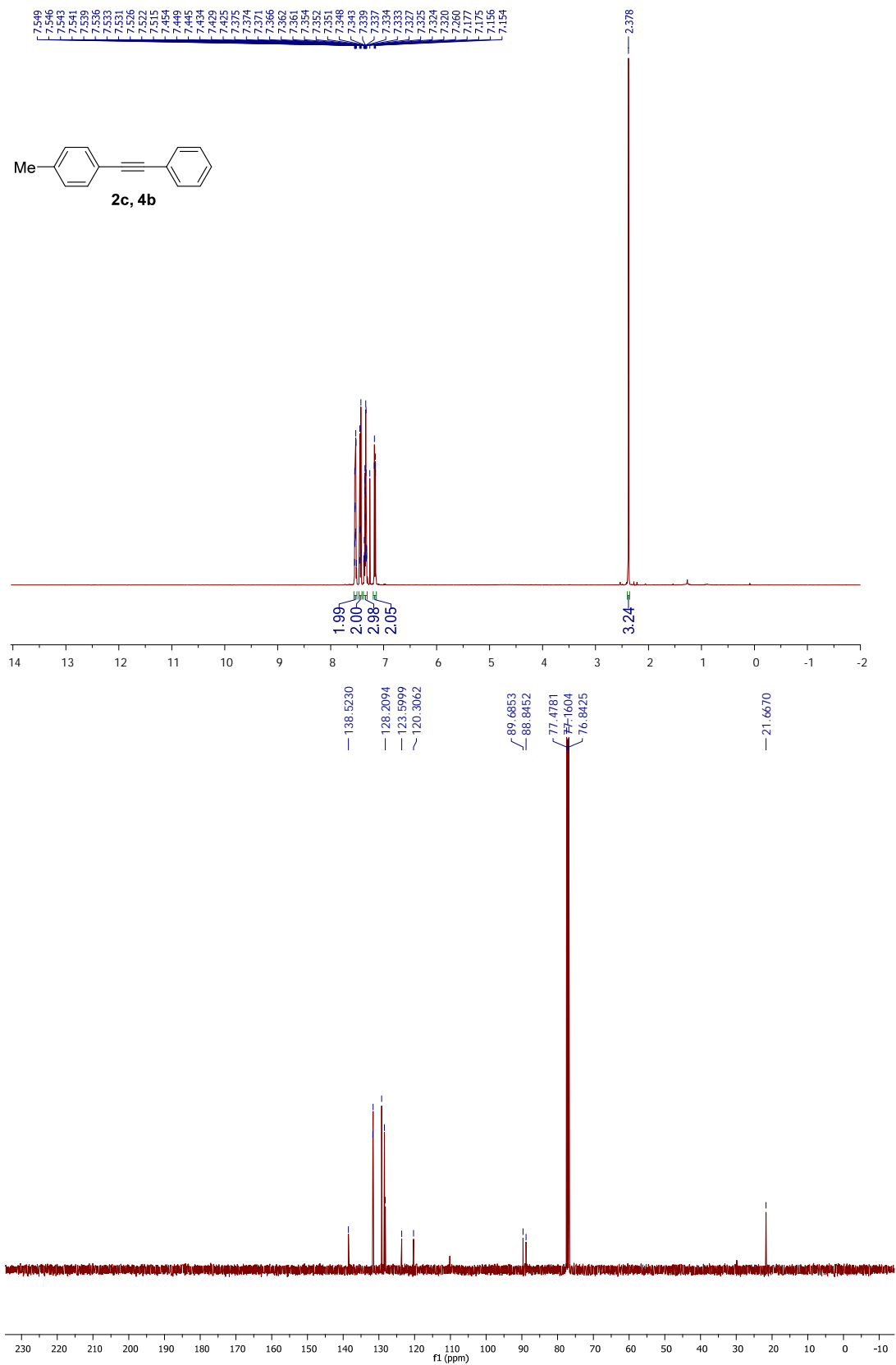

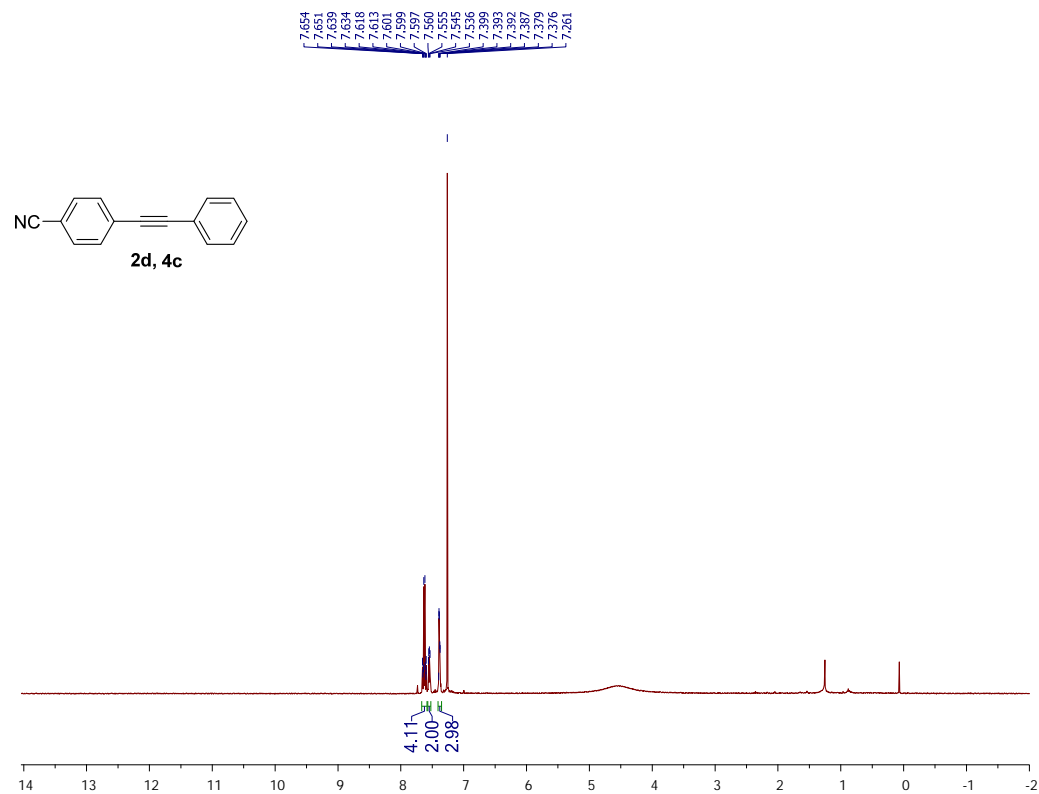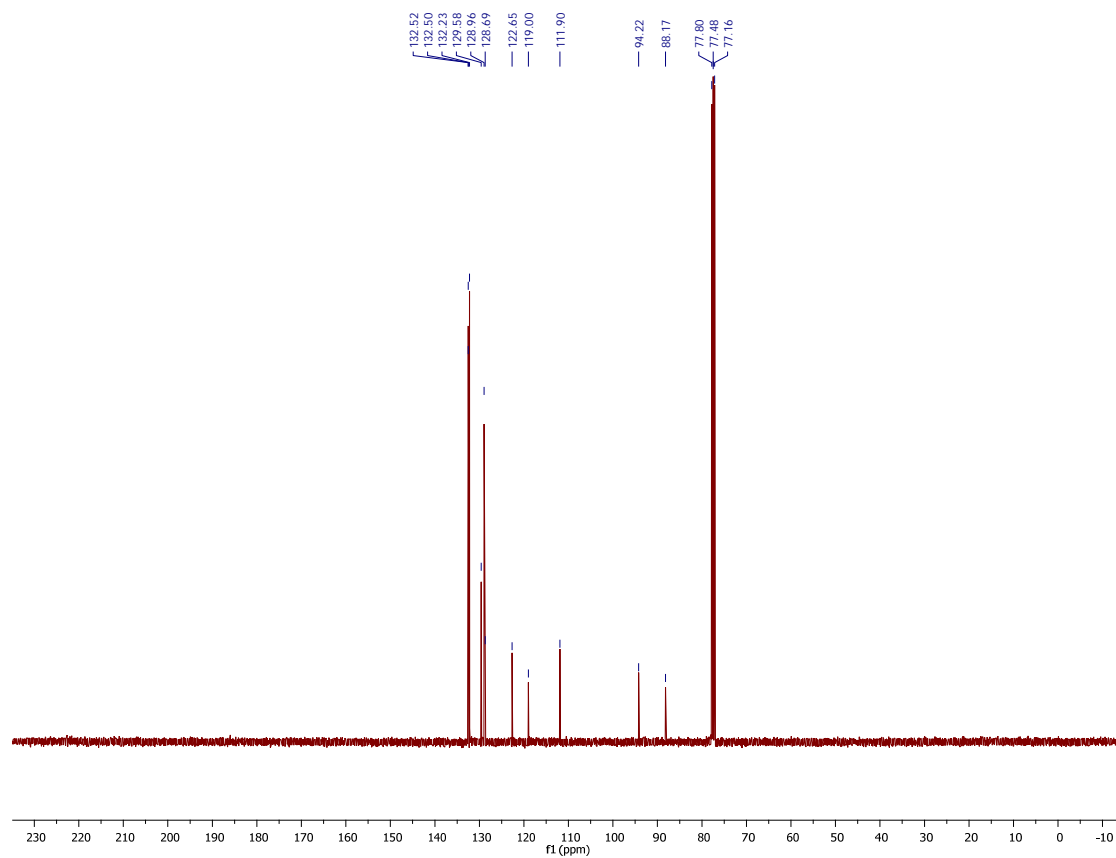

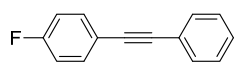

**2e, 4f**

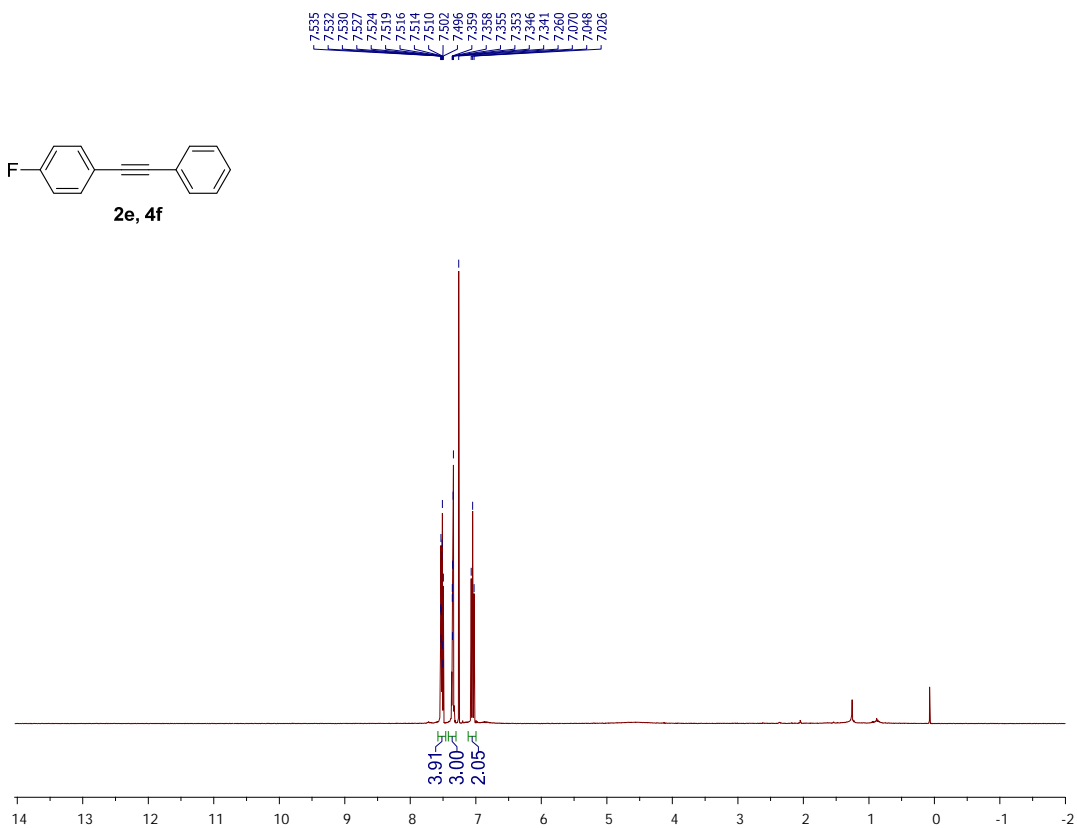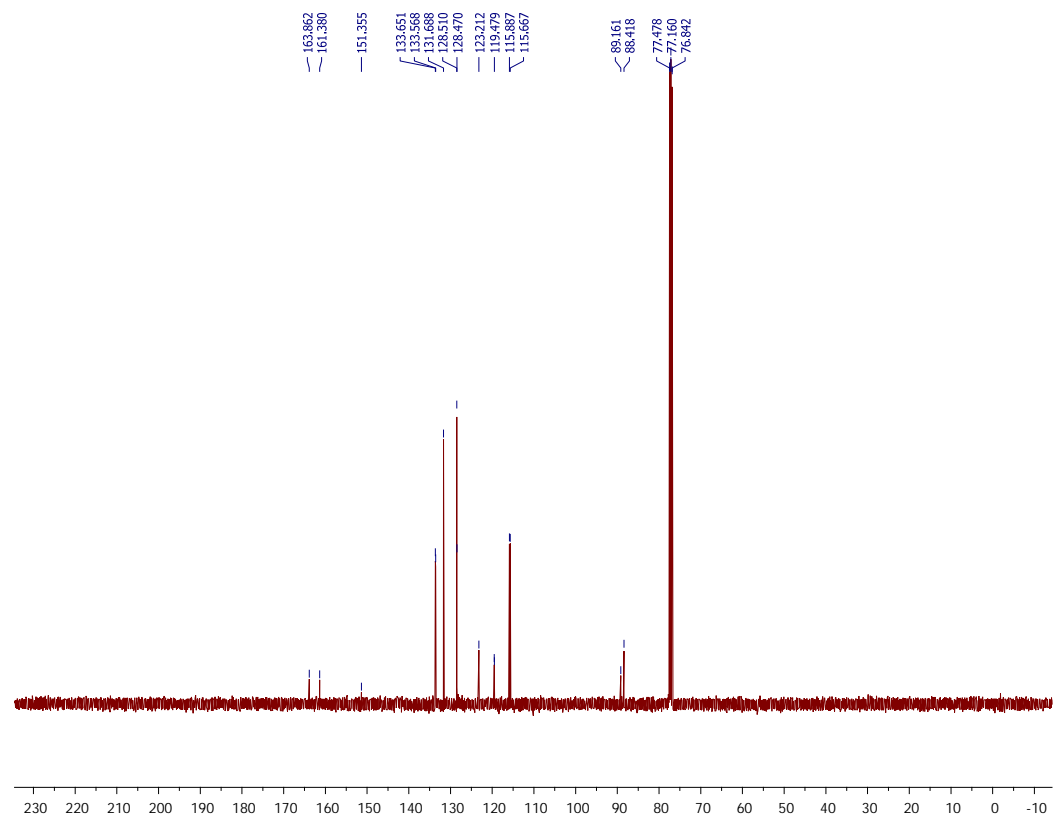

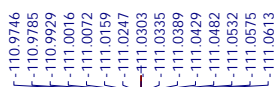

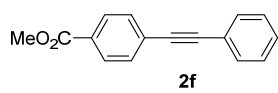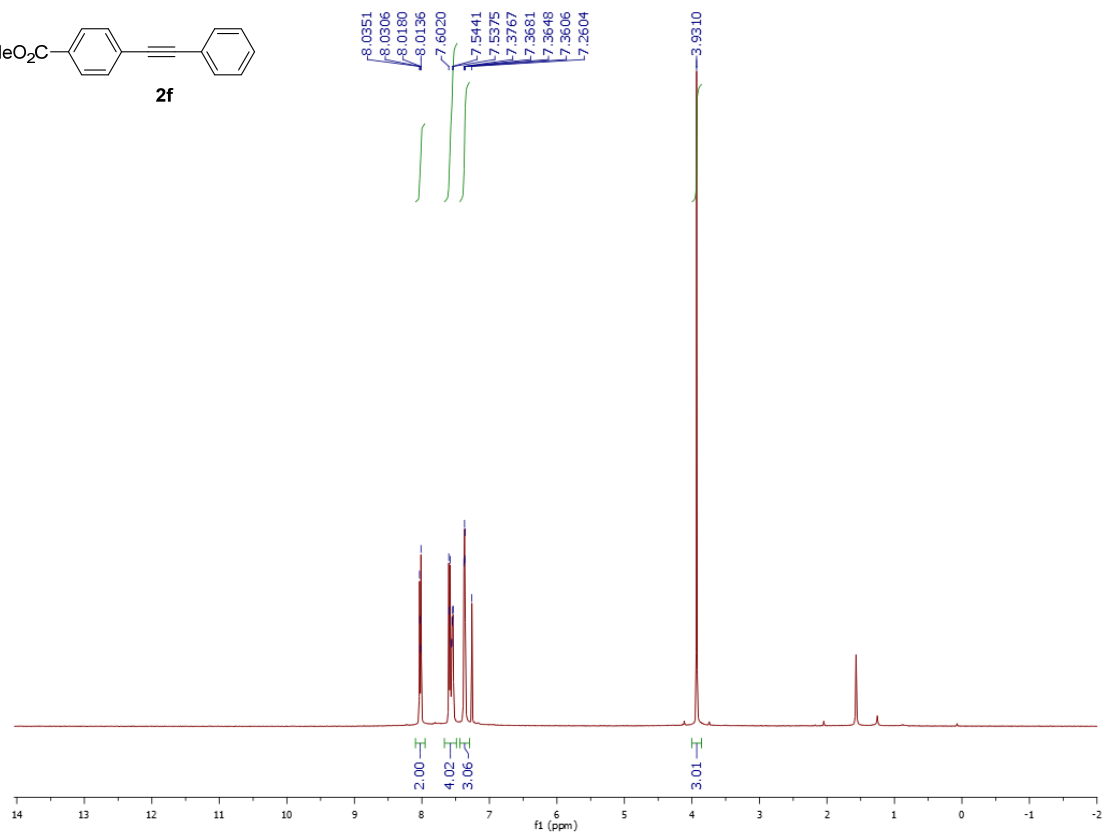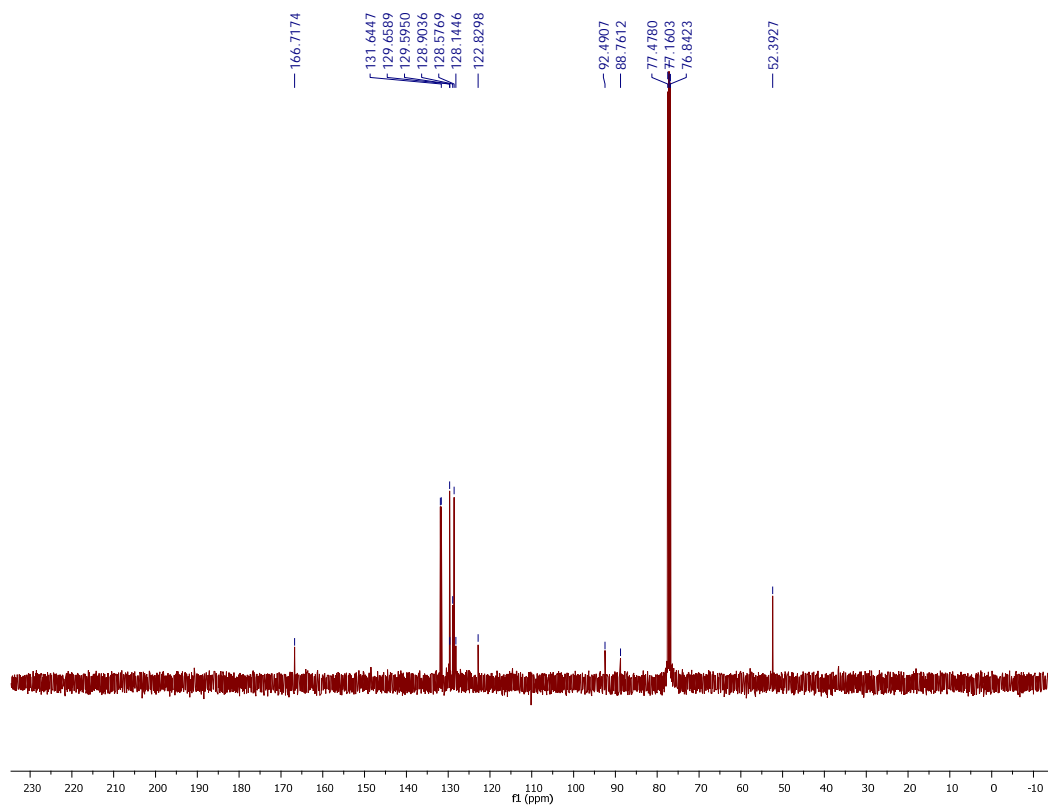

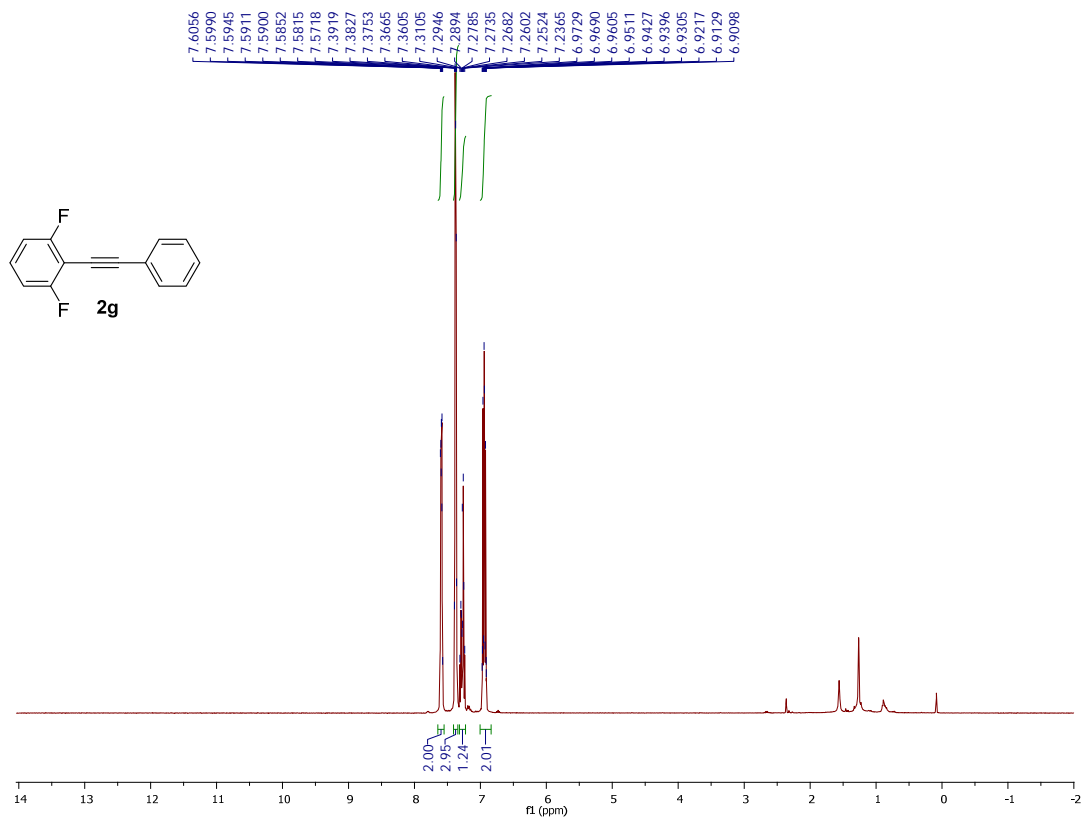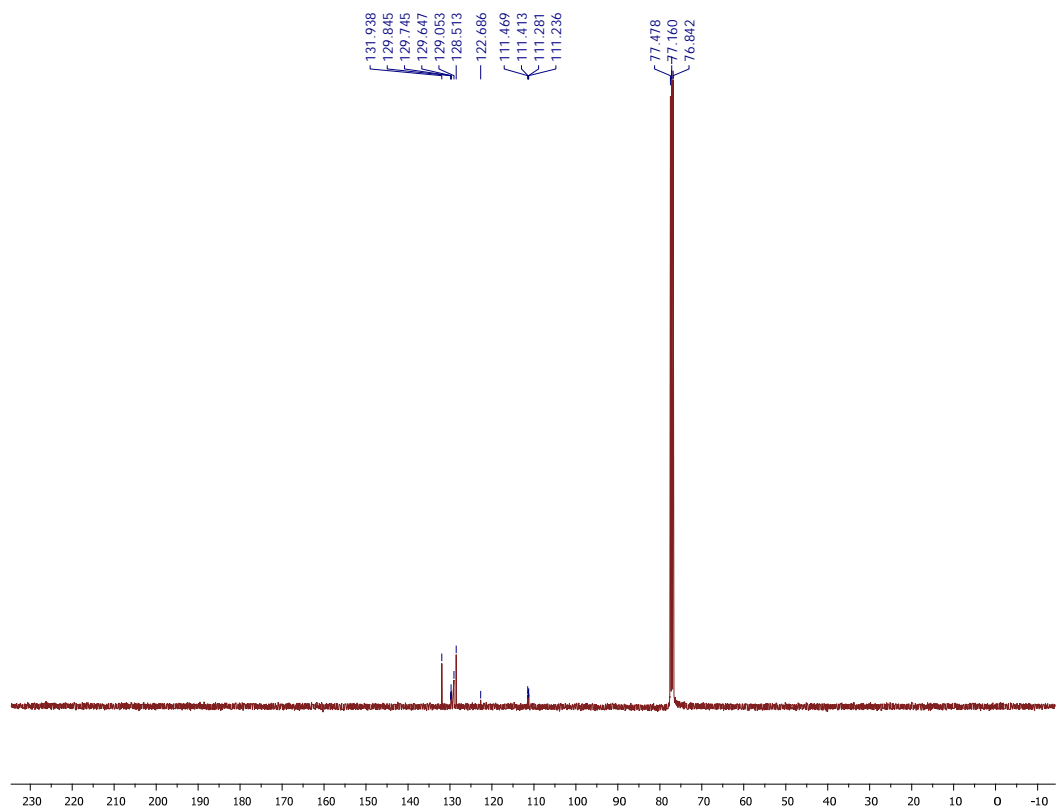

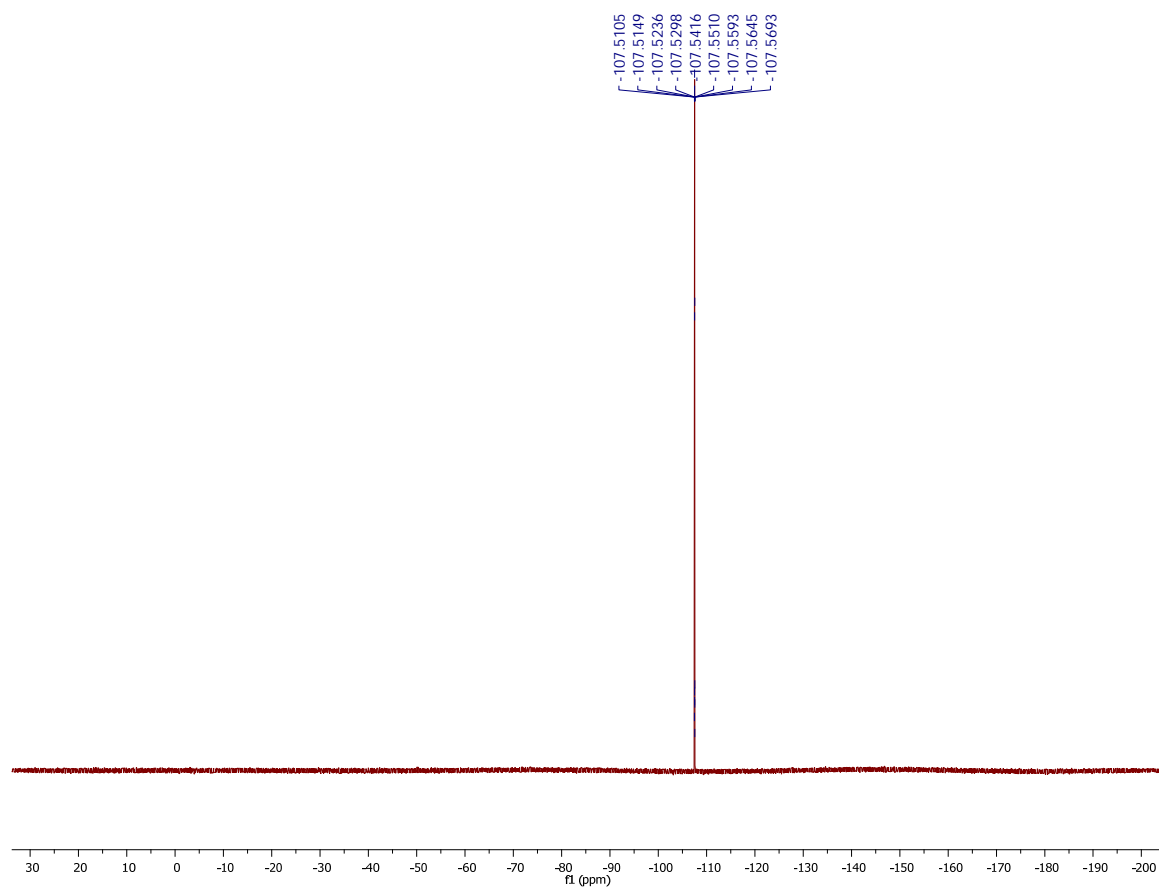

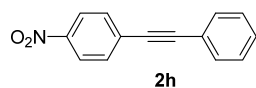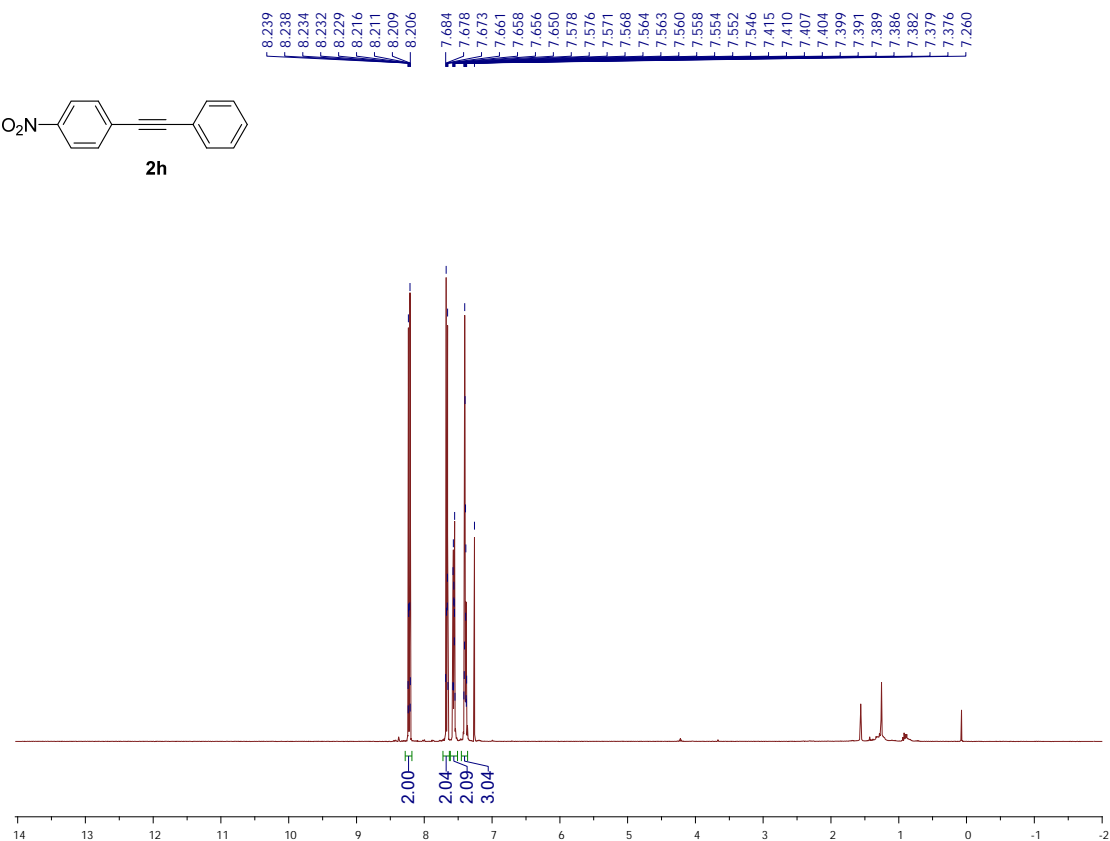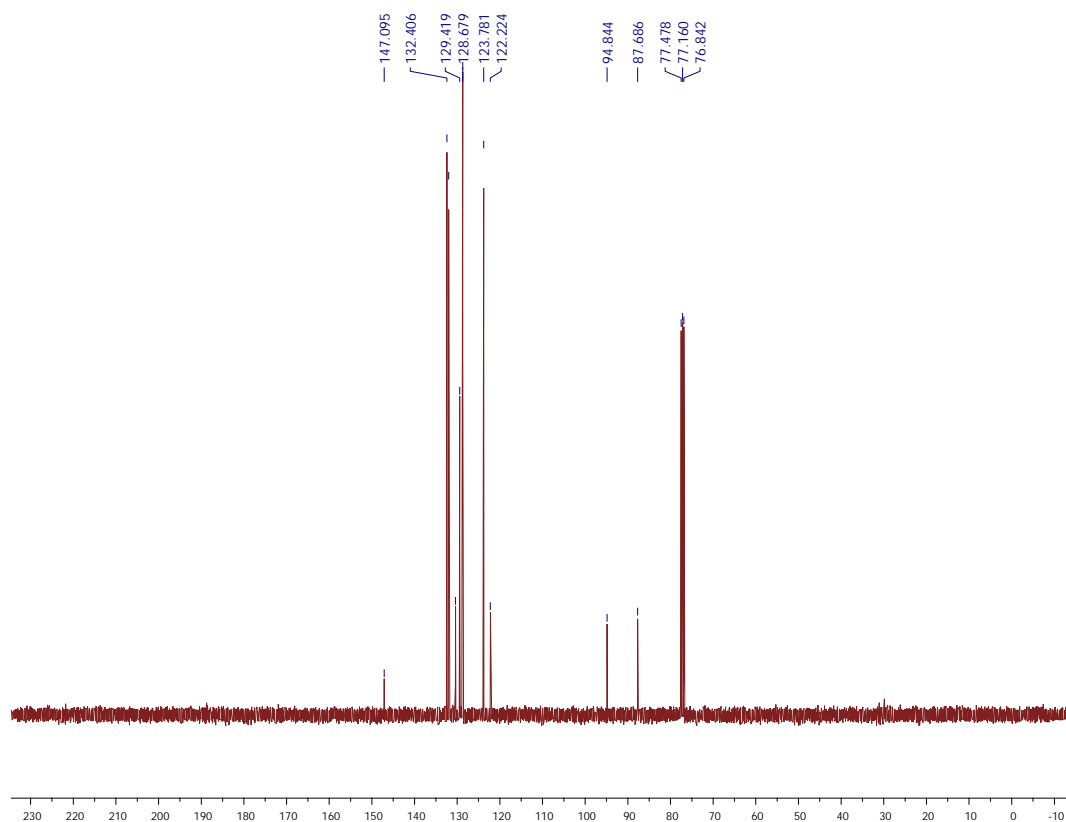

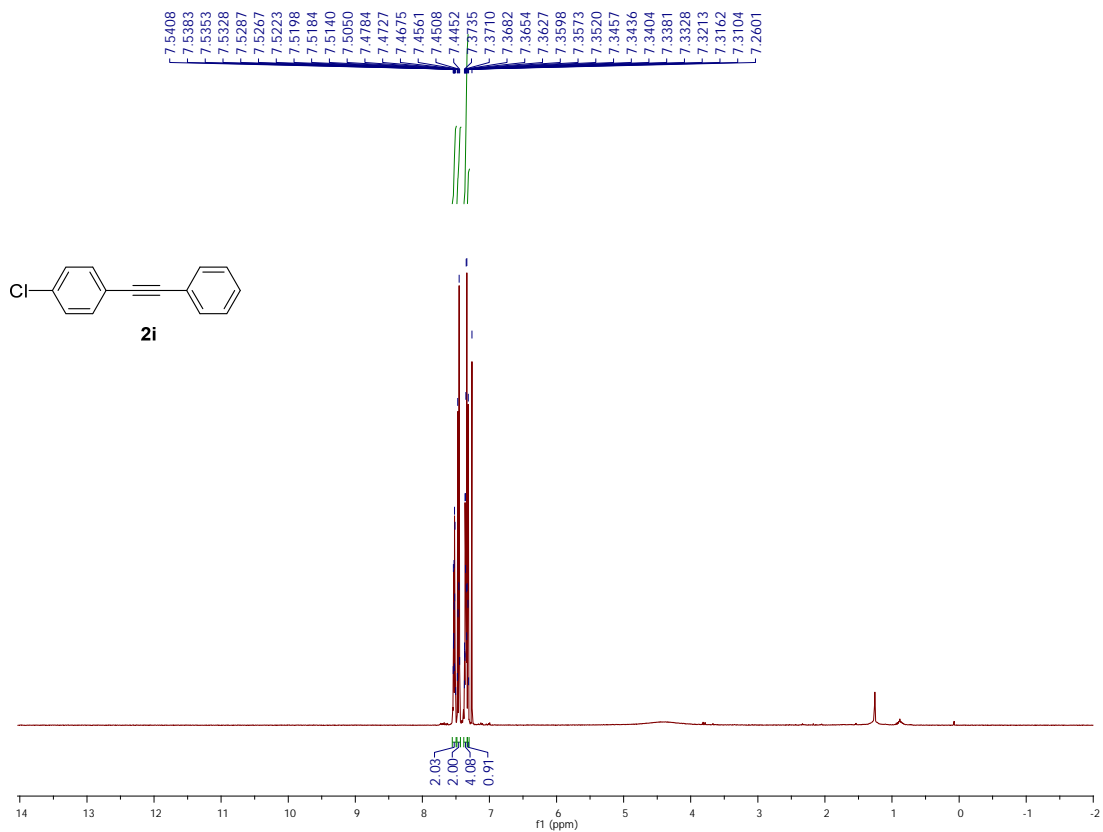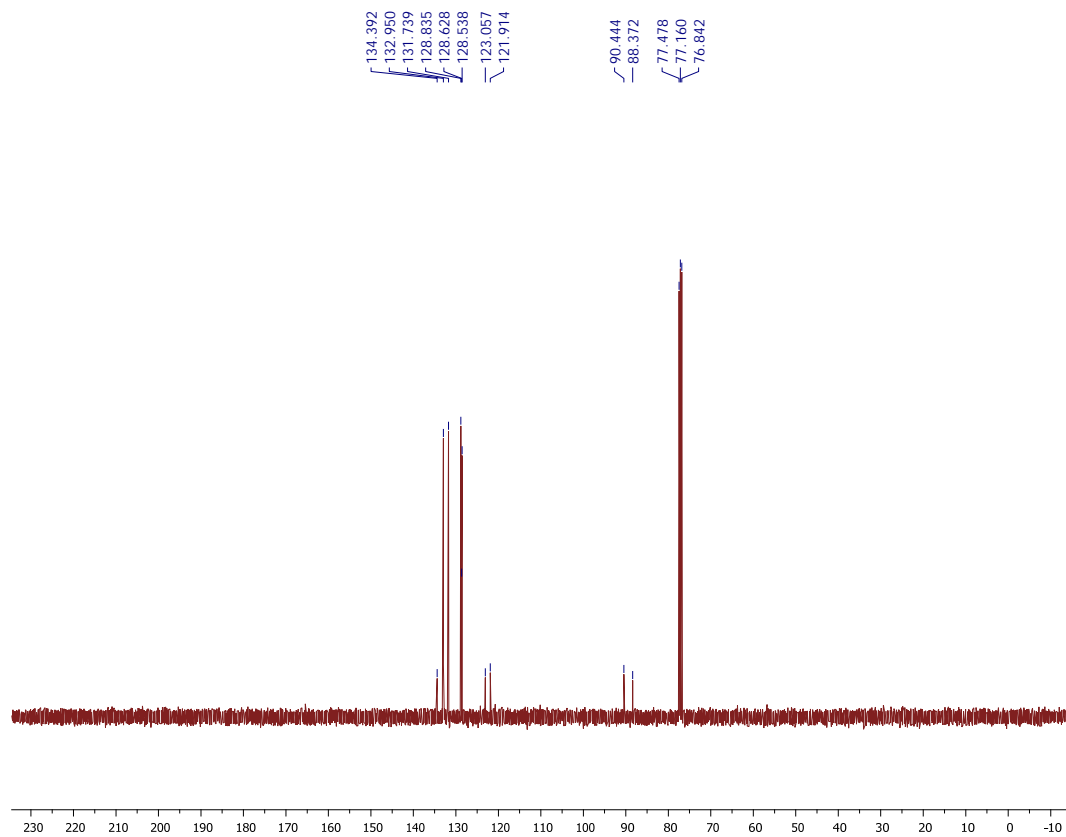

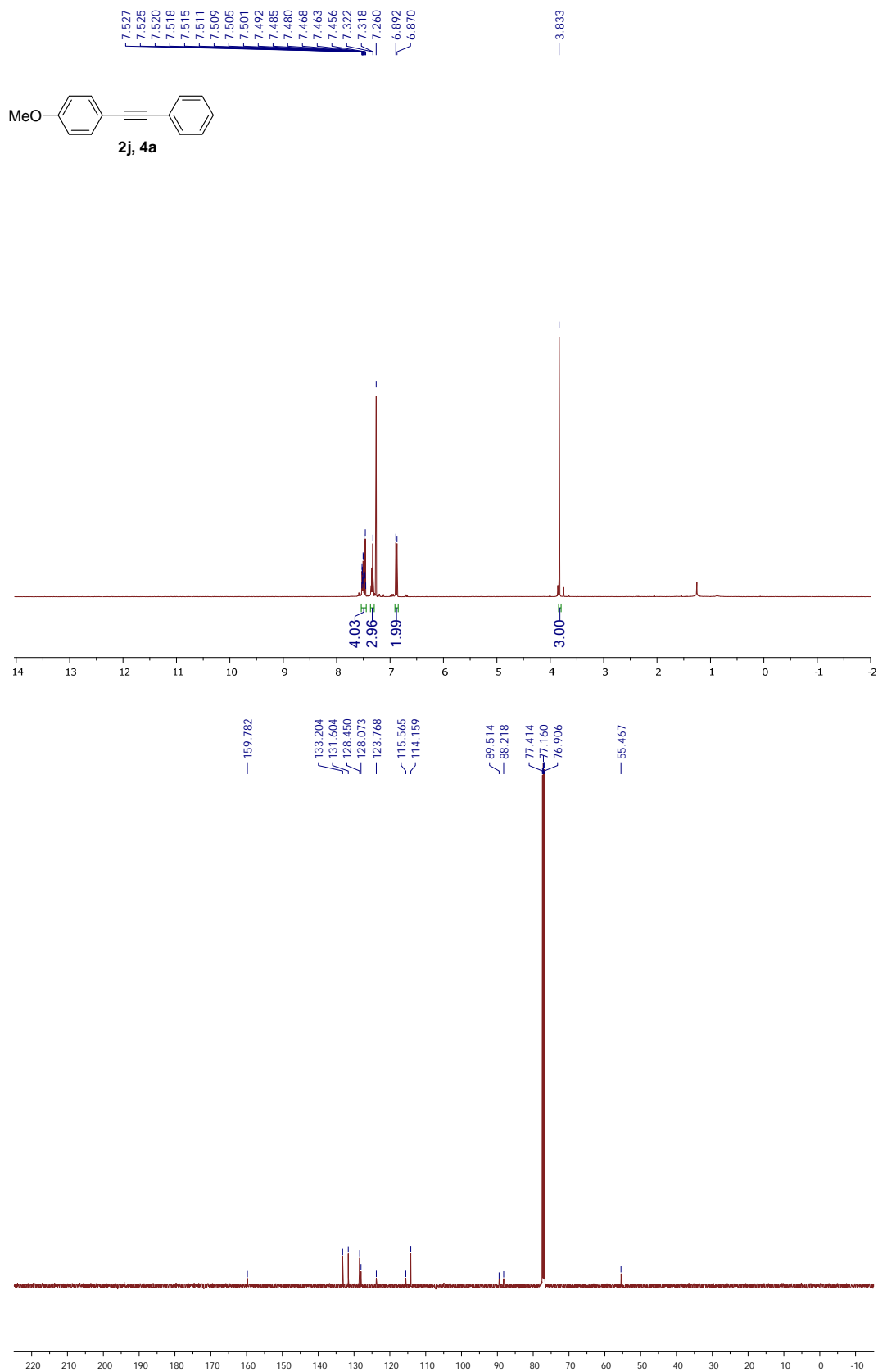

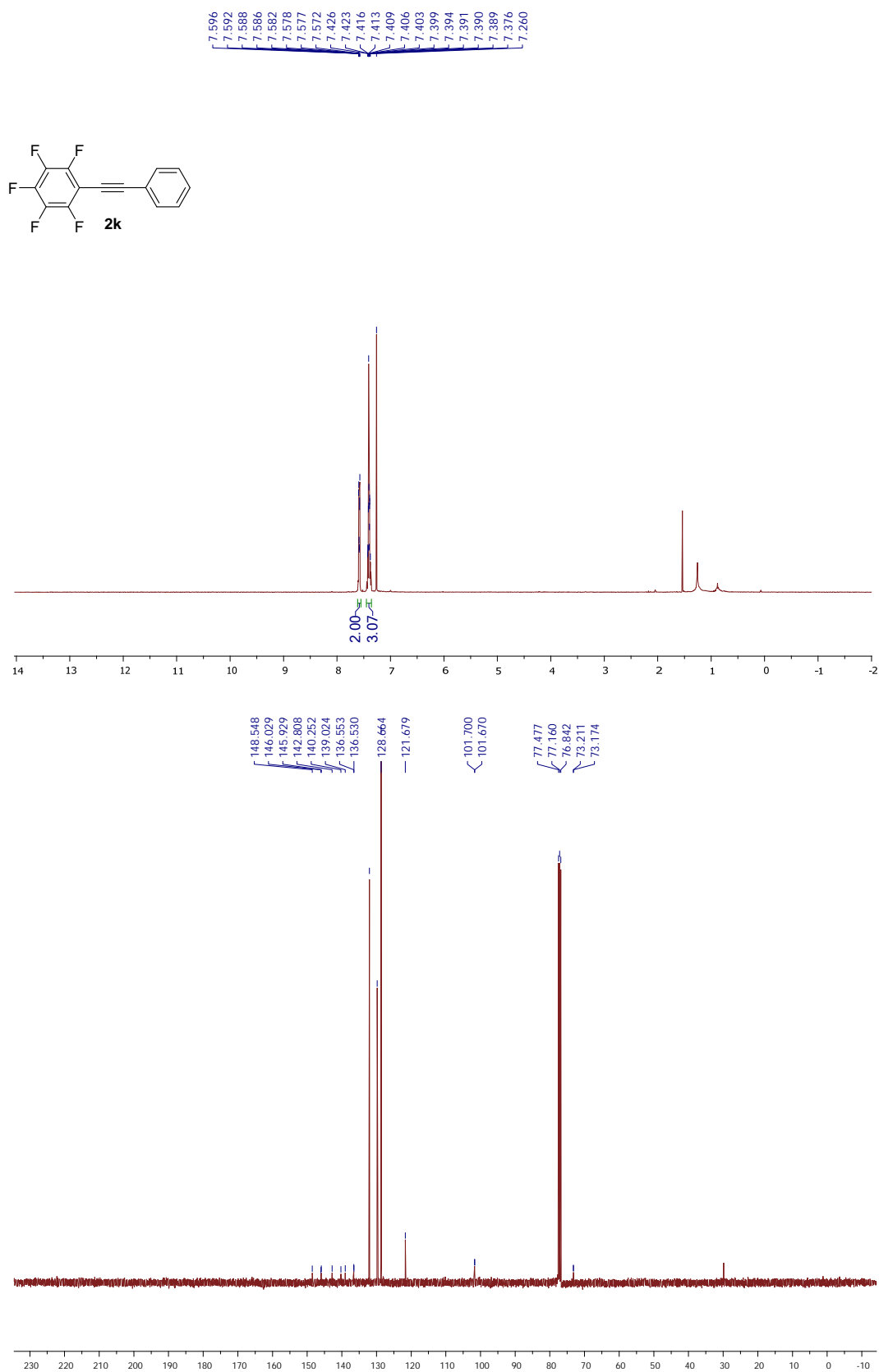

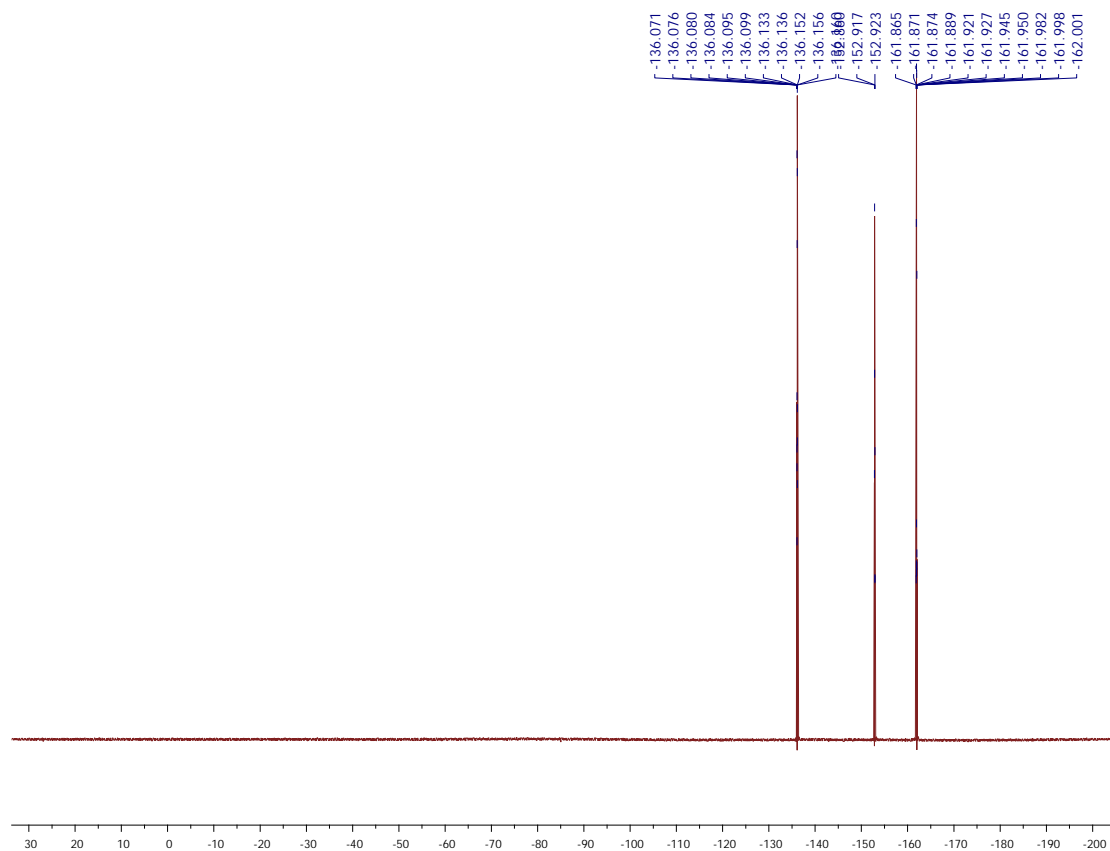

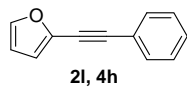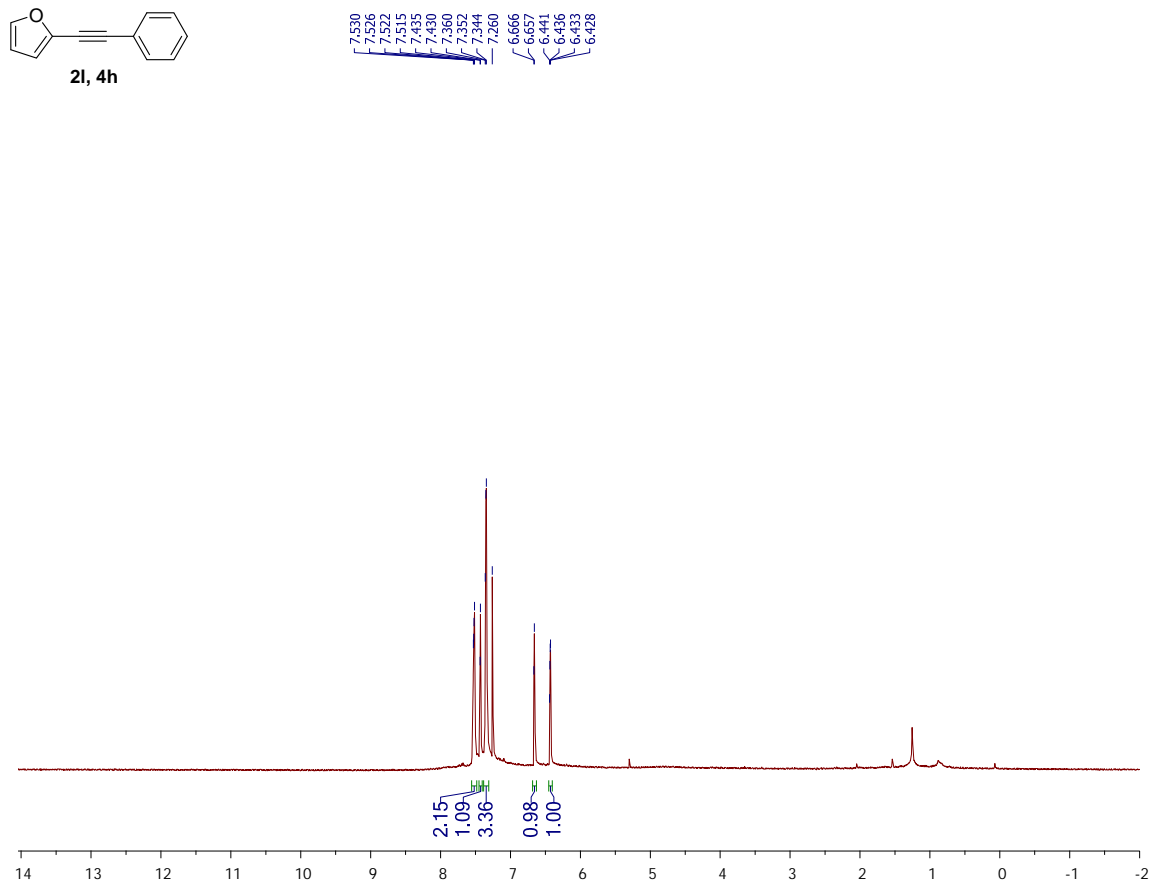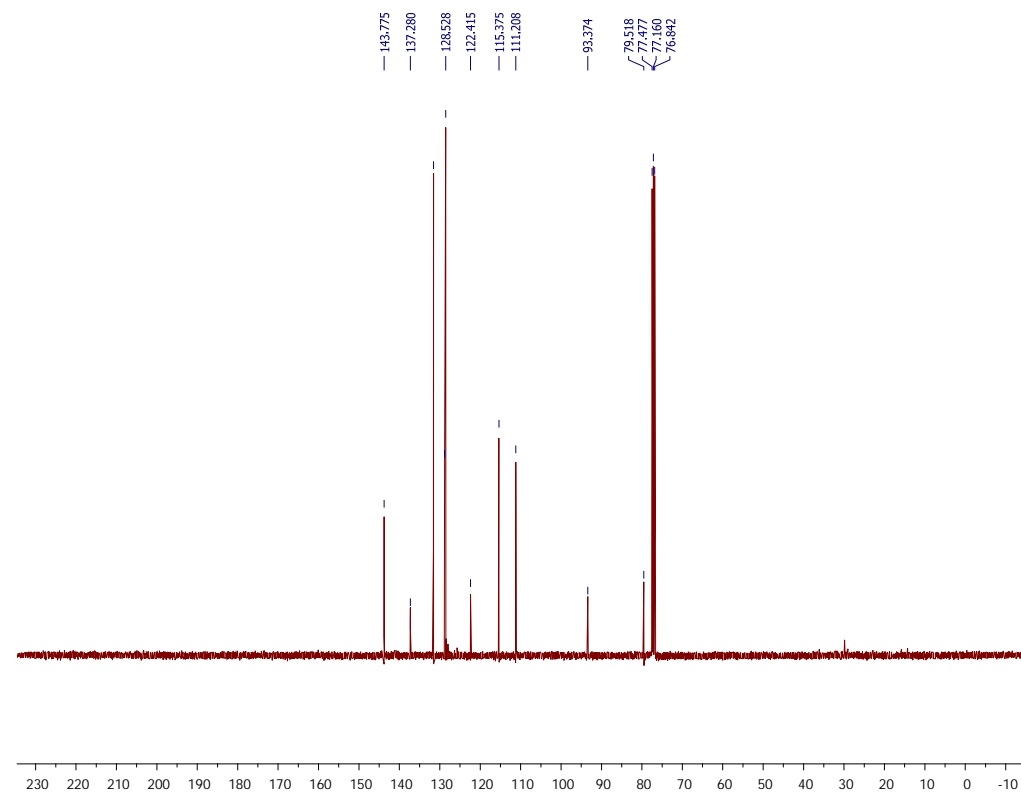

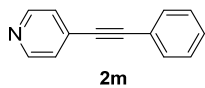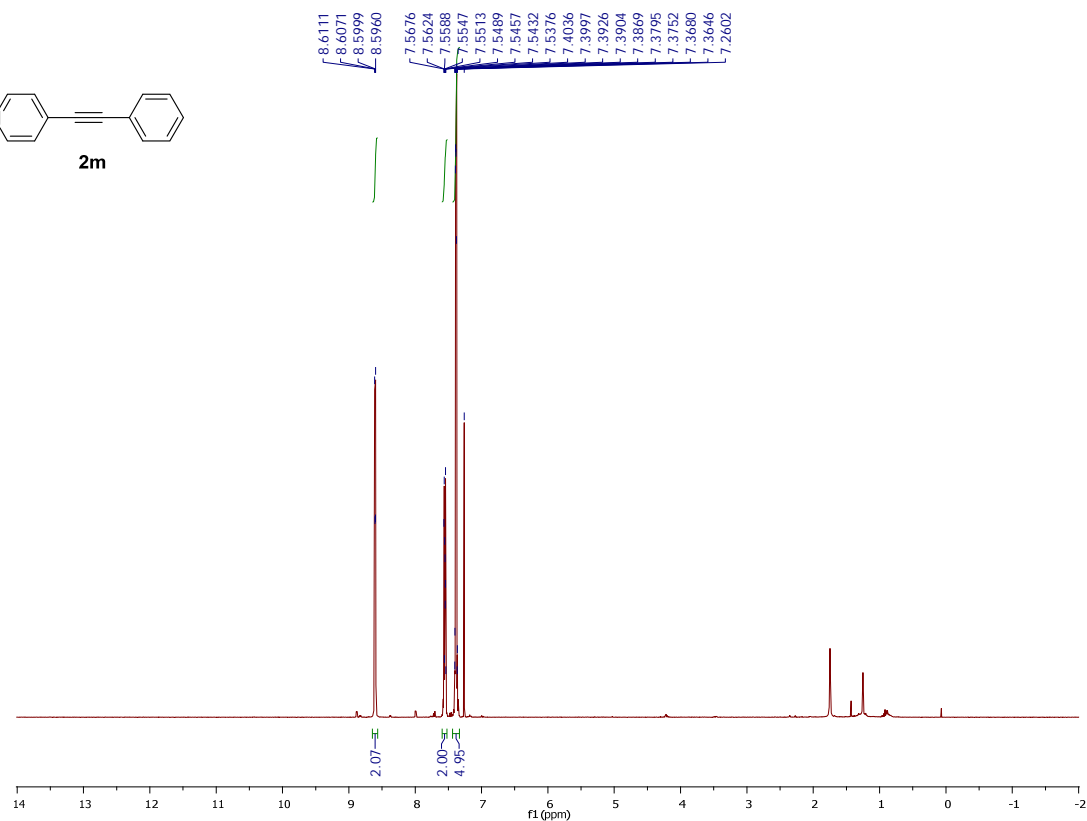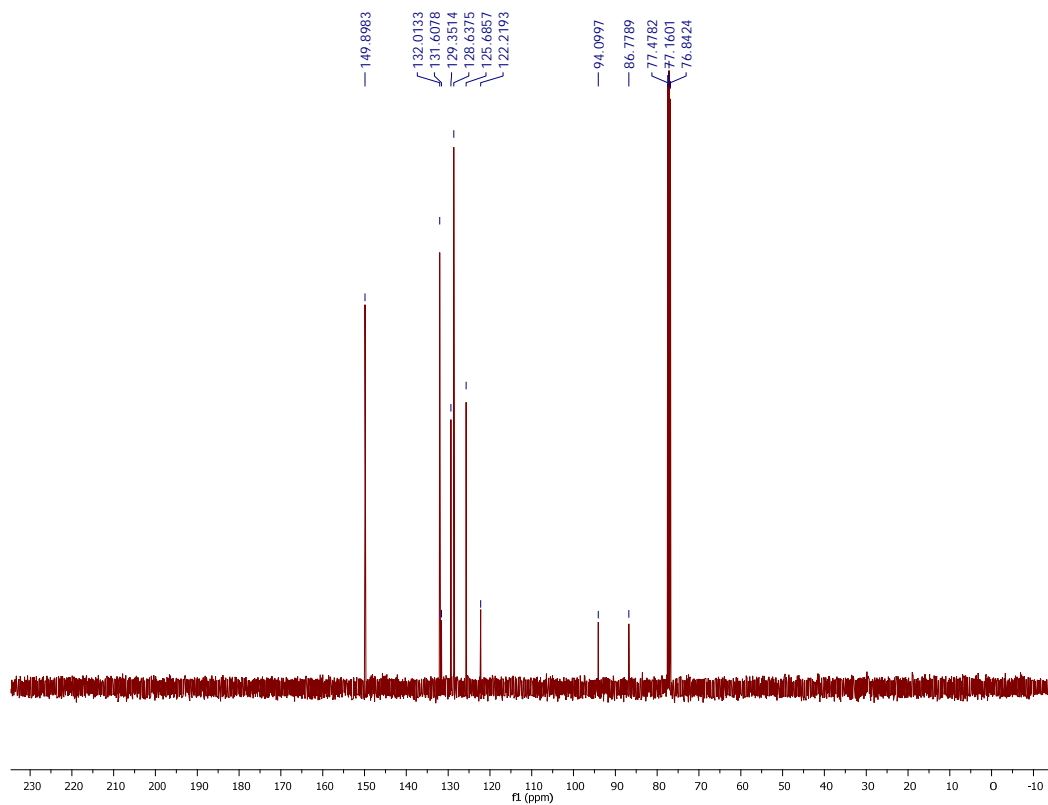

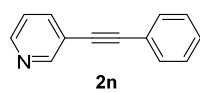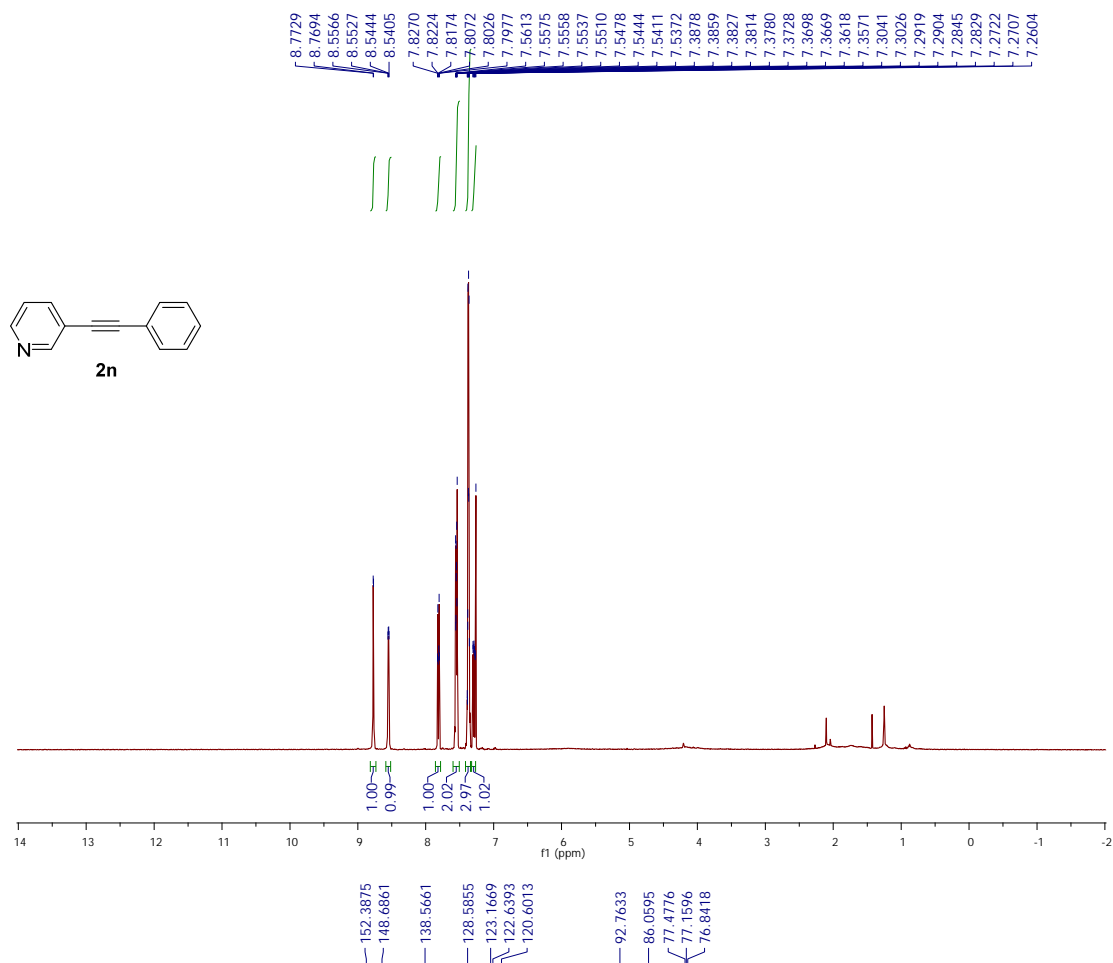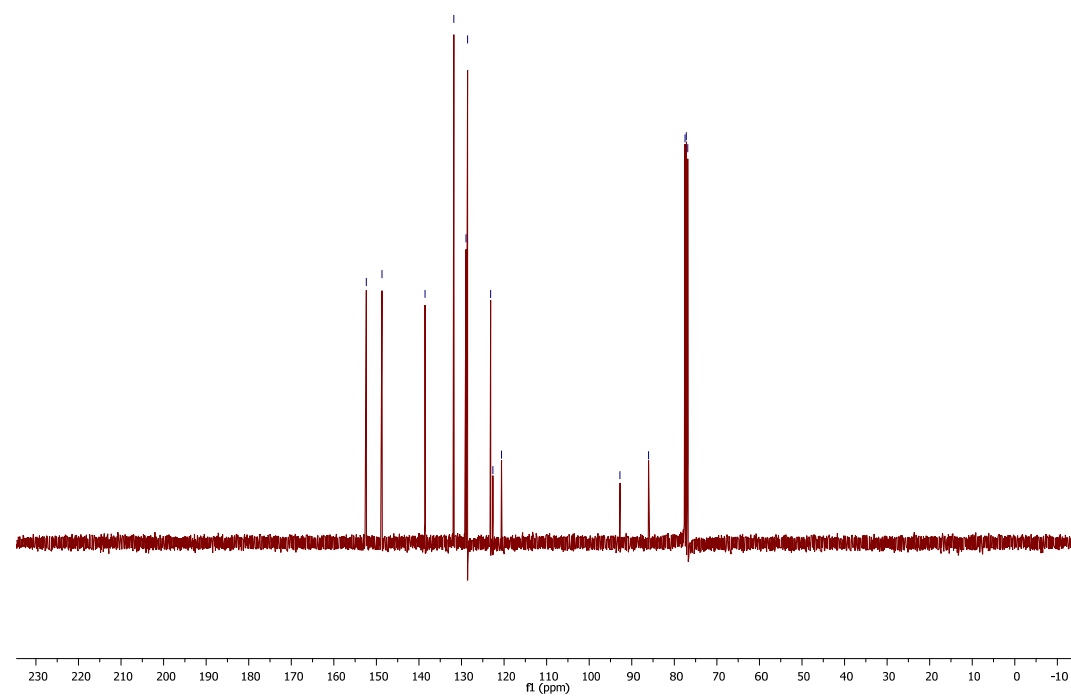

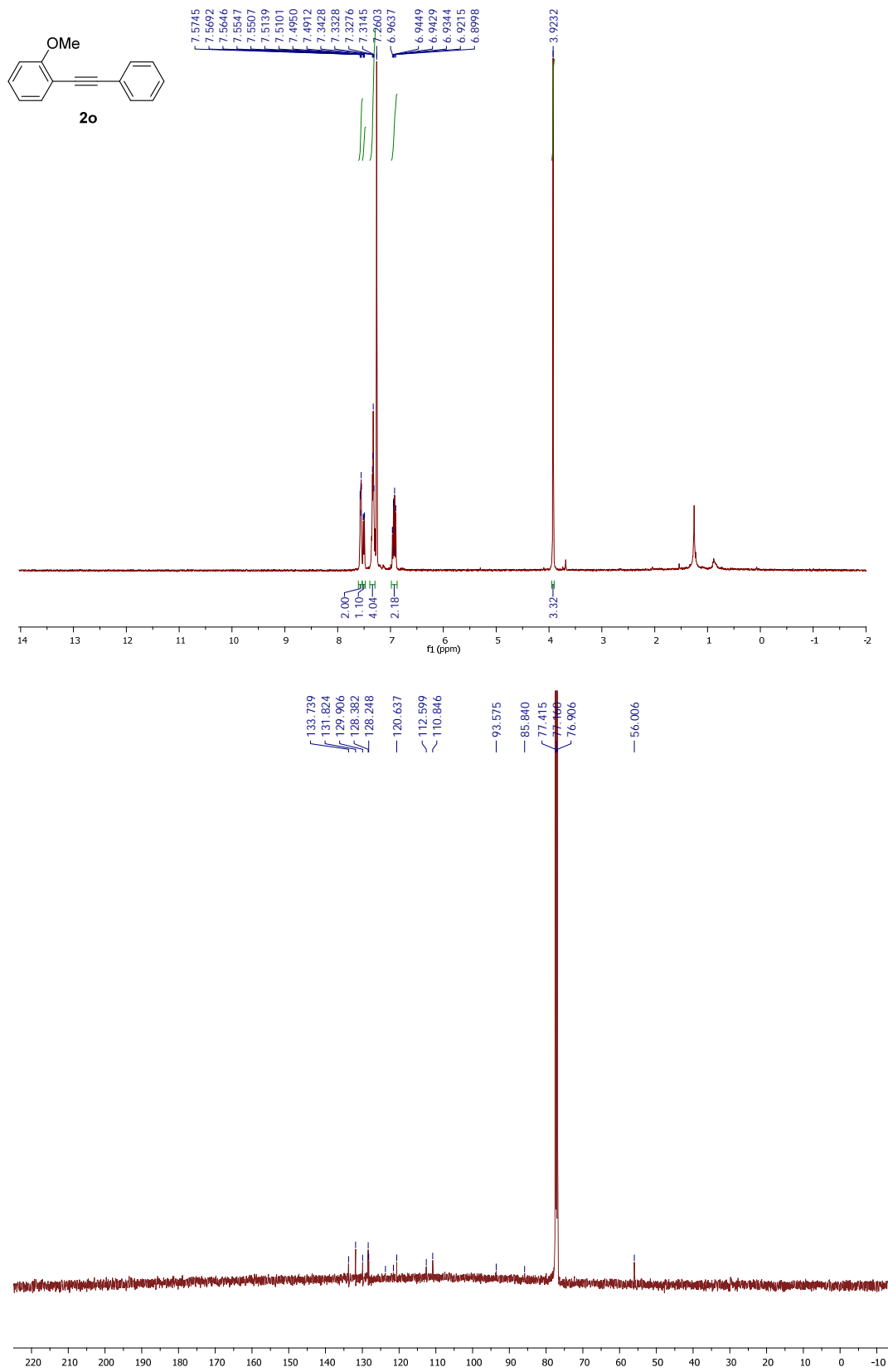

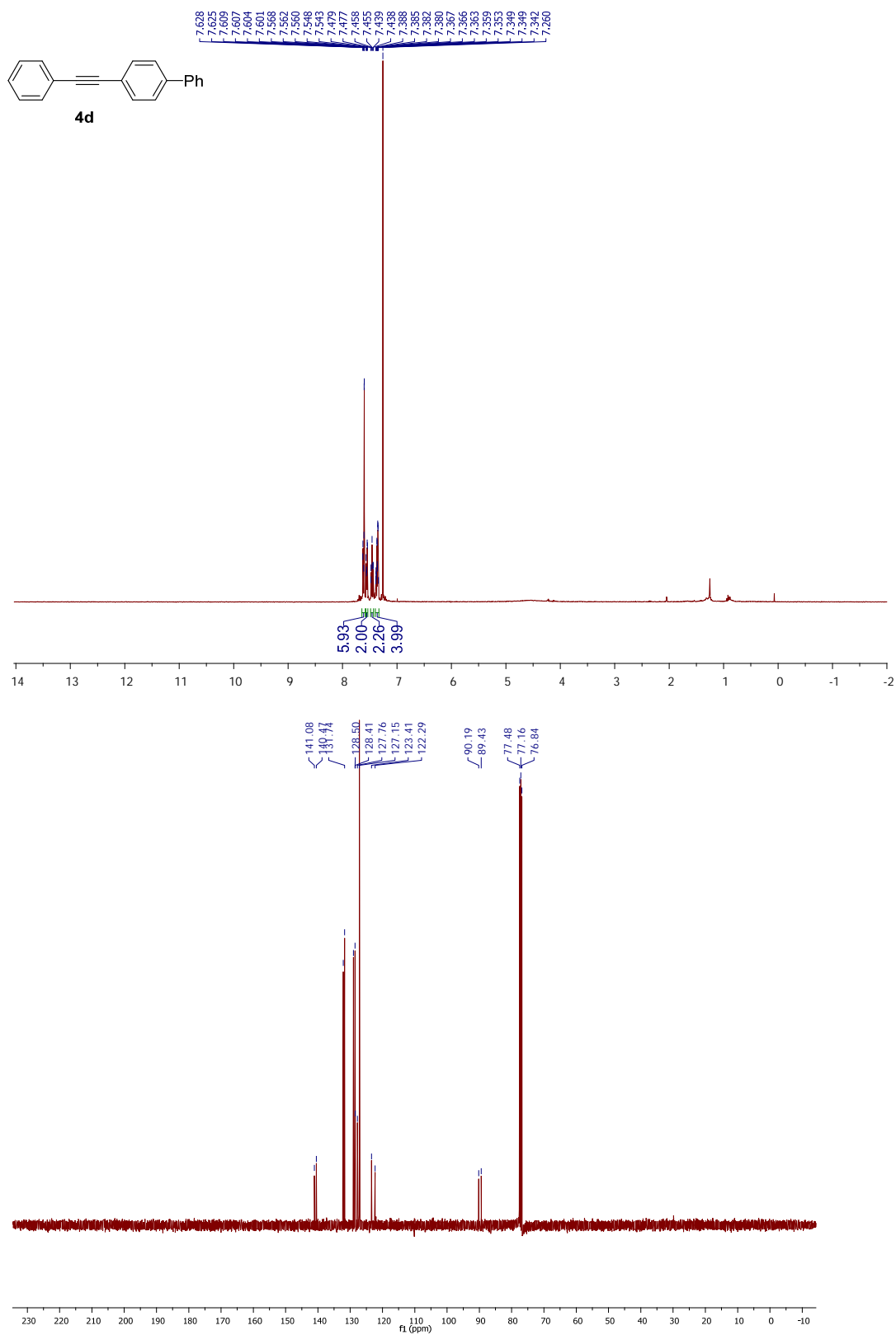

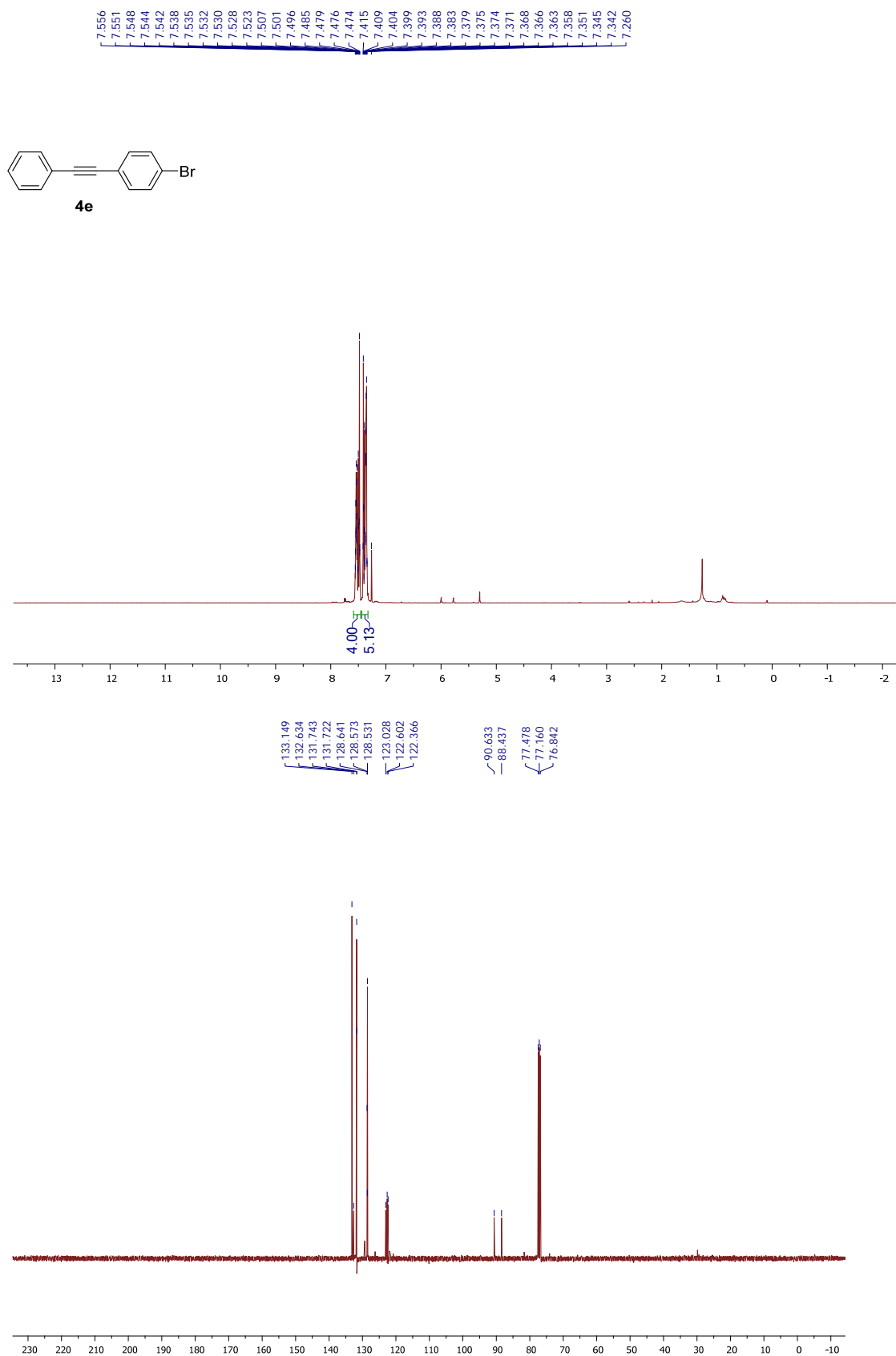

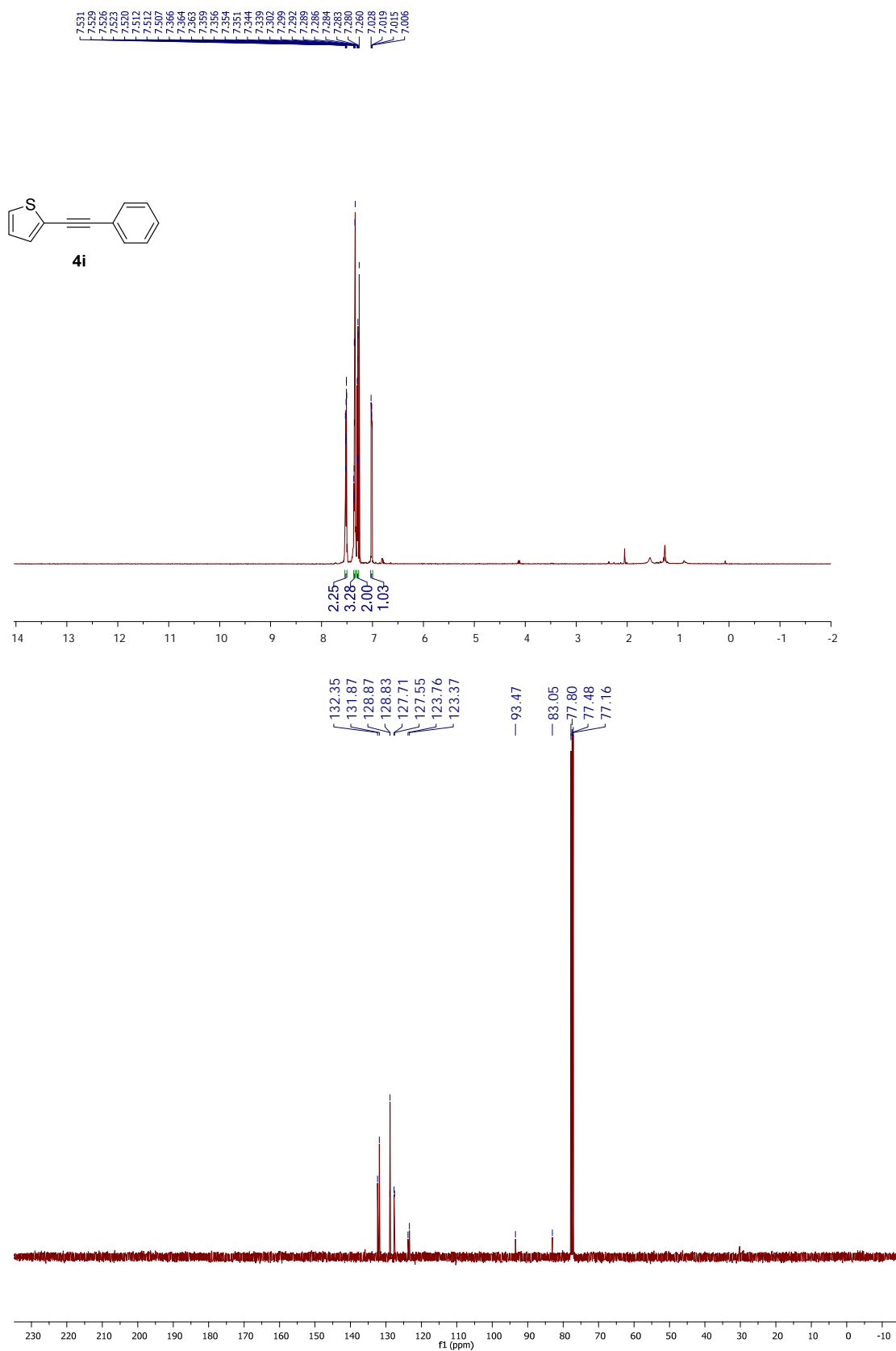

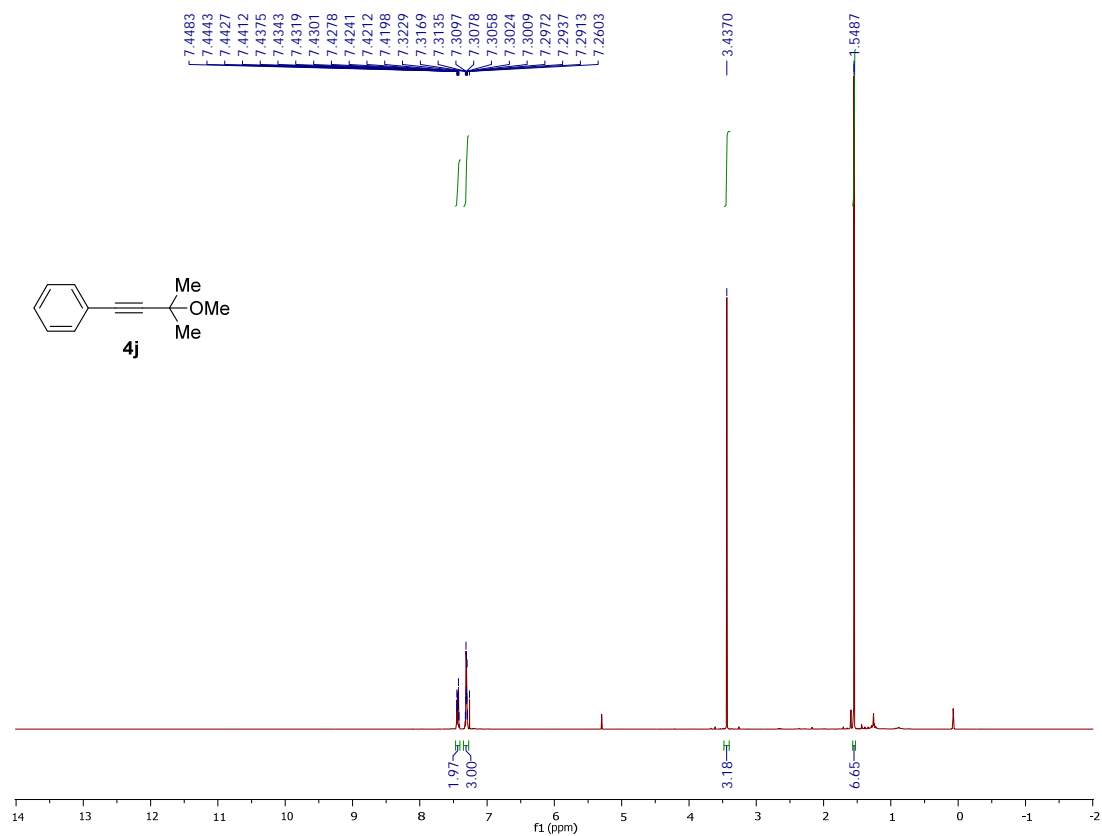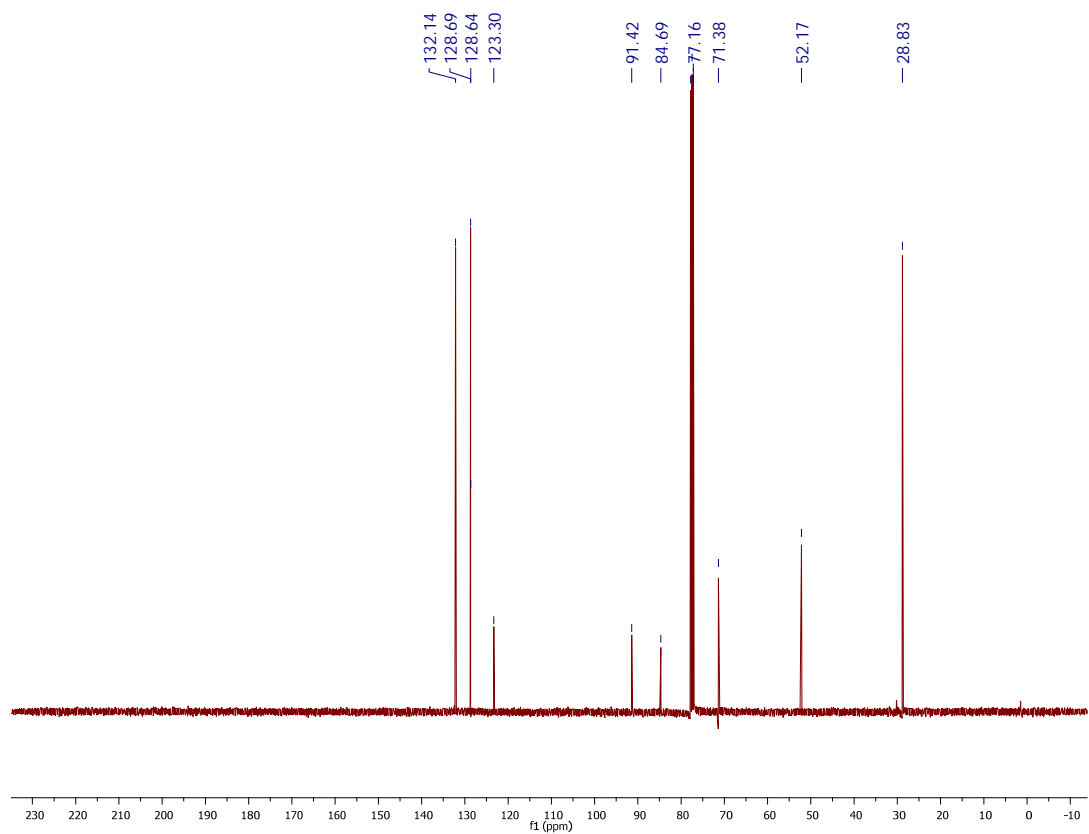

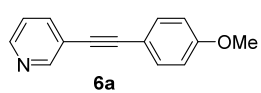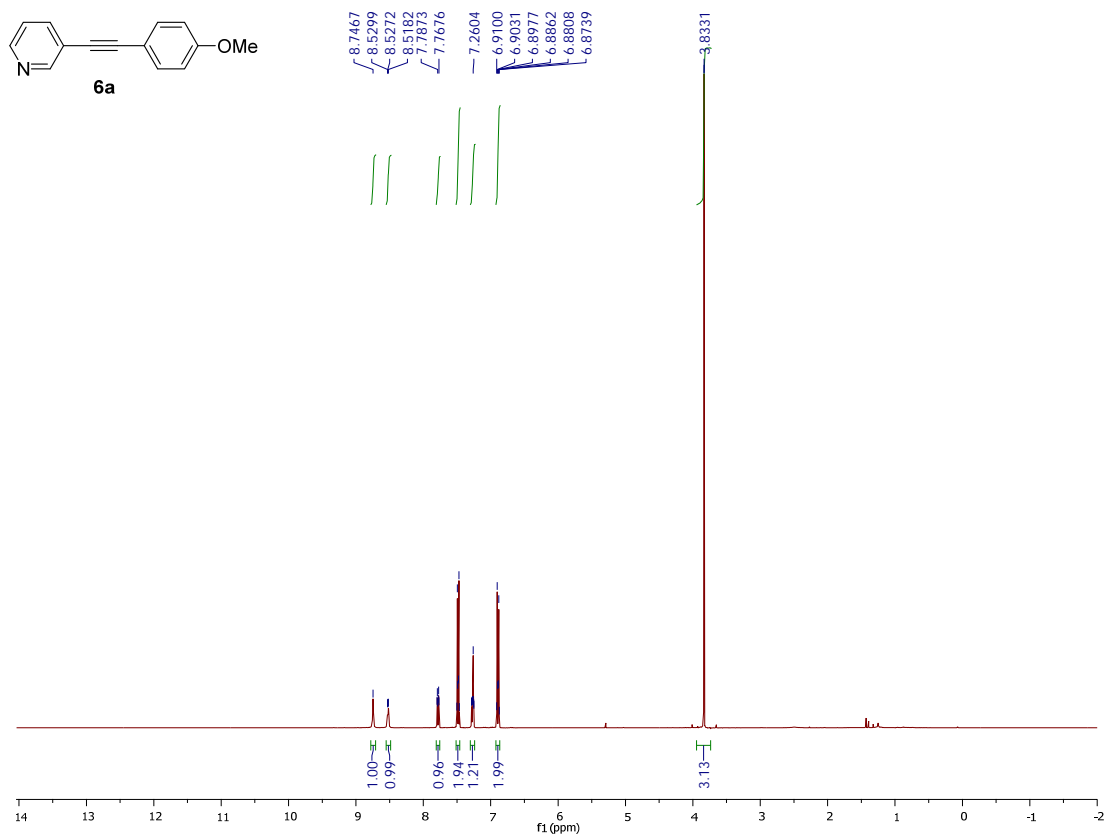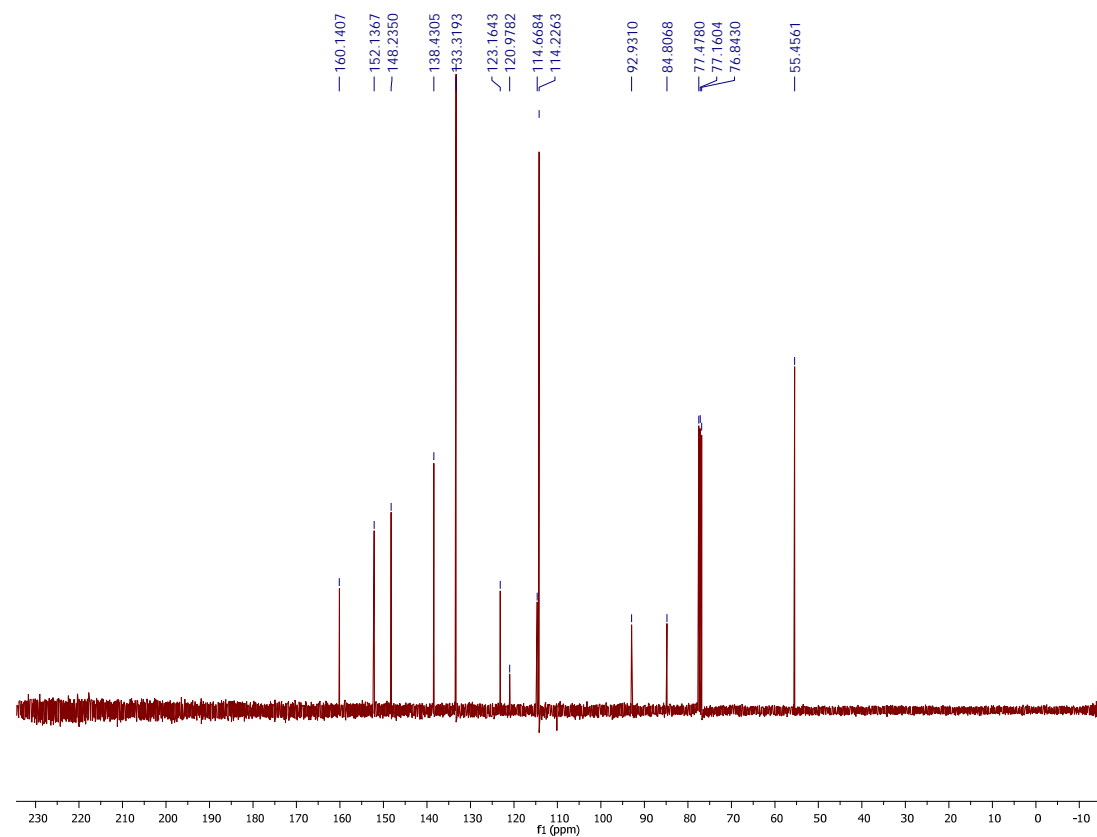

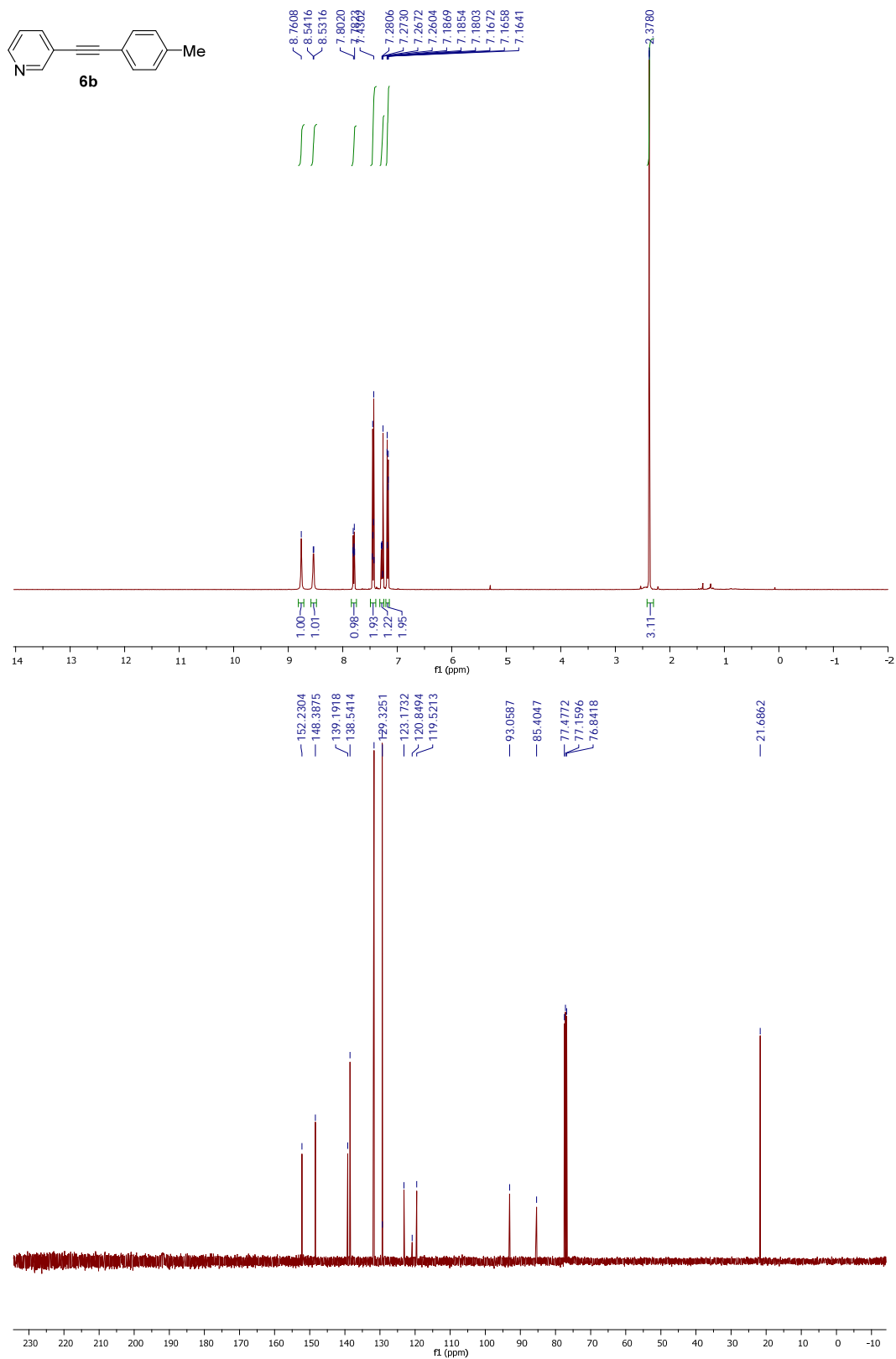

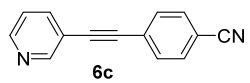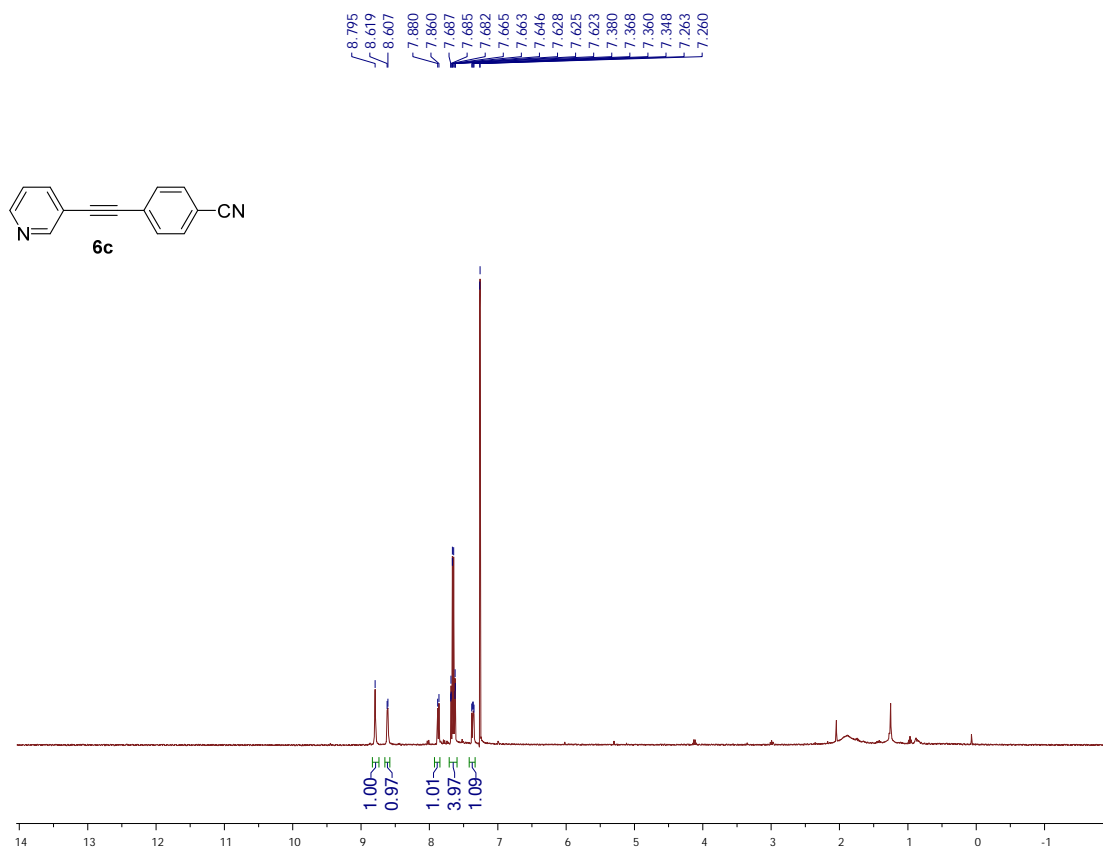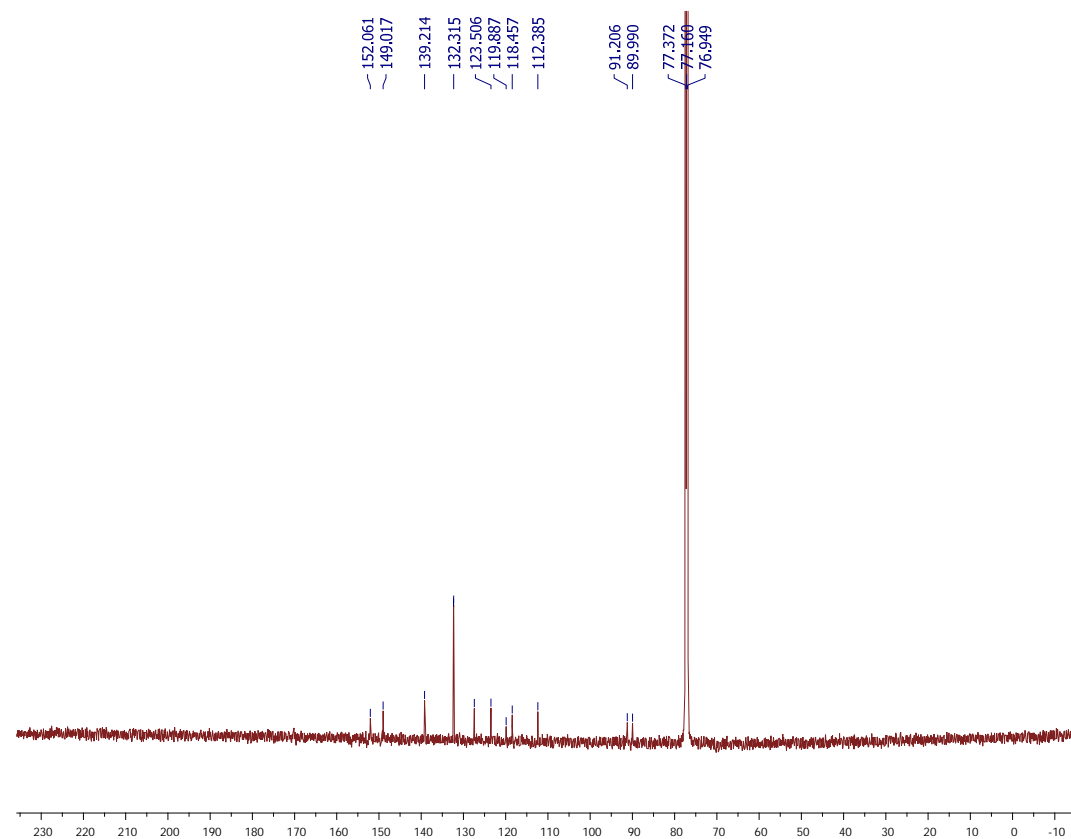

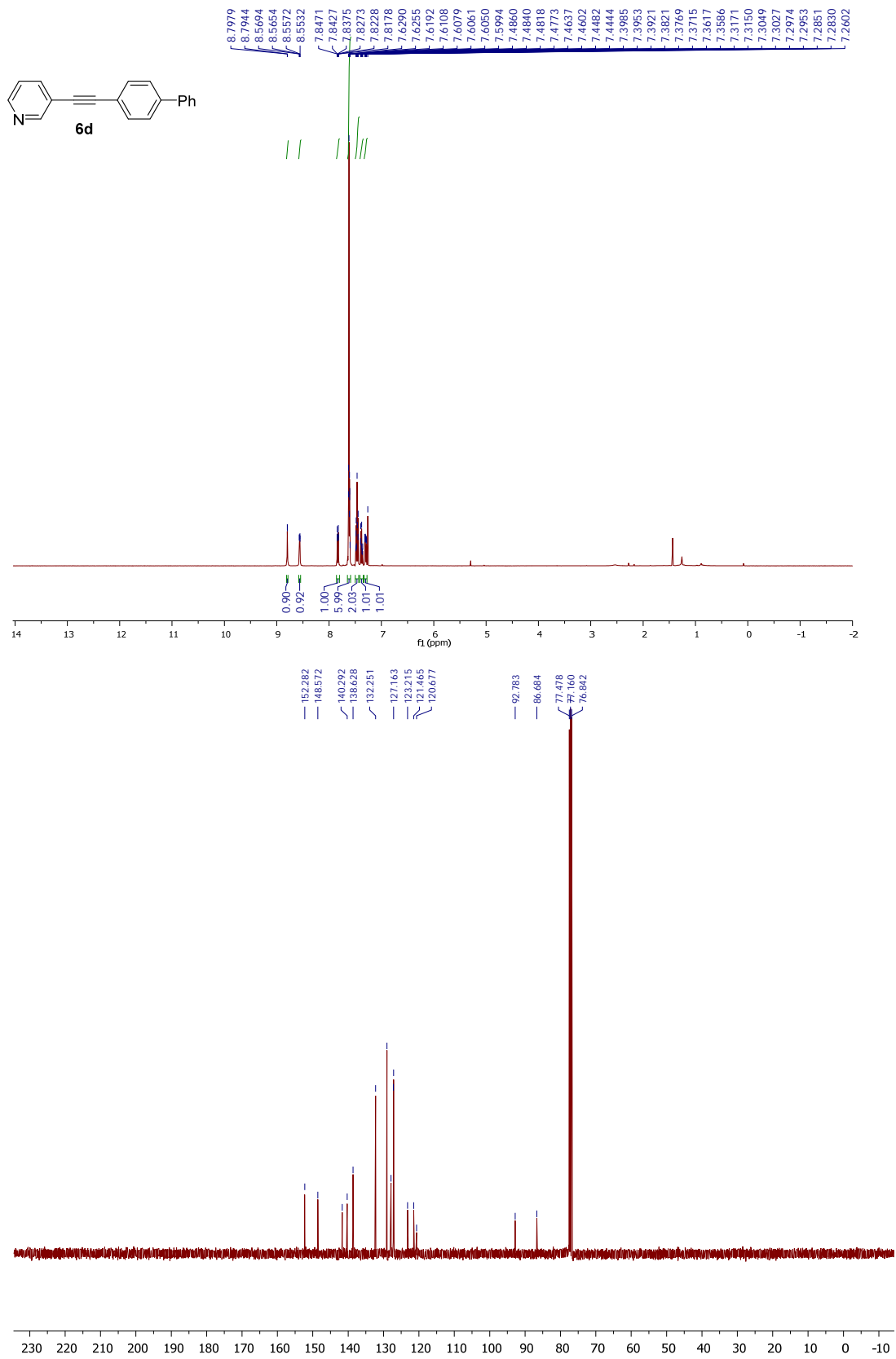

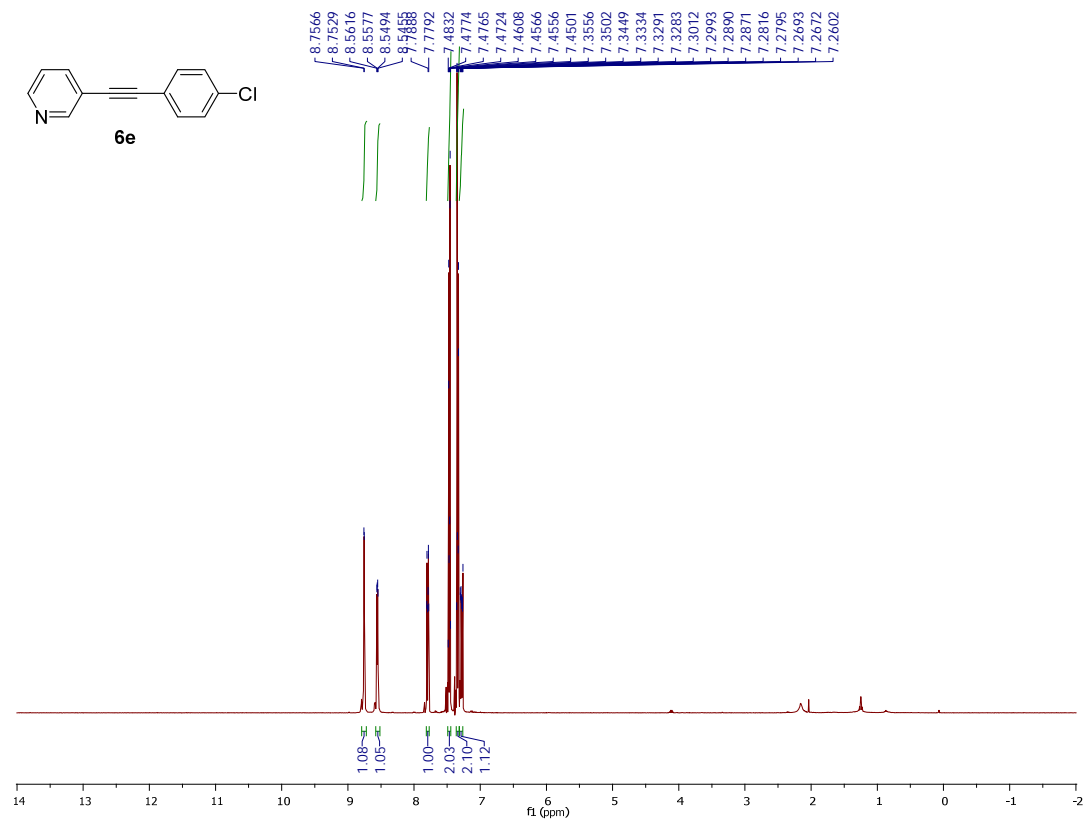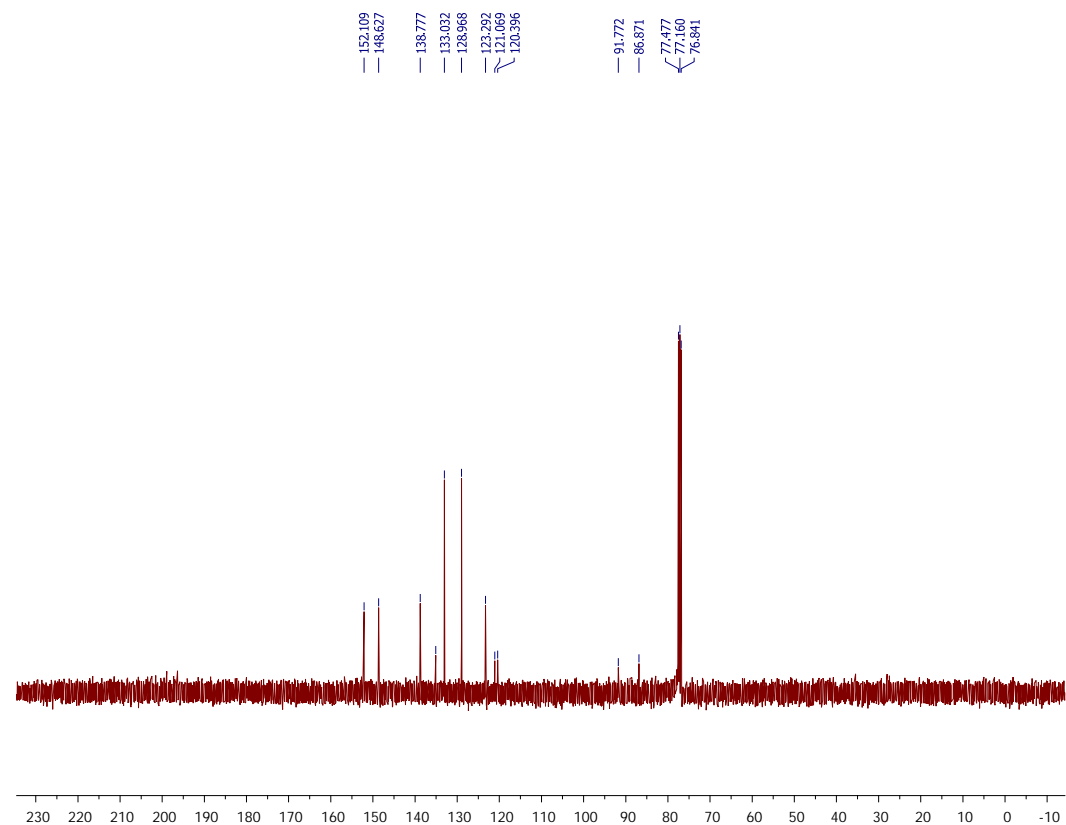

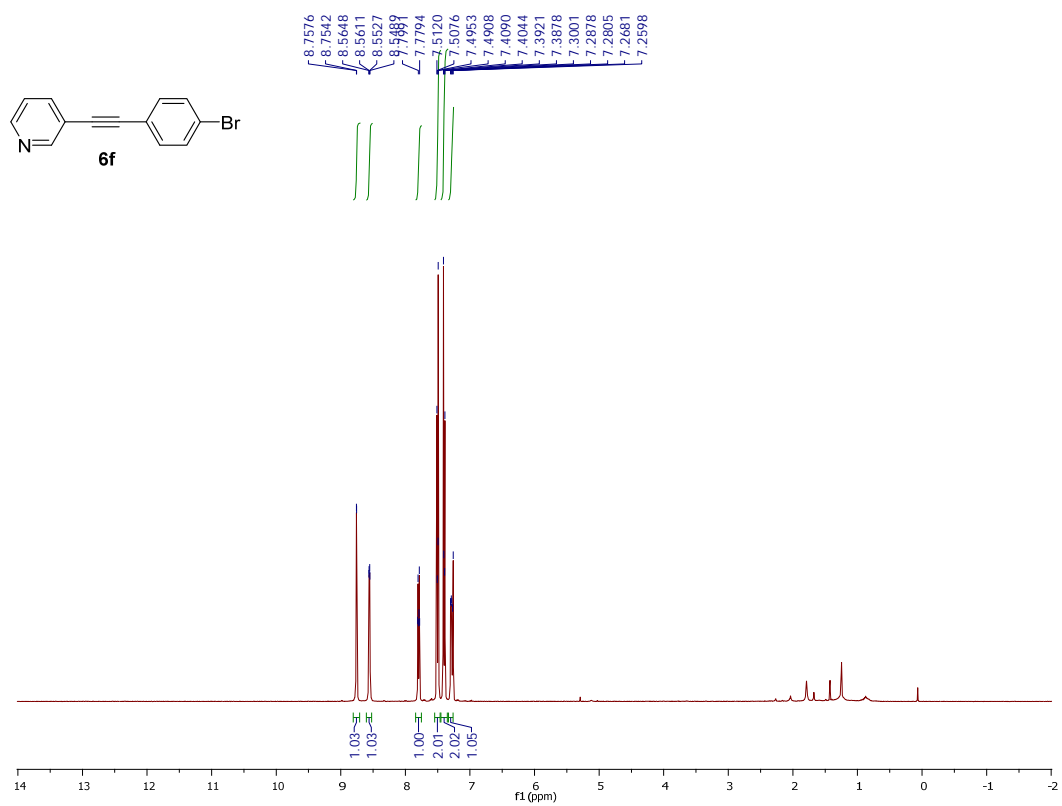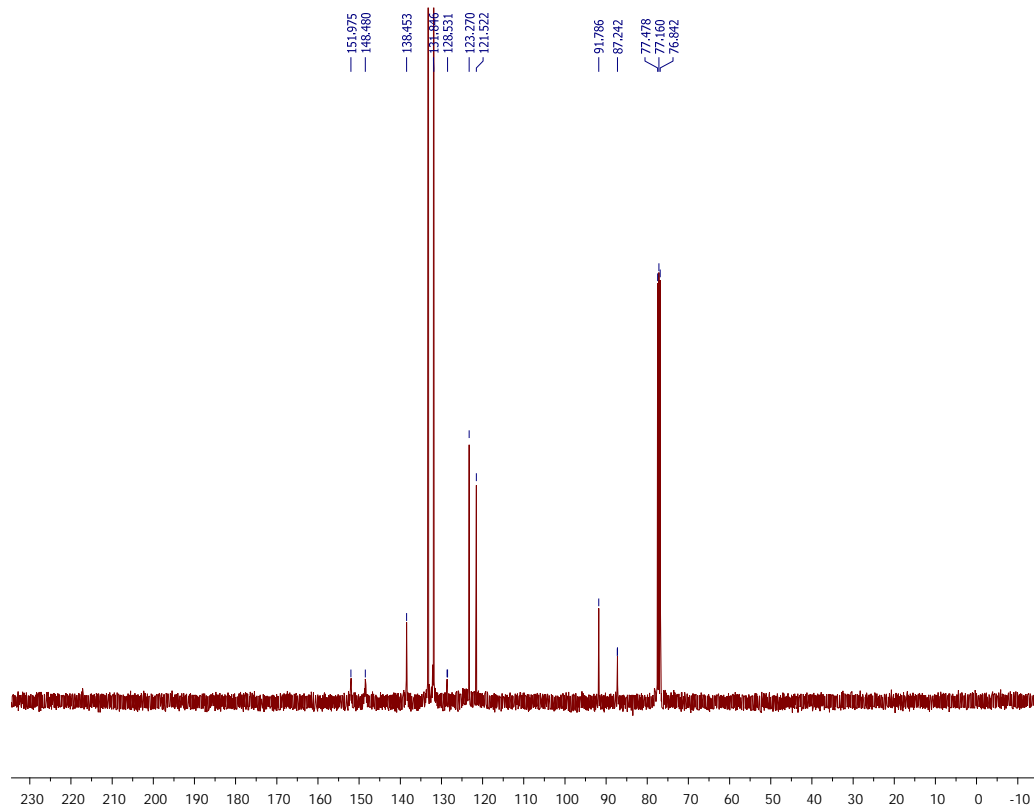



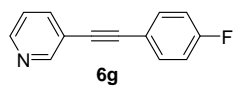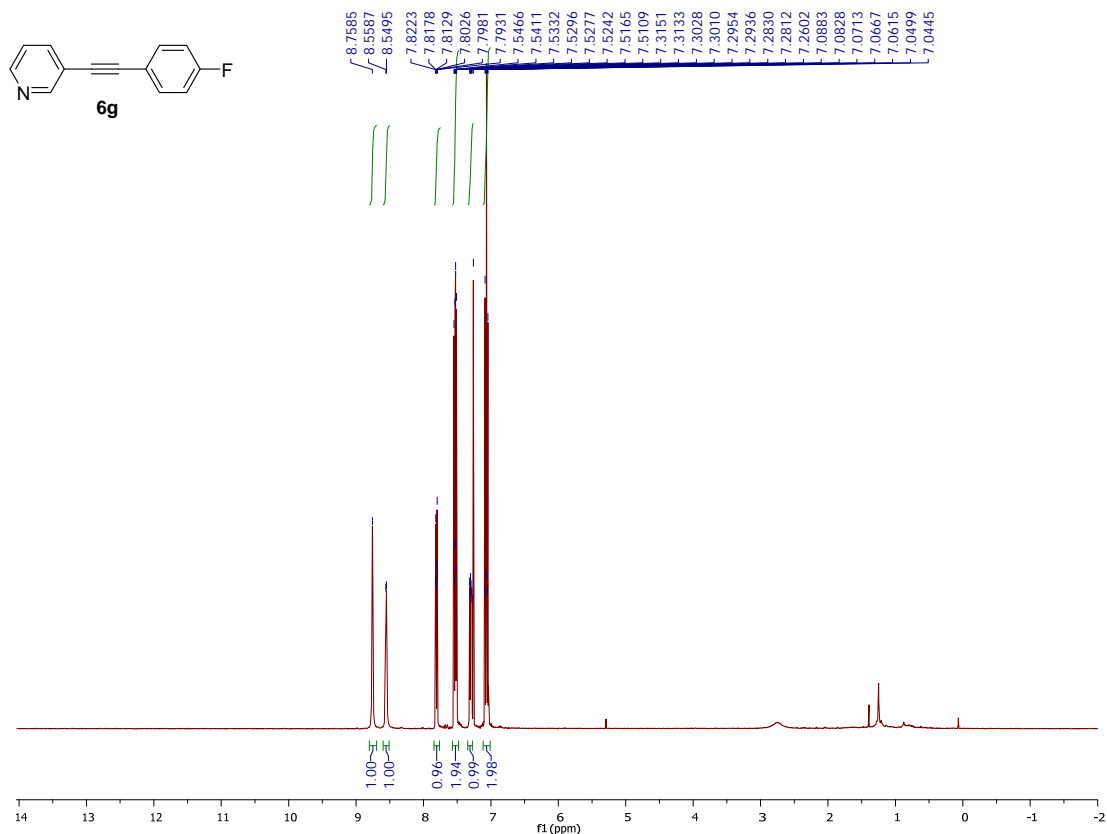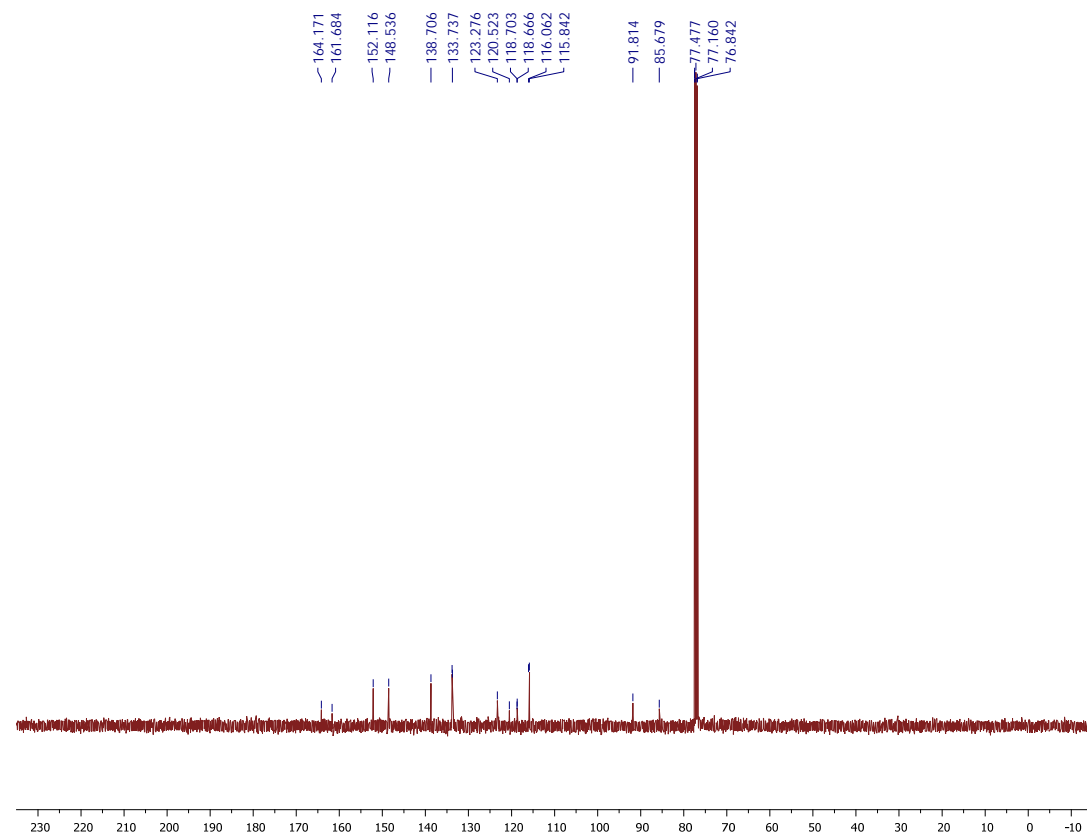

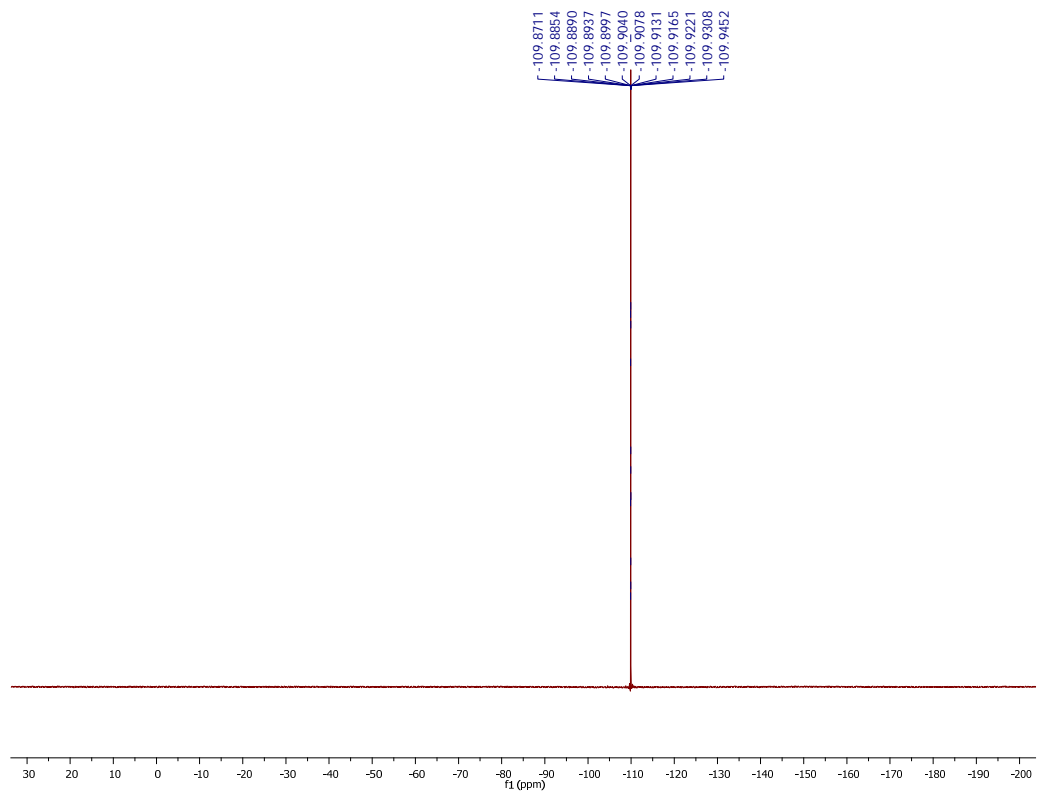

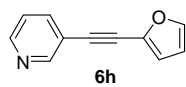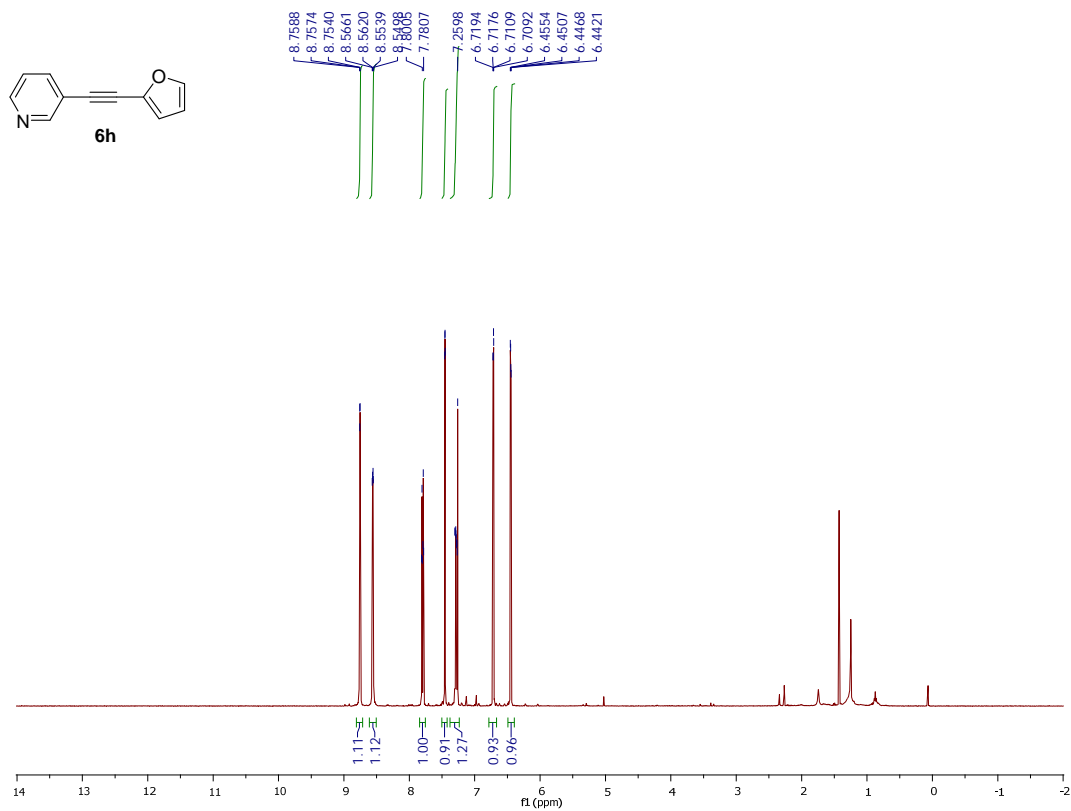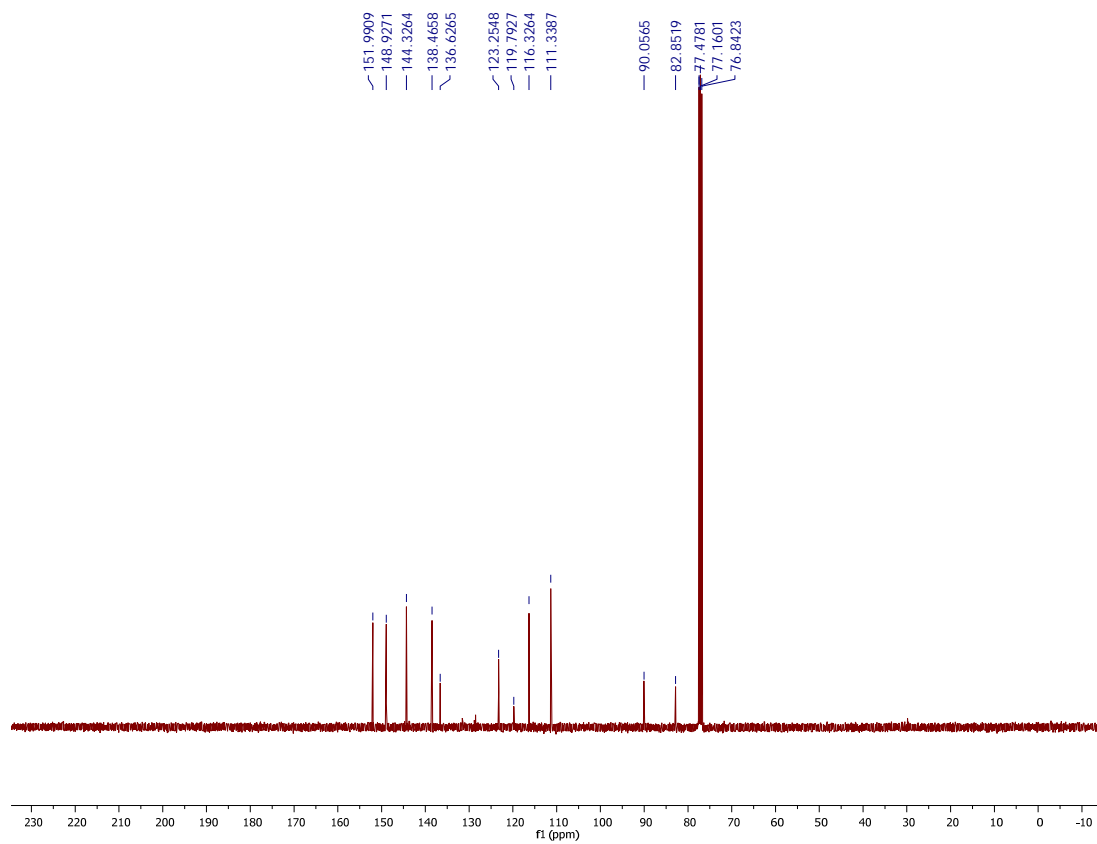

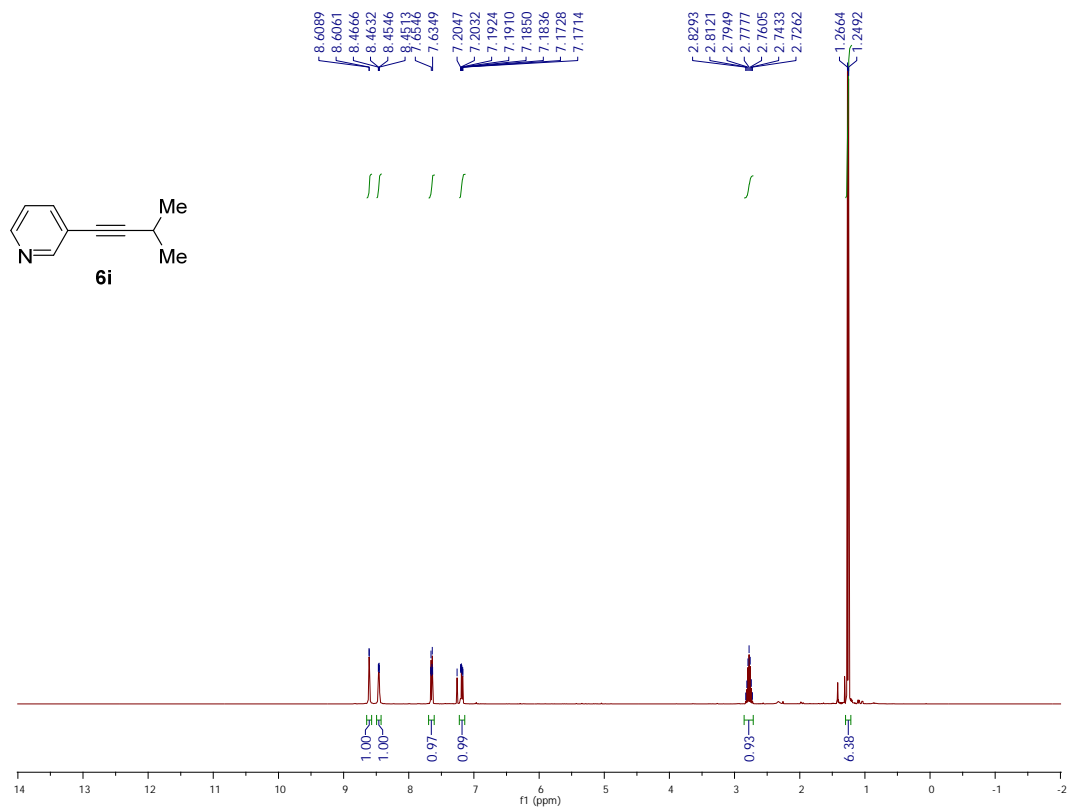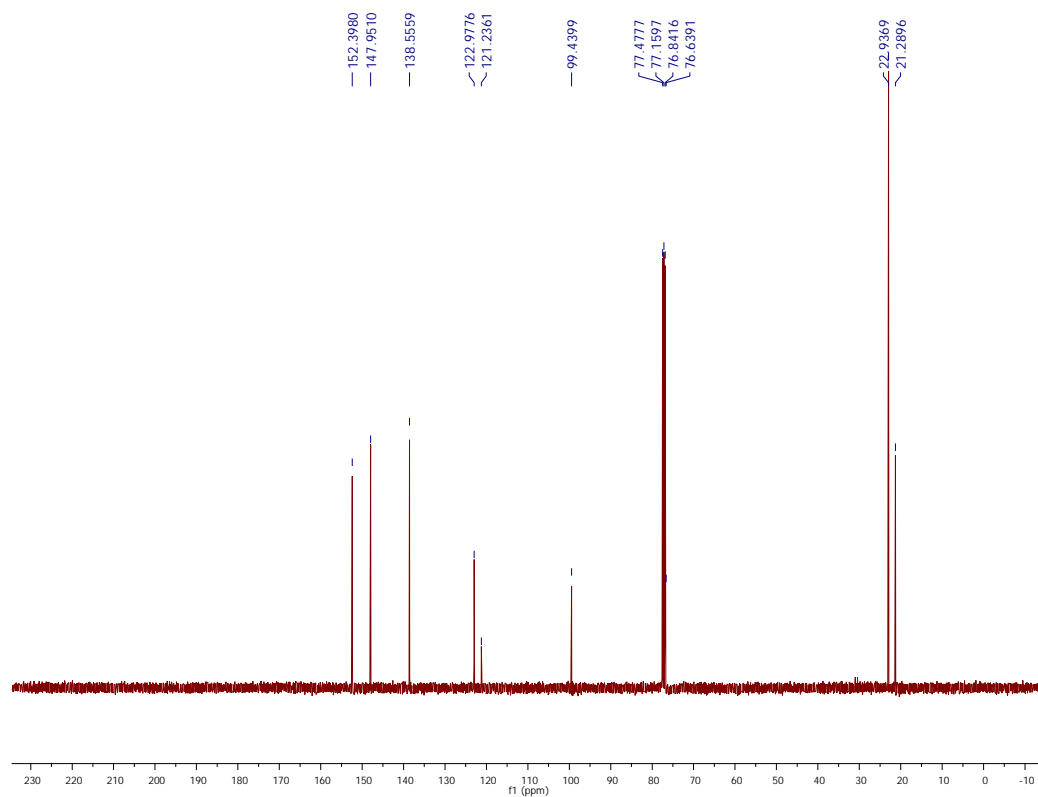

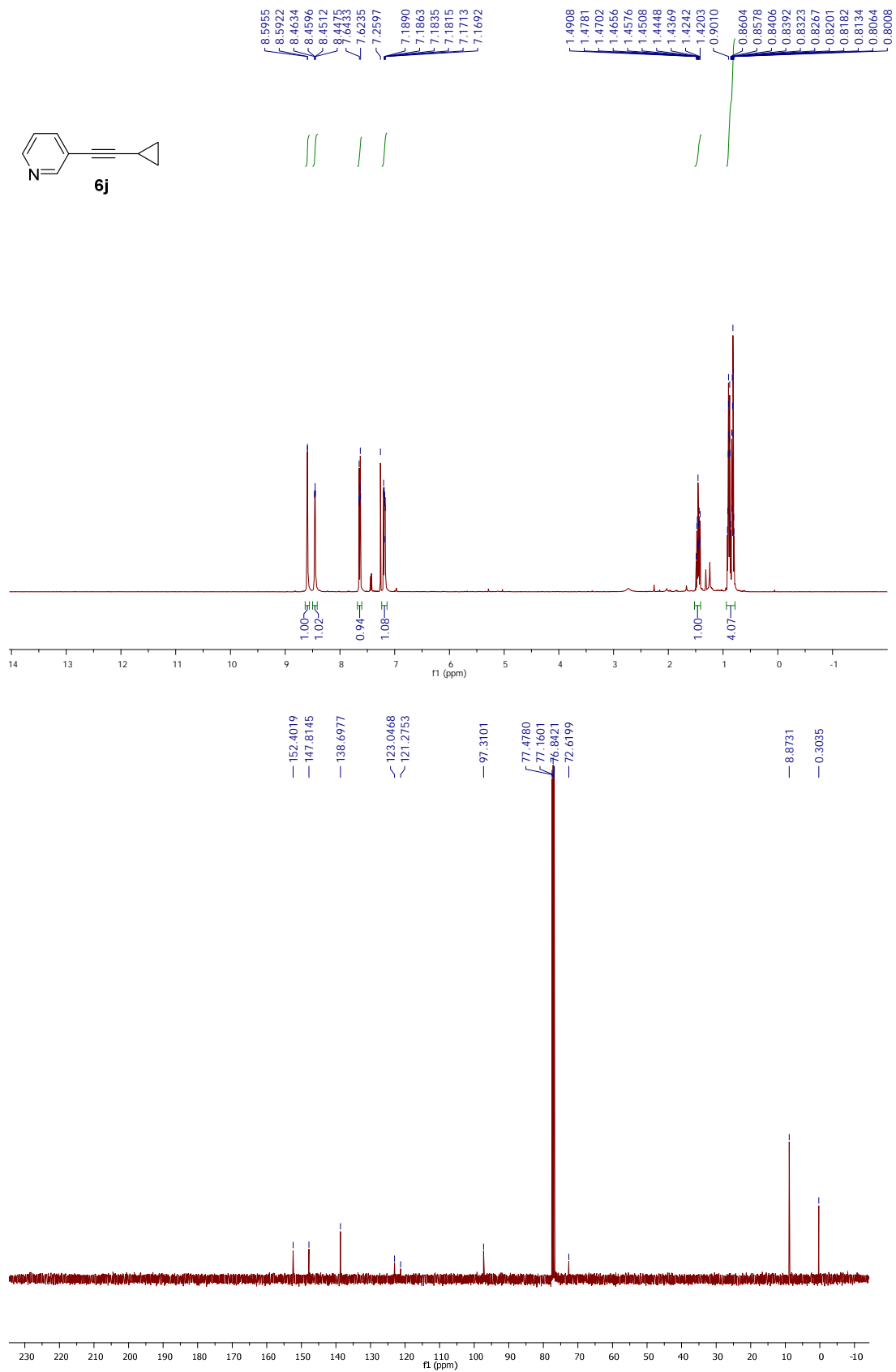

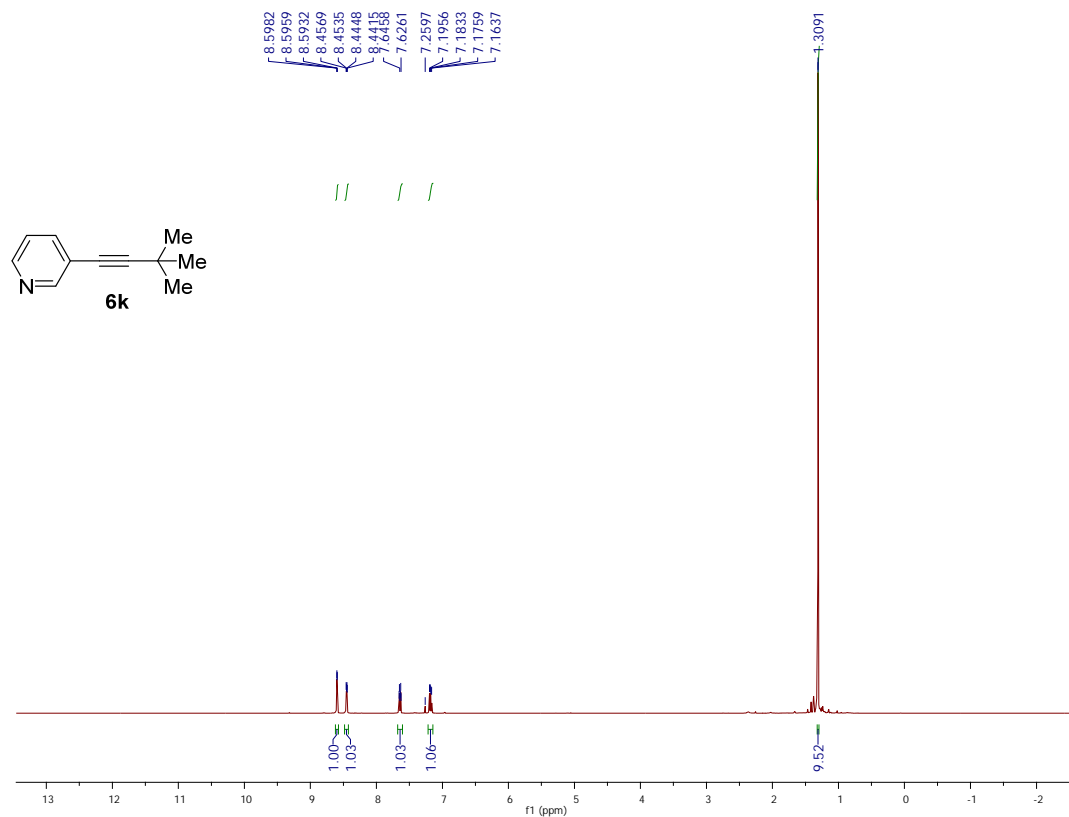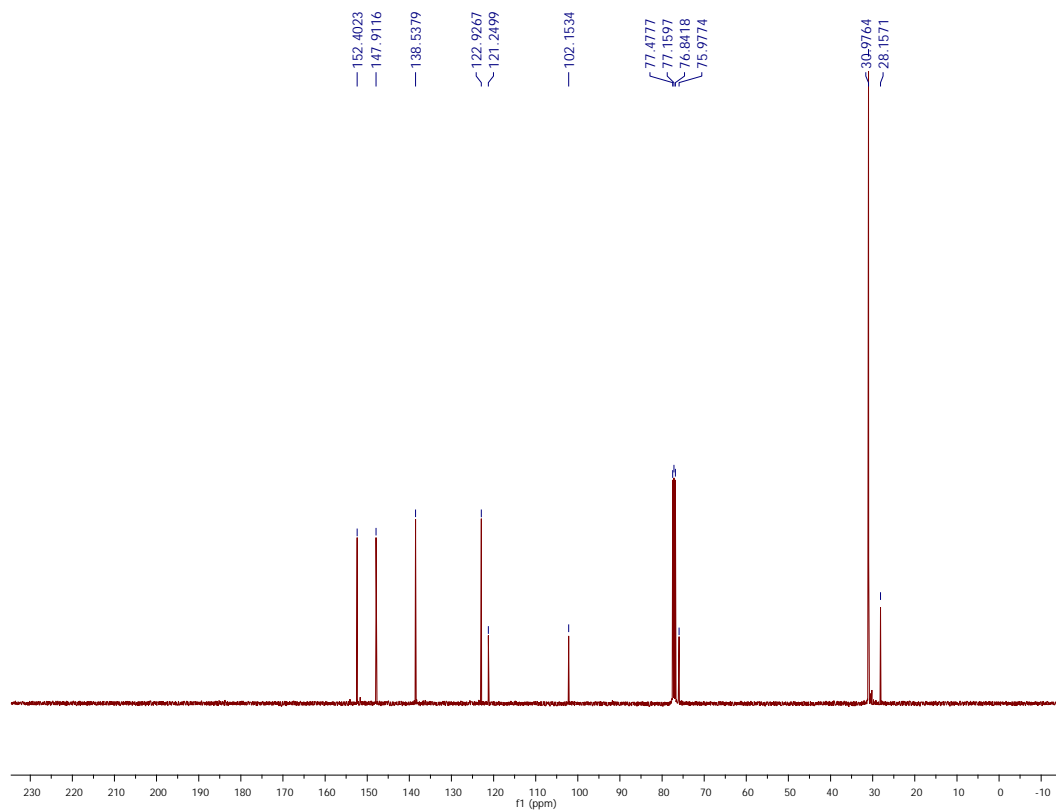

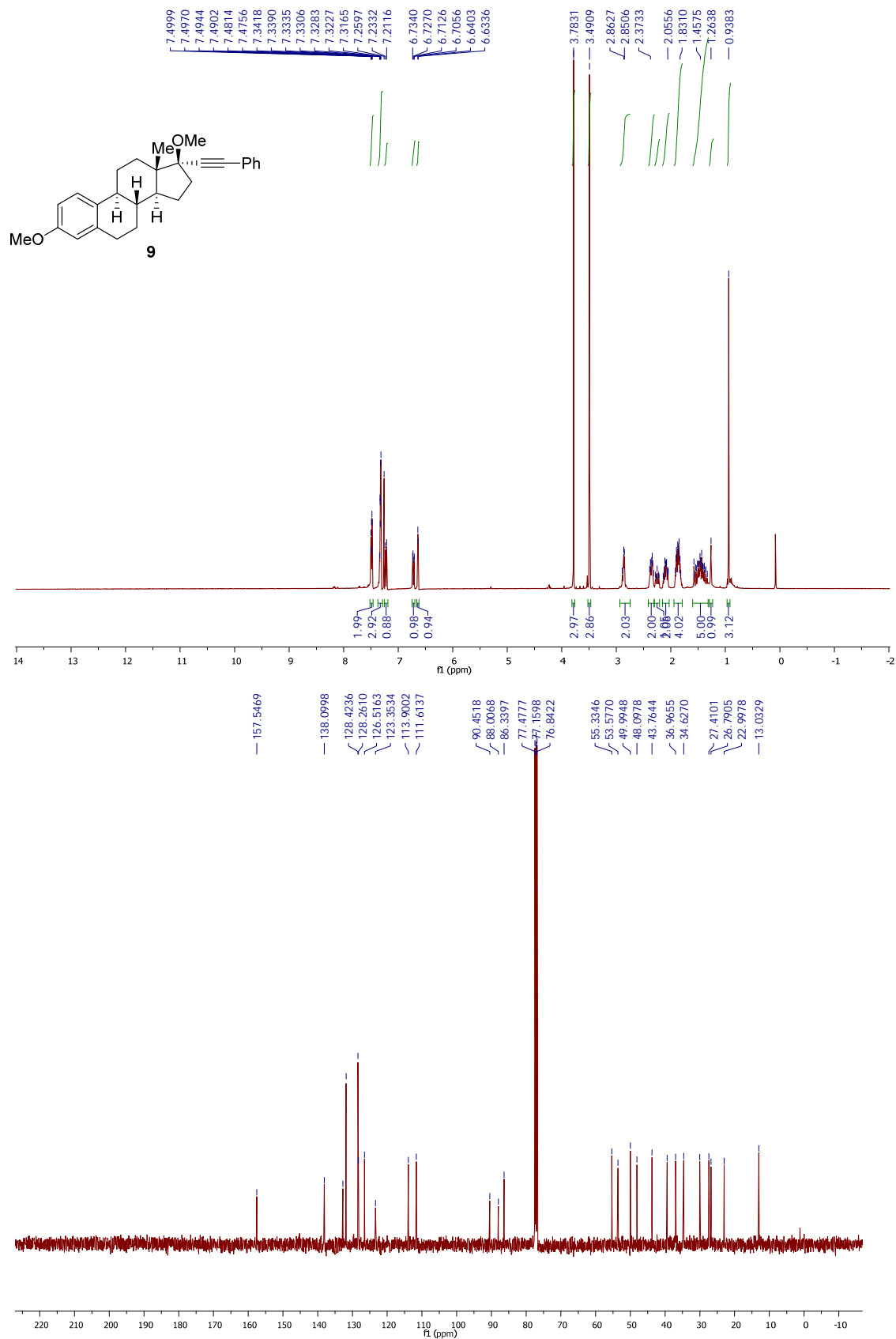

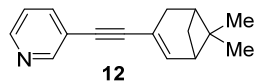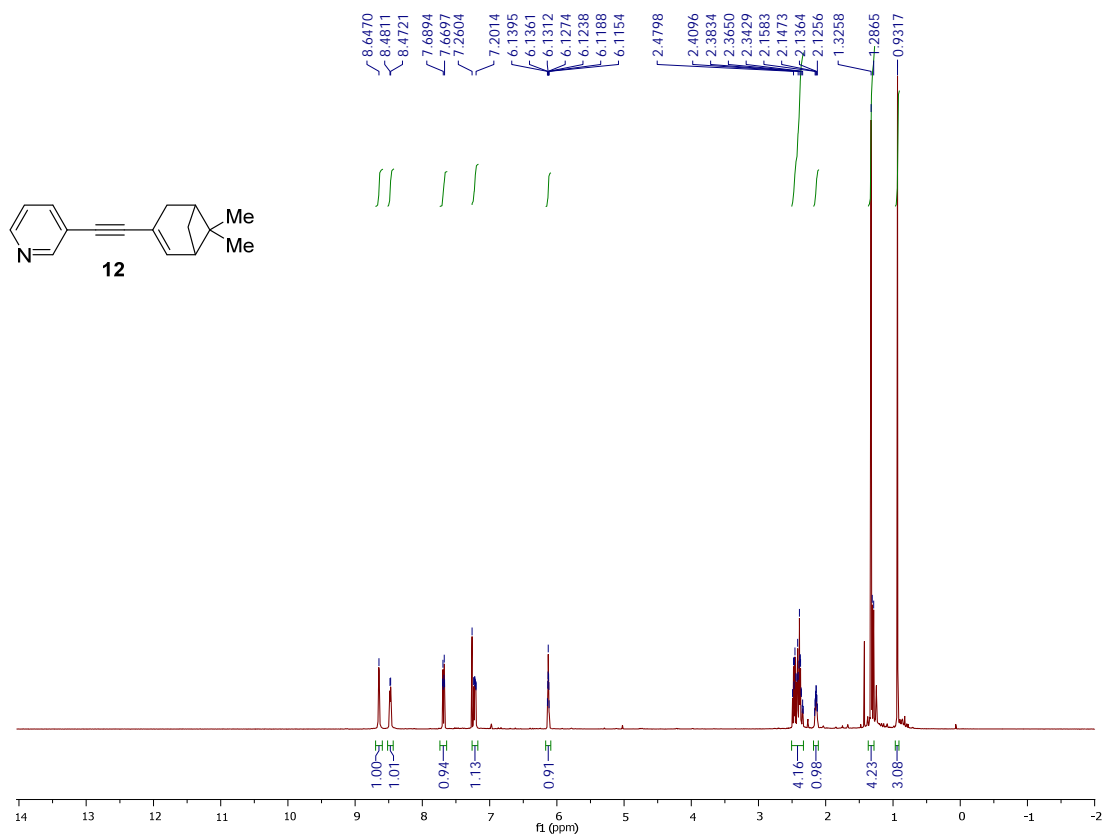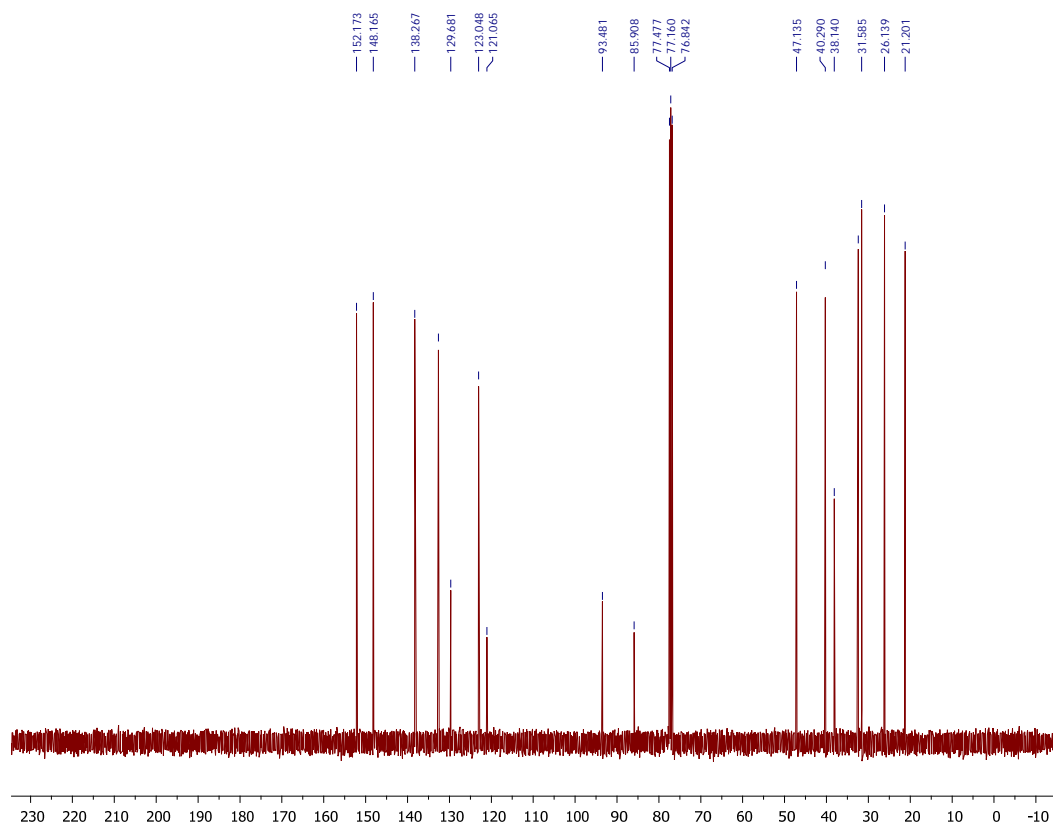

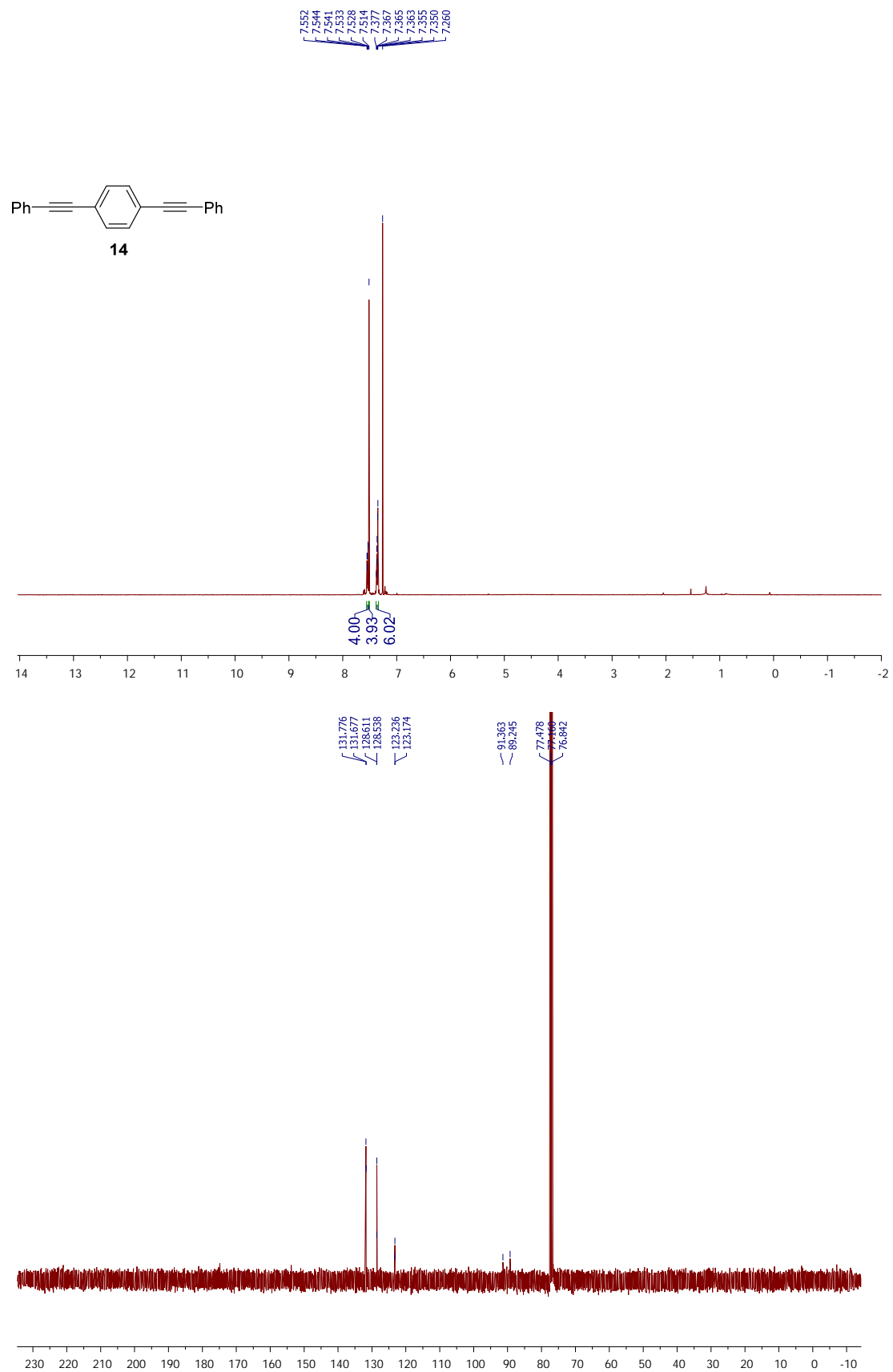

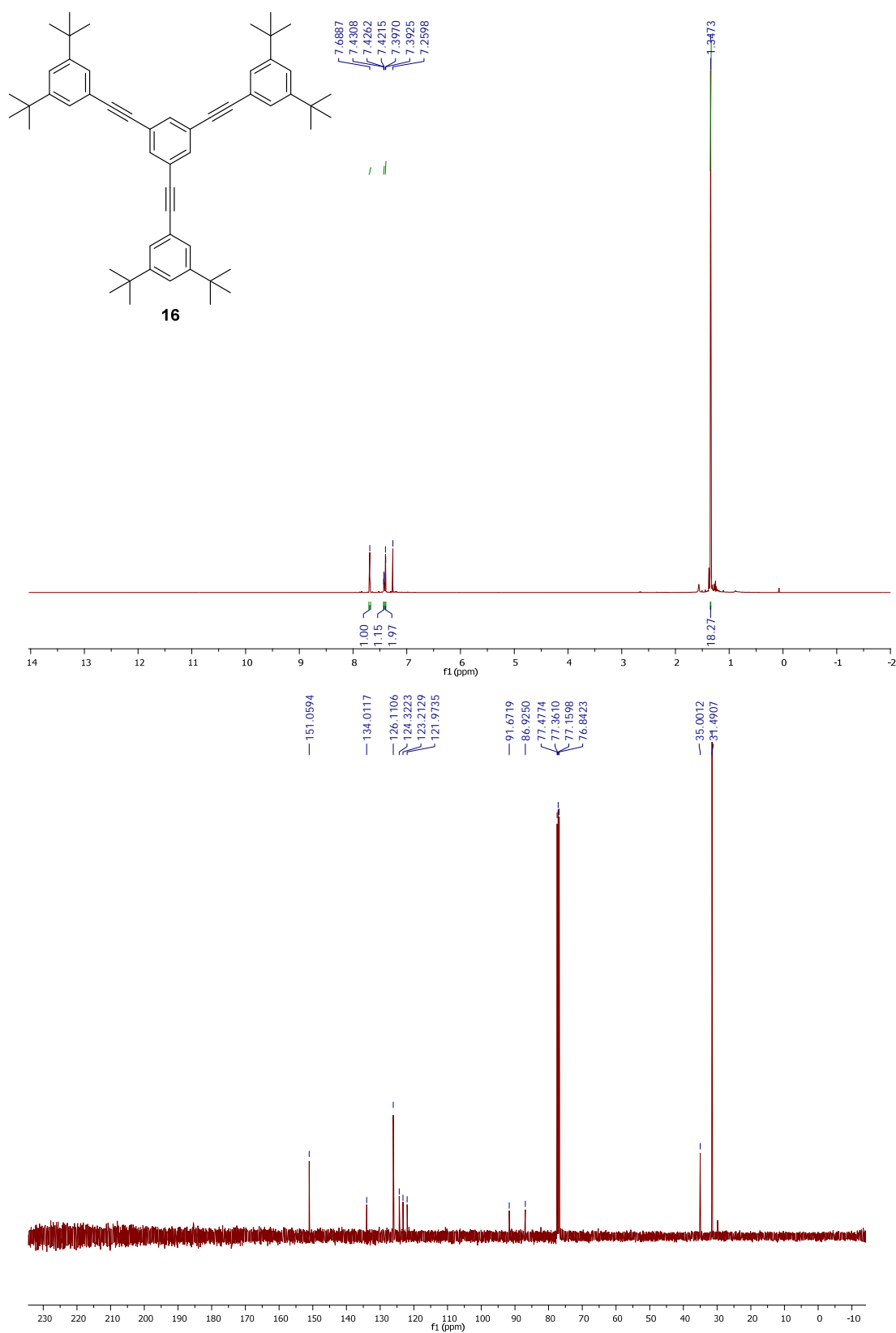

Supplement: Supplementary file 1 [file SC-006-C5SC00584A-s001.pdf]
