# Supplementary material for: Rh-catalyzed decarbonylation of conjugated ynones via carbon–alkyne bond activation: reaction scope and mechanistic exploration via DFT calculations
Source: Chem Sci. 2015 Mar 31;6(5):3201–10. doi: 10.1039/c5sc00584a (PMC4517480; doi:10.1039/c5sc00584a)
Supplement: Supplementary file 2 [file SC-006-C5SC00584A-s002.pdf]

Electronic Supplementary Material (ESI) for Chemical Science.
This journal is © The Royal Society of Chemistry 2015

Supporting Information

Rh-Catalyzed Decarbonylation of Conjugated Ynones via Carbon−Alkyne Bond Activation: Reaction Scope and Mechanistic Exploration via DFT Calculations

Alpay Dermenci[†Δ], Rachel E. Whittaker[†Δ], Yang Gao[‡§], Faben Cruz[Δ], Zhi-Xiang Yu[*‡], and Guangbin Dong[*Δ]

[Δ] The University of Texas at Austin, Department of Chemistry, Austin, TX 78712, United States
Current Address: Pfizer Inc., Eastern Point Road, Groton, CT 06340, United States

[†] AD and REW contributed equally

[‡] Beijing National Laboratory of Molecular Sciences (BNLMS), Key Laboratory of Bioorganic Chemistry and Molecular Engineering, College of Chemistry, Peking University, Beijing, 100871, China

[§] Key Laboratory of Pesticide & Chemical Biology, Ministry of Education, College of Chemistry, Central China Normal University, Hubei, Wuhan 430079, China

Section 1: Experimental Details

Page S2: General Information

Page S3: Additional Screening Data (Table S1)

Page S3-S21: Characterization Data for Substrates and Products

Page S22: Cycloisomerization to Furan Product (Scheme S1)

Page S22-S23: References

Section 2: Computational details

Page S24: Part I. Computed Energies of All Stationary Points

Page S26: Part II.

Page S26: Discussion of Different Structures of INT1-A

Page S27: Discussion of TS1

Page S28: Discussion of the Difference of Reactivities between Different Ligands: Xantphos vs dppp

Page S29-S137: Part III. Coordinates of All Stationary Points

Page S29-S78: Coordinates of all stationary points in figure 2

Page S79-S83: Coordinates of TS3-A-I versus TS3-A-II

Page S84-S92: Coordinates of TS3-deCO versus TS3-A

Page S93-S130: Coordinates of all stationary points of 1t in figure 3

Page S131-S137: Coordinates of radical structures in figure 4

Page S138-S150: Coordinates of radical structures in figure S1

Page S151-S172: Coordinates of radical structures in figure S2

Page S173-S205: Coordinates of radical structures in figure S3

Section 3: NMR Spectra

Page S206-S263: 1H and 13C NMR Spectra

Section 1: Experimental Details

General Information

Unless otherwise noted, all experiments were carried out under an inert atmosphere in a nitrogen-filled glovebox or by standard Schlenk techniques. Dichloromethane (CH_2Cl_2) was purified using a Pure-Solv MD-5 Solvent Purification System (Innovative Technology). Tetrahydrofuran (THF) was distilled over Na prior to use. Chlorobenzene, xylenes, and ethyl benzene were distilled over CaH_2 and degassed via freeze-pump-thaw (3x) and stored in the glovebox prior to use. All other reagents were used directly from the supplier without further purification unless noted. 4-phenylbut-3-yn-2-one **7p** bought from TCI America. Glass reaction vessels were either flame-dried or dried in the oven (140 ^{o}C) overnight. Analytical thin-layer chromatography (TLC) was carried out using 0.2 mm commercial silica gel plates (silica gel 60, F254, EMD chemical). Infrared spectra were recorded on a Nicolet 380 FTIR using neat thin film technique.
High-resolution mass spectra (HRMS) were obtained on a Karatos MS9 and are reported as m/z (relative intensity). Accurate masses are reported for the molecular ion $[M+Na]^+$, $[M+H]^+$, or [M+]. Nuclear magnetic resonance spectra (1H NMR and 13C NMR) were recorded with a Varian Gemini (400 MHz, ^{1}H at 400 MHz, ^{13}C at 100 MHz). For $CDCl_3$ solutions the chemical shifts are reported as parts per million (ppm) referenced to residual protium or carbon of the solvents; $CDCl_3$ δ ^{1}H (7.26 ppm) and $CDCl_3$ δ ^{13}C (77.0 ppm). Coupling constants are reported in Hertz (Hz). Data for ^{1}H NMR spectra are reported as follows: chemical shift (ppm, referenced to protium; s = singlet, d = doublet, t = triplet, q = quartet, sep = septet, dd = doublet of doublets, dq =doublet of quartets, td = triplet of doublets, tt=triplet of triplets, qd = quartet of doublets, ddd = doublet of doublet of doublets, m = multiplet, coupling constant (Hz), and integration). Ligand

abbreviations: triphos = Bis(diphenylphosphinoethyl)phenylphosphine, BINAP = 2, 2'-Bis(diphenylphosphino)-1,1'-binaphthyl, Xantphos= 4,5-Bis(diphenylphosphino)-9,9'-dimethylxanthene, dppf = 1,1'-Bis(diphenylphosphino)ferrocene.

Table S1: Additional Screening for Conditions

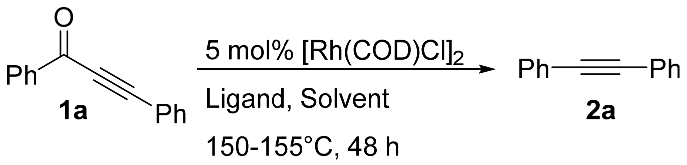

Entry	Ligand (12 mol%)	Solvent	Additive (mol%)	Yield[a]
1	triphos	xylenes	-	<5%
2	BINAP	xylenes	-	<5%
3	-[b]	xylenes	-	43% (82%)
4	Xantphos (16%)	xylenes	-	40%
5	Xantphos (20%)	xylenes	-	55%
6	dppf	*n*Bu_2O	-	NR
7	dppf	*n*Bu_2O	$ZnCl_2$ (20)	NR
8	dppf	*n*Bu_2O	$In(OTf)_3$ (20)	NR
9	dppf	*n*Bu_2O	$YbCl_3$ (20)	Decomp.
10	dppf	xylenes	Ph_3B (20)	NR
11	dppf	xylenes	$RuCl_2(PPh_3)_3$ (10)[c]	NR
12	dppf	xylenes	$[Ru(C_6H_6)Cl_2]_2$ (5)[c]	NR
13	dppf	xylenes	$[(p\text{-cymene})RuCl_2]_2$ (5)[c]	NR
14	dppf[d]	xylenes	-	NR
15	dppf[e]	xylenes	-	NR

[a] Isolated yields; Percent in parentheses is yield based on recovered starting material, [b] Pre-formed RhXantphos(COD)Cl was used as the catalyst, [c] [Ru] used as co-catalyst with $[Rh(COD)Cl]_2$ [d] $[Rh(COE)_2Cl]_2$ was used as the pre-catalyst, [e] $[Rh(C_2H_4)_2Cl]_2$ was used as the pre-catalyst

General Procedure A:[1]

Preparation of 1,3-diphenylprop-2-yn-1-one (1a)[2]

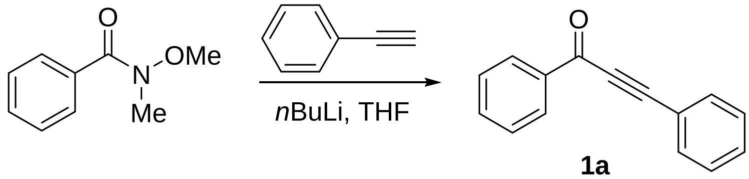

1a

To a stirring solution of phenylacetylene (1.79 g, 17.48 mmol) in THF (10 mL) at -78 °C was added *n*-BuLi (2.5 M, 6.99 mL). The reaction was stirred 15 min at 0 °C and added to a solution of Weinreb amide (2.75 g, 16.65 mmol) in THF (20 mL) at -78 °C. The reaction was warmed to r.t. and stirred for 3 hours. The reaction was quenched with H_2O and extracted with Et_2O. The combined organic layers were washed with $NaHCO_3$, H_2O, brine, dried over Na_2SO_4 and concentrated under reduced pressure. The crude material was purified via silica gel column chromatography (10:1→3:1 hexanes:EtOAc) to give a white solid in 85% yield (2.91 g).
^{1}H NMR (400 MHz, $CDCl_3$): δ 8.25-8.22 (m, 2H), 7.72-7.69 (m, 2H), 7.63 (tt, *J* = 7.2, 1.4 Hz, 1H), 7.55-7.41 (m, 5H). Mass calculated $[M+H]^+$: 207.0810, HRMS $[M+H]^+$: 207.0808.

The following compounds were prepared using General Procedure A. **1a-1d**, **1m-1n**, **1q**, **3a-3b**, **3e**, **3j-3l**, **3n-3o**, and **13** are known compounds from literature.

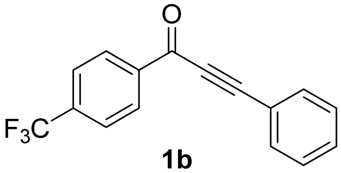

1b

3-phenyl-1-(4-(trifluoromethyl)phenyl)prop-2-yn-1-one (1b)[3]
Isolated as a white solid (859 mg, 3.13 mmol, 79%). ^{1}H NMR (400 MHz, $CDCl_3$): δ 8.32 (d, *J* = 8.0 Hz, 2H), 7.79 (d, *J* = 8.4 Hz, 2H), 7.69 (dt, *J* = 6.6, 1.6 Hz, 2H), 7.52 (tt, *J* = 6.4, 1.2 Hz, 1H), 7.46-7.43 (m, 3H). Mass calculated $[M+H]^+$: 275.0684, HRMS $[M+H]^+$: 275.0684.

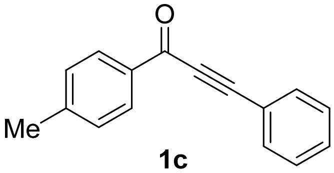

1c

3-phenyl-1-(p-tolyl)prop-2-yn-1-one (1c)[4]
Isolated as a white solid (159 mg, 0.72 mmol, 60%). ^{1}H NMR (400 MHz, $CDCl_3$): δ 8.12 (dt, *J* = 8.0, 1.6 Hz, 2H), 7.69 (dt, *J* = 7.2, 1.2 Hz, 2H), 7.50-7.40 (m, 3H), 7.31 (d, *J* = 8.4 Hz, 2H), 2.45 (s, 3H). Mass calculated $[M^+]$: 220.0888, HRMS $[M^+]$: 220.0890.

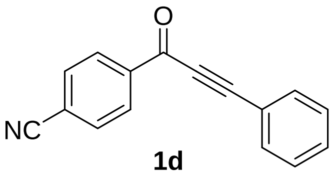

1d

4-(3-phenylpropioloyl)benzonitrile (1d)[5]
Isolated as a white solid (354 mg, 1.53 mmol, 49%). ^{1}H NMR (400 MHz, $CDCl_3$): δ 8.31 (dt, *J* = 8.4, 1.6 Hz, 2H), 7.83 (dt, *J* = 8.4, 1.6 Hz, 2H), 7.71-7.69 (m, 2H), 7.53 (tt, *J* = 7.6, 1.2 Hz, 1H), 7.45 (tt, *J* = 7.6, 1.2 Hz, 2H). Mass calculated $[M^+]$: 231.0684, HRMS $[M^+]$: 231.0685.

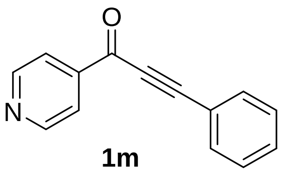

1m

3-phenyl-1-(pyridin-4-yl)prop-2-yn-1-one (1m)[12]
Isolated as a yellow solid (188 mg, 0.91 mmol, 76%). ^{1}H NMR (400 MHz, $CDCl_3$): δ 8.88 (dd, *J* = 4.8, 1.5 Hz, 2H), 8.00 (dd, *J* = 4.4, 1.7 Hz, 2H), 7.73-7.70 (m, 2H), 7.56-7.44 (m, 3H). Mass calculated $[M+H]^+$:

208.0762, HRMS $[M+H]^+$: 208.0767.

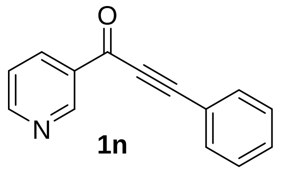

1n

1,3-diphenylprop-2-yn-1-one (1n)[13]
Isolated as a pale yellow solid (485 mg, 2.35 mmol, 78%). ^{1}H NMR (400 MHz, $CDCl_3$): δ 9.44 (d, *J* = 2.4 Hz, 1H), 8.84 (dd, *J* = 4.8, 1.6 Hz, 1H), 8.42 (dt, *J* = 8.0, 2.0 Hz, 1H), 7.69 (d, *J* = 6.8 Hz, 2H), 7.53-7.42 (m, 4H). Mass calculated $[M+H]^+$: 208.0762, HRMS $[M+H]^+$: 208.0767.

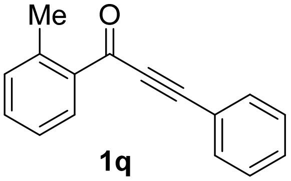

1q

3-phenyl-1-(o-tolyl)prop-2-yn-1-one (1q)[40]
Isolated as a yellow oil (687 mg, 3.12 mmol, 56%). ^{1}H NMR (400 MHz, $CDCl_3$): δ 8.31 (d, *J* = 7.0 Hz, 1H), 7.66 (dd, *J* = 6.2, 0.6 Hz, 2H), 7.47-7.35 (m, 5H), 7.27 (d, *J* = 1.5 Hz, 1H), 2.69 (s, 3H). Mass calculated $[M+H]^+$: 332.9776, HRMS $[M+H]^+$: 332.9773.

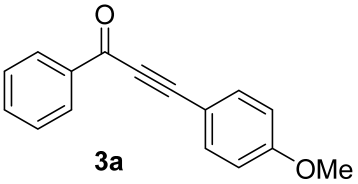

3a

3-(4-methoxyphenyl)-1-phenylprop-2-yn-1-one (3a)[40]
Isolated as a white solid (235 mg, 1.00 mmol, 83%). ^{1}H NMR (400 MHz, $CDCl_3$): δ 8.22 (dd, *J* = 8.0, 1.2 Hz, 2H), 7.67-7.61 (m, 3H), 7.52 (td, *J* = 4.8, 1.2 Hz, 2H), 6.94 (dt, *J* = 6.4, 2.0 Hz, 2H), 3.87 (s, 3H). Mass calculated $[M+H]^+$: 237.0916, HRMS $[M+H]^+$: 237.0911.

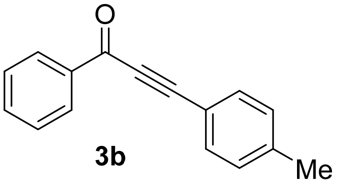

3b

1-phenyl-3-(p-tolyl)prop-2-yn-1-one (3b)[41]
Isolated as a white solid (210 mg, 0.96 mmol, 79%). ^{1}H NMR (400 MHz, $CDCl_3$): δ 8.23 (dd, *J* = 8.4, 1.2 Hz, 2H), 7.63-7.59 (m, 3H), 7.52 (td, *J* = 8.4, 1.2 Hz, 2H), 7.25-7.23 (m, 2H), 2.42 (s, 3H). Mass calculated $[M+H]^+$: 221.0966, HRMS $[M+H]^+$: 221.0966.

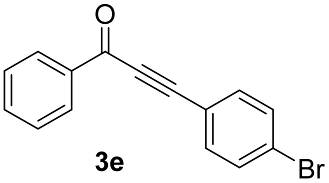

3e

3-(4-bromophenyl)-1-phenylprop-2-yn-1-one (3e)[42]
Isolated as a yellow oil (231 mg, 0.81 mmol, 67%). ^{1}H NMR (400 MHz, $CDCl_3$): δ 8.25 (dd, *J* = 4.8, 1.6 Hz, 2H), 7.72-7.65 (m, 3H), 7.56-7.44 (m, 4H). Mass calculated $[M+H]^+$: 284.9915, HRMS $[M+H]^+$: 284.9913.

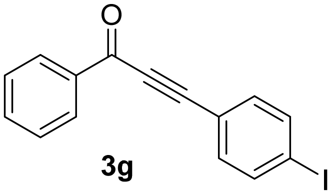

3g

3-(4-iodophenyl)-1-phenylprop-2-yn-1-one (3g)
Isolated as a cream solid (172 mg, 0.52 mmol, 67%). ^{1}H NMR (400 MHz, $CDCl_3$): δ 8.20 (dt, *J* = 8.4, 1.4 Hz, 2H), 7.78 (dt, *J* = 8.5, 1.9 Hz, 2H), 7.64 (tt, *J* = 7.4, 1.9 Hz, 1H), 7.53 (tt, *J* = 8.0, 1.6 Hz, 2H), 7.39 (dt, *J* = 8.5, 2.0 Hz, 2H). ^{13}C NMR (100 MHz, $CDCl_3$): δ 177.9, 138.1, 136.8, 134.4, 134.4, 134.3, 134.3, 129.7, 128.8, 119.7, 97.8, 88.0. IR: ν 3413, 1264, 3207, 3056, 2926, 2653, 2204, 1631, 1575, 1471, 1386, 1319, 1286,

1209, 1170, 1007, 816, 695 cm^{-1}. Mass calculated $[M+H]^+$: 332.9776, HRMS $[M+H]^+$: 332.9774. MP: 118-120°C.

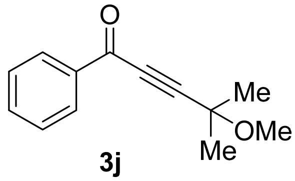

3j

4-methoxy-4-methyl-1-phenylpent-2-yn-1-one (3j)[39]
Isolated as a clear oil (105 mg, 0.52 mmol, 43%). ^1H NMR (400 MHz, $CDCl_3$) δ 8.16-8.09 (m, 2H), 7.66-7.60 (m, 1H), 7.53-7.47 (m, 2H), 3.47 (s, 3H), 1.60 (s, 6H). Mass calculated $[M+H]^+$: 203.1072, HRMS $[M+H]^+$: 203.1074.

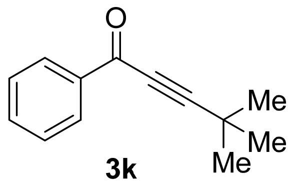

3k

4,4-dimethyl-1-phenylpent-2-yn-1-one (3k)[43]
Isolated as a clear oil (789 mg, 4.24 mmol, 70%). ^1H NMR (400 MHz, $CDCl_3$): δ 8.12 (dd, *J* = 8.5, 1.4 Hz, 2H), 7.59 (tt, *J* = 8.0, 1.3 Hz, 1H), 7.47 (tt, *J* = 7.8, 1.6 Hz, 2H), 1.38 (s, 9H). Mass calculated $[M+H]^+$: 187.1123, HRMS $[M+H]^+$: 187.1122.

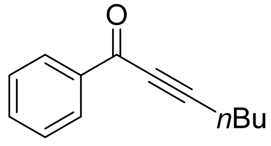

3l

1-phenylhept-2-yn-1-one (3l)[37]
Isolated as a clear oil (811 mg, 4.36 mmol, 72%). ^1H NMR (400 MHz, $CDCl_3$): δ 8.15-8.12 (m, 2H), 7.69 (tt, *J* = 7.4, 1.2 Hz, 1H), 7.47 (tt, *J* = 7.2, 1.6 Hz, 2H), 2.50 (t, *J* = 7.0 Hz, 2H), 1.70-1.62 (m, 2H), 1.55-1.46 (m, 2H), 0.96 (t, *J* = 7.2 Hz, 3H). Mass calculated $[M+H]^+$: 187.1123, HRMS $[M+H]^+$: 187.1123.

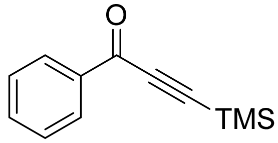

3n

1-phenyl-3-(trimethylsilyl)prop-2-yn-1-one (3n)[44]
Isolated as a clear oil (81 mg, 0.40 mmol, 70%). ^1H NMR (400 MHz, $CDCl_3$): δ 8.16-8.14 (m, 2H), 7.61 (tt, *J* = 5.6, 2.0 Hz, 1H), 7.49 (tt, *J* = 6.8, 1.6 Hz, 2H), 0.30 (s, 9H). Mass calculated $[M+H]^+$: 203.0892, HRMS $[M+H]^+$: 203.0893.

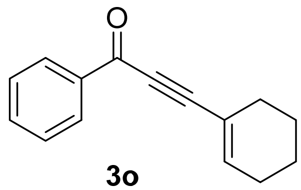

3o

3-(cyclohex-1-en-1-yl)-1-phenylprop-2-yn-1-one (3o)[37]
Isolated as an orange oil (726 mg, 3.45 mmol, 57%). ^1H NMR (400 MHz, $CDCl_3$): δ 8.15-8.13 (m, 2H), 7.59 (tt, *J* = 6.8, 1.4 Hz, 1H), 7.47 (tt, *J* = 7.6, 1.8 Hz, 2H), 6.58 (sept, *J* = 2.0 Hz, 1H), 2.30-2.18 (m, 4H), 1.74-1.61 (m, 4H). Mass calculated $[M+H]^+$: 210.1045, HRMS $[M+H]^+$: 210.1046.

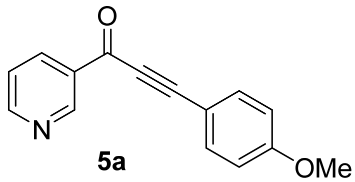

5a

3-(4-methoxyphenyl)-1-(pyridin-3-yl)prop-2-yn-1-one (5a)
Isolated as a pale yellow solid (242 mg, 1.02 mmol, 68%). ^1H NMR (400 MHz, $CDCl_3$): δ 9.41 (d, *J* = 1.6 Hz, 1H), 8.81 (dd, *J* = 4.8, 1.6 Hz, 1H), 8.41 (dt, *J* = 8.0, 2.0 Hz, 1H), 7.63 (dt, *J* = 9.2, 2.0 Hz, 2H), 7.45 (qd, *J* = 4.0, 0.8 Hz, 1H), 6.93 (dt, *J* = 8.8, 2.0 Hz, 2H),

3.85 (s, 3H). ^{13}C NMR (100 MHz, $CDCl_3$): δ 176.4, 162.2, 151.4, 136.2, 135.5, 132.4, 123.6, 114.6, 111.3, 96.2, 86.5, 55.5. IR: ν 3349, 2930, 2845, 2179, 1694, 1636, 1601, 1512, 1404 cm^{-1}. Mass calculated $[M+H]^+$: 238.0863, HRMS $[M+H]^+$: 238.0857. MP: 87-91°C.

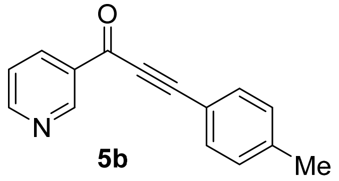

5b

1-(pyridin-3-yl)-3-(p-tolyl)prop-2-yn-1-one (5b)
Isolated as a white solid (159 mg, 0.72 mmol, 48 %). ^{1}H NMR (400 MHz, $CDCl_3$): δ 9.45 (s, 1H), 8.84 (dd, *J* = 4.8, 1.2 Hz, 1H), 8.44 (dt, *J* = 8.0, 2.0 Hz, 1H), 7.60 (dt, *J* = 8.4, 2.0 Hz, 2H), 7.47 (qd, *J* = 4.0, 0.8 Hz, 1H), 7.25 (d, *J* = 8.0 Hz, 2H), 2.43 (s, 3H). ^{13}C NMR (100 MHz, $CDCl_3$): δ 176.5, 154.2, 151.5, 142.2, 136.3, 133.4, 132.3, 129.7, 123.6, 116.5, 95.6, 86.3, 21.9. IR: ν 3415, 3256, 3190, 3035, 2915, 2849, 2446, 2202, 1636, 1578, 1504, 1404, 1299 cm^{-1}. Mass calculated $[M+H]^+$: 222.0913, HRMS $[M+H]^+$: 222.0910. MP: 113-115 °C.

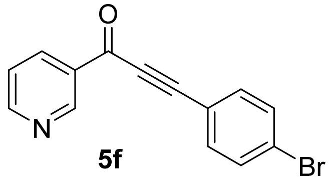

5f

3-(4-bromophenyl)-1-(pyridin-3-yl)prop-2-yn-1-one (5f)
Isolated as a light brown solid (110 mg, 0.39 mmol, 50%). ^{1}H NMR (400 MHz, $CDCl_3$): δ 9.42 (dd, *J* = 2.2, 1.0 Hz, 1H), 8.85 (dd, *J* = 4.8, 1.6 Hz, 1H), 8.41 (dt, *J* = 8.0, 2.0 Hz, 1H), 7.61-7.55 (m, 4H), 7.48 (qd, *J* = 4.0, 0.8 Hz, 1H). ^{13}C NMR (100 MHz, $CDCl_3$): δ 176.4, 154.5, 151.6, 136.3, 134.6, 132.4, 132.2, 126.3, 123.8, 118.6, 93.4, 87.2. IR: ν 3526, 3084, 3051, 2918, 2843, 2201, 1902, 1635, 1577, 1491 cm^{-1}. Mass calculated $[M^+]$: 287.9847, HRMS $[M^+]$: 287.9855. MP: 125-128 °C.

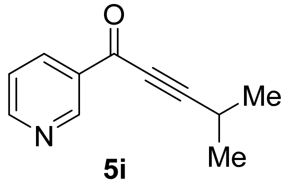

5i

4-methyl-1-(pyridin-3-yl)pent-2-yn-1-one (5i)
Isolated as a light brown oil (96 mg, 0.56 mmol, 37%). ^{1}H NMR (400 MHz, $CDCl_3$): δ 9.34 (d, *J* = 1.6 Hz, 1H), 8.81 (dd, *J* = 4.6, 1.4 Hz, 1H), 8.35 (dt, *J* = 7.6, 2.0 Hz, 1H), 7.44 (qd, *J* = 3.8, 0.6 Hz, 1H), 2.89 (sep, *J* = 7.0 Hz, 1H), 1.34 (d, *J* = 6.4 Hz, 6H). ^{13}C NMR (100 MHz, $CDCl_3$): δ 176.8, 154.1, 151.6, 136.4, 132.4, 123.6, 103.4, 78.4, 22.1, 21.2. IR: ν 2973, 2923, 2868, 2210, 2167, 1656, 1578, 1415, 1318, 1260 cm^{-1}. Mass calculated $[M+H]^+$: 174.0913, HRMS $[M+H]^+$: 174.0911.

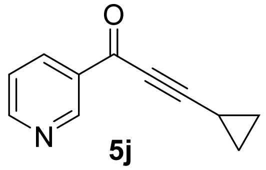

5j

3-cyclopropyl-1-(pyridin-3-yl)prop-2-yn-1-one (5j)
Isolated as an orange oil (103 mg, 0.60 mmol, 40%). ^{1}H NMR (400 MHz, $CDCl_3$): δ 9.25 (d, *J* = 1.6 Hz, 1H), 8.75 (dd, *J* = 4.8, 1.6 Hz, 1H), 8.28 (dt, *J* = 8.0, 2.0 Hz, 1H), 7.38 (qd, *J* = 4.0, 0.8 Hz, 1H), 1.56-1.49 (m, 1H), 1.08-1.00 (m, 4H). ^{13}C NMR (100 MHz, $CDCl_3$): δ 176.4, 154.1, 151.5, 144.7, 136.2, 123.5, 107.8, 83.4, 10.3, 0.2. IR: ν 2907, 2845, 2202, 1632, 1586, 1419, 1361, 1276, 1194 cm^{-1}. Mass calculated $[M+H]^+$: 172.0762, HRMS $[M+H]^+$: 172.0759.

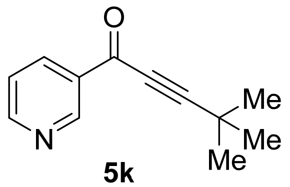

5k

4,4-dimethyl-1-(pyridin-3-yl)pent-2-yn-1-one (5k)

Isolated as a light brown solid (177 mg, 0.95 mmol, 63%). ^{1}H NMR (400 MHz, $CDCl_3$): δ 9.31 (dd, J = 2.2, 0.8 Hz, 1H), 8.79 (dd, J = 4.8, 1.8 Hz, 1H), 8.35-8.32 (m, 1H), 7.42 (qd, J = 4.0, 0.9 Hz, 1H), 1.38 (s, 9H). ^{13}C NMR (100 MHz, $CDCl_3$): δ 176.7, 153.8, 151.3, 136.5, 132.5, 123.7, 106.0, 77.7, 30.2, 28.3. IR: ν 3442, 3275, 3039, 2965, 2930, 2899, 2876, 2206, 1644, 1582, 1470, 1454, 1408 cm^{-1}. Mass calculated $[M+H]^+$: 188.1070, HRMS $[M+H]^+$: 188.1072. MP: 54-57°C.

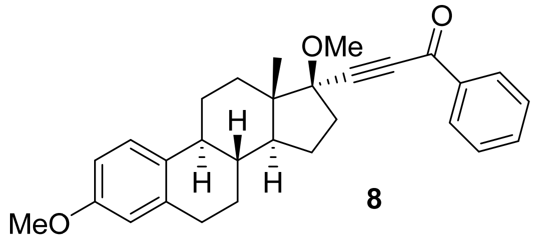

8

3-((8*R*,9*S*,13*S*,14*S*,17*S*)-3,17-dimethoxy-13-methyl-7,8,9,11,12,13,14,15,16,17-decahydro-6*H*-cyclopenta[*a*]phenanthren-17-yl)-1-phenylprop-2-yn-1-one (8)

Isolated as a clear, amorphous solid (503 mg, 1.18 mmol, 74%). ^{1}H NMR (400 MHz, $CDCl_3$): δ 8.19 (d, J = 7.2 Hz, 2H), 7.66-7.62 (m, 1H), 7.54- 7.50 (m, 2H), 7.22 (d, J = 8.4 Hz, 1H), 6.74-6.72 (m, 1H), 6.66 (s, 1H), 3.80 (s, 3H), 3.55 (s, 3H), 2.92-2.88 (m, 2H), 2.48-2.36 (m, 2H), 2.32-2.27 (m, 1H), 2.17-1.86 (m, 6H), 1.60-1.41 (m, 4H), 0.98 (s, 3H). ^{13}C NMR (100 MHz, $CDCl_3$): δ 177.7, 157.6, 137.9, 137.0, 134.2, 132.4, 129.6, 128.8, 126.5, 113.9, 111.6, 95.8, 86.4, 86.3, 55.3, 54.1, 50.3, 48.6, 43.6, 39.3, 36.4, 34.6, 29.9, 27.4, 26.6, 23.0, 12.9. IR: ν 3273, 3054, 2931, 2873, 2823, 2207, 1641, 1613, 1591, 1576, 1432 cm^{-1}. Mass calculated $[M+Na]^+$: 451.2244, HRMS $[M+Na]^+$: 451.2241.

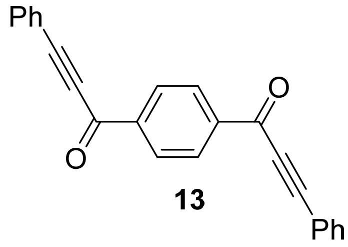

13

1,1'-(1,4-phenylene)bis(3-phenylprop-2-yn-1-one) (13)[15]

Isolated as a white solid (273 mg, 0.82 mmol, 49%). ^{1}H NMR (400 MHz, $CDCl_3$): δ 8.36 (s, 4H), 7.72 (dt, J = 5.2, 1.6 Hz, 4H), 7.55-7.51 (m, 2H), 7.45 (td, J = 6.0, 1.6 Hz, 4H). Mass calculated $[M^+]$: 334.0994, HRMS $[M^+]$: 334.0991.

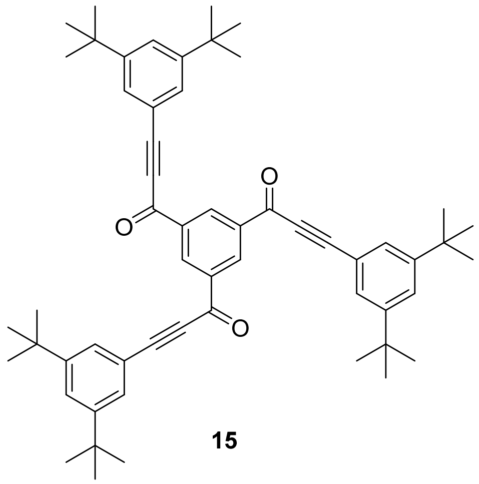

15

1,1',1''-(benzene-1,3,5-triyl)tris(3-(3,5-di-tert-butylphenyl)p-rop-2-yn-1-one) (15)

Isolated as a light brown foam (86 mg, 0.11 mmol, 26%). ^{1}H NMR (400 MHz, $CDCl_3$): δ 9.35 (s, 3H), 7.58 (d, J = 1.84 Hz, 6H), 7.55 (q, J = 1.8 Hz, 3H), 1.28 (s, 54H). ^{13}C NMR (100 MHz, $CDCl_3$): δ 176.1, 151.5, 138.1, 134.6, 127.9, 126.3, 118.4, 97.4, 85.9, 34.6, 31.1. IR: ν 2961, 2903, 2849, 2194, 1648, 1582, 1462, 1423, 1361 cm^{-1}. Mass calculated $[M^+]$: 798.5012, HRMS $[M^+]$: 798.5004.

General Procedure B:

Preparation of 1-(4-fluorophenyl)-3-phenylprop-2-yn-1-one (1e)[2,6,7]

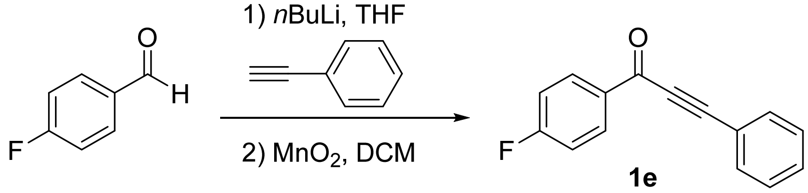

To a stirring solution of phenylacetylene (118 mg, 1.16 mmol) in THF (6 mL) at -78 °C was added *n*-BuLi (2.5 M, 0.46 mL). The reaction was stirred 15 min at 0 °C and added to a solution of aldehyde (137 mg, 1.10 mmol) in THF (10 mL) at -78 °C. The reaction was warmed to r.t. and stirred for 3 hours. The reaction was quenched with H_2O and extracted with Et_2O. The combined organic layers were washed with $NaHCO_3$, H_2O, brine, dried over Na_2SO_4 and concentrated under reduced pressure. Without purification, the crude material was redissolved in dichloromethane and manganese dioxide (1.44 g, 16.57 mmol) was added at room temperature. The reaction mixture was stirred for 1 hour and then filtered through celite and concentrated under reduced pressure. The crude material was purified via silica gel column chromatography (10:1→3:1 hexanes:EtOAc) to give a white solid in 68% yield (168 mg).

The following compounds were prepared using General Procedure B. **1e-1j**, **1l**, **1o-1p**, and **1r-1s** are known compounds from literature.

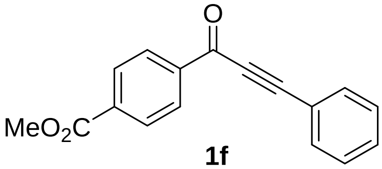

1f

Methyl 4-(3-phenylpropioloyl)benzoate (1f)[8]

Isolated as a white solid (234 mg, 0.88 mmol, 74%). ^{1}H NMR (400 MHz, $CDCl_3$): δ 8.27 (dd, *J* = 8.4, 2.0 Hz, 2H), 8.18 (dd, *J* = 6.8, 2.0 Hz, 2H) 7.71 (dt, *J* = 6.8, 1.2 Hz, 2H), 7.51 (m, 1H), 7.45 (m, 2H), 3.97 (s, 3H). Mass calculated [M^+]: 265.0865, HRMS [M^+]: 265.0864.

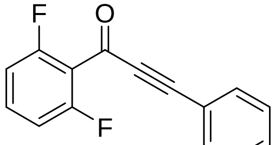

1g

1-(2,6-difluorophenyl)-3-phenylprop-2-yn-1-one (1g)[9]

Isolated as a light yellow solid (202 mg, 0.84 mmol, 60%). ^{1}H NMR (400 MHz, $CDCl_3$): δ 7.63 (dt, *J* = 7.2, 1.6 Hz, 2H), 7.51-7.46 (m, 2H), 7.44-7.38 (m, 2H), 7.00 (tt, *J* = 8.4, 2.0 Hz, 2H). Mass calculated $[M+H]^+$: 243.0621, HRMS $[M+H]^+$: 243.0621.

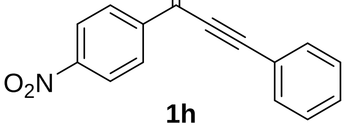

1h

1-(4-nitrophenyl)-3-phenylprop-2-yn-1-one (1h)[10]

Isolated as a bright yellow solid (482 mg, 1.92 mmol, 64%). ^{1}H NMR (400 MHz, $CDCl_3$): δ 8.88 (dd, *J* = 4.8, 1.5 Hz, 2H), 8.00 (dd, *J* = 4.4, 1.7 Hz, 2H), 7.73-7.70 (m, 2H), 7.56-7.44 (m, 3H). Mass calculated $[M+H]^+$: 252.0661, HRMS $[M+H]^+$: 252.0663.

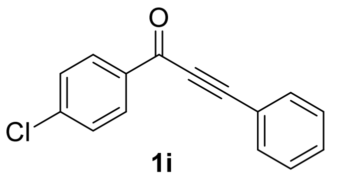

1i

1-(4-chlorophenyl)-3-phenylprop-2-yn-1-one (1i)[4]
Isolated as a white solid (254 mg, 1.05 mmol, 53%). ^{1}H NMR (400 MHz, $CDCl_3$): δ 8.16 (dt, *J* = 8.8, 1.8 Hz, 2H), 7.70-7.68 (m, 2H), 7.52-7.48 (m, 3H), 7.46-7.42 (m, 2H). Mass calculated $[M+H]^+$: 241.0420, HRMS $[M+H]^+$: 241.0419.

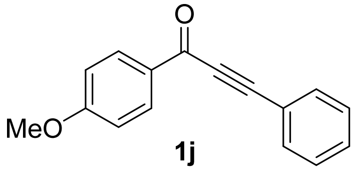

1j

1-(4-methoxyphenyl)-3-phenylprop-2-yn-1-one (1j)[2]
Isolated as a white solid (497 mg, 2.10 mmol, 65%). ^{1}H NMR (400 MHz, $CDCl_3$): δ 8.21-8.18 (m, 2H), 7.69-7.66 (m, 2H), 7.50-7.39 (m, 3H), 6.99 (dt, *J* = 9.1, 2.6 Hz, 2H), 3.91 (s, 3H). Mass calculated $[M+H]^+$: 237.0916, HRMS $[M+H]^+$: 237.0911.

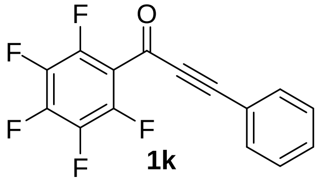

1k

1-(perfluorophenyl)-3-phenylprop-2-yn-1-one (1k)
Isolated as a pale yellow solid (617 mg, 2.08 mmol, 94%). ^{1}H NMR (400 MHz, $CDCl_3$): δ 7.65-7.63 (m, 2H), 7.52-7.50 (m, 1H), 7.45-7.40 (m, 2H). ^{13}C NMR (100 MHz): δ 167.8, 146.7 (m), 144.9 (m), 144.2 (m), 142.3 (m), 133.5, 131.7, 128.8, 119.1, 95.3 (t, *J* = 2.2 Hz), 88.7. ^{19}F NMR (377 MHz): δ -140.1 (m), -147.1 (m), -160.1 (m). IR: ν 2938, 2206, 2159, 1656, 1520, 1481 cm^{-1}. Mass calculated (M+H): 297.0339, HRMS (M+H): 297.0337. MP: 71-74°C.

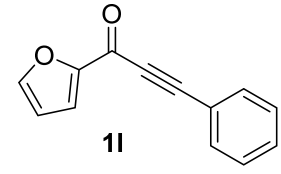

1l

1-(furan-2-yl)-3-phenylprop-2-yn-1-one (1l)[11]
Isolated as a white solid (1.32 g, 6.74 mmol, 70%). ^{1}H NMR (400 MHz, $CDCl_3$): δ 7.69 (t, *J* = 0.6 Hz, 1H), 7.64 (dt, *J* = 6.4, 1.6 Hz, 2H), 7.48 (t, *J* = 7.6 Hz, 1H), 7.44-7.39 (m, 3H), 6.60 (q, *J* = 1.6 Hz, 1H). Mass calculated $[M+H]^+$: 197.0603, HRMS $[M+H]^+$: 197.0600.

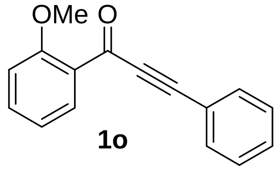

1o

1-(2-methoxyphenyl)-3-phenylprop-2-yn-1-one (1o)[14]
Isolated as a white solid (784 mg, 3.34 mmol, 76%). ^{1}H NMR (400 MHz, $CDCl_3$): δ 8.08 (dd, *J* = 8.0, 2.0 Hz, 1H), 7.63 (dd, *J* = 7.2, 1.2 Hz, 2H), 7.54 (td, *J* = 8.4, 2.0 Hz, 1H) 7.45-7.37 (m, 3H), 7.03 (q, *J* = 8.4 Hz, 2H), 3.96 (s, 3H). Mass calculated $[M+H]^+$: 237.0916, HRMS $[M+H]^+$: 237.0920.

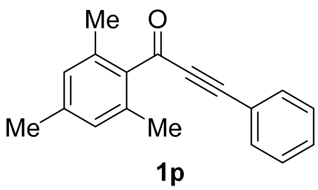

1p

1-mesityl-3-phenylprop-2-yn-1-one (1p)[37]
Isolated as a clear oil (165 mg, 0.66 mmol, 91%). ^{1}H NMR (400 MHz, $CDCl_3$): δ 7.57 (dt, *J* = 6.8, 1.6 Hz, 2H), 7.45 (tt, *J* = 6.4, 1.6 Hz, 1H), 7.37 (tt, *J* = 6.4, 1.6 Hz, 2H), 6.89 (s, 2H), 2.42, (s, 6H), 2.31 (s, 3H). Mass calculated $[M+H]^+$: 249.1279, HRMS $[M+H]^+$: 249.1278.

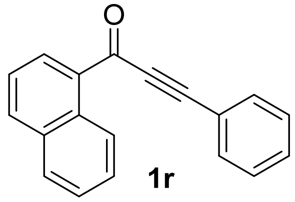

1r

1-(naphthalen-1-yl)-3-phenylprop-2-yn-1-one (1r)[4]
Isolated as a white solid (532 mg, 2.07 mmol, 72%). ^{1}H NMR (400 MHz, $CDCl_3$): δ 9.24 (d, *J* = 8.8 Hz, 1H), 8.65 (dd, *J* = 3.2, 1.2 Hz, 1H), 8.10 (d, *J* = 8.4 Hz, 1H), 7.92 (d, *J* = 8.4 Hz, 1H), 7.71-7.67 (m, 3H), 7.63- 7.57 (m, 2H), 7.51-7.41 (m, 3H). Mass calculated $[M+H]^+$: 257.0966, HRMS $[M+H]^+$: 257.0968.

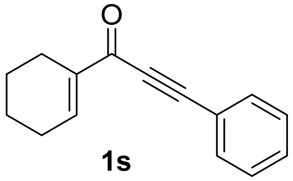

1s

1-(cyclohex-1-en-1-yl)-3-phenylprop-2-yn-1-one (1s)[4]
Isolated as a clear oil (845 mg, 4.02 mmol, 68%). ^{1}H NMR (400 MHz, $CDCl_3$): δ 7.58 (dt, *J* = 5.6, 1.6 Hz, 2H), 7.46-7.41 (m, 2H), 7.39-7.35 (m, 2H), 2.37-2.29 (m, 4H), 1.68-1.64 (m, 4H). Mass calculated $[M+H]^+$: 211.1123, HRMS $[M+H]^+$: 211.1120.

General Procedure C:
Preparation of 4-(3-oxo-3-phenylprop-1-yn-1-yl)benzonitrile (3c)

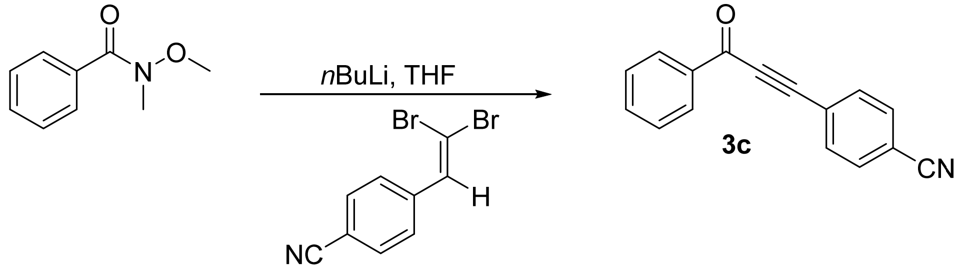

To a stirring solution of dibromoalkene (365 mg, 1.27 mmol) in THF (4 mL) at -78 °C was added *n*-BuLi (2.5 M, 1.02 mL). The reaction was stirred 15 min at -78 °C and added to a solution of Weinreb amide (200 mg, 1.21 mmol) in THF (8 mL) at -78 °C. The reaction was warmed to r.t. and stirred for 3 hours. The reaction was quenched with H_2O and extracted with Et_2O. The combined organic layers were washed with $NaHCO_3$, H_2O, brine, dried over Na_2SO_4 and concentrated under reduced pressure. The crude material was purified via silica gel column chromatography (10:1→3:1 hexanes:EtOAc) to give a white solid in 78% yield (218 mg).
White solid. ^{1}H NMR (400 MHz, $CDCl_3$): δ 8.20 (dt, *J* = 8.0, 1.6 Hz, 2H), 7.79-7.72 (m, 4H), 7.67 (tt, *J* = 7.2, 1.2 Hz, 1H), 7.54 (tt, *J* = 7.6, 1.6 Hz, 2H). ^{13}C NMR (100 MHz, $CDCl_3$): δ 177.6, 136.6, 134.8, 133.4, 132.5, 129.8, 128.9, 125.1, 118.0, 114.2, 89.8, 89.5. IR: ν 3095, 3067, 2967, 2924, 2853, 2235, 2204, 1653, 1634, 1597, 1580, 1448, 1319, 1294, 1209, 1170, 698 cm^{-1}. Mass calculated $[M+H]^+$: 232.0762, HRMS $[M+H]^+$: 232.0755. MP: 150-153°C.

The following compounds were prepared using General Procedure C from known Weinreb amides. **3d**, **3f**, **1q**, and **3h-3i** are known compounds from literature.

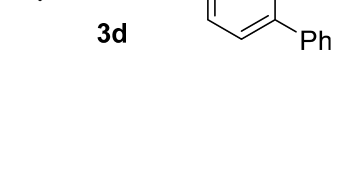

3d

3-([1,1'-biphenyl]-4-yl)-1-phenylprop-2-yn-1-one (3d)[4]
Isolated as a white solid (259 mg, 0.92 mmol, 76%). ^{1}H NMR (400 MHz, $CDCl_3$): δ 7.63-7.60 (m, 6H), 7.57-7.54 (m, 2H), 7.45 (t, *J* = 7.6 Hz, 2H), 7.38-7.34 (m, 4H). Mass calculated $[M+H)]^+$: 283.1123, HRMS $[M+H]^+$: 283.1112.

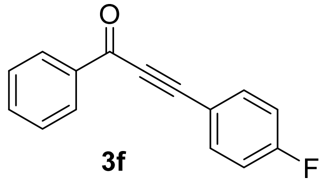

3f

3-(4-fluorophenyl)-1-phenylprop-2-yn-1-one (3f)[43]
Isolated as a white solid (95 mg, 0.42 mmol, 35%). ^{1}H NMR (400 MHz, $CDCl_3$): δ 8.21 (m, 2H), 7.68 (m, 3H), 7.53 (m, 2H), 7.11 (m, 2H). Mass calculated [M+H]$^+$: 225.0716, HRMS [M+H]$^+$: 225.0712.

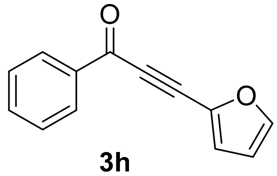

3h

3-(furan-2-yl)-1-phenylprop-2-yn-1-one (3h)[38]
Isolated as a white solid (55 mg, 0.28 mmol, 31%). ^{1}H NMR (400 MHz, $CDCl_3$) δ 8.19 (dt, *J* = 7.2, 1.6 Hz, 2H), 7.64 (tt, *J* = 7.6, 1.6 Hz, 1H), 7.58 (dd, *J* = 2.0, 0.8 Hz, 1H), 7.52 (td, *J* = 6.0, 1.6 Hz, 2H), 7.05 (d, *J* = 3.2 Hz, 1H), 6.53 (q, *J* = 2.0 Hz, 1H). Mass calculated [M+H]$^+$: 196.0603, HRMS [M+H]$^+$: 197.0600.

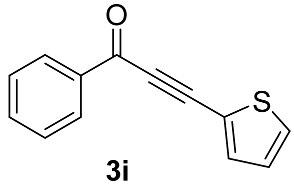

3i

1-phenyl-3-(thiophen-2-yl)prop-2-yn-1-one (3i)[37]
Isolated as a white solid (66 mg, 0.31 mmol, 26%). ^{1}H NMR (400 MHz, $CDCl_3$): δ 8.19 (dt, *J* = 5.2, 1.6 Hz, 2H), 7.64 (tt, *J* = 7.6, 1.6 Hz, 1H), 7.58 (dd, *J* = 2.0, 0.8 Hz, 1H), 7.52 (td, *J* = 6.0, 1.6 Hz, 2H), 7.05 (d, *J* = 3.6 Hz, 1H), 6.53 (q, *J* = 2.0 Hz, 1H). Mass calculated [M+H]$^+$: 213.0374, HRMS [M+H]$^+$: 213.0370.

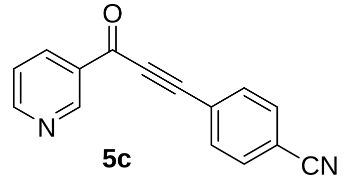

5c

4-(3-oxo-3-(pyridin-3-yl)prop-1-yn-1-yl)benzonitrile (5c)
Isolated as a white solid (97 mg, 0.42 mmol, 42%). ^{1}H NMR (400 MHz, $CDCl_3$): δ 9.45 (s, 1H), 8.87 (d, *J* = 3.6 Hz, 1H), 8.47 (dt, *J* = 8.0, 1.6 Hz, 1H), 8.01 (dt, *J* = 8.4, 2.0 Hz, 2H), 7.79 (dt, *J* = 8.8, 2.0 Hz, 2H), 7.53 (q, *J* = 3.2 Hz, 1H). ^{13}C NMR (100 MHz, $CDCl_3$): δ 176.1, 154.8, 151.6, 136.3, 133.6, 132.5, 124.5, 123.9, 117.9, 14.7, 91.2, 88.6. IR: ν 2963, 2926, 2862, 2197, 1679, 1639, 1569, 1302, 1209, 1094, 723 cm^{-1}. Mass calculated [M+H]$^+$: 233.0715, HRMS [M+H]$^+$: 233.0717. MP: 132-135°C.

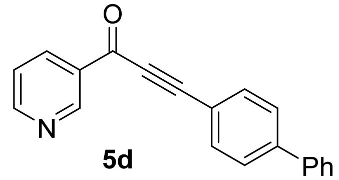

5d

3-([1,1'-biphenyl]-4-yl)-1-(pyridin-3-yl)prop-2-yn-1-one (5d)
Isolated as a white solid (160 mg, 0.57 mmol, 47%). ^{1}H NMR (400 MHz, $CDCl_3$): δ 9.47 (d, *J* = 1.6 Hz, 1H), 8.86 (dd, *J* = 5.2, 1.8 Hz, 1H), 8.46 (dt, *J* = 8.0, 2.0 Hz, 1H), 7.77 (dt, *J* = 8.4, 1.8 Hz, 2H), 7.68 (dt, *J* = 8.0, 2.0 Hz, 2H), 7.62 (dt, *J* = 8.0, 1.6 Hz, 2H), 7.51-7.46 (m, 3H), 7.41 (tt, *J* = 7.2, 1.4 Hz, 1H). ^{13}C NMR (100 MHz, $CDCl_3$): δ 176.4, 154.3, 151.5, 144.2, 139.7, 136.3, 133.9, 132.3, 129.1, 128.4, 127.5, 127.2, 123.7, 118.3, 94.9, 87.1. IR: ν 3039, 2927, 2845, 2186, 1652, 1601, 1590, 1477, 1408, 1326, 1307, 1210 cm^{-1}. Mass calculated [M^+]: 283.0997, HRMS [M^+]: 283.0997. MP: 118-120°C.

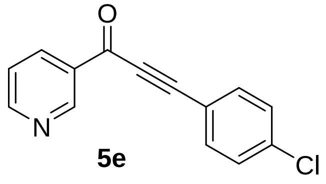

5e

3-(4-chlorophenyl)-1-(pyridin-3-yl)prop-2-yn-1-one (5e)

Isolated as a light yellow solid (62 mg, 0.26 mmol, 17%). ^{1}H NMR (400 MHz, $CDCl_3$): δ 9.42 (dd, J = 2.4, 0.8 Hz, 1H), 8.85 (dd, J = 4.8, 1.8Hz, 1H), 8.42 (dt, J = 8.4, 2.0 Hz, 1H), 7.63 (dt, J = 8.8, 2.2 Hz, 2H), 7.49 (qd, J = 4.2, 0.8 Hz, 1H), 7.43 (dt, J = 6.4, 2.0 Hz, 2H). ^{13}C NMR (100 MHz, $CDCl_3$): δ 176.3, 154.4, 151.5, 137.9, 136.4, 134.6, 132.3, 129.5, 123.8, 118.1, 110.2, 93.4, 87.1. IR: ν 3361, 2210, 1636, 1582, 1485, 1423, 1404, 1303 cm^{-1}. Mass calculated [M+H]$^{+}$: 242.0367, HRMS [M+H]$^{+}$: 242.0364. MP: 114-116°C.

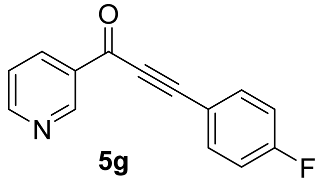

5g

3-(4-fluorophenyl)-1-(pyridin-3-yl)prop-2-yn-1-one (5g)

Isolated as a light yellow solid (115 mg, 0.51 mmol, 34%). ^{1}H NMR (400 MHz, $CDCl_3$): δ 9.44 (d, J = 1.6 Hz, 1H), 8.85 (dd, J = 4.8, 1.4 Hz, 1H), 8.44 (dt, J = 8.0, 1.8 Hz, 1H), 7.72 (tt, J = 6.8, 2.6 Hz, 2H), 7.50 (qd, J = 3.8, 0.8 Hz, 1H), 7.15 (tt, J = 8.4, 2.2 Hz, 2H). ^{13}C NMR (100 MHz, $CDCl_3$): δ 176.2, 154.1, 151.3, 136.6, 135.8, 135.8, 132.4, 123.9, 116.7, 116.5, 93.9, 86.3. ^{19}F NMR (377 MHz): δ -105.0 (m). IR: ν 3326, 2930, 2845, 2198, 1632, 1601, 1501, 1400, 1299, 1198 cm^{-1}. Mass calculated [M^{+}]: 225.0590, HRMS [M^{+}]: 225.0585. MP: 114-116°C.

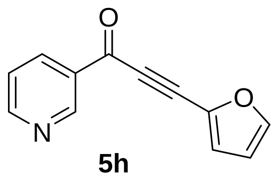

5h

3-(furan-2-yl)-1-(pyridin-3-yl)prop-2-yn-1-one (5h)

Isolated as a light brown amorphous solid (53 mg, 0.27 mmol, 29%). ^{1}H NMR (400 MHz, $CDCl_3$): δ 9.40 (dd, J = 2.4, 0.8 Hz, 1H), 8.84 (dd, J = 4.8, 2.0 Hz, 1H), 8.41 (dq, J = 8.0, 1.6 Hz, 1H), 7.61 (dd, J = 2.0, 0.8 Hz, 1H), 7.48 (qd, J = 4.0, 0.8 Hz, 1H), 7.10 (dd, J = 3.6, 0.8 Hz, 1H), 6.55 (q, J = 1.8 Hz, 1H). ^{13}C NMR (100 MHz, $CDCl_3$): δ 175.8, 154.4, 151.3, 147.3, 136.3, 134.6, 131.9, 123.7, 122.7, 112.3, 92.2, 85.0. IR: ν 2966, 2926, 2848, 2185, 1746, 1698, 1656, 1558, 1294, 1235, 1088 cm^{-1}. Mass calculated [M+H]$^{+}$: 198.0555, HRMS [M+H]$^{+}$: 198.0551.

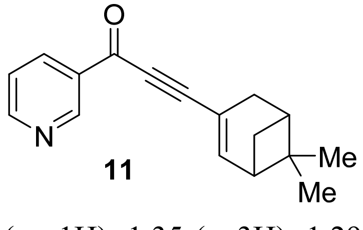

11

3-(6,6-dimethylbicyclo[3.1.1]hept-2-en-3-yl)-1-(pyridin-3-yl)prop-2-yn-1-one (11)

Isolated as a yellow oil (281 mg, 1.12 mmol, 60%). ^{1}H NMR (400 MHz, $CDCl_3$): δ 9.33 (dd, J = 2.4, 0.8 Hz, 1H), 8.80 (dd, J = 5.2, 2.0 Hz, 1H), 8.35 (dt, J = 8.0, 2.0 Hz, 1H), 7.43 (ddd, J = 8.0, 4.8, 0.8 Hz, 1H), 6.58 – 6.55 (m, 1H), 2.55 – 2.41 (m, 4H), 2.20 – 2.16 (m, 1H), 1.35 (s, 3H), 1.29 (d, J = 8.8 Hz, 1H), 0.91 (s, 3H). ^{13}C NMR (100 MHz, $CDCl_3$): δ 176.5, 153.9, 151.5, 141.0, 136.1, 128.2, 123.4, 95.7, 87.0, 46.4, 38.1, 31.1, 25.8, 21.1. IR: ν 2938, 2179, 1640, 1581, 1419, 1326, 1280 cm^{-1}. Mass calculated [M+H]$^{+}$: 252.1388, HRMS [M+H]$^{+}$: 252.1391.

Rh-Catalyzed Decarbonylation of Ynone Compounds: General Procedure

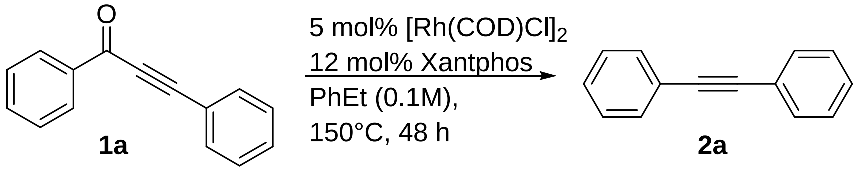

To an oven-dried test tube (18 X 150 mm) equipped with a magnetic stir bar was added monoynone (41.2 mg, 0.200 mmol), xantphos (13.9 mg, 0.024 mmol), and $[Rh(COD)Cl]_2$ (5.0 mg, 0.010 mmol). The vessel was capped with a septum and transferred into a glovebox (through standard glovebox procedure), where ethyl benzene (2.0 mL, 0.1 M) was added. The vessel was removed from the glovebox and placed under a positive flow of an inert Ar atmosphere. The reaction was heated at 90 ^{o}C for 30 min and then refluxed (150 ^{o}C) for 48 h (or until the reaction was completed by TLC). The reaction was concentrated under reduced pressure to ¼ the volume and directly loaded onto a silica gel column. The vessel was rinsed with a small portion of toluene and loaded onto the column after which the column was eluted with hexanes to obtain the desired product as a white solid in 91% yield (32.4 mg).

1,2-diphenylethyne (2a)[16]

^{1}H NMR (400 MHz, $CDCl_3$): δ 7.55-7.53 (m, 2H), 7.38-7.31 (m, 3H). ^{13}C NMR (100 MHz, $CDCl_3$): δ 131.6, 128.3, 128.2, 123.2, 89.3. IR: ν 3062, 1601, 1489, 1439 cm^{-1}. Mass calculated [M+H]$^{+}$: 179.0861, HRMS [M+H]$^{+}$: 179.0856. MP: 57-59°C.

The following compounds were prepared according to the above described procedure on a 0.2 mmol scale. **2a-2o**, **4a-4o**, **6a-6c**, **6e-6g**, **6j**, **14**, and **16** are known compounds from literature.

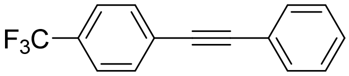

2b

1-(phenylethynyl)-4-(trifluoromethyl)benzene (2b)[17]

Isolated as a white solid (25.6 mg, 0.10 mmol, 52%). IR: ν 3081, 2926, 2862, 2218, 1963, 1929, 1887, 1607, 1563, 1488, 1444 cm^{-1}. ^{1}H NMR (400 MHz, $CDCl_3$): δ 7.62 (q, J = 5.6 Hz, 4H), 7.56-7.53 (m, 2H), 7.39-7.36 (m, 3H). ^{19}F NMR (377 MHz): δ -62.8 (t, J = 3.0 Hz, 3F). ^{13}C NMR (100 MHz, $CDCl_3$): δ 131.9, 131.8, 128.9, 128.6, 127.3, 125.44, 125.40, 125.3, 122.7, 110.2, 91.9, 88.1. Mass calculated [M^{+}]: 246.0656, HRMS [M^{+}]: 246.0659. MP: 87-88°C.

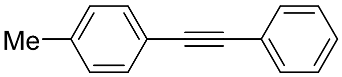

2c, 4b

1-methyl-4-(phenylethynyl)benzene (2c, 4b)[16]

Isolated as a white solid (25.7 mg, 0.13 mmol, 67% (90% BRSM)), (25.3 mg, 0.13 mmol, 66% (81% BRSM)). ^{1}H NMR (400 MHz, $CDCl_3$): δ 7.53 (dt, J = 6.0, 2.0 Hz, 2H), 7.43 (dd, J = 6.4, 2.0 Hz, 2H), 7.37-7.32 (m, 3H), 7.16 (d, J = 8.0 Hz, 2H), 2.38 (s, 3H). ^{13}C NMR (100 MHz, $CDCl_3$): δ 138.5, 131.7, 131.6, 129.3, 128.5, 128.2, 123.6, 120.3. IR: ν 3431, 3090, 3045, 3023, 2931, 2843, 2215, 1943, 1913, 1879, 1596, 1513, 1449 cm^{-1}. Mass calculated [M^{+}]: 192.0939, HRMS [M^{+}]: 192.0938. MP: 53-55°C.

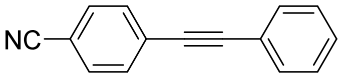

2d, 4c

4-(phenylethynyl)benzonitrile (2d, 4c)[18]

Isolated as a white solid (14.6, 0.07 mmol, 36% (50% BRSM)), (28.8 mg, 0.14 mmol, 71%). ^{1}H NMR (400 MHz, $CDCl_3$): δ 7.65-7.59 (m, 4H), 7.56-7.53 (m, 2H), 7.39-7.36 (m, 3H). ^{13}C NMR (100 MHz, $CDCl_3$): δ 132.2, 131.9, 129.3, 128.6, 128.4, 122.3, 118.7, 113.5, 111.6, 93.9, 87.8. IR: ν 3440, 3090, 3049, 3021, 2973, 2928, 2848, 2257, 2229, 2216, 1929, 1610, 1500, 1437, 1410, 1275 cm^{-1}. Mass calculated $[M+H]^+$: 204.0813, HRMS $[M+H]^+$: 204.0812. MP: 108-110°C.

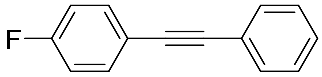

2e, 4f

1-fluoro-4-(phenylethynyl)benzene (2e, 4f)[17]

Isolated as a white solid (26.3 mg, 0.13 mmol, 67% (87% BRSM)), (11.8, 0.06 mmol, 30% (48% BRSM)). ^{1}H NMR (400 MHz, $CDCl_3$): δ 7.55-7.49 (m, 4H), 7.38-7.33 (m, 3H), 7.08-7.02 (m, 2H). ^{19}F NMR (377 MHz): δ -110.9-(-111.1) (m, 1F). ^{13}C NMR (100 MHz, $CDCl_3$): δ 163.9, 161.4, 133.7, 133.6, 131.7, 128.5, 128.5, 123.2, 119.5, 119.4, 115.9, 115.7, 89.2, 89.1, 88.4. IR: ν 3431, 2954, 2918, 2843, 1877, 1649, 1596, 1505, 1438 cm^{-1}. Mass calculated $[M^+]$: 196.0688, HRMS $[M^+]$: 196.0688. MP: 92-94°C.

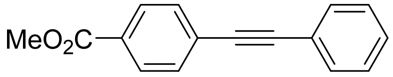

2f

Methyl 4-(phenylethynyl)benzoate (2f)[19]

Isolated as a white solid (34.9 mg, 0.15 mmol, 74% (92% BRSM)). ^{1}H NMR (400 MHz, $CDCl_3$): δ 8.02 (dt, J = 8.4, 2.0 Hz, 2H), 7.60-7.54 (m, 4H), 7.38-7.36 (m, 3H), 3.93 (s, 3H). ^{13}C NMR (100 MHz, $CDCl_3$): δ 166.7, 131.9, 131.6, 129.64, 129.57, 128.9, 128.6, 128.1, 122.8, 92.5, 88.8, 52.4. IR: ν 3416, 3084, 3056, 3035, 3007, 2949, 2852, 2222, 1721, 1614, 1444, 1403, 1282, 1209, 1171, 1109 cm^{-1}. Mass calculated $[M+H]^+$: 237.0916, HRMS $[M+H]^+$: 237.0914. MP: 125-127°C.

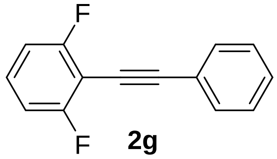

2g

1,3-difluoro-2-(phenylethynyl)benzene (2g)[20]

Isolated as a light yellow solid (24.8 mg, 0.12 mmol, 58% (83% BRSM)). ^{1}H NMR (400 MHz, $CDCl_3$): δ 7.63 (dt, J = 7.2, 1.6 Hz, 2H), 7.50-7.38 (m, 4H), 7.03-6.97 (m, 2H). ^{13}C NMR (100 MHz, $CDCl_3$): δ 131.9, 129.8, 129.7, 129.6, 129.1, 128.5, 122.7, 111.5, 111.4, 111.3, 111.2. ^{19}F NMR (377 MHz): δ -107.5 (m, 2F). IR: ν 3440, 3077, 3035, 2925, 2845, 2229, 1655, 1635, 1579, 1555, 1493, 1472 cm^{-1}. Mass calculated $[M^+]$: 214.0594, HRMS $[M^+]$: 214.0592. MP: 121-123 °C.

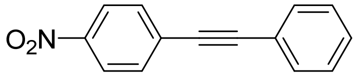

2h

1-nitro-4-(phenylethynyl)benzene (2h)[21]

Isolated as a white solid (34.4 mg, 0.15 mmol, 77%). ^{1}H NMR (400 MHz, $CDCl_3$): δ 8.22 (dt, J = 8.8, 2.0 Hz, 2H), 7.66 (dt, J = 8.8, 2.0 Hz, 2H), 7.58-7.55 (m, 2H), 7.42-7.38 (m, 3H). ^{13}C NMR (100 MHz, $CDCl_3$): δ 146.9, 132.3, 131.8, 130.2, 129.3, 128.5, 123.6, 122.1, 94.7, 87.5. IR: ν 2546, 2837, 2210, 1605, 1524, 1512, 1357, 1070 cm^{-1}. Mass calculated $[M+H]^+$: 224.0712, HRMS $[M+H]^+$: 224.0713. MP: 117-120°C.

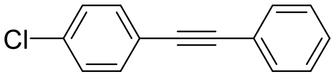

2i

1-chloro-4-(phenylethynyl)benzene (2i)[22]

Isolated as a white solid (15.3 mg, 0.07 mmol, 36% (43% BRSM)). ^{1}H NMR (400 MHz, $CDCl_3$): δ 7.54-7.51 (m, 2H), 7.46 (dt, J = 8.8, 2.0 Hz, 2H), 7.37-7.35 (m, 3H), 7.32 (dt, J = 8.8, 2.0 Hz, 2H). ^{13}C NMR (100 MHz, $CDCl_3$): δ 134.2, 132.8, 131.6, 128.7, 128.5, 128.4, 122.9, 121.8, 90.3, 88.2. IR: ν 2911, 2849, 1497, 1442, 1400, 1094 cm^{-1}. Mass calculated $[M+H]^+$: 213.0468, HRMS $[M+H]^+$: 213.0471. MP: 79-82°C.

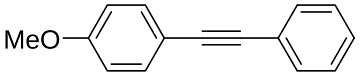

2j, 4a

1-methoxy-4-(phenylethynyl)benzene (2j, 4a)[23]

Isolated as a white solid (15.4 mg, 0.07 mmol, 37% (77% BRSM)), (25.0 mg, 0.12 mmol, 60% (83% BRSM)). ^{1}H NMR (400 MHz, $CDCl_3$): δ 7.53-7.46 (m, 4H), 7.36-7.31 (m, 3H), 6.88 (dd, J = 6.8, 2.0 Hz, 2H), 3.83 (s, 3H). ^{13}C NMR (100 MHz, $CDCl_3$): δ 159.6, 133.0, 131.4, 128.3, 127.9, 123.6, 115.4, 114.0, 89.3, 88.0, 55.3. IR: ν 2922, 2845, 1493, 1439, 1396, 1094 cm^{-1}. Mass calculated $[M^+]$: 208.0888, HRMS $[M^+]$: 208.0890. MP: 57-59°C.

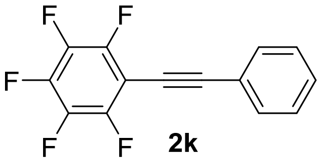

2k

1,2,3,4,5-pentafluoro-6-(phenylethynyl)benzene (2k)[24]

Isolated as a cream solid (29.5 mg, 0.11 mmol, 55% (69% BRSM)). ^{1}H NMR (400 MHz, $CDCl_3$): δ 7.58-7.56 (m, 2H), 7.41-7.35 (m, 3H). ^{13}C NMR (100 MHz, $CDCl_3$): δ 148.4-148.3 (m), 146.0-145.8 (m), 142.8-142.5 (m), 140.1-140.0 (m), 138.9-138.7 (m), 136.6-136.2 (m), 131.9, 129.6, 128.5, 121.5, 101.6-101.5 (m), 100.3-100.2 (m), 73.1-73.0 (m). ^{19}F NMR (377 MHz, $CDCl_3$): δ -136.0 (m), -152.9 (t, J = 20.7 Hz), -161.9-(-162.3) (m). IR: ν 3085, 3058, 2923, 2845, 2667, 2620, 2469, 2434, 2051, 1969, 1892, 1584, 1439 cm^{-1}. Mass calculated $[M+H]^+$: 269.0390, HRMS $[M+H]^+$: 269.0388. MP: 94-96°C.

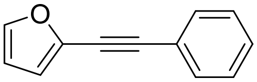

2l, 4h

2-(phenylethynyl)furan (2l,4h)[25]

Isolated as a light red oil (14.8 mg, 0.09 mmol, 44% (68% BRSM)) (12.7 mg, 0.08 mmol, 38%). ^{1}H NMR (400 MHz, $CDCl_3$): δ 7.55-7.50 (m, 2H), 7.43 (dd, J = 1.8, 0.7 Hz, 1H), 7.36-7.34 (m, 3H), 6.66 (dd, J = 3.4, 0.7 Hz, 1H), 6.43 (q, J = 1.5 Hz, 1H). ^{13}C NMR (100 MHz, $CDCl_3$): δ 143.8, 137.3, 131.6, 128.8, 128.5, 122.4, 115.4, 111.2, 93.4, 79.5. IR: ν 2956, 2921, 2661, 2187, 1743, 1656, 1622, 1558, 1516, 1451, 1282.4, 1218, 1077 cm^{-1}. Mass calculated $[M^+]$: 168.0575, HRMS $[M^+]$: 168.0575.

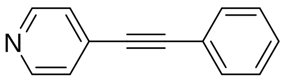

2m

4-(phenylethynyl)pyridine (2m)[26]

Isolated as a white solid (24.4 mg, 0.14 mmol, 68%). ^{1}H NMR (400 MHz, $CDCl_3$): δ 8.60 (dd, J = 4.5, 1.6, 2H), 7.60-7.51 (m, 2H), 7.44-7.31 (m, 5H). ^{13}C NMR (100 MHz, $CDCl_3$): δ 149.7, 131.9, 131.5, 129.2, 128.5, 125.5, 122.1, 93.9, 86.6. IR: ν 2917, 2848, 1588, 1535 cm^{-1}. Mass calculated $[M+H]^+$: 180.0808, HRMS $[M+H]^+$: 180.0806. MP: 89-91°C.

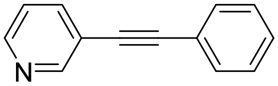

2n

3-(phenylethynyl)pyridine (2n)[19]

Isolated as a white solid (32.6 mg, 0.18 mmol, 91%). ^{1}H NMR (400 MHz, $CDCl_3$): δ 8.77 (d, *J* = 1.6 Hz, 1H), 8.55 (dd, *J* = 5.2, 1.6 Hz, 1H), 7.81 (dt, *J* = 7.6, 2.0 Hz, 1H), 7.56 – 7.54 (m, 2H), 7.39 – 7.35 (m 3H), 7.28 (ddd, *J* = 8.0, 4.8, 0.4 Hz, 1H). ^{13}C NMR (100 MHz, $CDCl_3$): δ 152.2, 148.5, 138.4, 131.7, 128.8, 128.4, 123.0, 122.5, 120.4, 92.6, 85.9. IR: ν 3062, 2912, 2845, 2215, 1952, 1593, 1555, 1494, 1405 cm^{-1}. Mass calculated $[M+H]^+$: 180.0808, HRMS $[M+H]^+$: 180.0810. MP: 50-52°C.

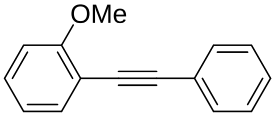

2o

1-methoxy-2-(phenylethynyl)benzene (2o)[27]

Isolated as a light yellow oil (6.7 mg, 0.03 mmol, 16% (21% BRSM)). ^{1}H NMR (400 MHz, $CDCl_3$): δ 7.56 (dd, *J* = 7.2, 1.8 Hz, 2H), 7.50 (dd, *J* = 8.0, 1.6 Hz, 1H), 7.36-7.28 (m, 4H), 6.93 (q, *J* = 8.4 Hz, 2H). ^{13}C NMR (100 MHz, $CDCl_3$): δ 133.7, 131.8, 129.9, 128.3, 128.2, 123.7, 121.5, 120.6, 112.6, 110.8, 93.6, 85.8, 56.0. IR: ν 3064, 2957, 2929, 2851, 1662, 1594, 1496, 1459, 1437, 1277, 1246, 1105, 1027 cm^{-1}. Mass calculated $[M^+]$: 208.0888, HRMS $[M^+]$: 208.0886.

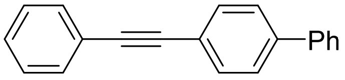

4d

4-(phenylethynyl)-1,1'-biphenyl (4d)[16]

Isolated as a cream solid (37.1 mg, 0.15 mmol, 73% (86% BRSM)). ^{1}H NMR (400 MHz, $CDCl_3$): δ 7.63-7.58 (m, 6H), 7.55 (dd, *J* = 7.9, 1.7 Hz, 2H), 7.46 (t, *J* = 7.5 Hz, 2H), 7.38-7.35 (m, 4H). ^{13}C NMR (100 MHz, $CDCl_3$): δ 141.1, 140.5, 132.2, 131.7, 129.0, 128.5, 128.4, 127.8, 127.2, 123.4, 122.3, 90.2, 89.4. IR: ν 3464, 3080, 3059, 2966, 2931, 2845, 1666, 1451, 1403, 1171, 1067, 853, 760 cm^{-1}. Mass calculated $[M+H]^+$: 255.1174, HRMS $[M+H]^+$: 255.1170. MP: 155-158°C.

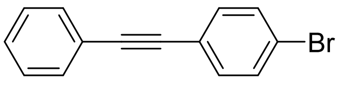

4e

1-bromo-4-(phenylethynyl)benzene (4e)[23]

Isolated as a clear oil (6.2 mg, 0.02 mmol, 12% (23% BRSM)). ^{1}H NMR (400 MHz, $CDCl_3$): δ 7.56-7.50 (m, 4H), 7.48-7.34 (m, 5H). ^{13}C NMR (100 MHz, $CDCl_3$): δ 133.1, 132.6, 131.8, 131.7, 128.6, 128.6, 128.5, 123.0, 122.6, 122.4, 90.6, 88.4. IR: ν 2960, 2918, 2856, 1954, 1842, 1726, 1656, 1558, 1457, 1066. 790 cm^{-1}. Mass calculated $[M^+]$: 255.9888, HRMS $[M^+]$: 255.9890.

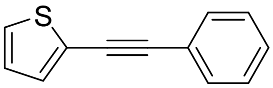

4i

2-(phenylethynyl)thiophene (4i)[47]

Isolated as a cream solid (8.1 mg, 0.04 mmol, 22% (29% BRSM)). ^{1}H NMR (400 MHz, $CDCl_3$) δ 7.53-7.51 (m, 2H), 7.37-7.34 (m, 3H), 7.30-7.28 (m, 2H), 7.01 (dd, *J* = 3.6, 1.6 Hz, 1H). ^{13}C NMR (100 MHz, $CDCl_3$): δ 132.4, 131.9, 128.9, 128.8, 127.7, 127.6, 123.8, 123.4, 93.5, 83.1. IR: ν 3457, 3115, 3070, 3056, 2935, 2842, 2205, 1655, 1586, 1479, 1444, 1420, 1209 cm^{-1}. Mass calculated $[M^+]$: 184.0347, HRMS $[M^+]$: 184.0351.

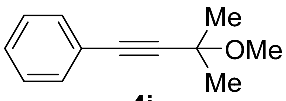

4j

(3-methoxy-3-methylbut-1-yn-1-yl)benzene (4j)[48]
Isolated as a clear oil (7.3 mg, 0.04 mmol, 40% (41% BRSM)). ^{1}H NMR (400 MHz, $CDCl_3$) δ 8.11 (d, *J* = 8.3 Hz, 1H), 8.05 (d, *J* = 8.2 Hz, 1H), 7.95 (d, *J* = 8.5 Hz, 1H), 7.69-7.64 (m, 2H), 7.55-7.51 (m, 1H), 7.44-7.41 (m, 1H), 7.40-7.34 (m, 3H), 7.26 (q, *J* = 7.0 Hz, 1H), 7.12 (ddd, *J* = 8.2, 6.9, 1.3 Hz, 1H), 6.80 (dd, *J* = 8.3, 0.8 Hz, 1H). ^{13}C NMR (100 MHz, $CDCl_3$): δ 132.1, 128.7, 128.6, 123.3, 91.4, 84.5, 71.4, 52.2, 28.8. IR: ν 3471, 3087, 3056, 2994, 2935, 2835, 2236, 1949, 1600, 1493, 1462, 1441, 1386, 1361, 1285, 1168 cm^{-1}. Mass calculated $[M+H]^+$: 175.1123, HRMS $[M+H)]^+$: 175.1123. Product is slightly volatile.

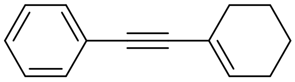

4o

(Cyclohex-1-en-1-ylethynyl)benzene (4o)[49]
Mass calculated $[M^+]$: 182.1096, HRMS $[M^+]$: 182.1093. Unable to isolate from mesitylene solvent, but 1H NMR and HRMS data matches literature reports.[50]

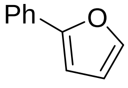

4p'

2-phenylfuran (4p')[50]
^{1}H NMR (400 MHz, $CDCl_3$): δ 7.68 (dt, *J* = 2.9, 1.7 Hz, 2H), 7.48 (dd, *J* = 4.4, 3.9 Hz, 1H), 7.42-7.36 (m, 2H), 7.29-7.23 (m, 1H), 6.66 (d, *J* = 3.4 Hz, 1H), 6.48 (dd, *J* = 3.4, 1.8 Hz, 1H). Unable to isolate from mesitylene solvent, but GC/MS, IR, 1H NMR and 13C NMR data matches literature reports.[50] See **Scheme S1** below.

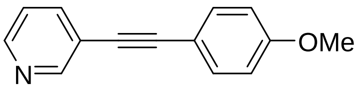

6a

3-((4-methoxyphenyl)ethynyl)pyridine (6a)[28]
Isolated as a white solid (40.1 mg, 0.19 mmol, 96%). ^{1}H NMR (400 MHz, $CDCl_3$): δ 8.75 (s, 1H), 8.52 (d, *J* = 3.6 Hz, 1H), 7.77 (dt, *J* = 8.0, 2.0 Hz, 1H), 7.48 (dt, *J* = 9.2, 2.6 Hz, 2H), 7.27 (qd, *J* = 3.8, 0.4 Hz, 1H), 6.89 (dt, *J* = 8.8, 2.4 Hz, 2H), 3.83 (s, 3H). ^{13}C NMR (100 MHz, $CDCl_3$): δ 160.1, 152.1, 148.2, 138.4, 133.3, 123.2, 121.0, 114.7, 114.2, 92.9, 84.8, 55.4. IR: ν 3074, 3039, 2996, 2965, 2927, 2834, 2217, 1605, 1559, 1512, 1466, 1404 cm^{-1}. Mass calculated $[M+H]^+$: 210.0913, HRMS $[M+H]^+$: 210.0911. MP: 46-47 °C.

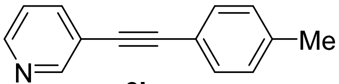

6b

3-(p-tolylethynyl)pyridine (6b)[29]
Isolated as a white solid (36.7 mg, 0.19 mmol, 95%). ^{1}H NMR (400 MHz, $CDCl_3$): δ 8.76 (s, 1H), 8.54 (d, *J* = 4.0 Hz, 1H), 7.79 (dt, *J* = 8.3, 1.8 Hz, 1H), 7.43 (dt, *J* = 8.0, 1.6 Hz, 2H), 7.28 (t, *J* = 3.0 Hz, 1H), 7.17 (dt, *J* = 7.8, 0.6 Hz, 2H), 2.38 (s, 3H). ^{13}C NMR (100 MHz, $CDCl_3$): δ 152.2, 148.4, 139.2, 138.6, 131.7, 129.3, 123.2, 120.9, 119.5, 93.1, 85.4, 21.7. IR: ν 3082, 3051, 2911, 2221, 1927, 1671, 1586, 1562, 1508, 1477, 1419 cm^{-1}. Mass calculated $[M+H]^+$: 194.0964, HRMS $[M+H]^+$: 194.0965. MP: 73-75 °C.

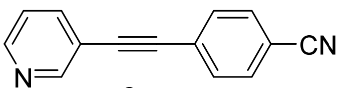

6c

4-(pyridin-3-ylethynyl)benzonitrile (6c)[30]

Isolated as an amorphous white solid (14.7 mg, 0.07 mmol, 36% (50% BRSM)). ^{1}H NMR (400 MHz, $CDCl_3$): δ 8.80 (s, 1H), 8.61 (d, *J* = 4.8 Hz, 1H), 7.87 (d, *J* = 8.0 Hz, 1H), 7.69-7.62 (m, 4H), 7.36 (q, *J* = 4.0 Hz, 1H). ^{13}C NMR (100 MHz, $CDCl_3$): δ 152.1, 149.0, 139.2, 132.4, 132.3, 127.4, 123.5, 119.9, 118.5, 112.4, 91.2, 89.9. IR: ν 2963, 2929, 2859, 2235, 1665, 1201, 1094, 844, 550 cm^{-1}. Mass calculated $[M+H]^+$: 205.0766, HRMS $[M+H]^+$: 205.0767.

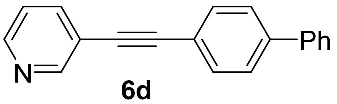

6d

3-([1,1'-biphenyl]-4-ylethynyl)pyridine (6d)

Isolated as a white solid (26.0 mg, 0.10 mmol, 51%). ^{1}H NMR (400 MHz, $CDCl_3$): δ 8.80 (d, *J* = 1.2 Hz, 1H), 8.56 (dd, *J* = 5.2, 1.6 Hz, 1H), 7.83 (dt, *J* = 7.6, 2.0 Hz, 1H), 7.64-7.60 (m, 6H), 7.46 (tt, *J* = 8.4, 0.8 Hz, 2H), 7.38 (tt, *J* = 7.6, 1.6 Hz, 1H), 7.30 (qd, *J* = 4.8, 0.8 Hz, 1H). ^{13}C NMR (100 MHz, $CDCl_3$): δ 152.3, 148.6, 141.7, 140.3, 138.6, 132.3, 129.0, 127.9, 127.2, 127.1, 123.2, 121.5, 120.7, 92.8, 86.7. IR: ν 3069, 3053, 3028, 2974, 2926, 2861, 2218, 1920, 1558, 1493, 1406, 1260, 1131, 1021, 844, 807, 756, 737, 701, 684 cm^{-1}. Mass calculated $[M^+]$: 255.1048, HRMS $[M^+]$: 255.1052. MP: 121-123 °C.

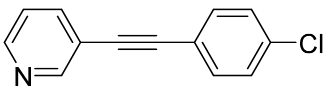

6e

3-((4-chlorophenyl)ethynyl)pyridine (6e)[31]

Isolated as a white solid (26.1 mg, 0.12 mmol, 61%). ^{1}H NMR (400 MHz, $CDCl_3$): δ 8.75 (d, *J* = 1.5 Hz, 1H), 8.55 (dd, *J* = 4.9, 1.6 Hz, 1H), 7.79 (dt, *J* = 8.0, 1.7 Hz, 1H), 7.47 (dq, *J* = 8.7, 2.0 Hz, 2H), 7.34 (dt, *J* = 8.8, 2.1 Hz, 2H), 7.28 (qd, *J* = 3.9, 0.8 Hz, 1H). ^{13}C NMR (100 MHz, $CDCl_3$): δ 152.1, 148.7, 138.4, 134.9, 132.9, 128.8, 123.0, 120.9, 120.1, 91.5, 86.8. IR: ν 3454, 3090, 3062, 3035, 2931, 2842, 2219, 1901, 1572, 1493, 1410, 1085, 1016, 822, 812, 704 cm^{-1}. Mass calculated $[M+H]^+$: 214.0418, HRMS $[M+H]^+$: 214.0420. MP: 88-91°C.

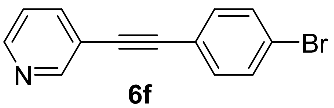

6f

3-((4-bromophenyl)ethynyl)pyridine (6f)[32]

Isolated as a light brown solid (28.9 mg, 0.11 mmol, 56% (76% BRSM)). ^{1}H NMR (400 MHz, $CDCl_3$): δ 8.76 (s, 1H), 8.56 (dd, *J* = 4.4, 1.2 Hz, 1H), 7.80 (dq, *J* = 8.0, 2.0 Hz, 1H), 7.51 (dt, *J* = 8.8, 2.0 Hz, 2H), 7.41 (dt, *J* = 8.8, 2.0 Hz, 2H), 7.29 (qd, *J* = 4.0, 0.8 Hz, 1H). ^{13}C NMR (100 MHz, $CDCl_3$): δ 152.0, 148.5, 138.4, 133.2, 131.8, 128.7, 128.5, 123.3, 121.5, 91.8, 87.2. IR: ν 3085, 3054, 3027, 2923, 2857, 2210, 1907, 1648, 1582, 1559, 1485 cm^{-1}. Mass calculated $[M+H]^+$: 257.9918, HRMS $[M+H]^+$: 257.9917. MP: 68-70 °C.

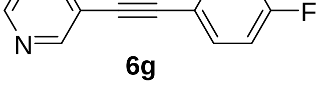

6g

3-((4-fluorophenyl)ethynyl)pyridine (6g)[33]

Isolated as a white solid (25.6 mg, 0.13 mmol, 65%). ^{1}H NMR (400 MHz, $CDCl_3$): δ 8.76 (s, 1H), 8.55 (d, *J* = 4.0 Hz, 1H), 7.81 (dt, *J* = 8.0, 2.0 Hz, 1H), 7.55-7.50 (m, 2H), 7.30 (qd, *J* = 3.2, 0.8 Hz, 1H), 7.06 (tt, *J* = 6.4, 2.0 Hz, 2H). ^{13}C NMR (100 MHz, $CDCl_3$): δ 164.2, 161.7, 152.1, 148.5, 138.7, 133.8, 133.7, 123.3, 120.5, 118.7, 116.1, 115.8, 91.8, 85.7. IR: ν 3101, 3070, 3043, 2919, 2845, 2210, 1609, 1574, 1551,

1504, 1404 cm^{-1}. ^{19}F NMR (377 MHz): δ -109.9 (m). Mass calculated $[M+H]^+$: 198.0714, HRMS $[M+H]^+$: 198.0710. MP: 77-78 °C.

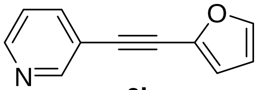

6h

2-(phenylethynyl)thiophene (6h)

Isolated as a clear amorphous solid (12.8 mg, 0.08 mmol, 38%). ^1H NMR (400 MHz, $CDCl_3$): δ 8.75 (dd, *J* = 2.0, 0.8 Hz, 1H), 8.55 (dd, *J* = 4.8, 1.6 Hz, 1H), 7.80 (dt, *J* = 8.0, 2.0 Hz, 1H), 7.45 (dd, *J* = 2.0, 0.8 Hz, 1H), 7.28 (qd, *J* = 4.8, 0.8 Hz, 1H), 6.71 (dd, *J* = 3.2, 0.6 Hz, 1H), 6.45 (q, *J* = 1.6 Hz, 1H). ^{13}C NMR (100 MHz, $CDCl_3$): δ 151.9, 148.9, 144.3, 138.5, 136.6, 123.3, 119.8, 116.3, 111.3, 90.1, 82.9. IR: ν 3154, 3112, 3039, 2966, 2926, 2848, 2215, 1569, 1485, 1468, 1403, 1302, 1215, 1181, 1013, 937, 796, 746 cm^{-1}. Mass calculated $[M+H]^+$: 170.0606, HRMS $[M+H]^+$: 170.0601.

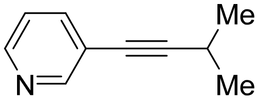

6i

3-(3-methylbut-1-yn-1-yl)pyridine (6i)

Isolated as a clear oil (7.6 mg, 0.05 mmol, 26%). ^1H NMR (400 MHz, $CDCl_3$): δ 8.60 (d, *J* = 1.2 Hz, 1H), 8.46 (dd, *J* = 4.4, 1.6 Hz, 1H), 7.64 (dt, *J* = 8.0, 1.8 Hz, 1H), 7.19 (qd, *J* = 4.0, 0.6 Hz, 1H), 2.78 (sep, *J* = 2.8 Hz, 1H), 1.25 (d, *J* = 6.8 Hz, 6H). ^{13}C NMR (100 MHz, $CDCl_3$): δ 152.4, 147.9, 138.6, 122.9, 121.2, 99.4, 76.6, 22.9, 21.3. IR: ν 3436, 3059, 2973, 2931, 2855, 1690, 1455, 1434, 1410, 1368, 1227, 1185, 749, 694 cm^{-1}. Mass calculated $[M+H]^+$: 146.0964, HRMS $[M+H]^+$: 146.0965.

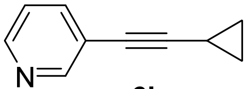

6j

3-(cyclopropylethynyl)pyridine (6j)[34]

Isolated as a clear oil (12.9 mg, 0.09 mmol, 45%). ^1H NMR (400 MHz, $CDCl_3$): δ 8.59 (d, *J* = 1.6 Hz, 1H), 8.45 (dd, *J* = 5.0, 1.4 Hz, 1H), 7.64 (dt, *J* = 8.0, 2.0 Hz, 1H), 7.18 (qd, *J* = 4.8, 0.8 Hz, 1H), 1.49-1.42 (m, 1H), 0.92-0.87 (m, 2H), 0.86-0.80 (m, 2H). ^{13}C NMR (100 MHz, $CDCl_3$): δ 152.4, 147.8, 138.7, 123.1, 121.3, 97.3, 72.6, 8.9, 0.3. IR: ν 2957, 2926, 2848, 2373, 2345, 1653, 1561, 1457, 1066, 1159 cm^{-1}. Mass calculated $[M+H]^+$: 144.0808, HRMS $[M+H]^+$: 144.0805.

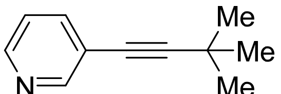

6k

3-(3,3-dimethylbut-1-yn-1-yl)pyridine (6k)

Isolated as a clear oil (13.1 mg, 0.08 mmol, 41% (91% BRSM)). ^1H NMR (400 MHz, $CDCl_3$): δ 8.59 (d, *J* = 2.0 Hz, 1H), 8.45 (dd, *J* = 4.6, 1.4 Hz, 1H), 7.64 (dt, *J* = 8.0, 1.8 Hz, 1H), 7.18 (q, *J* = 4.0 Hz, 1H), 1.31 (s, 9H). ^{13}C NMR (100 MHz, $CDCl_3$): δ 152.4, 147.9, 138.5, 122.9, 121.3, 102.2, 75.9, 31.0, 28.2. IR: ν 2977, 2911, 2857, 1710, 1644, 1559, 1473 1450, 1408 cm^{-1}. Mass calculated $[M+H]^+$: 160.1121, HRMS $[M+H]^+$: 160.1117.

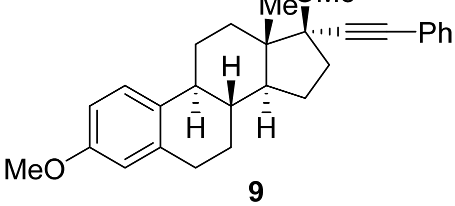

9

(8*R*,9*S*,13*S*,14*S*,17*R*)-17-phenylethynyl-3,17-dimethoxy-13-methyl-7,8,9,11,12,13,14,15,16,17-decahydro-6*H*-cyclopenta[*a*]phenanthrene (9)

Isolated as a white amorphous solid (1st run: 40.9 mg, 0.10 mmol, 51%; 2nd run (0.1 mmol scale): 21.4mg, 0.06 mmol, 58%; Avg yield: 55%). ^1H NMR (400 MHz, $CDCl_3$): δ

7.50-7.47 (m, 2H), 7.33-7.31 (m, 3H), 7.22 (d, *J* = 8.4 Hz, 1H), 6.73-6.71 (m, 1H), 6.64 (s, 1H), 3.78 (s, 3H), 3.49 (s, 3H), 2.89-2.85 (m, 2H), 2.37-2.34 (m, 2H), 2.28-2.23 (m, 1H), 2.14-2.06 (m, 2H), 1.92-1.82 (m, 3H), 1.58-1.35 (m, 5H), 0.94 (s, 3H) . ^{13}C NMR (100 MHz, $CDCl_3$): δ 157.6, 138.1, 132.8, 131.9, 128.5, 128.3, 126.5, 123.4, 113.9, 111.6, 90.5, 88.0, 55.4, 53.6, 50.0, 48.1, 43.8, 39.4, 37.0, 34.6, 30.0, 27.4, 26.8, 23.0, 13.1. IR: ν 3059, 2926, 2806, 2254, 2206, 2073, 1965, 1885, 1738, 1607, 1496, 1446 cm^{-1}. Mass calculated [M+Na]$^+$: 423.2295, HRMS [M+Na]$^+$: 423.2286.

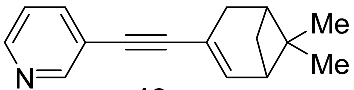

12

3-((6,6-dimethylbicyclo[3.1.1]hept-2-en-3-yl)ethynyl)pyridine (12)

Isolated as a clear oil (25.4 mg, 0.11 mmol, 57%). ^{1}H NMR (400 MHz, $CDCl_3$): δ 8.65 (s, 1H), 8.47 (d, *J* = 3.6 Hz, 1H), 7.68 (dt, *J* = 7.6, 2.0 Hz, 1H), 7.21 (qd, *J* = 4.0, 0.8 Hz, 1H), 6.14-6.12 (m, 1H), 2.49-2.34 (m, 4H), 2.16-2.13 (m, 1H), 1.33 (s, 3H), 1.29 (q, *J* = 9.2 Hz, 1H), 0.9 (s, 3H). ^{13}C NMR (100 MHz, $CDCl_3$): δ 152.2, 148.1, 138.3, 132.6, 129.7, 123.1, 121.1, 110.2, 93.5, 85.9, 47.1, 40.3, 38.2, 32.4, 31.6, 26.1, 21.2. IR: ν 3031, 2923 2822, 2210, 1710, 1590, 1563, 1477, 1408 cm^{-1}. Mass calculated [M+H]$^+$: 224.1439, HRMS [M+H]$^+$: 224.1441.

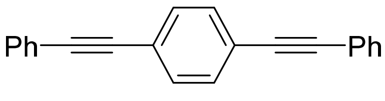

14

1,4-bis(phenylethynyl)benzene (14)[33]

Isolated as a white solid (13.1 mg, 0.05 mmol, 94%). ^{1}H NMR (400 MHz, $CDCl_3$): δ 7.54 (dd, 8.0, 4.4 Hz, 4H), 7.51 (s, 4H), 7.38-7.7.35 (m, 6H). ^{13}C NMR (100 MHz, $CDCl_3$): δ 131.8, 131.7, 128.6, 128.5, 123.2, 123.1, 91.4, 89.2. IR: ν 3084, 3053, 3025, 2963, 2929, 2853, 2199, 1729, 1639, 1597, 1510, 1482, 1434, 1400, 1288, 1221, 1162, 838, 760, 695 cm^{-1}. Mass calculated [M$^+$]: 278.1096, HRMS [M$^+$]: 278.1097. MP: 157-160°C.

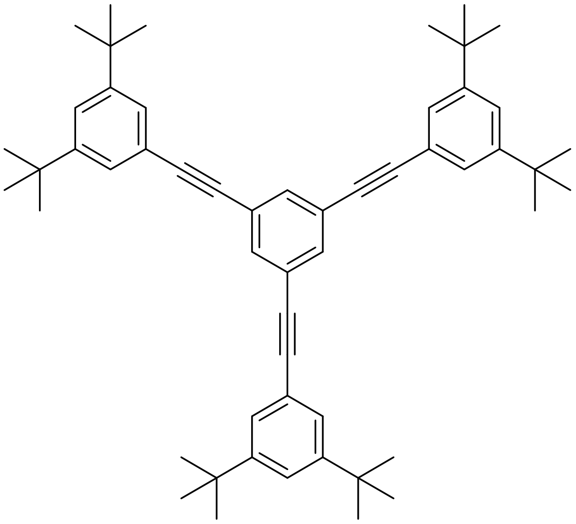

16

1,3,5-tris((3,5-di-tert-butylphenyl)-ethynyl)benzene (16)[34]

Isolated as a light brown oil (26.4 mg, 0.04 mmol, 74%). ^{1}H NMR (400 MHz, $CDCl_3$): δ 7.69 (s, 3H), 7.43 (q, *J* = 1.88 Hz, 3H), 7.40 (d, *J* = 1.8 Hz, 6H), 1.35 (s, 54H). ^{13}C NMR (100 MHz, $CDCl_3$): δ 151.1, 134.0, 126.1, 124.3, 123.2, 122.0, 91.7, 86.9, 35.0, 31.5. IR: ν 2967, 2932, 2856, 1670, 1597, 1561, 1465, 1443, 1378, 1358, 1263, 1249, 1094, 1021, 881, 793 cm^{-1}. Mass calculated [M$^+$]: 714.5165, HRMS [M$^+$]: 714.5164.

For substrate **3o**, following the above general procedure, no reaction was observed at 150 °C. However, using mesitylene as the solvent at 170 °C only led to formation of 2-phenylfuran (**4o'**), which was confirmed by ^{1}H-NMR, IR and GC-MS (Scheme S1).[52] The yield of **4o'** was not determined due to the separation difficulty from mesitylene.

Scheme S1. Cycloisomerization of Compound **3o** to Furan[a]

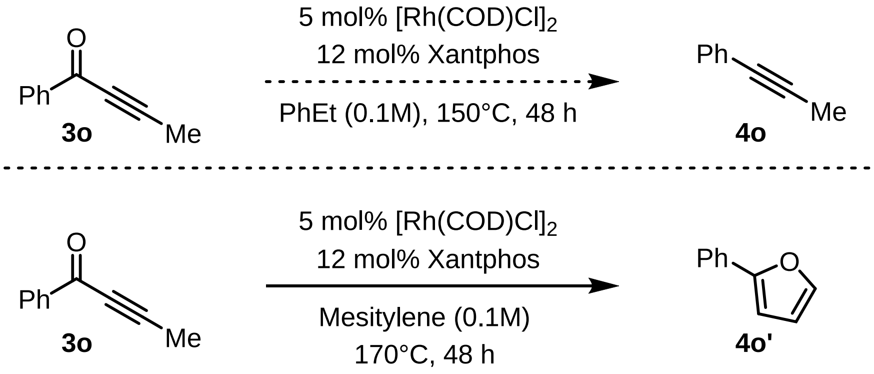

[a] Reactions on 0.200 mmol scale

References:

1. (a) Substrate **1u** prepared according to a literature procedure: S. Sasaki, Y. Ikekame, M. Tanayama, T. Yamauchi, K. Higashiyama, *Synlett.*, 2012, **23**, 2699-2703; (b) Substrate **3o** prepared according to a literature procedure: J. Suffert, D. Toussaint, *J. Org. Chem.*, 1995, **60**, 3550-3553.
2. X.-F. Wu, H. Neumann, M. Beller, *Chem. Eur. J.*, 2010, **16**, 12104-12107.
3. A. Park, K. Park, Y. Kim, S. Lee, *Org. Lett.*, 2011, **13**, 944-947.
4. X.-F. Wu, B. Sundararaju, H. Neumann, P. H. Dixneuf, M. Beller, *Chem. Eur. J.*, 2011, **17**, 106-110.
5. R. Lerebours, A. Camacho-Soto, C. Wolf, *J. Org. Chem.*, 2005, **70**, 8601-8604.
6. P. Rao, N. Praveen, *J. Med. Chem.*, 2004, **47**, 3972-3990.
7. K. Yoshizawa, T. Shioiri, *Tetrahedron*, 2007, **63**, 6259-6286.
8. Y. Nishihara, D. Saito, E. Inoue, Y. Okada, M. Miyazaki, Y. Inoue, K. Takagi, *Tetrahedron Lett.*, 2010, **51**, 306-308.
9. V. O. Iaroshenko, S. Mkrtchyan, A. Villinger, *Synthesis*, 2013, **45**, 205-218.

10. R. J.Cox, D. J. Ritson, T. A. Dane, J. Berge, J. P. H. Charmant, A. Kantacha, *Chem. Commun.*, 2005, 1037-1039.
11. J. Y. Chen, T. C. Lin, S. C. Chen, A. J. Chen, C. Y. Mou, F. Y. Tsai, *Tetrahedron*, 2009, **65**, 10134-10141.
12. C. Boersch, E. Merkul, T. J. J. Muller, *Angew. Chem. Int. Ed.*, 2011, **50**, 10448-10452.
13. J. P. Waldo, R. C. Larock, *J. Org. Chem.*, 2007, **72**, 9643-9647.
14. J. Liu, X. Peng, W. Sun, Y. Zhao, C. Xia, *Org. Lett.*, 2008, **10**, 3933-3936.
15. D. A. Alonso, C. Nájera, M. C. Pacheco, *J. Org. Chem.*, 2004, **69**, 1615−1619.
16. C. Feng, T. P. Loh, *Chem. Commun.*, 2010, **46**, 4779-4781.
17. P. H. Li, L. Wang, H. J. Li, *Tetrahedron*, 2005, **61**, 8633-8640.
18. H. Huang, H. Liu, H. Jiang, K. Chen, *J. Org. Chem.*, 2008, **73**, 6037-6040.
19. J. Moon, M. Jang, S. Lee, *J. Org. Chem.*, 2009, **74**, 1403-1406.
20. M. Feuerstein, F. Berthiol, H. Doucet, M. Santelli, *Synthesis*, 2004, 1281-1289.
21. Y. Liang, Y.-X. Xie, J.-H. Li, *J. Org. Chem.*, 2006, **71**, 379-381.
22. L. Melzig, A. Metzger, P. Knochel, *Chem. Eur. J.*, 2011, **17**, 2948-2956.
23. M.-Y. Wu, J.-C. Mao, J. Guo, S.-J. Ji, *Eur. J. Org. Chem.*, 2008, 4050-4054.
24. S. A. Johnson, F.-Q. Liu, M. C. Suh, S. Zőrcher, M. Haufe, S. S. Mao, T. D. Tilley, *J. Am. Chem. Soc.*, 2003, **125**, 4199-4211.
25. A. Orita, N. Yoshioka, P. Struwe, A. Braier, A. Beckmann, J. Otera, *Chem. Eur. J.*, 1999, **5**, 1355-1363.
26. A. Sagadevan, K. C. Hwang, *Adv. Synth. Catal.*, 2012, **354**, 3421-3427.
27. A. D. Finke, E. C. Elleby, M. J. Boyd, H. Weissman, J. S. Moore, *J. Org. Chem.*, 2009, **74**, 8897-8900.
28. M. Csékei, Z. Novák, A. Kotschy, *Tetrahedron*, 2008, **64**, 975-982.
29. J. Moon, M. Jeong, H. Nam, J. Ju, J. H. Moon, H. M. Jung, S. Lee, *Org. Lett.*, 2008, **10**, 945-948.
30. Z. Wang, W. Lin, C. Jiang, Q. Guo, *Chin. Sci. Bull.*, 2001, **46**, 1606-1608.
31. J. Gil-Molto, C. Najera, *Adv. Synth. Catal.*, 2006, **348**, 1874-1882.
32. A. M. Johnson, O. Moshe, A. S. Gamboa, B. W. Langloss, J. F. K. Limtiaco, C. K. Larive, R. J. Hooley, R.J. *Inorg. Chem.*, 2011, **50**, 9430-9442.
33. S. Prateeptongkum, K. M. Driller, R. Jackstell, A. Spannenberg, M. Beller, *Chem. Eur. J.*, 2010, **16**, 9606-9615.
34. H. Yu, J. Li, Z. Kou, X. Du, Y. Wei, H.-K. Fun, J. Xu, Y. Zhang, *J. Org. Chem.*, 2010, **75**, 2989-3001.
35. X. Qu, T. Li, P. Sun, Y. Zhu, H. Yang, J. Mao, *Org. Biomol. Chem.*, 2011, **9**, 6938-6942.
36. A. L.Thompson, K. M. Gaab, J. Xu, C. J. Bardeen, T. J. Martinez, *J. Phys. Chem. A*, 2004, **108**, 671-682.
37. H. Yuan, Y. Shen, S. Yu, L. Shan, Q. Sun, W. Zhang, *Synth. Comm.*, 2013, **43**, 2817-2823.
38. B. Huang, L. Yin, M. Cai, *New J. Chem.*, 2013, **37**, 3137-3144.
39. I. E. Sokolov, A. S. Zania, S. I. Shergina, M. S. Shvartsberg, *Izvestiya Akademii Nauk*, 1996, 147-149.
40. C. Taylor, Y. Bolshan, *Org. Lett.*, 2014, **16**, 488-491.
41. W. Kim, K. Park. A. Park, J. Choe, S. Lee, *Org. Lett.*, 2013, **15**, 1654-1657.
42. J. Liu, X. Peng, W. Sun, Y. Zhao, C. Xia, *Org. Lett.*, 2008, **10**, 3933-3936.
43. C.-M. Yu, J-H. Kwein, P-S. Ho, S.-C. Kang, G. Lee, *Synlett*, 2005, 2631-2634.

44. W. P. Gallagher, R. E. Maleczka, *J. Org. Chem.*, 2003, **68**, 6775-6779.
45. J. Liu, X. Xie, S. Ma, *Synthesis*, 2012, **44**, 1569-1576.
46. X.-F. Wu, H. Neumann, M. Beller, *Angew. Chem. Int. Ed.*, 2011, **50**, 11142-11146.
47. CAS Number: 1817-57-8
48. C.-C. Tai, M.-S. Yu, Y.-L. Chen, W.-H. Chuang, T.-H. Lin, G. P. A. Yap, T.-G. Ong, *Chem. Commun.*, 2014, **50**, 4344-4346.
49. J. L. Garcia Ruano, J. Aleman, L. Marzo, C. Alvarado, M. Tortosa, S. Diaz-Tendero, A. Fraile, *Chem. Eur. J.*, 2012, **18**, 8414-8422.
50. Z.-F. Xu, C.-X. Cai, J.-T. Liu, *Org. Lett.*, 2013, **15**, 2096-2099.
51. S. R. K. Minkler, N .A. Isley, D. J. Lippincott, N. Krause, B. H. Lipshutz, *Org. Lett.*, 2014, **16**, 724-726.
52. For selected examples of cycloisomerizations of ynones to furans, see: (a) H. Sheng, S. Lin, Y. Z. Huang, *Tetrahedron Lett.*, 1986, **27**, 4893; (b) A. Kel'in, V. Gevorgyan, *J. Org. Chem.*, 2002, **67**, 95.

Section 2: Computational details

Part I. Computed Energies of All Stationary Points

Thermal correction to Gibbs Free Energy by B3LYP ($TCGFE_{\mathrm{B3LYP}}$ in Hartree)

Sum of electronic and thermal Enthalpies by B3LYP (H_{B3LYP}, in Hartree)

Sum of electronic and thermal Free Energies by B3LYP (G_{B3LYP}, in Hartree)

Single point energies were calculated by M06L//B3LYP method (E_{M06L}, in Hartree)

Total free energy in solution calculated by M06L//B3LYP method ($E_{\mathrm{M06L,sol}}$, in Hartree)

name	$TCGFE_{\mathrm{B3LYP}}$	H_{B3LYP}	G_{B3LYP}	E_{M06L}	$E_{\mathrm{M06L,sol}}$
CO	-0.014102	-113.298573	-113.321016	-113.330193	-113.325763
CAT-2a	0.704040	-3371.741858	-3371.883626	-3372.973193	-3373.026397
1a	0.159441	-652.555606	-652.611834	-652.859999	-652.879496
2a	0.152858	-539.246874	-539.297935	-539.514874	-539.531737
INT1-A	0.710957	-3485.055655	-3485.202352	-3486.322624	-3486.377773
INT2-A	0.713023	-3485.042641	-3485.187255	-3486.311292	-3486.365565
INT2-B	0.710970	-3485.024666	-3485.170579	-3486.298465	-3486.352335
INT3-A	0.709872	-3485.023925	-3485.170812	-3486.297685	-3486.347748
INT3-deCO	0.702682	-3371.696597	-3371.839547	-3372.921221	-3372.980061
INT4-A	0.708357	-3485.016572	-3485.164562	-3486.290939	-3486.344336
INT5-A	0.708069	-3485.064862	-3485.213461	-3486.338217	-3486.388933
TS1-A	0.713029	-3485.018250	-3485.160860	-3486.286566	-3486.340722
TS1-B	0.711718	-3484.978431	-3485.122242	-3486.250577	-3486.305055
TS2-A	0.710139	-3485.013013	-3485.158302	-3486.285456	-3486.336356
TS3-A	0.707743	-3485.001021	-3485.148031	-3486.273472	-3486.325423
TS3S-A	0.707389	-3484.989620	-3485.136746	-3486.262244	-3486.314302
TS3-deCO	0.701510	-3371.667168	-3371.809519	-3372.892620	-3372.949072

1t	0.110417	-460.874719	-460.923370	-461.099773	-461.114731
2t	0.103453	-347.565198	-347.609352	-347.755158	-347.766859
CAT-2t	0.654444	-3180.057555	-3180.192473	-3181.205912	-3181.254683
INT1-1t	0.662093	-3293.378382	-3293.517514	-3294.561512	-3294.612127
INT2-1t	0.659660	-3293.363860	-3293.505253	-3294.542752	-3294.598155
INT3-1t	0.659639	-3293.352948	-3293.493567	-3294.536967	-3294.584053
INT4-1t	0.660565	-3293.345583	-3293.485153	-3294.532173	-3294.580926
INT5-1t	0.659716	-3293.384817	-3293.525733	-3294.572152	-3294.618917
TS1-1t	0.661165	-3293.336023	-3293.474232	-3294.522219	-3294.572082
TS2-1t	0.660101	-3293.338335	-3293.477008	-3294.521918	-3294.569937
TS3-1t	0.656915	-3293.317429	-3293.458815	-3294.501802	-3294.550400

Part II.

1. Discussion of Different Structures of INT1-A

To find the minimum of the starting intermediate of the catalytic cycle (**INT1-A**), we go through different coordination structures. Here we present four main coordination structures: the carbonyl group coordinating (**S1**), the alkynyl group coordinating (**S2**), the carbonyl group coordinating with extra coordination of the oxygen atom from xantphos ligand (**S3**) and the alkynyl group coordinating with extra coordination of the oxygen atom from xantphos ligand (**INT1-A**). The computational results indicate that coordination of the carbonyl group is more thermodynamically unstable than coordination of the alkynyl group (**S1** vs **S2**, **S3** vs **INT1-A**). It also shows that the extra coordination of the oxygen atom from the xantphos ligand plays a stablizing effect in **S3** and **INT1-A**. This could be understood by the extra coordination of the oxygen atom making the rhodium center become approximately 18e$^-$ which stabilizes the thermodynamic energy of **S3** and **INT1-A** compared to **S1** and **S2** (16e$^-$ structures).

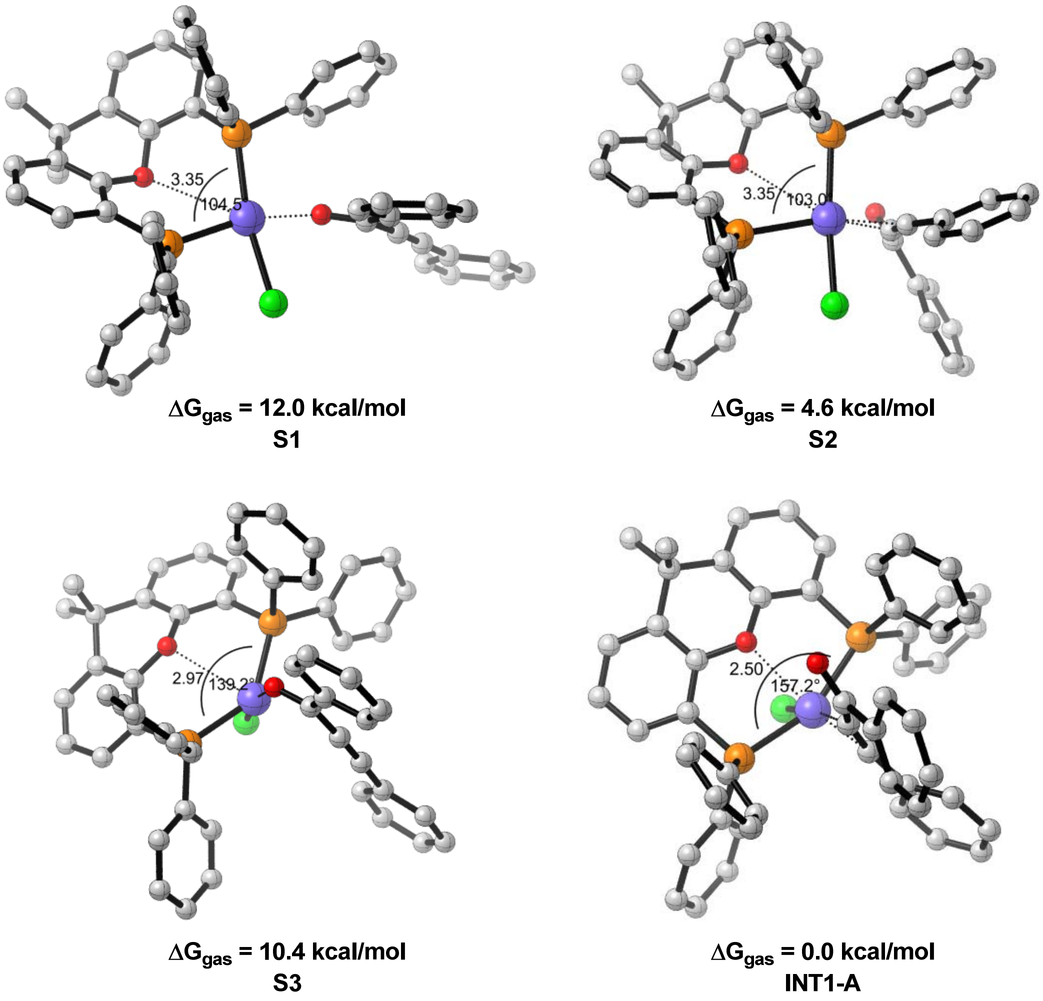

Figure S1 Four mainly coordination structures of starting intermediate (distances in Å).

2. Discussion of TS1

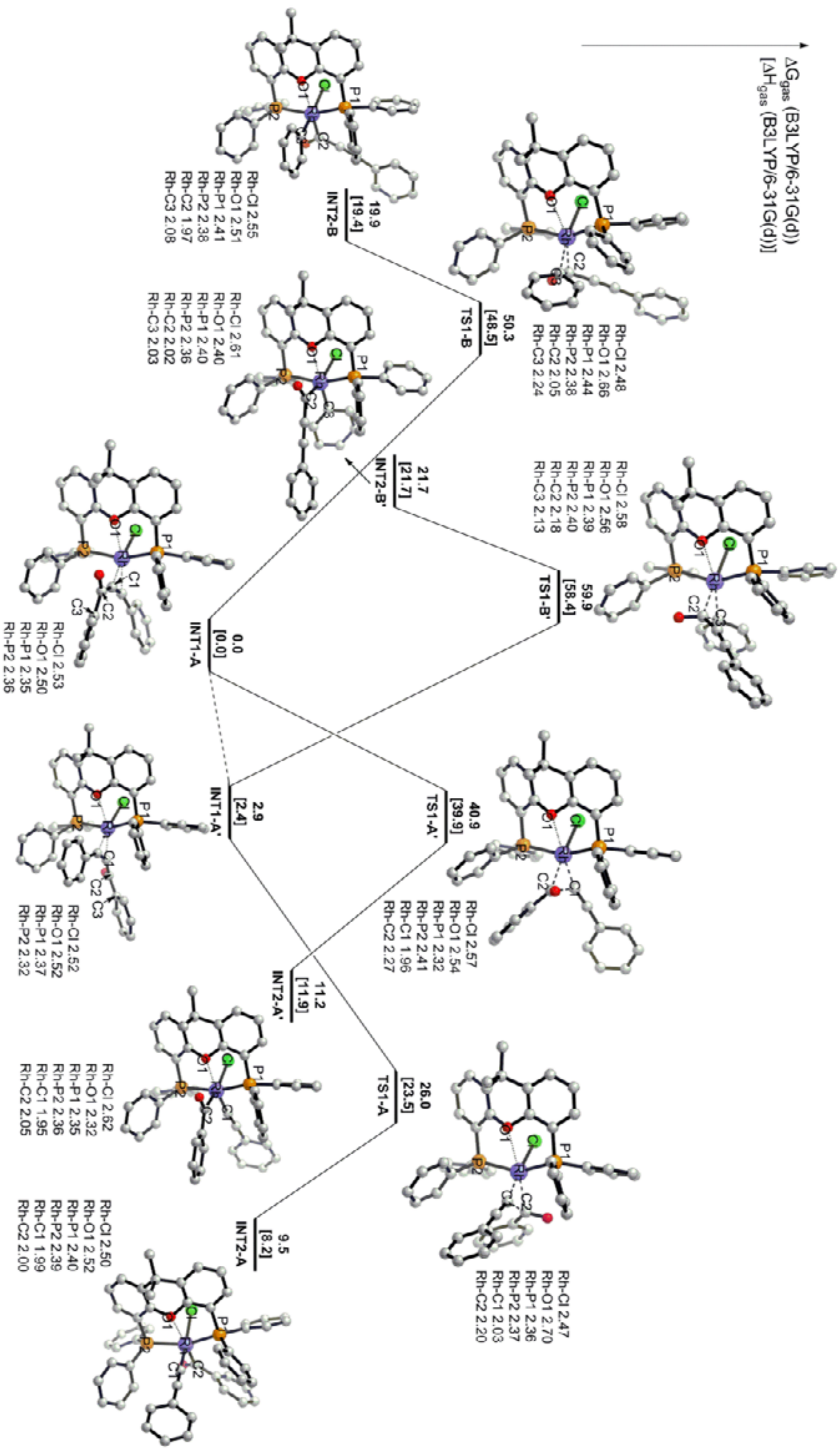

Figure S2 Discussion of TS1 (distances in Å, hydrogen atoms were omitted for clarity).

3. Discussion of the Difference of Reactivities between Different Ligands: Xantphos vs dppp

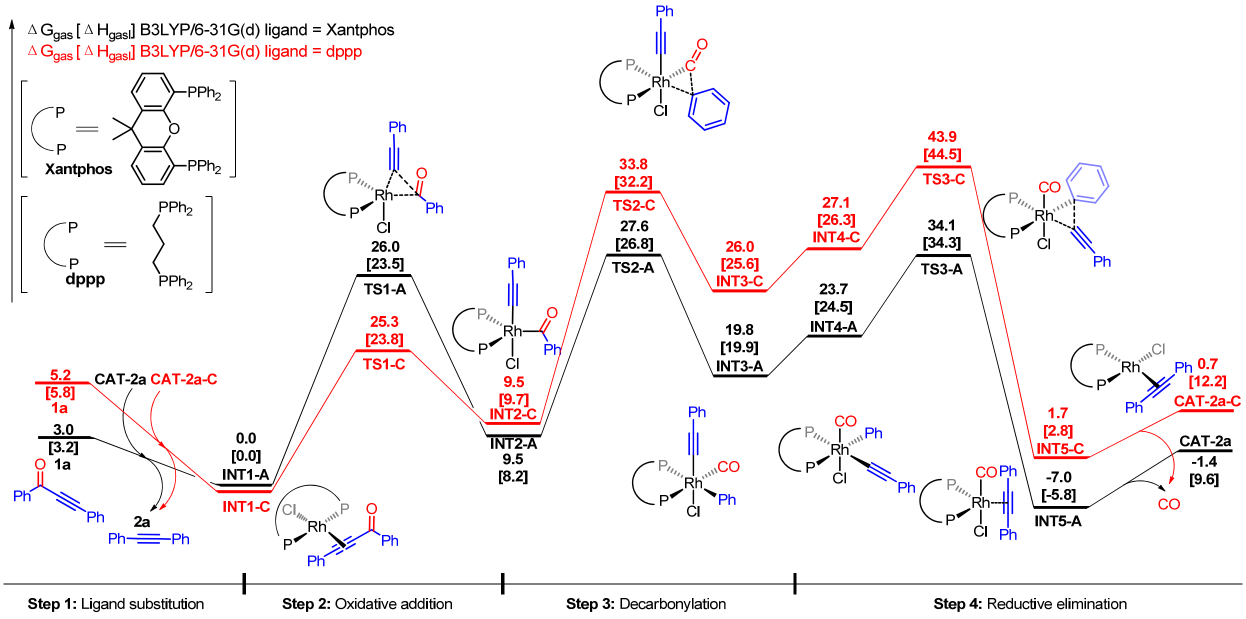

Figure S3 Comparison of the energy profiles of xantphos (black) and dppp (red).

We have explored the reactivities between xantphos and dppp in B3LYP/6-31G(d) level (figure S1). At the oxidative addition step, the activation energy of transition-state structures with dppp is slightly more favorable than that with xantphos. The activation energy of the decarbonylation step becomes 6.2 kcal/mol higher than that with xantphos. The reductive elimination step is also the rate-determining step for both ligands. The activation energy of dppp is 6.4 kcal/mol higher than that of xantphos for this step (16.8 kcal/mol for dppp and 10.4 kcal/mol for xantphos) and the total activation energy is 9.8 kcal/mol higher. This indicates that using dppp as the ligand is disfavored in this reaction.

Part III. Coordinates of All Stationary Points

1. Coordinates of all stationary points in figure 2

CO

Cartesian coordinates

ATOM	X	Y	Z
O	0.00000000	0.00000000	0.48761100
C	0.00000000	0.00000000	-0.65014800

CAT-2a

Cartesian coordinates

ATOM	X	Y	Z
C	2.41489800	1.84216600	-0.47317400
C	3.63026400	2.45730800	-0.80426900
H	4.49118000	1.83745400	-1.03126400
C	3.74360200	3.84253800	-0.86929200
H	4.68997500	4.30008400	-1.14236600
C	2.63989700	4.64028500	-0.57703600
H	2.74545500	5.71851500	-0.61617400
C	1.40888400	4.07543500	-0.22872200
C	1.30967200	2.67677600	-0.21315900
C	0.20149400	4.90153000	0.22303100
C	-1.06892600	4.17568400	-0.22877100
C	-1.08413200	2.77373700	-0.21085900
C	-2.25446300	2.03130200	-0.46660300

C	-3.41490900	2.74261900	-0.80174700
H	-4.32357700	2.19503000	-1.02850500
C	-3.41393300	4.13234000	-0.87230400
H	-4.31916500	4.66480300	-1.14905400
C	-2.24936800	4.83826200	-0.58035200
H	-2.26656600	5.92135500	-0.62273400
C	0.20341800	4.95138900	1.77950300
H	0.16590900	3.94576700	2.21002100
H	-0.66634400	5.51096500	2.14295700
H	1.11250100	5.44456900	2.14289100
C	0.25945400	6.34608800	-0.30625000
H	1.15571500	6.85704400	0.05713500
H	-0.59237500	6.92727100	0.05848200
H	0.25991600	6.37910500	-1.40075700
C	3.53058800	-0.60958400	-1.64801000
C	4.88041700	-0.82890200	-1.32214700
H	5.23450000	-0.65020800	-0.31199600
C	5.77632900	-1.29102400	-2.28776700
H	6.81658200	-1.45688000	-2.01956400
C	5.33656700	-1.54386000	-3.58819300
H	6.03403400	-1.90709700	-4.33865200
C	3.99675100	-1.33179000	-3.91813300
H	3.64595700	-1.52687400	-4.92812200
C	3.09535900	-0.87085700	-2.95728200
H	2.05777400	-0.69176300	-3.21932600

C	3.14562000	-0.43763000	1.19391200
C	3.30445900	-1.80027200	1.50221100
H	2.96030900	-2.55733500	0.80332900
C	3.89936400	-2.19066000	2.70038000
H	4.01550700	-3.24773600	2.92377900
C	4.33629900	-1.22817200	3.61420000
H	4.79527800	-1.53372700	4.55063700
C	4.17671200	0.12566600	3.32046900
H	4.51383400	0.88080300	4.02585000
C	3.58494700	0.52030700	2.11782500
H	3.47314500	1.57754900	1.90027700
C	-3.56964800	-0.33829600	-1.61464100
C	-4.93368300	-0.41923900	-1.28389800
H	-5.26698700	-0.17847100	-0.27942900
C	-5.87064900	-0.82225300	-2.23673300
H	-6.92170200	-0.88069200	-1.96577900
C	-5.45772400	-1.15442700	-3.52827900
H	-6.18736000	-1.47203600	-4.26880200
C	-4.10430100	-1.08126200	-3.86219300
H	-3.77494600	-1.34008400	-4.86498800
C	-3.16189800	-0.67910100	-2.91427600
H	-2.11235500	-0.60816400	-3.17936700
C	-3.15004800	-0.17409400	1.22192200
C	-3.39923100	-1.51933500	1.54703800
H	-3.11051600	-2.30646900	0.85624700

C	-4.01149700	-1.85431500	2.75320300
H	-4.19731000	-2.89837100	2.99037500
C	-4.37714000	-0.85376800	3.65754300
H	-4.85030600	-1.11656400	4.59988200
C	-4.12835800	0.48271000	3.34665500
H	-4.40989600	1.26711900	4.04446400
C	-3.51788400	0.82219500	2.13648200
H	-3.33460800	1.86649000	1.90534300
O	0.08731000	2.08592700	0.09312000
P	2.30909500	-0.00893300	-0.39295100
P	-2.29700800	0.17780400	-0.37521300
Cl	0.01266200	0.69211200	-2.80542500
Rh	-0.01338300	-0.35853900	-0.50011700
C	-0.10325300	-2.32676300	0.12889900
C	-0.05977200	-1.61698900	1.19886800
C	-0.02481300	-1.50758600	2.63579800
C	0.01364500	-2.66073400	3.45067600
C	-0.02765600	-0.24962800	3.26943400
C	0.04158200	-2.55526600	4.83772200
H	0.03301900	-3.63964900	2.98134800
C	-0.00161600	-0.14868300	4.65855500
H	-0.05322200	0.64178300	2.65083800
C	0.03183000	-1.29846100	5.45077900
H	0.07176300	-3.45709000	5.44451300
H	-0.00683600	0.83313400	5.12554200

H	0.05186500	-1.21784500	6.53444800
C	-0.19629400	-3.57302100	-0.58973800
C	-0.03470500	-3.61203000	-1.98960600
C	-0.45506400	-4.78355800	0.09076600
C	-0.11773700	-4.81966100	-2.67933700
H	0.15416100	-2.68067000	-2.51496500
C	-0.53971900	-5.98566200	-0.60607700
H	-0.59915700	-4.76801200	1.16697000
C	-0.36922600	-6.01094400	-1.99369100
H	0.01188900	-4.83026700	-3.75849000
H	-0.74114300	-6.90699400	-0.06513600
H	-0.43509300	-6.95105000	-2.53486700

1a

Cartesian coordinates

ATOM	X	Y	Z
C	-1.37067500	1.34003200	-0.00001500
O	-1.58226500	2.54924600	-0.00003100
C	-0.00895600	0.83824300	-0.00000300
C	1.15700300	0.48993100	0.00000300
C	2.52789400	0.10211300	0.00000700
C	2.89045600	-1.25980600	-0.00005300
C	3.54019800	1.08329000	0.00007600
C	4.23316100	-1.62714300	-0.00004700
H	2.11166800	-2.01612800	-0.00010600

C	4.88018900	0.70622400	0.00008300
H	3.25934700	2.13170200	0.00012400
C	5.22980300	-0.64713300	0.00002100
H	4.50391400	-2.67925100	-0.00009600
H	5.65394500	1.46872400	0.00013600
H	6.27675300	-0.93770400	0.00002600
C	-2.48151300	0.34089700	-0.00001200
C	-2.24273500	-1.04051300	0.00003700
C	-3.80213900	0.81640400	-0.00005500
C	-3.31142800	-1.93615000	0.00004300
H	-1.22028500	-1.40601600	0.00006900
C	-4.86733000	-0.07890600	-0.00005100
H	-3.96362500	1.88939200	-0.00009000
C	-4.62340500	-1.45677000	-0.00000200
H	-3.12204900	-3.00604600	0.00008200
H	-5.88853200	0.29229800	-0.00008700
H	-5.45614800	-2.15520500	0.00000200

2a

Cartesian coordinates

ATOM	X	Y	Z
C	-0.60825700	-0.00000200	0.00000100
C	0.60825700	-0.00000200	-0.00000200
C	2.03323900	-0.00000100	0.00000000
C	2.75093200	-1.21362400	0.00184100

C	2.75093000	1.21362400	-0.00184200
C	4.14329400	-1.20876100	0.00184600
H	2.20316900	-2.15098900	0.00326200
C	4.14329200	1.20876300	-0.00184500
H	2.20316600	2.15098800	-0.00326400
C	4.84470900	0.00000100	0.00000100
H	4.68362100	-2.15162300	0.00328300
H	4.68361800	2.15162500	-0.00328300
H	5.93125100	0.00000200	0.00000100
C	-2.03323900	-0.00000100	0.00000000
C	-2.75093200	-1.21362400	-0.00184100
C	-2.75093000	1.21362400	0.00184200
C	-4.14329400	-1.20876100	-0.00184500
H	-2.20316900	-2.15098900	-0.00326300
C	-4.14329200	1.20876300	0.00184500
H	-2.20316500	2.15098700	0.00326300
C	-4.84470900	0.00000100	0.00000000
H	-4.68362100	-2.15162300	-0.00328300
H	-4.68361800	2.15162500	0.00328300
H	-5.93125100	0.00000200	0.00000000

INT1-2a

Cartesian coordinates

ATOM	X	Y	Z
C	-1.64050700	2.75793100	0.31822400

C	-2.07571900	4.08999700	0.33127700
H	-1.40769900	4.86894000	-0.02063000
C	-3.35480900	4.42421100	0.76460400
H	-3.68129900	5.45995400	0.75375500
C	-4.21220100	3.42550800	1.22046200
H	-5.19918700	3.70166700	1.57371800
C	-3.81990300	2.08321100	1.23938200
C	-2.54369100	1.76739100	0.75190500
C	-4.66840800	0.96922300	1.85813100
C	-4.38567300	-0.32985800	1.09914800
C	-3.08886900	-0.56097100	0.61742100
C	-2.70587700	-1.79556600	0.05682700
C	-3.68639800	-2.78941800	-0.06516500
H	-3.42314400	-3.74111800	-0.51443500
C	-4.99268400	-2.56689400	0.35844400
H	-5.74366600	-3.34274900	0.24185300
C	-5.33136700	-1.34822300	0.94199000
H	-6.34807000	-1.19616400	1.28620800
C	-4.20568600	0.77497400	3.33273800
H	-3.14208600	0.52211400	3.38659200
H	-4.77424700	-0.03436300	3.80550900
H	-4.36731800	1.69573400	3.90531600
C	-6.16755800	1.32035300	1.86030000
H	-6.35343800	2.22897800	2.44001700
H	-6.75074100	0.52901900	2.33967900

H	-6.55199700	1.47015300	0.84591900
C	0.47946600	3.61371900	-1.53358600
C	1.00721700	4.87092700	-1.19016700
H	1.19599400	5.11747700	-0.15011500
C	1.30811700	5.80662900	-2.18101100
H	1.71607100	6.77432500	-1.90058100
C	1.09152000	5.49754500	-3.52525700
H	1.32949300	6.22550500	-4.29665700
C	0.57263100	4.24939200	-3.87360800
H	0.40272500	4.00121900	-4.91793100
C	0.26876900	3.30889300	-2.88771900
H	-0.15055600	2.34535000	-3.15819600
C	1.15554700	2.84298000	1.15635300
C	2.54845000	2.82059500	0.96420800
H	2.95991300	2.57015100	-0.00986300
C	3.41330500	3.12595200	2.01423900
H	4.48732000	3.10704000	1.84958500
C	2.90142500	3.44970400	3.27346100
H	3.57615300	3.68546200	4.09217300
C	1.52129000	3.46586200	3.47465100
H	1.11468400	3.71256100	4.45180200
C	0.65272400	3.16249500	2.42421200
H	-0.41801400	3.17233300	2.59775900
C	-1.14445600	-3.32096300	-1.90735100
C	-1.20458600	-4.71144800	-1.70845100

H	-1.14055600	-5.12231400	-0.70613200
C	-1.33387700	-5.57747900	-2.79559800
H	-1.37759100	-6.65020600	-2.62573900
C	-1.40270800	-5.06776000	-4.09333600
H	-1.50073100	-5.74301000	-4.93951100
C	-1.34335500	-3.68815500	-4.29866800
H	-1.39850000	-3.28366400	-5.30589800
C	-1.21261700	-2.81633600	-3.21637700
H	-1.18730800	-1.74302900	-3.37664600
C	-0.27683300	-3.19708700	0.84143700
C	0.96850900	-3.81450500	0.62962800
H	1.47234100	-3.70489900	-0.32706100
C	1.56035000	-4.58176800	1.63212000
H	2.52240500	-5.05339800	1.45187100
C	0.92502200	-4.73340000	2.86692800
H	1.38983000	-5.32582200	3.65028800
C	-0.30472100	-4.11458800	3.09070700
H	-0.80370100	-4.22258100	4.05002100
C	-0.90262500	-3.35072600	2.08606800
H	-1.85885900	-2.87498300	2.27595500
O	-2.13356800	0.44166900	0.71457400
P	0.07384000	2.34697300	-0.25054700
P	-0.96949300	-2.13649800	-0.49742500
Cl	-1.76952800	0.53379800	-2.48242000
Rh	-0.08609400	0.04262800	-0.65973900

C	1.88683200	-0.53919300	1.93057800
O	1.05957700	-0.21408500	2.78267400
C	1.60122400	-0.41135100	0.51066200
C	1.92367300	-0.34383000	-0.73449900
C	2.87481500	-0.31377300	-1.82272100
C	4.25959100	-0.19148800	-1.57762400
C	2.42474600	-0.37913800	-3.15608300
C	5.16142900	-0.14978200	-2.63790200
H	4.61515700	-0.12516800	-0.55453500
C	3.33456500	-0.34672400	-4.21051800
H	1.35663800	-0.44653400	-3.33971100
C	4.70397500	-0.23262700	-3.95647500
H	6.22475000	-0.05091900	-2.43529100
H	2.97337600	-0.40271100	-5.23389400
H	5.41129700	-0.20184100	-4.78101500
C	3.23506700	-1.05166300	2.36360400
C	4.05813600	-1.83176100	1.54027200
C	3.65847200	-0.75453100	3.66792000
C	5.28819800	-2.29727300	2.00831200
H	3.72640400	-2.08894300	0.54005300
C	4.89102400	-1.20877900	4.13096500
H	3.00162700	-0.16358000	4.29748800
C	5.70988800	-1.98184300	3.30185600
H	5.91523900	-2.90840800	1.36410700
H	5.21487600	-0.96399400	5.13940000

H	6.67022800	-2.33980500	3.66424900

TS1-A

Cartesian coordinates

ATOM	X	Y	Z
Rh	0.03574200	0.05154000	0.71787300
C	-0.53257700	1.76694300	-0.20305100
C	-0.56707800	2.04546400	1.42790700
O	0.36592700	2.67323600	1.92923300
C	-0.83165800	2.55470100	-1.11552300
C	4.36164700	-3.30620100	-1.12984300
H	4.85237600	-4.18080100	-1.54244500
C	3.00663800	-3.08193500	-1.38639000
C	2.40192100	-1.95211200	-0.81429000
C	2.15708600	-3.95462300	-2.31281400
C	0.75063400	-4.02323100	-1.71195900
C	0.22896000	-2.85983900	-1.12373100
C	-1.09746900	-2.78350900	-0.65542200
C	-1.87831800	-3.94427200	-0.75327100
H	-2.89349700	-3.93586500	-0.37648000
C	-1.37004000	-5.12162900	-1.29193700
H	-1.99295900	-6.01016200	-1.33926700
C	-0.06421000	-5.15571500	-1.77412400
H	0.31742700	-6.07382200	-2.20651100
C	2.06536400	-3.25332700	-3.70015900

H	1.64087500	-2.24859800	-3.61180100
H	1.43038000	-3.83518000	-4.37840100
H	3.06238000	-3.16382500	-4.14689200
C	2.76925900	-5.35040700	-2.51910500
H	3.76264900	-5.27363700	-2.97114300
H	2.16038700	-5.94192900	-3.20939600
H	2.85873800	-5.90050400	-1.57641100
C	3.31459600	0.89884100	2.17155000
C	4.54951800	1.56837800	2.12924200
H	4.96365200	1.88935400	1.17867700
C	5.24745800	1.84423500	3.30599500
H	6.20125600	2.36330600	3.25660600
C	4.71766600	1.46212800	4.53973600
H	5.25929600	1.68047700	5.45660400
C	3.48713700	0.80522100	4.59033600
H	3.06576900	0.50896500	5.54732200
C	2.78539200	0.52653600	3.41653300
H	1.83583100	0.00492900	3.45796500
C	2.93728100	1.83049600	-0.52391700
C	2.78361700	3.18005700	-0.15677300
H	2.30139900	3.42739300	0.78298000
C	3.24129300	4.19547500	-0.99715400
H	3.13384900	5.23279900	-0.68958800
C	3.83421400	3.88512600	-2.22503200
H	4.19331700	4.67834300	-2.87585600

C	3.96191500	2.55036500	-2.60917700
H	4.41710600	2.29717800	-3.56323800
C	3.52131800	1.52931500	-1.76294500
H	3.64886000	0.49588600	-2.06773700
C	-3.12474200	-1.80638100	1.25027600
C	-4.35902500	-2.36425000	0.86855900
H	-4.61634200	-2.46012100	-0.18168900
C	-5.27764100	-2.78376700	1.83120400
H	-6.22635100	-3.21232800	1.51840400
C	-4.97960900	-2.65010500	3.18904300
H	-5.69612000	-2.97644300	3.93848300
C	-3.76140200	-2.09257800	3.57752100
H	-3.52198300	-1.98215800	4.63150400
C	-2.83999000	-1.66999100	2.61755900
H	-1.88773900	-1.25241400	2.92318100
C	-2.83943800	-0.63415400	-1.40473900
C	-4.09922500	-0.03122700	-1.25538400
H	-4.54111800	0.06570100	-0.26978600
C	-4.80831200	0.42885600	-2.36859000
H	-5.79068000	0.87389400	-2.23092400
C	-4.26479800	0.30971900	-3.64855800
H	-4.81878500	0.66145700	-4.51481800
C	-3.00150800	-0.26417100	-3.80651800
H	-2.56386400	-0.35600300	-4.79698500
C	-2.29710100	-0.73093300	-2.69680600

H	-1.31995700	-1.18097500	-2.84093000
O	1.04540500	-1.74078400	-1.02820400
P	2.35075000	0.51825200	0.64135500
P	-1.84674700	-1.22420000	0.04813700
Cl	0.59492800	-1.86491900	2.17897400
C	3.12757900	-1.01734100	-0.05135600
H	5.06923200	-0.59172200	0.77140200
C	5.09978400	-2.41589100	-0.35278300
C	4.48831100	-1.27922900	0.16673300
H	6.15116600	-2.60555000	-0.15677700
C	-1.15712800	3.48737200	-2.13006100
C	-2.45995600	3.53495300	-2.67686200
C	-0.18020600	4.38828500	-2.61249200
C	-2.77201800	4.45956500	-3.66789500
H	-3.20771700	2.83417400	-2.32184000
C	-0.50290400	5.30654500	-3.60620900
H	0.82100900	4.34700500	-2.19782100
C	-1.79712000	5.34712000	-4.13444300
H	-3.77654400	4.48866800	-4.08113000
H	0.25503000	5.99463200	-3.97055000
H	-2.04526300	6.06777900	-4.90918400
C	-1.92082700	2.06114900	2.08486400
C	-1.93978500	2.11151000	3.48875500
C	-3.13324700	2.09392000	1.38673200
C	-3.15012700	2.16177500	4.17618600

H	-0.99597200	2.11184800	4.02373000
C	-4.34385900	2.16310900	2.07785100
H	-3.12366100	2.08524800	0.30328500
C	-4.35809000	2.18873500	3.47341100
H	-3.15113800	2.19043800	5.26280900
H	-5.27819900	2.20440600	1.52328600
H	-5.30201500	2.23764900	4.00980300

INT2-A

Cartesian coordinates

ATOM	X	Y	Z
Rh	0.03414000	-0.14065700	-0.74294300
C	-0.11449600	1.53476500	0.31361900
C	-0.36280100	0.80374000	-2.45644900
O	0.52188000	0.87290500	-3.27862600
C	-0.21511900	2.53200000	1.01990800
C	-0.76127900	-4.31543800	3.02189700
H	-0.49232100	-5.09017900	3.73048600
C	0.17914300	-3.34456300	2.66645500
C	-0.19551600	-2.37640700	1.72247700
C	1.55648300	-3.22724600	3.32011900
C	2.53588400	-2.75287000	2.24665500
C	2.09066400	-1.81092600	1.30657600
C	2.96528400	-1.21193700	0.38086000
C	4.30263600	-1.63677800	0.37579600

H	4.98819100	-1.21055800	-0.34879800
C	4.75553300	-2.60486300	1.26534400
H	5.79130600	-2.93006900	1.23742300
C	3.87550100	-3.14874500	2.19791200
H	4.24298500	-3.88886800	2.89954800
C	1.47904500	-2.13356600	4.42654400
H	1.17124200	-1.16794100	4.01352300
H	2.45880300	-2.00403600	4.90063100
H	0.75471000	-2.42397600	5.19627500
C	2.00398100	-4.54555800	3.97521000
H	1.30335500	-4.84543500	4.75996500
H	2.97583200	-4.42473400	4.46242300
H	2.07904600	-5.35962100	3.24656700
C	-3.40537800	-1.63808100	-0.94203600
C	-4.71100600	-1.83808800	-0.45588300
H	-4.96900900	-1.54226500	0.55555800
C	-5.69344400	-2.40223400	-1.26999100
H	-6.69612100	-2.54987000	-0.87753100
C	-5.38943000	-2.77038900	-2.58206500
H	-6.15559800	-3.20772700	-3.21695300
C	-4.09905600	-2.57251600	-3.07328300
H	-3.85278100	-2.85730600	-4.09248600
C	-3.11130900	-2.00976100	-2.26295600
H	-2.10485600	-1.88758300	-2.64412300
C	-3.00055600	0.15334000	1.30722200

C	-4.05904100	0.96679400	0.87053300
H	-4.36779500	0.94649800	-0.16863000
C	-4.73366800	1.79782900	1.76677700
H	-5.55976300	2.40904800	1.41227100
C	-4.35415200	1.84037400	3.10938500
H	-4.87908400	2.48753000	3.80674600
C	-3.29269600	1.04837800	3.54912000
H	-2.98338600	1.07905100	4.59018000
C	-2.62206800	0.21173300	2.65688600
H	-1.80269500	-0.39913500	3.02017400
C	3.45722300	-0.17766600	-2.30801700
C	4.69968700	0.46520800	-2.44898300
H	5.04982700	1.15379500	-1.68751400
C	5.49401500	0.23272400	-3.57311500
H	6.45179600	0.73802600	-3.66651900
C	5.05578400	-0.63686400	-4.57246300
H	5.67247300	-0.81465400	-5.44981000
C	3.81917000	-1.27116300	-4.44209500
H	3.46648400	-1.94416200	-5.21905200
C	3.01978900	-1.04456100	-3.32142200
H	2.05993400	-1.53788400	-3.22944200
C	3.11415700	1.66326900	-0.13950500
C	2.82067900	2.84449700	-0.84240900
H	2.17059600	2.80876000	-1.71176600
C	3.36010200	4.06110000	-0.43146500

H	3.12639000	4.96680400	-0.98403600
C	4.18953600	4.11864300	0.69280900
H	4.60827900	5.06911000	1.01266000
C	4.47006700	2.95353300	1.40634500
H	5.10970000	2.99042000	2.28443800
C	3.93462300	1.72912700	0.99307800
H	4.16661200	0.82879800	1.55228900
O	0.74670700	-1.43980900	1.29943900
P	-2.07284900	-0.91221100	0.11291800
P	2.41490400	0.09391800	-0.80556400
Cl	0.24492100	-2.39676200	-1.79387100
C	-1.50401900	-2.29971200	1.21010400
H	-3.42309200	-3.27180300	1.21009700
C	-2.04680900	-4.29977300	2.48757700
C	-2.41558100	-3.28707300	1.60880800
H	-2.76402800	-5.06587100	2.76694000
C	-0.32918700	3.68889300	1.85077300
C	0.79471100	4.21473700	2.52287100
C	-1.56990300	4.33916400	2.02207400
C	0.67807500	5.34434700	3.33006800
H	1.75556400	3.72655000	2.39876800
C	-1.67805500	5.47081300	2.82709700
H	-2.44570400	3.93820800	1.52161800
C	-0.55611000	5.98077200	3.48579600
H	1.55744500	5.73249300	3.83872000

H	-2.64396300	5.95696000	2.94261000
H	-0.64298800	6.86369000	4.11379500
C	-1.72685700	1.34441300	-2.79842700
C	-2.47473800	2.14069400	-1.92539600
C	-2.19673900	1.10925500	-4.10191400
C	-3.67947300	2.70145600	-2.35762900
H	-2.09236300	2.34124600	-0.93039300
C	-3.41716200	1.63930900	-4.51280800
H	-1.59438300	0.51413900	-4.78097600
C	-4.15958100	2.44315100	-3.64248600
H	-4.24260300	3.34311900	-1.68465000
H	-3.78277700	1.43872900	-5.51644200
H	-5.10399300	2.87118000	-3.96853400

TS2-A

Cartesian coordinates

ATOM	X	Y	Z
Rh	0.75234500	-0.46559200	-1.27373800
C	0.65825600	-0.64427400	-3.14753400
O	0.39186000	-0.96842700	-4.23159000
C	2.05440300	0.62320000	-2.81077400
C	-2.79324300	1.82021100	3.07124100
C	-3.22628800	0.81670000	1.99203200
C	-2.24618900	0.02001800	1.38942300
C	-2.52053400	-0.93939600	0.40677400

C	-3.86486700	-1.14646600	0.06803600
H	-4.12617500	-1.88890600	-0.67665500
C	-4.86962200	-0.38560900	0.66385100
H	-5.90657200	-0.55141300	0.38645400
C	-4.55103700	0.59640100	1.60213100
H	-5.34910300	1.18907000	2.03606400
C	-2.72320800	1.07404500	4.43460800
H	-2.01620800	0.23939000	4.39309600
H	-3.70908600	0.67634100	4.70223400
H	-2.39689500	1.75813900	5.22655700
C	-3.78735300	2.98953100	3.19124700
H	-3.48114800	3.67689300	3.98578200
H	-4.78379100	2.62873000	3.46369700
H	-3.86303600	3.55055000	2.25419500
C	3.44165300	1.16666200	0.69260400
C	3.52992500	2.28257500	-0.15746000
H	2.63784600	2.67140200	-0.63800400
C	4.75884000	2.89607700	-0.39775400
H	4.80607400	3.75927900	-1.05575200
C	5.92256300	2.39146900	0.18724700
H	6.88122700	2.86565100	-0.00624100
C	5.84819800	1.27069800	1.01424700
H	6.74885800	0.86591600	1.46837100
C	4.61666900	0.66307300	1.26944400
H	4.57472700	-0.20397000	1.91941300

C	2.11661900	-0.76238600	2.39622700
C	2.15689900	-0.27601600	3.71454800
H	1.97228200	0.77470400	3.91375600
C	2.43521900	-1.13384500	4.77826600
H	2.46171200	-0.74201400	5.79194900
C	2.68031000	-2.48858200	4.54132500
H	2.89666600	-3.15672300	5.37114300
C	2.64663900	-2.97771200	3.23570300
H	2.83120500	-4.03018300	3.04008100
C	2.36509200	-2.12311000	2.16637300
H	2.34584700	-2.51045300	1.15388400
C	-2.01315300	-2.57319100	-1.87856700
C	-2.33570500	-3.93429600	-1.97276200
H	-2.05329700	-4.62008000	-1.18231300
C	-3.02767200	-4.42163900	-3.08474900
H	-3.26407200	-5.48068300	-3.14481600
C	-3.41546500	-3.55703200	-4.10777400
H	-3.95211600	-3.93900700	-4.97208200
C	-3.10978800	-2.19704300	-4.01561600
H	-3.40650700	-1.51483700	-4.80751300
C	-2.41038300	-1.70856200	-2.91358700
H	-2.17893600	-0.64946000	-2.84931200
C	-0.80878000	-3.26480500	0.69401800
C	0.03973000	-4.30519200	0.27434000
H	0.53167700	-4.24790600	-0.68913400

C	0.27006200	-5.40448000	1.10189900
H	0.92307800	-6.20229200	0.75829100
C	-0.32539400	-5.47654800	2.36321200
H	-0.14134400	-6.33278400	3.00694300
C	-1.15768300	-4.44260000	2.79205800
H	-1.62516200	-4.48671600	3.77208900
C	-1.40240300	-3.34657300	1.96299500
H	-2.06443200	-2.56074200	2.30832900
O	-0.93004100	0.15308300	1.76328600
P	1.79543700	0.37473000	0.96679600
P	-1.12960300	-1.83428200	-0.42991900
C	0.82934000	1.71399900	1.79922200
H	2.33583100	3.24876300	1.95095900
C	0.45523600	3.90616000	2.76585200
C	1.30539300	2.98075100	2.15595000
H	0.83344000	4.88856500	3.03436500
C	-0.87282500	3.57651400	3.04204300
H	-1.51022500	4.31261800	3.52029300
C	-1.38214300	2.31260400	2.72155100
C	-0.50538900	1.41980400	2.10147400
C	-1.35432500	1.96203200	-1.32200700
Cl	2.29689600	-2.43278200	-1.33193200
C	-2.30441500	3.03007700	-1.33661500
C	-2.05563800	4.23055600	-0.63784200
C	-3.51824200	2.91077600	-2.04530300

C	-2.98773000	5.26678400	-0.64959900
H	-1.12678100	4.33345600	-0.08544700
C	-4.44482300	3.95086100	-2.05371500
H	-3.72335200	1.99037600	-2.58385000
C	-4.18610900	5.13401400	-1.35667000
H	-2.77560800	6.18492400	-0.10701600
H	-5.37367400	3.83756000	-2.60727300
H	-4.90968400	5.94485600	-1.36610000
C	-0.53865700	1.04960500	-1.27652000
C	3.36212600	0.16502300	-3.02994900
C	1.68778000	1.90964000	-3.24131300
C	4.29452100	0.99463400	-3.65583600
H	3.64086000	-0.83259100	-2.71206800
C	2.62587100	2.73217300	-3.86574700
H	0.67282200	2.25952800	-3.08978600
C	3.93078000	2.27644700	-4.07378200
H	5.30768000	0.63499800	-3.81641100
H	2.33367600	3.72575700	-4.19701300
H	4.65851700	2.91575600	-4.56759800

INT3-A

Cartesian coordinates

ATOM	X	Y	Z
Rh	-0.78068800	0.50883700	-1.35331200
C	-0.26880600	0.87871100	-3.14704400

O	-0.05074300	1.08274500	-4.24997200
C	-2.35570900	-0.44264200	-2.41480600
C	2.41970600	-2.28173800	3.05545700
C	3.03901500	-1.32646100	2.02602800
C	2.21666800	-0.38306300	1.39942000
C	2.68300700	0.55994400	0.47488400
C	4.06133800	0.58464700	0.21886600
H	4.46641000	1.30786300	-0.47966700
C	4.91242500	-0.33001700	0.83609300
H	5.97731200	-0.30547300	0.62364500
C	4.40232500	-1.28382600	1.71741900
H	5.08277500	-1.99411100	2.17459900
C	2.43365600	-1.57884300	4.44382600
H	1.87315700	-0.63870600	4.41788700
H	3.46292900	-1.35509900	4.74754400
H	1.97932100	-2.22575900	5.20339100
C	3.21222300	-3.59905800	3.15276700
H	2.78004200	-4.25694800	3.91273900
H	4.24477200	-3.41119500	3.46140000
H	3.22428000	-4.13107900	2.19609100
C	-3.65204500	-0.57962700	0.73001400
C	-4.03785000	-1.72049000	0.00484700
H	-3.29041400	-2.33023600	-0.49024800
C	-5.38317300	-2.06828500	-0.10616400
H	-5.66085200	-2.95286600	-0.67207700

C	-6.36634500	-1.27117400	0.48406000
H	-7.41550600	-1.53854100	0.38946600
C	-5.99524100	-0.12471600	1.18601300
H	-6.75279100	0.50704500	1.64201000
C	-4.64739000	0.21867000	1.31293700
H	-4.37645600	1.10976700	1.86754800
C	-1.86667300	1.09939200	2.25379200
C	-1.90533100	0.65886700	3.58843000
H	-1.89316200	-0.40218800	3.81412800
C	-1.96500400	1.57774400	4.63527400
H	-1.99275800	1.22091300	5.66165200
C	-1.99120300	2.94825800	4.36577700
H	-2.03634800	3.66392200	5.18269500
C	-1.96049500	3.39152600	3.04441500
H	-1.97476900	4.45461100	2.82277600
C	-1.89791900	2.47545500	1.99056600
H	-1.88184500	2.83015400	0.96613600
C	2.48953600	2.19873900	-1.84341700
C	2.90745600	3.51794000	-2.06325000
H	2.65453200	4.29649600	-1.35233800
C	3.65684600	3.84420700	-3.19739700
H	3.96854300	4.87355700	-3.35353900
C	4.00658600	2.85856600	-4.11946900
H	4.58956700	3.11499500	-4.99991400
C	3.60156200	1.53854800	-3.90482900

H	3.86818100	0.76249300	-4.61733700
C	2.84570900	1.21126600	-2.78058000
H	2.53397200	0.18157900	-2.62308400
C	1.43639400	3.16744300	0.69748000
C	0.67583600	4.28150500	0.29882700
H	0.08700000	4.23642300	-0.61096300
C	0.65379300	5.43734200	1.08069200
H	0.06806200	6.29210700	0.75263100
C	1.36946600	5.49304000	2.27874700
H	1.34753800	6.39291100	2.88800600
C	2.11128000	4.38526300	2.69021700
H	2.66895700	4.41642200	3.62259200
C	2.14990500	3.23214000	1.90409600
H	2.74244500	2.38556800	2.23332500
O	0.87329900	-0.33457600	1.68777500
P	-1.86627300	-0.12569100	0.86238400
P	1.47314500	1.66822200	-0.38410700
C	-1.11051900	-1.59695000	1.69297400
H	-2.85187200	-2.85875100	1.85377100
C	-1.11613400	-3.80489400	2.69576800
C	-1.79306300	-2.76163500	2.06102500
H	-1.65554400	-4.70639500	2.97185700
C	0.24269700	-3.69227000	2.98863500
H	0.74304800	-4.51404200	3.48952100
C	0.95765100	-2.53338800	2.66345800

C	0.25141600	-1.51645600	2.01588800
C	0.98112100	-2.17905300	-1.37462400
Cl	-1.87912000	2.76613700	-1.47986000
C	1.77782300	-3.36590000	-1.39188400
C	1.39642300	-4.50248700	-0.64741800
C	2.96243200	-3.43648100	-2.15403700
C	2.17302100	-5.65970900	-0.66654900
H	0.48597500	-4.46192400	-0.05787200
C	3.73386900	-4.59634800	-2.16894100
H	3.26805200	-2.57024000	-2.73339300
C	3.34527400	-5.71385400	-1.42576400
H	1.85837600	-6.52599500	-0.08956200
H	4.64274900	-4.62856900	-2.76465100
H	3.94745000	-6.61840100	-1.44088000
C	0.28281700	-1.17452600	-1.33112500
C	-3.57042600	0.22778700	-2.60706200
C	-2.18408800	-1.70591100	-2.99905600
C	-4.59948700	-0.36703100	-3.34458800
H	-3.72261600	1.21422600	-2.18841400
C	-3.22020300	-2.29801900	-3.73082600
H	-1.24859700	-2.23953400	-2.88493500
C	-4.43317200	-1.63245600	-3.90750600
H	-5.53515700	0.17187000	-3.47589500
H	-3.06446700	-3.28095700	-4.17062000
H	-5.23384800	-2.08935200	-4.48412400

INT4-A

Cartesian coordinates

ATOM	X	Y	Z
Rh	-0.60203300	0.98838100	0.03166500
C	-0.56707900	0.54680300	-1.78643600
O	-0.61862300	0.27272400	-2.89969100
C	-0.66204200	2.99852600	-0.54201500
C	-3.79094200	1.17774600	-0.33342400
C	-0.85913500	-3.91153500	-1.37498400
C	2.52606200	-0.65105100	-1.73060600
C	3.03113000	-1.15076600	-2.93399000
C	3.71676400	-0.24671500	-3.75454300
H	4.11822100	-0.57682300	-4.70659700
C	3.89879700	1.08115800	-3.36595000
C	3.42524400	1.53179400	-2.13225200
C	2.73243100	0.66225900	-1.27998600
H	-0.95708200	-5.42066100	-2.90395900
H	1.11443600	-4.69700400	-4.02396000
H	-1.78978800	-4.22147900	-0.91449400
H	4.42602200	1.76702200	-4.02260100
H	3.58800300	2.56158500	-1.83484700
O	1.77607100	-1.45535300	-0.89834000
C	2.87970100	-2.65170200	-3.21469700
C	2.98134400	-2.96461200	-4.71849500

H	2.90713200	-4.04104300	-4.89859200
H	2.19434600	-2.46263900	-5.29091100
H	3.95212500	-2.65200600	-5.11480800
C	4.02440700	-3.39838200	-2.47009800
H	3.98857800	-3.20631300	-1.39299100
H	3.93731000	-4.47972300	-2.62644100
H	5.00068500	-3.06948700	-2.84477700
P	-0.81209300	-1.76214500	0.53709000
P	1.95866300	1.12387300	0.33319200
C	-2.56270600	-2.35941500	0.62394200
C	-3.06085300	-3.10851700	1.70009200
C	-3.42666700	-2.05383100	-0.44187300
C	-4.39013500	-3.53879200	1.71044700
H	-2.41711800	-3.36194600	2.53427300
C	-4.74980300	-2.49252800	-0.43353400
H	-3.07111500	-1.46482900	-1.27922000
C	-5.23698600	-3.23381000	0.64538300
H	-4.75807400	-4.11643400	2.55439600
H	-5.40321900	-2.23798700	-1.26285100
H	-6.27137400	-3.56691300	0.65558900
C	-0.06432300	-2.48265900	2.06009300
C	-0.38663000	-1.89237900	3.29489000
C	0.74890000	-3.62458800	2.04016300
C	0.07174900	-2.45974500	4.48428000
H	-0.98761700	-0.98853100	3.32187200

C	1.22255400	-4.17462700	3.23287900
H	1.00538200	-4.09481400	1.09631500
C	0.87699000	-3.60064500	4.45707100
H	-0.19559900	-2.00163700	5.43293900
H	1.85344300	-5.05916500	3.20271600
H	1.23599900	-4.03824100	5.38502300
C	2.85450500	0.05744600	1.55715800
C	2.41447400	0.07579400	2.88887600
C	4.02047000	-0.65209500	1.23286800
C	3.13446000	-0.60066700	3.87454400
H	1.50923100	0.61540700	3.14883800
C	4.73286400	-1.33146500	2.22216100
H	4.38655900	-0.67052300	0.21146300
C	4.29337200	-1.30516200	3.54643900
H	2.78068800	-0.58005800	4.90125300
H	5.63500500	-1.87630200	1.95544900
H	4.85127100	-1.83022500	4.31737800
C	2.71553800	2.75268900	0.78054400
C	1.92626700	3.77416900	1.32461900
C	4.10827800	2.93660100	0.70181500
C	2.51481800	4.96844900	1.74899800
H	0.85861000	3.63475200	1.43000000
C	4.68957400	4.13355500	1.11595800
H	4.74515000	2.13946200	0.33040600
C	3.89153500	5.15575200	1.63716800

H	1.88713500	5.75052000	2.16674800
H	5.76623600	4.26279300	1.04244600
H	4.34545900	6.08759900	1.96427400
C	-0.39381700	-4.58216300	-2.50479100
Cl	-0.85628100	1.54440500	2.39577000
C	0.78224700	-4.17402400	-3.13389600
C	1.53842800	-3.11241300	-2.62665500
C	1.05496300	-2.47945400	-1.47490200
C	-0.15329800	-2.82003200	-0.84561000
C	-5.20613700	1.29573200	-0.47916400
C	-5.77543900	1.70671300	-1.70252400
C	-6.07077100	1.00005500	0.59552200
C	-7.15710200	1.81811900	-1.84259200
H	-5.11792300	1.94089000	-2.53470000
C	-7.45129700	1.11517100	0.44858500
H	-5.64086500	0.68238500	1.54035700
C	-8.00208100	1.52346100	-0.76925800
H	-7.57632700	2.13884200	-2.79313500
H	-8.10115100	0.88558300	1.28950400
H	-9.07959500	1.61290400	-0.88034500
C	-2.58585500	1.05524400	-0.17737300
C	0.10792600	3.46799600	-1.61273900
C	-1.49515300	3.89618200	0.13762100
C	0.07306700	4.81918300	-1.97978200
H	0.76064700	2.80117400	-2.16886900

C	-1.52221500	5.24468000	-0.22974200
H	-2.11128100	3.55049700	0.95749000
C	-0.73946400	5.71398500	-1.28710500
H	0.68620000	5.16237300	-2.81014800
H	-2.16976800	5.92826600	0.31480800
H	-0.76944600	6.76289900	-1.57087900

TS3-A

Cartesian coordinates

ATOM	X	Y	Z
Rh	-0.73199000	0.78565800	0.04913000
C	-2.71012500	0.98703100	-0.23479500
C	-3.92203100	0.80741400	-0.34316000
C	-1.74514900	2.67123500	-0.42712500
C	3.50305900	-2.22601700	-3.07353300
C	3.47505600	-0.72496500	-2.75525000
C	2.79473800	-0.30228600	-1.60944700
C	2.77918100	1.02513600	-1.15508100
C	3.49066000	1.96978900	-1.90535300
H	3.50916500	3.00590100	-1.58654400
C	4.16228700	1.58797300	-3.06752800
H	4.70272900	2.33163600	-3.64605600
C	4.15355800	0.25716900	-3.48667600
H	4.69167200	-0.01467000	-4.38834100
C	4.64741700	-2.87618800	-2.24288000

H	4.50414100	-2.70801200	-1.17077300
H	5.61572700	-2.44943800	-2.52918700
H	4.67748700	-3.95781200	-2.41839700
C	3.76723600	-2.48801200	-4.56704700
H	3.82181700	-3.56168800	-4.76943600
H	4.73040700	-2.06697500	-4.87016700
H	2.98488200	-2.05501800	-5.19910300
C	-2.08142300	-2.76984200	0.56837400
C	-3.07459400	-2.40753400	-0.35649400
H	-2.88579000	-1.61687700	-1.07258200
C	-4.31698100	-3.04271600	-0.35169800
H	-5.07714300	-2.73543900	-1.06401000
C	-4.58744800	-4.04571200	0.58040900
H	-5.55836900	-4.53396700	0.59034700
C	-3.60738100	-4.41438200	1.50353700
H	-3.80849300	-5.19634500	2.23123000
C	-2.36210400	-3.78323500	1.49894600
H	-1.61143500	-4.08361000	2.22125100
C	0.44500200	-2.54256000	1.95870200
C	1.55161200	-3.39916300	1.86683900
H	1.92572500	-3.70629700	0.89603400
C	2.17273800	-3.88059500	3.02096200
H	3.02867500	-4.54452100	2.93164600
C	1.69182900	-3.52091900	4.28016000
H	2.17110000	-3.90379500	5.17746800

C	0.59176200	-2.66623500	4.38082500
H	0.21138400	-2.37728200	5.35711600
C	-0.02347200	-2.17008600	3.23158200
H	-0.86219900	-1.48809600	3.31962500
C	2.06489200	3.22572600	0.56907800
C	2.92070000	3.74675400	1.55225700
H	3.44219800	3.08042600	2.22995200
C	3.10892800	5.12525700	1.66803500
H	3.77203000	5.51189800	2.43751200
C	2.45306900	6.00078500	0.80167000
H	2.60076400	7.07360200	0.89428400
C	1.60011000	5.49181300	-0.17930900
H	1.07071400	6.16210000	-0.85007200
C	1.39914600	4.11562600	-0.28993900
H	0.71405000	3.73884500	-1.04158400
C	2.82876600	0.68356000	1.70519700
C	2.30272600	0.59655200	3.00396600
H	1.27490500	0.88953000	3.19012100
C	3.09558100	0.12654100	4.05273000
H	2.67343500	0.06265300	5.05168700
C	4.41385000	-0.26806900	3.82162700
H	5.02653900	-0.63619500	4.64054000
C	4.94218800	-0.18766300	2.53203900
H	5.96884800	-0.48979200	2.34127300
C	4.15755600	0.28861200	1.48100800

H	4.58938800	0.36138200	0.48844200
O	2.09105400	-1.20779200	-0.84305100
P	-0.44969700	-1.90693200	0.47469200
P	1.78552100	1.41040200	0.36088900
C	0.35410300	-2.80389000	-0.93841200
H	-1.11952500	-4.35928100	-1.16172900
C	0.39272200	-4.49357400	-2.68329300
C	-0.20476700	-3.93188700	-1.55591200
H	-0.05640700	-5.36290000	-3.15487300
C	1.56222600	-3.94574600	-3.21148100
H	2.00388700	-4.39880000	-4.09278100
C	2.17046000	-2.83524000	-2.61602100
C	1.54286800	-2.29795300	-1.48611900
C	-0.44564600	0.42683700	-1.74888800
Cl	-1.14305300	1.04024600	2.45008500
C	-5.32521400	0.63254500	-0.45106300
C	-5.94060500	0.44985300	-1.71050600
C	-6.14349700	0.63172000	0.70144900
C	-7.31811100	0.27202400	-1.80853900
H	-5.32206800	0.45455500	-2.60344600
C	-7.51973000	0.45642200	0.59226500
H	-5.67802600	0.76921600	1.67247000
C	-8.11509700	0.27451100	-0.65997000
H	-7.77345500	0.13458700	-2.78620700
H	-8.13303200	0.46017000	1.48982800

H	-9.19014300	0.13732800	-0.74010100
O	-0.29363400	0.15471100	-2.85803100
C	-1.80075500	3.12938000	-1.76067900
C	-1.93639400	3.60521000	0.60703000
C	-2.02611600	4.47588900	-2.04533500
H	-1.70869600	2.43259600	-2.58674400
C	-2.16658500	4.94911100	0.31017800
H	-1.91374200	3.27516300	1.63727400
C	-2.20972400	5.39800100	-1.01149900
H	-2.06991200	4.79894000	-3.08306500
H	-2.31237000	5.65040200	1.12855600
H	-2.39458800	6.44565900	-1.23379200

INT5-A

Cartesian coordinates

ATOM	X	Y	Z
Rh	-0.98438000	0.37930800	-0.05501400
C	-2.66664100	1.67825200	-0.55053700
C	-3.10980000	0.49081600	-0.53393200
C	-2.92790200	3.10039100	-0.60516800
O	-0.36765800	0.12438400	-2.97958600
C	0.79362400	-3.99716800	-1.56909000
C	2.83370700	0.23690200	-1.43176200
C	3.70798600	-0.01809600	-2.49304300
C	4.19633900	1.09050200	-3.19375600

H	4.87021700	0.94876000	-4.03195400
C	3.83963400	2.38683800	-2.82153300
C	2.98441900	2.60417300	-1.74053000
C	2.45014300	1.52344300	-1.02665500
H	1.43865900	-5.35912700	-3.10311100
H	3.30040500	-3.93619000	-3.85943700
H	-0.02771800	-4.63193300	-1.25856400
H	4.23763400	3.23456000	-3.37198600
H	2.72197900	3.61722000	-1.45813200
O	2.30221200	-0.80824300	-0.70489200
C	4.13225700	-1.46974300	-2.74876800
C	4.62333300	-1.67806900	-4.19196200
H	4.95275200	-2.71006700	-4.34510700
H	3.84040400	-1.45156700	-4.92332800
H	5.48858300	-1.04259500	-4.40269400
C	5.29368200	-1.81378200	-1.77141000
H	4.98953500	-1.67276300	-0.72952400
H	5.60266100	-2.85763700	-1.90012200
H	6.15789100	-1.16761900	-1.96456200
P	-0.13492400	-2.08434500	0.37672700
P	1.25594700	1.66642300	0.38743700
C	-1.49503000	-3.34003000	0.30527900
C	-1.60558600	-4.40613600	1.21023400
C	-2.42017800	-3.24927000	-0.74645900
C	-2.61972600	-5.35596000	1.06538300

H	-0.89920900	-4.50057700	2.02735300
C	-3.42251300	-4.20624900	-0.89929200
H	-2.35967000	-2.42282300	-1.44728000
C	-3.52722800	-5.26122600	0.00978100
H	-2.69375200	-6.17322200	1.77824200
H	-4.13403700	-4.11080600	-1.71384500
H	-4.31502800	-6.00168500	-0.10151300
C	0.72685600	-2.50772400	1.95573900
C	0.06984800	-2.23219100	3.16827900
C	1.99183200	-3.11221700	1.99150800
C	0.66211900	-2.57588500	4.38373500
H	-0.89443900	-1.73536200	3.15997400
C	2.58707200	-3.43855900	3.21173400
H	2.51380300	-3.34140100	1.06836900
C	1.92213000	-3.17799100	4.41019200
H	0.13756700	-2.36403900	5.31186600
H	3.56873400	-3.90514700	3.22148300
H	2.38302500	-3.44120500	5.35877600
C	2.34494800	1.27382300	1.83283300
C	1.75214000	0.94266600	3.06070400
C	3.74527700	1.35324000	1.75088500
C	2.54853100	0.70153600	4.18256700
H	0.67169200	0.86972600	3.13341400
C	4.53556700	1.10373100	2.87327000
H	4.22342800	1.61585600	0.81268300

C	3.93868100	0.77910000	4.09324400
H	2.07525500	0.44203300	5.12532400
H	5.61791300	1.16735900	2.79338600
H	4.55497700	0.58646200	4.96778700
C	1.05971000	3.50082000	0.51595900
C	0.32585800	4.16625400	-0.47955400
C	1.62009400	4.25426100	1.55803400
C	0.18184800	5.55268600	-0.45205400
H	-0.14041400	3.59826600	-1.27853800
C	1.45828400	5.64131000	1.59445100
H	2.18589900	3.76289500	2.34166200
C	0.74675300	6.29444300	0.58787700
H	-0.39314000	6.04603300	-1.22976400
H	1.89546800	6.20958300	2.41140700
H	0.62535200	7.37414000	0.61683000
C	1.61971900	-4.40586400	-2.61478600
Cl	-1.69440200	0.65472100	2.30377800
C	2.67541900	-3.59869600	-3.03934300
C	2.93951800	-2.37534400	-2.41529200
C	2.08853700	-1.99582500	-1.36952500
C	1.00079900	-2.76521800	-0.93053600
C	-4.24439400	-0.40648600	-0.57333900
C	-4.91614300	-0.66107300	-1.78371000
C	-4.72804900	-0.99015200	0.61316000
C	-6.05256100	-1.46915800	-1.80342800

H	-4.54713800	-0.21040200	-2.70086000
C	-5.86748500	-1.79057700	0.58531900
H	-4.20172700	-0.79298700	1.54185600
C	-6.53347500	-2.03451600	-0.61948000
H	-6.56814800	-1.64941300	-2.74346400
H	-6.23740500	-2.22786900	1.50898500
H	-7.42270100	-2.65936900	-0.63547700
C	-0.58752200	0.21900800	-1.84890400
C	-3.14418100	3.74780000	-1.83599300
C	-3.02992800	3.84380800	0.58642500
C	-3.47147300	5.10300900	-1.87227000
H	-3.06697800	3.17742900	-2.75758100
C	-3.36416400	5.19522000	0.54107000
H	-2.84895000	3.34042500	1.53095100
C	-3.58712200	5.83018400	-0.68446700
H	-3.64668100	5.58853600	-2.82909500
H	-3.44992900	5.75658600	1.46772200
H	-3.84994300	6.88446900	-0.71388900

TS1-B

Cartesian coordinates

ATOM	X	Y	Z
Rh	-0.06824300	-0.39694200	0.07015800
C	0.51534900	-0.82633100	2.18528500
C	0.85250000	-2.06185400	0.83412000

O	0.22827500	-3.12196100	0.94391500
C	3.41539800	-2.28527300	0.13052000
C	-1.49734100	4.93960500	-1.72081700
H	-2.12879700	5.74863200	-2.07060800
C	-2.07329900	3.79740600	-1.16183400
C	-1.22627200	2.75814200	-0.74119000
C	-3.57525500	3.63138400	-0.92516800
C	-3.91087200	2.17113500	-1.23045900
C	-3.00442100	1.18930700	-0.79963800
C	-3.28068900	-0.18565900	-0.91687800
C	-4.47657500	-0.55046800	-1.55323400
H	-4.70597000	-1.60056900	-1.68868200
C	-5.36335000	0.40442300	-2.03862200
H	-6.27409400	0.09464800	-2.54284300
C	-5.08452800	1.75810000	-1.86481400
H	-5.79177300	2.49614300	-2.22610200
C	-3.87497900	3.90595700	0.57808000
H	-3.30853000	3.23304600	1.22864300
H	-4.94205000	3.76200900	0.78386100
H	-3.60737200	4.93735900	0.83548500
C	-4.41038900	4.61048200	-1.76654200
H	-4.16888200	5.64574900	-1.50760100
H	-5.47744700	4.48051800	-1.56226100
H	-4.24355400	4.47346500	-2.84019100
C	2.82369800	1.62180500	-1.21848300

C	3.76612300	2.66767900	-1.21057900
H	3.66527700	3.49115600	-0.51131600
C	4.85516800	2.65352400	-2.08266900
H	5.56990700	3.47219100	-2.06232400
C	5.02480500	1.59214400	-2.97417000
H	5.87230600	1.58125100	-3.65460000
C	4.10533300	0.54322100	-2.98047200
H	4.23337400	-0.29178100	-3.66333800
C	3.01552900	0.55231000	-2.10709600
H	2.29979000	-0.25976100	-2.12642400
C	1.92735800	2.49542600	1.40711600
C	3.24404800	2.37687800	1.88228600
H	3.96840200	1.78309700	1.33406400
C	3.64465700	3.02328600	3.05252400
H	4.67227100	2.92648500	3.39345200
C	2.73208900	3.78806000	3.78066400
H	3.04317300	4.29127100	4.69220100
C	1.41644600	3.90037700	3.32849900
H	0.69556400	4.49095400	3.88761500
C	1.01887300	3.26313600	2.15290100
H	-0.00634200	3.37320800	1.81402200
C	-2.26693600	-2.95459200	-1.33608100
C	-3.40924600	-3.77402600	-1.37507600
H	-4.25419100	-3.56619700	-0.72653500
C	-3.46309300	-4.88274400	-2.22034600

H	-4.35628200	-5.50183900	-2.23853900
C	-2.36892500	-5.20090600	-3.02644100
H	-2.40741100	-6.06767000	-3.68129600
C	-1.22226500	-4.40780500	-2.97703400
H	-0.36046100	-4.65470700	-3.59110300
C	-1.16782400	-3.29387500	-2.13792500
H	-0.27813600	-2.67862600	-2.11428600
C	-3.17731000	-2.09103100	1.22733000
C	-2.88002800	-3.34285000	1.79926900
H	-2.07752800	-3.94430700	1.38777200
C	-3.58931100	-3.79774600	2.91030300
H	-3.34731800	-4.76686000	3.33838400
C	-4.59797900	-3.01299600	3.47514800
H	-5.14888900	-3.37067700	4.34108000
C	-4.89142300	-1.76583100	2.92262600
H	-5.67380000	-1.14691200	3.35422700
C	-4.18681500	-1.30693100	1.80716500
H	-4.43362800	-0.33756000	1.38722700
O	-1.80208700	1.60083100	-0.22828300
P	1.34332500	1.58582300	-0.10370700
P	-2.16735600	-1.49077000	-0.20612500
Cl	-0.29317800	-0.07973700	-2.37843200
C	0.17565000	2.86553500	-0.80205500
H	1.77887400	4.14862400	-1.44523400
C	-0.11410100	5.05869500	-1.82962600

C	0.70527800	4.03910100	-1.35891900
H	0.32501000	5.94830300	-2.27169900
C	4.76381500	-2.53503400	-0.26073400
C	5.70612900	-1.49026500	-0.34254000
C	5.17546600	-3.84906200	-0.56567100
C	7.02159800	-1.75696300	-0.71408800
H	5.39253500	-0.47543100	-0.12121300
C	6.49155300	-4.10553100	-0.94059600
H	4.45133600	-4.65526300	-0.50201700
C	7.41901500	-3.06246700	-1.01445700
H	7.73835000	-0.94228300	-0.77329700
H	6.79561500	-5.12236900	-1.17392100
H	8.44602800	-3.26617400	-1.30542100
C	2.26206100	-2.10562500	0.47807500
C	-0.56310800	-1.02838200	3.06955800
C	1.72553400	-0.37821300	2.74750100
C	-0.45806000	-0.72932500	4.42629500
H	-1.49095300	-1.43706300	2.70355600
C	1.84932200	-0.11245900	4.11074900
H	2.59890700	-0.27510700	2.11536500
C	0.75128900	-0.27079100	4.95559200
H	-1.31949800	-0.87415100	5.07356800
H	2.80324300	0.22448300	4.50677500
H	0.84072300	-0.05865600	6.01811400

INT2-B

Cartesian coordinates

ATOM	X	Y	Z
Rh	0.15840900	0.42209300	-0.19765800
C	0.11481300	0.68252800	1.86220600
C	-0.81326600	2.11073600	-0.45667900
O	-0.23288700	3.17755100	-0.57402700
C	-3.46744800	2.20948900	-0.51259000
C	0.95327100	-5.11447900	-1.14788000
H	1.50100500	-6.01995700	-1.38210600
C	1.64480800	-3.97562100	-0.72452200
C	0.90391000	-2.81393800	-0.46039900
C	3.14895400	-3.96477800	-0.45033400
C	3.68021600	-2.58251300	-0.82924800
C	2.88060200	-1.45995200	-0.57008600
C	3.35847000	-0.14744400	-0.74242700
C	4.65369800	0.01364500	-1.25575300
H	5.03309900	1.01612100	-1.42249100
C	5.44644400	-1.08438700	-1.57135300
H	6.44161800	-0.93971300	-1.98134500
C	4.96133000	-2.37029700	-1.34810800
H	5.59547000	-3.21908100	-1.57661200
C	3.36574200	-4.16915300	1.07860900
H	2.86351100	-3.39131400	1.66207500
H	4.43488800	-4.13738000	1.31809700

H	2.96639200	-5.14078500	1.39151600
C	3.88299800	-5.09419300	-1.19465500
H	3.50746900	-6.07210500	-0.87992700
H	4.95028800	-5.08559500	-0.95608800
H	3.76855700	-5.01000400	-2.28043400
C	-3.01674100	-1.46894300	-1.12680200
C	-4.15156700	-2.17299900	-0.68594600
H	-4.17463100	-2.60300000	0.30991500
C	-5.25846300	-2.32921000	-1.52387900
H	-6.12457800	-2.88321900	-1.17078700
C	-5.25101800	-1.77558500	-2.80565600
H	-6.11303000	-1.89536900	-3.45681100
C	-4.13225400	-1.06499700	-3.24426300
H	-4.11933200	-0.62640800	-4.23828400
C	-3.02022500	-0.90930500	-2.41484300
H	-2.14708000	-0.37179700	-2.76885000
C	-2.17763400	-1.72873300	1.61158100
C	-2.95002800	-0.75353600	2.26529900
H	-3.11089900	0.21334800	1.79854500
C	-3.49999700	-1.01178400	3.51907500
H	-4.09116300	-0.24604700	4.01361300
C	-3.28078900	-2.24256500	4.14321100
H	-3.70585500	-2.44045700	5.12366100
C	-2.50823000	-3.21269200	3.50574300
H	-2.32898900	-4.17123400	3.98588200

C	-1.95888400	-2.95913000	2.24601800
H	-1.36340500	-3.72512700	1.76081800
C	2.86518400	2.63635400	-1.52120300
C	3.96148100	3.47520800	-1.25484500
H	4.50626200	3.38419200	-0.32102100
C	4.35505800	4.44189400	-2.18161800
H	5.20456100	5.08288200	-1.96038200
C	3.65514600	4.58863300	-3.37994000
H	3.95830800	5.34503800	-4.09942000
C	2.56091000	3.76406100	-3.64636400
H	2.00603500	3.87576800	-4.57398900
C	2.16260400	2.79426500	-2.72562600
H	1.31565300	2.15401100	-2.94170000
C	3.09584500	1.96182400	1.25807800
C	2.53024600	3.11576700	1.82770600
H	1.67910500	3.59127900	1.34978000
C	3.05341600	3.64699000	3.00447500
H	2.60647500	4.53867700	3.43555400
C	4.14065100	3.03318700	3.63267200
H	4.54478000	3.44841600	4.55219600
C	4.70141000	1.88379300	3.07734300
H	5.54555500	1.39859500	3.56058200
C	4.18226200	1.34895300	1.89503800
H	4.63245900	0.45772500	1.47071100
O	1.57606100	-1.64488800	-0.09912800

P	-1.51175200	-1.30864300	-0.05829000
P	2.34929800	1.34163500	-0.30892800
Cl	0.31904000	-0.10781500	-2.68251600
C	-0.50199900	-2.79378500	-0.52372900
H	-2.23092400	-3.97082400	-1.02668700
C	-0.43339600	-5.11181500	-1.26881500
C	-1.14998200	-3.96574500	-0.94205700
H	-0.95429100	-6.00277600	-1.60684600
C	-4.87571000	2.43313000	-0.52086600
C	-5.78792000	1.35978700	-0.52479100
C	-5.36862200	3.75476000	-0.52209800
C	-7.15830700	1.60867800	-0.52785200
H	-5.41273300	0.34257700	-0.53052000
C	-6.74009800	3.99232100	-0.52300100
H	-4.66361100	4.58018600	-0.52240900
C	-7.63814300	2.92102500	-0.52578000
H	-7.85457100	0.77447500	-0.53305200
H	-7.10979300	5.01400800	-0.52295800
H	-8.70848100	3.10903700	-0.52755400
C	-2.25602100	2.08029300	-0.49920200
C	0.78912200	-0.25165300	2.66748400
C	-0.55517900	1.72412800	2.52727300
C	0.79261800	-0.15767900	4.06263200
H	1.32625200	-1.07556500	2.20604100
C	-0.55643200	1.82288000	3.92262200

H	-1.09196200	2.48421600	1.96875400
C	0.11722500	0.88239800	4.70127200
H	1.32747200	-0.90276100	4.64851500
H	-1.08876200	2.64459000	4.39830500
H	0.11773300	0.95895900	5.78582000

2. Coordinates of TS3-A versus TS3S-A

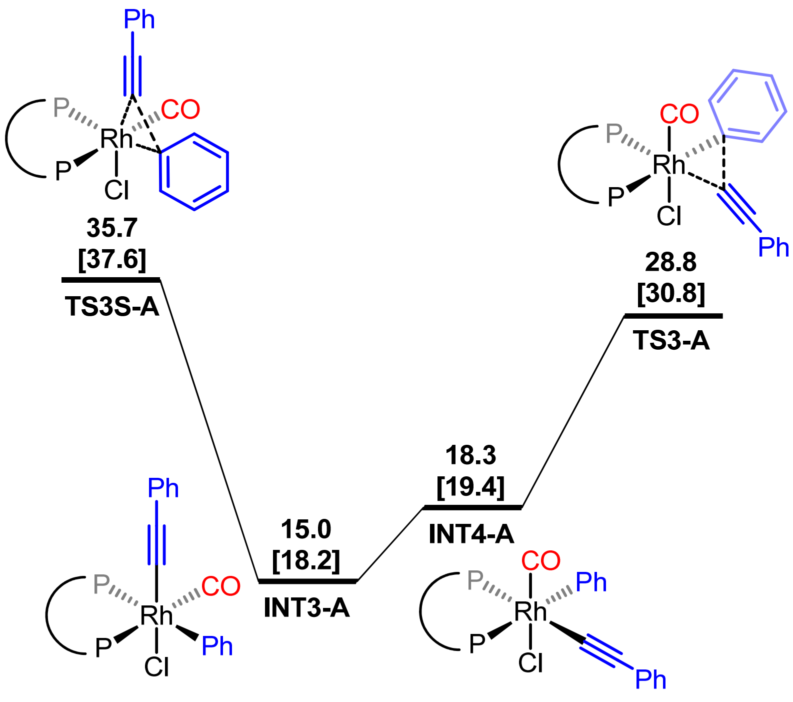

Figure S3

TS3S-A

Cartesian coordinates

ATOM	X	Y	Z
Rh	-0.59459800	-0.04460700	-1.57301600
C	0.70032200	-1.52454400	-1.19753700
C	1.69165400	-2.17296200	-0.85155500
C	-0.76489200	-2.13809000	-2.24963500
C	1.87263200	-1.14654600	3.84921400
C	2.52951300	-0.20733200	2.82693800
C	1.73396200	0.42038700	1.86058000
C	2.23168100	1.32640200	0.91164000
C	3.59508900	1.64701800	0.98664900

H	4.02062500	2.34649300	0.27589400
C	4.41372500	1.05682200	1.94767100
H	5.46832800	1.31314000	1.99139500
C	3.88694200	0.13011200	2.84708200
H	4.54653300	-0.32592500	3.57748000
C	1.41924300	-0.29599200	5.07077000
H	0.71920600	0.48935000	4.76699900
H	2.28445100	0.18351900	5.54302300
H	0.92211400	-0.92917600	5.81504100
C	2.85690700	-2.22966000	4.33186700
H	2.38669200	-2.88049900	5.07511400
H	3.72268500	-1.77815800	4.82494900
H	3.21295200	-2.84570200	3.50011000
C	-3.74332400	-1.01787700	0.10864500
C	-4.15307500	-1.25033800	-1.21111900
H	-3.47453700	-1.04578200	-2.02995800
C	-5.44370600	-1.71636000	-1.47453400
H	-5.74902900	-1.88648300	-2.50314700
C	-6.33392400	-1.95532800	-0.42829400
H	-7.33681500	-2.31883100	-0.63687800
C	-5.93878200	-1.71119200	0.89008100
H	-6.63237700	-1.87950600	1.70969800
C	-4.65610000	-1.23500700	1.15661300
H	-4.37054900	-1.01799100	2.18198100
C	-2.55293800	1.17342800	1.44378400

C	-2.40771200	1.22892600	2.83852100
H	-1.93309200	0.41581500	3.37600400
C	-2.89040300	2.32274100	3.55936900
H	-2.76944800	2.34713600	4.63939600
C	-3.52855400	3.37299500	2.89963100
H	-3.90642900	4.22286600	3.46195700
C	-3.68137700	3.32172300	1.51362200
H	-4.17708000	4.13353500	0.98844600
C	-3.19859200	2.23301700	0.78700900
H	-3.31745200	2.20681400	-0.29074900
C	2.35951100	2.48555600	-1.68697300
C	2.48927600	3.80571900	-2.14024700
H	1.87111900	4.58972800	-1.71632100
C	3.41402000	4.12589500	-3.13830400
H	3.49935900	5.15488800	-3.47773200
C	4.22516100	3.13610800	-3.69210400
H	4.94468800	3.38783200	-4.46657000
C	4.10389600	1.81678200	-3.24716400
H	4.72911000	1.03714000	-3.67457600
C	3.17581400	1.49215000	-2.25960400
H	3.08535300	0.46111000	-1.92659300
C	0.49087200	3.57783700	0.22991300
C	-0.55679900	4.19679000	-0.47335800
H	-1.00988000	3.69376400	-1.32425100
C	-1.02734700	5.44858800	-0.07282000

H	-1.83157000	5.92156800	-0.63019500
C	-0.48008700	6.08195900	1.04474600
H	-0.85368600	7.05304100	1.35934800
C	0.54443000	5.46058800	1.76190100
H	0.97044500	5.94568600	2.63648800
C	1.03288700	4.21817700	1.35457500
H	1.83939800	3.74989700	1.91011800
O	0.38112800	0.17966600	1.81161100
P	-2.06694300	-0.32100400	0.45210500
P	1.10852600	1.96678500	-0.41937400
C	-1.32019600	-1.43083900	1.72386200
H	-2.79635100	-3.00137500	1.75742800
C	-1.14901200	-3.43681400	3.07332300
C	-1.84588400	-2.65563700	2.14940800
H	-1.56032400	-4.38999500	3.39247200
C	0.06281100	-2.99234300	3.60341200
H	0.57054200	-3.60734300	4.33887200
C	0.61972300	-1.76647300	3.21564300
C	-0.08605400	-1.03052500	2.25995100
C	0.23327200	0.45181900	-3.20132200
Cl	-2.32008700	1.55409100	-2.51027200
C	2.78357500	-2.99723900	-0.47820700
C	2.67347900	-3.89807500	0.60680800
C	4.00466800	-2.95572500	-1.18918800
C	3.74252500	-4.71707000	0.96029000

H	1.73798300	-3.94619500	1.15512800
C	5.06875700	-3.77564700	-0.82529100
H	4.10023500	-2.27477400	-2.02980500
C	4.94611300	-4.65983800	0.25046400
H	3.63476600	-5.41027300	1.79109500
H	5.99889400	-3.72744400	-1.38584300
H	5.77784000	-5.30073700	0.53001700
O	0.66124900	0.80828900	-4.20314500
C	-0.53056700	-2.39447300	-3.61801200
C	-1.50973000	-3.09252500	-1.53037700
C	-1.05865200	-3.52660000	-4.23810500
H	0.07828100	-1.72570500	-4.21318300
C	-2.03043000	-4.22443000	-2.15459100
H	-1.64440300	-2.98184700	-0.46327900
C	-1.81952300	-4.44746800	-3.51640800
H	-0.86594200	-3.68507100	-5.29645600
H	-2.59915000	-4.93895500	-1.56422400
H	-2.22665500	-5.32968800	-4.00244900

3. Coordinates of TS3-deCO versus TS3-A

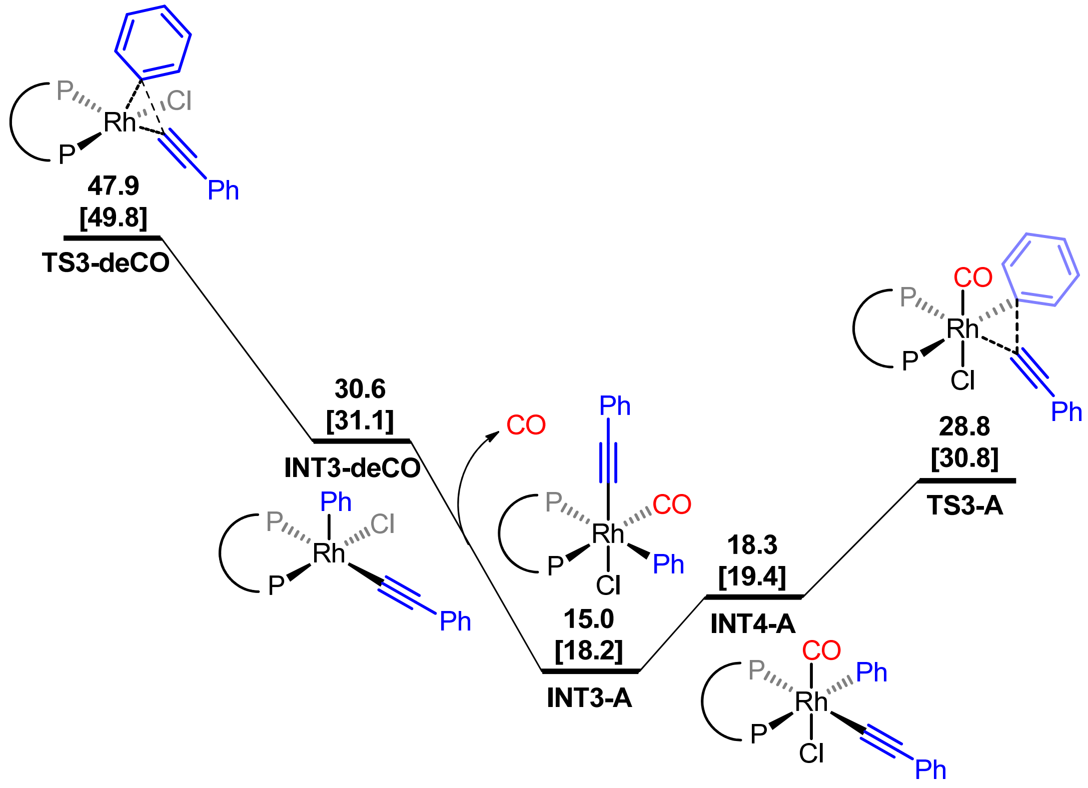

Figure S4

INT3-deCO

Cartesian coordinates

ATOM	X	Y	Z
Rh	-0.66003800	0.90159800	-0.16182900
C	-2.63396200	0.85971900	-0.10836700
C	-3.84895300	0.96616000	0.00696100
C	-0.54538800	0.82288500	-2.17118100
C	-0.09202500	-3.96222700	-1.04965800
C	3.27955200	-0.85625200	-0.24211100
C	4.31820300	-1.68691400	-0.67680200

C	5.44604500	-1.06521400	-1.22125200
H	6.27686800	-1.66383200	-1.57974900
C	5.52051900	0.32592500	-1.30661700
C	4.46722800	1.12196000	-0.85752800
C	3.30953100	0.53905200	-0.31978100
H	0.47949200	-5.81147100	-1.98240900
H	2.90189400	-5.41917000	-1.72651000
H	-1.14971800	-4.15294200	-1.17776600
H	6.40596400	0.79349900	-1.72757800
H	4.53764300	2.20027100	-0.93686800
O	2.13334600	-1.41774700	0.29218600
C	4.14814800	-3.19737100	-0.47881400
C	5.08704800	-4.00938300	-1.38587900
H	4.96228600	-5.08248800	-1.21087400
H	4.90505100	-3.80774900	-2.44685600
H	6.13323900	-3.77716300	-1.16478000
C	4.46861600	-3.54352200	1.00425600
H	3.82659900	-2.98511700	1.69213200
H	4.31700600	-4.61390000	1.18528500
H	5.51143200	-3.29629500	1.23353700
P	-0.85577000	-1.46921500	0.14587500
P	1.82779900	1.50621400	0.23899300
C	-2.43892800	-2.20301800	-0.45448800
C	-3.19384900	-3.07344500	0.34302100
C	-2.85240400	-1.94575400	-1.77123700

C	-4.34588900	-3.67781400	-0.16799400
H	-2.88946300	-3.28128800	1.36370000
C	-3.99768700	-2.55689200	-2.27955300
H	-2.28848300	-1.25868000	-2.39303700
C	-4.74809600	-3.42349200	-1.48001300
H	-4.92543800	-4.34727500	0.46221600
H	-4.31047900	-2.34504600	-3.29819100
H	-5.64356300	-3.89420900	-1.87712800
C	-0.90172200	-1.69165900	1.97860400
C	-1.76024500	-0.86542800	2.72752800
C	-0.10871400	-2.63508200	2.64846300
C	-1.81910100	-0.98821600	4.11585700
H	-2.39083700	-0.13925700	2.22195400
C	-0.16886700	-2.74852000	4.03858600
H	0.55152400	-3.28989600	2.08995700
C	-1.02187500	-1.92571800	4.77538200
H	-2.49022700	-0.34648000	4.68004100
H	0.45177900	-3.48396100	4.54365300
H	-1.06637000	-2.01595400	5.85745600
C	2.04767600	1.56814300	2.07754500
C	1.05933100	2.20888000	2.84694700
C	3.14364800	0.98575100	2.73191900
C	1.17338600	2.26302500	4.23653600
H	0.21352100	2.68036800	2.35466000
C	3.24913000	1.03813400	4.12344200

H	3.92378100	0.49535400	2.15921700
C	2.26469000	1.67532000	4.87955500
H	0.40392000	2.76691200	4.81534200
H	4.10553700	0.58238900	4.61392700
H	2.34881700	1.71614300	5.96244000
C	2.30721000	3.19073200	-0.34047800
C	1.99649800	3.55360400	-1.66036300
C	3.01286100	4.09057000	0.46895400
C	2.39697900	4.79104800	-2.16156800
H	1.42521800	2.87719800	-2.28797900
C	3.40416100	5.33388000	-0.03417300
H	3.25441300	3.82678700	1.49371200
C	3.09980000	5.68499200	-1.34968800
H	2.14517700	5.06297300	-3.18304400
H	3.94470000	6.02659600	0.60565300
H	3.40241900	6.65340600	-1.73950900
C	0.83122700	-4.90044500	-1.50717600
Cl	-0.94460000	3.29240300	0.07043100
C	2.20046000	-4.67691800	-1.36099500
C	2.67147200	-3.51370500	-0.74653500
C	1.71677400	-2.58756900	-0.30573400
C	0.33514500	-2.77042900	-0.44104300
C	-5.25789900	1.14432700	0.15391400
C	-6.14192400	0.04519300	0.16553700
C	-5.79755100	2.44119900	0.29353000

C	-7.51376600	0.24012400	0.31227400
H	-5.73746200	-0.95625600	0.05735000
C	-7.17009500	2.62747700	0.43848700
H	-5.12093800	3.29039600	0.28599100
C	-8.03502300	1.52941900	0.44898700
H	-8.18065800	-0.61881000	0.31902000
H	-7.56731600	3.63401500	0.54421600
H	-9.10588800	1.67755100	0.56242400
C	0.41847900	0.04153200	-2.81952900
C	-1.41577700	1.61518800	-2.93395900
C	0.51830300	0.05666600	-4.21686500
H	1.10415200	-0.57671200	-2.25068000
C	-1.30733000	1.62268400	-4.32867100
H	-2.16303600	2.22623000	-2.44440400
C	-0.34393900	0.84628000	-4.97688500
H	1.27617100	-0.55429200	-4.70207500
H	-1.98579300	2.24562000	-4.90706700
H	-0.26687900	0.85748000	-6.06101300

TS3-deCO

Cartesian coordinates

ATOM	X	Y	Z
Rh	0.71740800	0.76674300	-0.06725900
C	1.59067000	1.02441200	1.84428500
C	2.65975500	0.66295700	0.30355300

C	0.06102800	-3.96256100	1.29470500
C	-3.17599400	-0.63281100	0.86004100
C	-4.13670200	-1.28491600	1.64227800
C	-5.07203200	-0.48179400	2.30121500
H	-5.83337800	-0.93562900	2.92677600
C	-5.04440200	0.90627200	2.15908600
C	-4.07271700	1.51931600	1.36909400
C	-3.09934100	0.75437600	0.70739800
H	-0.39591000	-5.62554900	2.57840200
H	-2.76169200	-5.00330700	2.87831000
H	1.09853500	-4.25152100	1.18003100
H	-5.78356600	1.51571600	2.67109100
H	-4.06026400	2.59876000	1.27877600
O	-2.22810600	-1.37564200	0.18531600
C	-4.12101400	-2.81887700	1.66770000
C	-4.84955700	-3.38277900	2.89926400
H	-4.84259300	-4.47708000	2.88715200
H	-4.39068600	-3.04368400	3.83403100
H	-5.90057000	-3.07836500	2.89970200
C	-4.83182900	-3.34061600	0.38541900
H	-4.34728300	-2.96109100	-0.51934800
H	-4.80641200	-4.43605400	0.35457300
H	-5.87873300	-3.01629300	0.37412500
P	0.63593100	-1.62514900	-0.30609700
P	-1.71962100	1.49549700	-0.29839300

C	2.24053100	-2.56407400	-0.30071300
C	2.60060900	-3.39133100	-1.37564600
C	3.07884300	-2.52826800	0.82653300
C	3.76235400	-4.16544700	-1.32409200
H	1.97434400	-3.44005400	-2.25897000
C	4.22756200	-3.31859200	0.88467300
H	2.84435000	-1.88293600	1.66400300
C	4.57561000	-4.13801400	-0.19182700
H	4.02362400	-4.79489000	-2.17055900
H	4.85479300	-3.28446300	1.77165800
H	5.47439900	-4.74736500	-0.14763300
C	0.08183700	-1.82341200	-2.05527500
C	0.65430400	-0.97127200	-3.01555000
C	-0.84626300	-2.78970600	-2.46782200
C	0.30502400	-1.08935000	-4.36090000
H	1.37588700	-0.21701900	-2.71033000
C	-1.19704300	-2.90013100	-3.81465500
H	-1.29102100	-3.46344100	-1.74246300
C	-0.62289700	-2.05185500	-4.76307600
H	0.75571700	-0.42392400	-5.09173900
H	-1.91889900	-3.65247800	-4.12157700
H	-0.89819000	-2.14006000	-5.81058200
C	-2.39907500	1.44956700	-2.02060700
C	-1.60450500	1.96611600	-3.05968700
C	-3.66443200	0.92951500	-2.32859600

C	-2.07847100	1.97205800	-4.37154000
H	-0.62396300	2.37548900	-2.83225100
C	-4.12843900	0.92595700	-3.64625500
H	-4.29784100	0.53250600	-1.54209100
C	-3.33891300	1.44926900	-4.67029600
H	-1.45729100	2.38518200	-5.16207700
H	-5.11163800	0.51843500	-3.86768700
H	-3.70372800	1.45182100	-5.69427700
C	-1.91393100	3.26711900	0.19725200
C	-1.36556900	3.67100400	1.42435000
C	-2.62853500	4.19752900	-0.56782300
C	-1.53465500	4.97787400	1.87779600
H	-0.78891600	2.96917500	2.01831600
C	-2.78887200	5.50993500	-0.11452600
H	-3.05751400	3.90599800	-1.52062100
C	-2.24529600	5.90277000	1.10837700
H	-1.09945300	5.27608700	2.82798000
H	-3.33922100	6.22350400	-0.72238600
H	-2.37005900	6.92410000	1.45872500
C	-0.78488900	-4.73815700	2.08741500
Cl	1.15970700	2.99875700	-0.95776700
C	-2.12495900	-4.38537900	2.25406700
C	-2.65163600	-3.25605600	1.61973600
C	-1.77282600	-2.49735000	0.83906100
C	-0.41941100	-2.80587300	0.66120400

C	5.27940600	0.92908000	0.01215900
C	6.20460500	-0.13480500	0.09547200
C	5.77005800	2.23303300	-0.23419200
C	7.56773600	0.10092400	-0.05752700
H	5.83560200	-1.13946500	0.27268900
C	7.13480700	2.45779600	-0.38415600
H	5.05960900	3.05026900	-0.31012000
C	8.03980800	1.39522100	-0.29568200
H	8.26749600	-0.72855800	0.00794600
H	7.49581400	3.46543600	-0.57337600
H	9.10521400	1.57429400	-0.41429700
C	3.88423200	0.72543500	0.16383000
C	1.34620600	0.01928200	2.79908500
C	1.84287800	2.33558100	2.30026500
C	1.33888000	0.31936800	4.16419300
H	1.12662200	-0.99479200	2.48867400
C	1.83888500	2.61920700	3.66507700
H	2.03949500	3.11781900	1.57825600
C	1.58709300	1.61767300	4.60831800
H	1.13096600	-0.47431600	4.87821700
H	2.03969800	3.63726200	3.99125200
H	1.58737400	1.84677300	5.67046900

4. Coordinates of all stationary points of 1t in figure 3

CAT-2t

Cartesian coordinates

ATOM	X	Y	Z
C	-2.33173900	1.58832000	-0.56263300
C	-3.51702700	2.32002800	-0.71888100
H	-4.39985200	2.02096300	-0.16371700
C	-3.57240000	3.43499500	-1.54974100
H	-4.49589200	3.99866600	-1.64483100
C	-2.44088800	3.82159400	-2.26449000
H	-2.50206900	4.68230300	-2.92114200
C	-1.23850400	3.11651500	-2.15273200
C	-1.19595300	2.02562300	-1.27264800
C	0.00001400	3.41546300	-3.00204700
C	1.23853000	3.11649400	-2.15273400
C	1.19596400	2.02560200	-1.27265200
C	2.33174500	1.58827800	-0.56264000
C	3.51704700	2.31996200	-0.71890200
H	4.39987200	2.02088100	-0.16374600
C	3.57243700	3.43492600	-1.54976500
H	4.49594000	3.99857700	-1.64486300
C	2.44092600	3.82154900	-2.26450300
H	2.50211900	4.68225600	-2.92115600
C	0.00000700	2.44218800	-4.21792100
H	0.00000800	1.39762200	-3.89117700

H	0.89018800	2.60689200	-4.83598100
H	-0.89017900	2.60689500	-4.83597300
C	0.00002300	4.85787400	-3.53756000
H	-0.87518100	5.03658400	-4.16894600
H	0.87524200	5.03658600	-4.16892300
H	0.00001200	5.59356100	-2.72651100
C	-3.53096600	0.45673500	1.87954500
C	-4.89739200	0.16303500	1.72843000
H	-5.26083600	-0.28398700	0.80848100
C	-5.79725800	0.42798400	2.76219900
H	-6.85087900	0.19511300	2.63083400
C	-5.34416100	0.98361700	3.96019200
H	-6.04502400	1.18677900	4.76591500
C	-3.98770900	1.27321200	4.11941500
H	-3.62748000	1.70575200	5.04911900
C	-3.08234300	1.01067600	3.08973200
H	-2.03099600	1.25378100	3.20686200
C	-3.15312100	-1.20337000	-0.43733600
C	-3.38160500	-2.44664300	0.17809200
H	-3.08179600	-2.60206800	1.21033900
C	-3.98753900	-3.48613400	-0.52452900
H	-4.15636600	-4.44096700	-0.03400600
C	-4.36763400	-3.30511700	-1.85677300
H	-4.83519600	-4.11790100	-2.40602500
C	-4.13997200	-2.07782900	-2.47867100

H	-4.43278200	-1.92818700	-3.51470900
C	-3.53619200	-1.03266400	-1.77484200
H	-3.37032700	-0.08165400	-2.27033900
C	3.53094900	0.45675000	1.87955900
C	4.89737200	0.16301700	1.72847400
H	5.26081800	-0.28406800	0.80855600
C	5.79723500	0.42801800	2.76223300
H	6.85085300	0.19512200	2.63089000
C	5.34413800	0.98373600	3.96018700
H	6.04499900	1.18694000	4.76590100
C	3.98769000	1.27336200	4.11938100
H	3.62746000	1.70596700	5.04905400
C	3.08232700	1.01077400	3.08970800
H	2.03098400	1.25390400	3.20681700
C	3.15314000	-1.20341500	-0.43727200
C	3.38157800	-2.44668200	0.17818600
H	3.08169900	-2.60209300	1.21041500
C	3.98755900	-3.48618200	-0.52438000
H	4.15635300	-4.44100900	-0.03383300
C	4.36774200	-3.30518400	-1.85660200
H	4.83534000	-4.11797600	-2.40581300
C	4.14012300	-2.07790400	-2.47853100
H	4.43300100	-1.92827500	-3.51455200
C	3.53630000	-1.03272800	-1.77475400
H	3.37047400	-0.08172300	-2.27027500

O	-0.00000100	1.33398400	-1.10939400
P	-2.29731900	0.10166600	0.54703000
P	2.29730900	0.10163500	0.54704400
Cl	-0.00003200	2.13643400	1.98352100
Rh	-0.00000700	-0.16987500	0.93052000
C	-0.00003500	-2.25506100	0.50102600
C	-0.00007000	-2.06255800	1.76238700
C	-0.00001700	-3.16301300	-0.62319100
C	0.00003100	-2.69287100	-1.94884200
C	-0.00004700	-4.55872600	-0.41100900
C	0.00004500	-3.58302900	-3.02154300
H	0.00005700	-1.62092800	-2.12020300
C	-0.00003500	-5.44409300	-1.48467900
H	-0.00007900	-4.93771100	0.60752800
C	0.00001100	-4.96125500	-2.79723600
H	0.00008300	-3.19819200	-4.03841900
H	-0.00005900	-6.51549200	-1.29833600
H	0.00002300	-5.65365100	-3.63508000
C	-0.00015600	-2.49036700	3.17716200
H	-0.00022500	-3.58606900	3.25985800
H	0.88084900	-2.10633900	3.70681500
H	-0.88116600	-2.10623400	3.70673200

1t

Cartesian coordinates

ATOM	X	Y	Z
C	-2.99401200	1.24713500	-0.00028100
C	-3.72416000	0.05524000	0.00003200
C	-3.05783400	-1.17351700	0.00031900
C	-1.66640800	-1.21551300	0.00028100
C	-0.92146500	-0.01879900	-0.00002300
C	-1.60228000	1.21525400	-0.00030000
H	-3.51111100	2.20255500	-0.00050800
H	-4.81030000	0.08401500	0.00005500
H	-3.62445900	-2.10038000	0.00056700
H	-1.14056200	-2.16501600	0.00048700
H	-1.02848800	2.13680300	-0.00053000
C	0.50261900	-0.06029900	-0.00004500
C	1.71824400	-0.11285600	-0.00006500
C	3.16734100	-0.18257600	-0.00007600
O	3.75369300	-1.25402800	-0.00061100
C	3.89336400	1.14993100	0.00059100
H	3.61069100	1.73743300	-0.88138800
H	3.61052900	1.73667100	0.88301000
H	4.97170200	0.97615200	0.00059000

2t

Cartesian coordinates

ATOM	X	Y	Z
C	2.60701800	0.00039200	0.00008400

C	1.39455400	0.00036500	0.00010100
C	-0.03528400	0.00020600	0.00007500
C	-0.75282300	-1.21215800	0.00004500
C	-0.75326300	1.21231800	0.00003500
C	-2.14583600	-1.20834000	-0.00002300
H	-0.20498300	-2.14957600	0.00002900
C	-2.14626000	1.20800800	-0.00003900
H	-0.20582900	2.14997500	0.00008600
C	-2.84787300	-0.00029500	-0.00006800
H	-2.68558400	-2.15165700	-0.00008200
H	-2.68633700	2.15113700	-0.00004600
H	-3.93449600	-0.00048100	-0.00013200
C	4.06665400	-0.00012700	-0.00009900
H	4.46549300	-0.49691100	-0.89351600
H	4.46470700	1.02173000	0.01685700
H	4.46571100	-0.52643400	0.87613100

INT1-1t

Cartesian coordinates

ATOM	X	Y	Z
C	2.34272600	1.87168500	-0.37605400
C	3.53947900	2.52778700	-0.69476400
H	4.42965100	1.93964500	-0.89087800
C	3.59801100	3.91501100	-0.78519400
H	4.53066300	4.40552900	-1.04797100

C	2.45772000	4.67291400	-0.52793800
H	2.52235700	5.75377600	-0.58170900
C	1.24264400	4.06680600	-0.19366600
C	1.19798000	2.66551500	-0.15857600
C	0.00055100	4.85224200	0.23671900
C	-1.24173900	4.06707400	-0.19358000
C	-1.19736700	2.66577300	-0.15851200
C	-2.34229100	1.87218800	-0.37594300
C	-3.53893000	2.52854500	-0.69455000
H	-4.42923600	1.94059000	-0.89061300
C	-3.59718300	3.91578400	-0.78493600
H	-4.52975300	4.40650200	-1.04763000
C	-2.45671300	4.67344200	-0.52774900
H	-2.52113000	5.75431900	-0.58149300
C	0.00061700	4.93464900	1.79232600
H	0.00051100	3.93796500	2.24476600
H	-0.88929900	5.47002800	2.14358200
H	0.89069000	5.46980800	2.14351900
C	0.00068700	6.28496500	-0.32727500
H	0.87517700	6.84052300	0.02345000
H	-0.87364200	6.84072400	0.02353400
H	0.00063600	6.29168100	-1.42237600
C	3.56387700	-0.57636300	-1.46098500
C	4.92474200	-0.68479200	-1.12413900
H	5.26309000	-0.42062300	-0.12723400

C	5.85176500	-1.14521200	-2.06046600
H	6.90028000	-1.22379600	-1.78487800
C	5.43232100	-1.50786500	-3.34174700
H	6.15438000	-1.86915900	-4.06955300
C	4.08217100	-1.40787400	-3.68163300
H	3.74773600	-1.68909200	-4.67673200
C	3.14954100	-0.94847100	-2.74998500
H	2.10292600	-0.85630500	-3.02099700
C	3.11953500	-0.33919900	1.37002300
C	3.38257200	-1.68328000	1.69026500
H	3.14176600	-2.46820500	0.97836400
C	3.96228000	-2.02092600	2.91265600
H	4.16984400	-3.06336800	3.14032300
C	4.27416200	-1.02313200	3.84066200
H	4.72314500	-1.28625800	4.79473500
C	4.00263300	0.31078900	3.53739800
H	4.23563100	1.09219900	4.25568700
C	3.42820300	0.65206400	2.31043400
H	3.21974200	1.69337500	2.08951800
C	-3.56398300	-0.57553000	-1.46095500
C	-4.92486300	-0.68373500	-1.12409400
H	-5.26314900	-0.41960100	-0.12715900
C	-5.85199000	-1.14388800	-2.06045000
H	-6.90051400	-1.22230300	-1.78484800
C	-5.43264400	-1.50648800	-3.34177800

H	-6.15478600	-1.86757000	-4.06960600
C	-4.08248200	-1.40671500	-3.68168100
H	-3.74811800	-1.68789300	-4.67681500
C	-3.14974600	-0.94758800	-2.75000300
H	-2.10311900	-0.85560100	-3.02102600
C	-3.11952700	-0.33858200	1.37007100
C	-3.38302200	-1.68259400	1.69022400
H	-3.14255200	-2.46755100	0.97824300
C	-3.96276300	-2.02013200	2.91263000
H	-4.17068600	-3.06251700	3.14022700
C	-4.27421800	-1.02230100	3.84074100
H	-4.72322900	-1.28534300	4.79482300
C	-4.00222700	0.31154600	3.53756800
H	-4.23488900	1.09298100	4.25593900
C	-3.42776400	0.65271400	2.31059000
H	-3.21893900	1.69396800	2.08974300
O	0.00024400	2.02739200	0.12248000
P	2.30463900	0.02213500	-0.24504300
P	-2.30458900	0.02263000	-0.24500500
Cl	0.00010500	0.47489100	-2.71185000
Rh	-0.00003500	-0.42527300	-0.35708900
C	-0.00004500	-1.03229200	2.89432400
O	0.00013300	0.13631700	3.26526100
C	-0.00012900	-1.40717300	1.48855600
C	-0.00029000	-2.27768800	0.54184400

C	-0.00054000	-3.63413700	0.05082300
C	-0.00063400	-4.72562800	0.94631800
C	-0.00070700	-3.89259800	-1.33385500
C	-0.00088000	-6.03187400	0.46580100
H	-0.00051400	-4.53538400	2.01525800
C	-0.00095600	-5.20293700	-1.80729700
H	-0.00064100	-3.04809600	-2.01675500
C	-0.00104100	-6.27545700	-0.91158400
H	-0.00094900	-6.86351600	1.16573300
H	-0.00108300	-5.38790200	-2.87811100
H	-0.00123400	-7.29671100	-1.28318700
C	-0.00020700	-2.17744700	3.90076500
H	0.88602900	-2.80713700	3.75978700
H	-0.00006300	-1.76744300	4.91360000
H	-0.88671900	-2.80677400	3.75989700

TS1-1t

Cartesian coordinates

ATOM	X	Y	Z
Rh	0.00585700	-0.15784700	1.01372500
C	-0.01940800	1.90161400	1.08901500
C	0.16329500	1.58549600	2.59545800
O	1.27705300	1.61305300	3.11437200
C	-0.13099600	2.92508600	0.38945800
C	2.54710400	-3.22457400	-3.15800700

H	2.63084000	-3.89494400	-4.00617800
C	1.34151800	-2.56458900	-2.90743300
C	1.26621200	-1.72373000	-1.78652800
C	0.11365800	-2.64951600	-3.81788600
C	-1.12371300	-2.63906600	-2.91572800
C	-1.10562500	-1.80050500	-1.78913600
C	-2.23311200	-1.63305200	-0.95951800
C	-3.38044200	-2.37417000	-1.27844100
H	-4.25811500	-2.29178800	-0.64845700
C	-3.40851700	-3.24092200	-2.36604300
H	-4.30496000	-3.81597400	-2.57941400
C	-2.28573600	-3.36703300	-3.18082700
H	-2.32240000	-4.03648700	-4.03305800
C	0.07864700	-1.37731900	-4.71519000
H	0.05107300	-0.46518900	-4.11154200
H	-0.80915900	-1.39051700	-5.35823700
H	0.96947300	-1.33835700	-5.35286300
C	0.15347000	-3.88388800	-4.73445300
H	1.03213500	-3.85356500	-5.38585300
H	-0.71936600	-3.90415200	-5.39402600
H	0.17794500	-4.81735100	-4.16230200
C	3.53953100	-0.96543500	1.70688500
C	4.91476200	-0.70368900	1.57777600
H	5.28078800	-0.10453500	0.74973700
C	5.82191100	-1.19300000	2.51860200

H	6.88210300	-0.98184800	2.40418300
C	5.36712400	-1.94123700	3.60602800
H	6.07311600	-2.31820200	4.34169300
C	4.00215600	-2.19483100	3.74801500
H	3.63916900	-2.77000600	4.59564300
C	3.09074600	-1.70923500	2.80879500
H	2.03241900	-1.91707600	2.91719300
C	3.17354800	1.18658600	-0.14094200
C	3.46086600	2.22028700	0.77058000
H	3.12247300	2.13692000	1.79897200
C	4.16693400	3.34939000	0.35593900
H	4.39296400	4.13282500	1.07479100
C	4.57980900	3.47769400	-0.97441400
H	5.13630300	4.35575300	-1.29259400
C	4.26984300	2.47243200	-1.89092000
H	4.57963300	2.56392400	-2.92893700
C	3.57281900	1.33280900	-1.47710900
H	3.35784100	0.55113200	-2.19812700
C	-3.43786100	-1.24447000	1.71525300
C	-4.83503000	-1.19412800	1.55830900
H	-5.27101900	-0.68970300	0.70168300
C	-5.68007700	-1.76930100	2.50827700
H	-6.75707900	-1.72095000	2.36949300
C	-5.14344800	-2.39852800	3.63345300
H	-5.80169500	-2.84482400	4.37439900

C	-3.75935900	-2.44615300	3.80344700
H	-3.33278300	-2.93239700	4.67666800
C	-2.91028500	-1.87187000	2.85502400
H	-1.83464400	-1.93064500	2.97954100
C	-3.25539700	0.93819700	-0.15734500
C	-3.98945200	1.76423800	0.71174300
H	-4.00912700	1.55099700	1.77608900
C	-4.73838300	2.83543000	0.22073600
H	-5.31627200	3.44633700	0.90966300
C	-4.75578100	3.11180400	-1.14858400
H	-5.34679700	3.93891900	-1.53307200
C	-4.00785600	2.31692800	-2.01827300
H	-4.00808100	2.52538600	-3.08487100
C	-3.26508100	1.24103600	-1.52755200
H	-2.70349400	0.62407100	-2.22148700
O	0.05749600	-1.10030900	-1.50554000
P	2.29998100	-0.31826100	0.49477300
P	-2.25634700	-0.48034200	0.50608400
Cl	0.08070000	-2.61548500	1.36711900
C	2.37499600	-1.48092600	-0.95177900
H	4.43062300	-2.01776500	-0.61244000
C	3.65303900	-3.03028600	-2.33298500
C	3.56604000	-2.15903400	-1.25143400
H	4.58235700	-3.55520400	-2.53471600
C	-0.23813100	4.08262300	-0.41269700

C	-1.48030200	4.74370900	-0.55353400
C	0.89665100	4.60024100	-1.08037600
C	-1.57775200	5.89123400	-1.33236200
H	-2.35122100	4.33954900	-0.04995000
C	0.78295600	5.74151600	-1.86665100
H	1.84929400	4.09509600	-0.97081600
C	-0.44960600	6.39107200	-1.99148700
H	-2.53483200	6.39550800	-1.43294700
H	1.65781000	6.13057400	-2.38008700
H	-0.53130400	7.28608300	-2.60264300
C	-1.09572900	1.65977900	3.44957300
H	-1.99036100	1.85487700	2.85678100
H	-1.22067400	0.71521500	3.98890000
H	-0.96468000	2.45949700	4.18866200

INT2-1t

Cartesian coordinates

ATOM	X	Y	Z
Rh	-0.60808470	0.93619909	-0.41923924
C	-0.44232654	0.73668580	-2.38955849
O	0.28673678	-0.09506371	-2.86685185
C	-2.59093731	0.99134467	-0.37760098
C	-3.79987496	1.16828761	-0.28081494
C	-0.28148781	-4.01091314	-1.03234661
C	3.22964148	-1.00375323	-0.40972144

C	4.23261492	-1.89405055	-0.80836241
C	5.39837104	-1.34112346	-1.34659431
H	6.20425848	-1.98811199	-1.67668315
C	5.54620854	0.04207416	-1.45920695
C	4.52519701	0.89880934	-1.05027792
C	3.32919396	0.38477982	-0.52607460
H	0.20140002	-5.93535143	-1.85707267
H	2.63998918	-5.61791671	-1.67733427
H	-1.34784768	-4.17115497	-1.12503145
H	6.46252361	0.45510233	-1.87106015
H	4.65141661	1.96996071	-1.15253831
O	2.05828986	-1.48856465	0.13657945
C	3.99498828	-3.38955992	-0.57245647
C	4.88208782	-4.26324492	-1.47530167
H	4.71283683	-5.32565674	-1.27508729
H	4.69079051	-4.07553035	-2.53708649
H	5.94131652	-4.07490850	-1.27584030
C	4.32453750	-3.71385862	0.91368548
H	3.71749737	-3.11176897	1.59672464
H	4.13028929	-4.77201309	1.12406810
H	5.38039084	-3.50577399	1.12153482
P	-0.92013103	-1.41712652	0.00949203
P	1.88808906	1.43388068	-0.01929007
C	-2.52724222	-2.08757428	-0.60911185
C	-3.43461319	-2.74677136	0.22859077

C	-2.80752845	-1.99743163	-1.98225936
C	-4.60636897	-3.30043000	-0.29587051
H	-3.23481229	-2.82897501	1.29159887
C	-3.97241640	-2.55624486	-2.50400780
H	-2.10908894	-1.49956742	-2.64762761
C	-4.87766742	-3.20824116	-1.66117982
H	-5.30402485	-3.80498915	0.36728091
H	-4.17589467	-2.47872805	-3.56856568
H	-5.78742977	-3.64140779	-2.06826318
C	-1.01576408	-1.57820926	1.84583345
C	-1.81780696	-0.66795716	2.55834421
C	-0.30626216	-2.55998998	2.55288675
C	-1.90820393	-0.74964137	3.94800871
H	-2.38005896	0.09199697	2.02269483
C	-0.39417957	-2.63032745	3.94449676
H	0.31212221	-3.27561186	2.02149269
C	-1.19422430	-1.72687539	4.64497322
H	-2.53679922	-0.04416015	4.48446052
H	0.16296379	-3.39526127	4.47894606
H	-1.26167682	-1.78407792	5.72813008
C	2.12004321	1.59762350	1.81064226
C	1.11862947	2.23828500	2.56222486
C	3.24548459	1.08822556	2.47639566
C	1.24904912	2.36648967	3.94575014
H	0.25057340	2.65515572	2.06002253

C	3.36708047	1.21326189	3.86177282
H	4.03334474	0.59475108	1.91700092
C	2.36981228	1.85153570	4.60014605
H	0.46886248	2.86949358	4.51073827
H	4.24497596	0.81186729	4.36156685
H	2.46618443	1.94831901	5.67844648
C	2.43065755	3.05862181	-0.71147769
C	2.97484611	4.07696574	0.07933806
C	2.31885019	3.25717266	-2.09700435
C	3.39398897	5.27598956	-0.50353956
H	3.06563545	3.94211829	1.15201141
C	2.74499328	4.45004127	-2.67723665
H	1.90018989	2.47626121	-2.72615850
C	3.28084959	5.46558977	-1.88039757
H	3.80707592	6.06146840	0.12386555
H	2.65111598	4.59041314	-3.75082095
H	3.60535090	6.39931690	-2.33194225
C	0.59612925	-5.00993115	-1.44793246
Cl	-0.77349909	3.35817757	-0.27739359
C	1.97519140	-4.82835660	-1.34386767
C	2.50229957	-3.65078593	-0.80762271
C	1.59201317	-2.66412420	-0.40634750
C	0.20364654	-2.79980402	-0.51210545
C	-5.19512032	1.44088830	-0.14084356
C	-5.63020632	2.73579895	0.21501867

C	-6.16739636	0.44080008	-0.34953167
C	-6.98710997	3.01485482	0.35837273
H	-4.88561207	3.51013206	0.37315506
C	-7.52321898	0.72855339	-0.20549701
H	-5.84369317	-0.55786423	-0.62490959
C	-7.94035221	2.01405275	0.14927939
H	-7.30298450	4.01828483	0.63301475
H	-8.25907143	-0.05480959	-0.37106034
H	-8.99884878	2.23467031	0.26084920
C	-1.27236097	1.70857381	-3.20913812
H	-1.06694433	1.54892822	-4.27277897
H	-2.32984119	1.54551858	-2.98080515
H	-1.04066552	2.73239206	-2.90350885

TS2-1t

Cartesian coordinates

ATOM	X	Y	Z
Rh	0.75354500	0.11041900	-1.64057800
C	0.92726100	-0.82778800	-3.24425100
O	1.07965100	-1.60622700	-4.09099300
C	0.27582900	0.97241700	-3.76593600
C	-1.72093700	-2.05740300	2.47109700
C	-0.66457200	-1.55136100	1.71200600
C	0.22030900	-2.34746300	0.97531100
C	0.03946900	-3.73384100	1.02638300

H	0.71060000	-4.38919200	0.48317100
C	-1.01278800	-4.27630600	1.76653700
H	-1.15267000	-5.35307200	1.79298400
C	-1.88436500	-3.44793600	2.47389700
H	-2.69255200	-3.89763600	3.04066800
C	-1.84428500	-0.85658600	4.66502000
H	-0.82992700	-0.47046100	4.52219700
H	-1.77487100	-1.80202400	5.21541200
H	-2.40141500	-0.13772600	5.27711900
C	-3.97640400	-1.62469600	3.56027300
H	-4.55289000	-0.93674700	4.18595400
H	-3.93347600	-2.57259600	4.10483700
H	-4.52078800	-1.78541600	2.62392600
C	-0.77017900	3.42156600	-1.22500900
C	-1.83212900	3.03411000	-2.06414000
H	-2.18597300	2.00637100	-2.04475800
C	-2.43402000	3.95868200	-2.91702400
H	-3.25604400	3.64417400	-3.55448500
C	-1.97785300	5.27878900	-2.95793100
H	-2.44351900	5.99707700	-3.62739800
C	-0.91949800	5.66857400	-2.13722900
H	-0.55771400	6.69310100	-2.16206100
C	-0.32002900	4.74824600	-1.27285600
H	0.49513800	5.07067300	-0.63439700
C	1.17873000	3.06143700	0.91021200

C	0.94198500	3.36511700	2.25932200
H	0.01870700	3.05131400	2.73467000
C	1.88683700	4.07602800	3.00163500
H	1.68919000	4.30031100	4.04660400
C	3.07398800	4.50256800	2.40480900
H	3.80500800	5.06226600	2.98257500
C	3.31861400	4.20436200	1.06224000
H	4.24261400	4.52649600	0.58936000
C	2.38429300	3.48074200	0.31952100
H	2.60179400	3.21853700	-0.71091800
C	2.45836300	-2.82085100	-0.80911300
C	3.82795300	-3.02881100	-0.59306400
H	4.36757300	-2.40723900	0.11280900
C	4.50707300	-4.03124600	-1.28859600
H	5.56975900	-4.17708400	-1.11486900
C	3.82807800	-4.83936800	-2.20064100
H	4.35887100	-5.61900800	-2.74035300
C	2.46415800	-4.63693000	-2.42421500
H	1.92926600	-5.25479100	-3.14016900
C	1.78496100	-3.62850400	-1.74195400
H	0.72856300	-3.46858300	-1.93626200
C	2.67035600	-0.94572600	1.40915800
C	3.47568200	0.19483300	1.29156400
H	3.44140600	0.78734400	0.38526900
C	4.33619700	0.55778300	2.33101200

H	4.94479900	1.45144400	2.22730700
C	4.40665900	-0.21142100	3.49184300
H	5.07563800	0.07544900	4.29917200
C	3.61297100	-1.35468700	3.61275400
H	3.66278300	-1.96338300	4.51190700
C	2.74973200	-1.72010500	2.58079200
H	2.13737600	-2.60910700	2.69120500
O	-0.42577800	-0.19671600	1.66499700
P	-0.03284300	2.12618100	-0.12349700
P	1.54142400	-1.46424000	0.03633100
C	-1.45065600	1.83026000	1.03410300
H	-2.49907900	3.67476400	0.65071400
C	-3.60426100	2.41356500	1.99373900
C	-2.51479300	2.73114100	1.18399600
H	-4.42244000	3.12011300	2.09977900
C	-3.65605400	1.18629200	2.65739600
H	-4.52385400	0.95372300	3.26540300
C	-2.60768300	0.26595400	2.55582600
C	-1.51582100	0.63375000	1.75972700
C	-2.26055300	-0.99408500	-1.46473000
Cl	3.04390200	1.00224700	-2.04821700
C	-3.60206500	-1.48541900	-1.41939700
C	-4.60118200	-0.80572800	-0.69063600
C	-3.96186300	-2.66603200	-2.10207000
C	-5.90602800	-1.29358100	-0.64710300

H	-4.33946600	0.10682600	-0.16429000
C	-5.26821000	-3.14767300	-2.05426000
H	-3.20189200	-3.19632100	-2.66834600
C	-6.24729300	-2.46609900	-1.32672800
H	-6.66209100	-0.75247400	-0.08311600
H	-5.52371400	-4.05926100	-2.58880400
H	-7.26607700	-2.84294000	-1.29246400
C	-1.11070400	-0.57333600	-1.47843500
C	-2.56053000	-1.07624700	3.30163800
H	0.11964400	1.91652300	-3.24880900
H	1.10584200	1.07841400	-4.46310200
H	-0.64810400	0.62559200	-4.22625200

INT3-1t

Cartesian coordinates

ATOM	X	Y	Z
Rh	-0.63234600	0.40366400	-1.74570200
C	0.08501000	1.51603200	-3.09764100
O	0.45639900	2.13481100	-3.98514400
C	2.20746100	-1.91084400	3.17335300
C	2.46780300	-0.49933900	2.62790000
C	1.50275500	0.12590300	1.82846300
C	1.65082200	1.41910500	1.30738500
C	2.80097300	2.13447700	1.66983800
H	2.94732200	3.14095500	1.29438400

C	3.76782100	1.55534400	2.48844000
H	4.65523600	2.11903800	2.76117200
C	3.60659900	0.24797300	2.94674900
H	4.38023200	-0.18746000	3.57015900
C	1.54970800	-1.78130200	4.57660300
H	0.61087000	-1.22042700	4.52204600
H	2.22147800	-1.25733000	5.26648300
H	1.33110100	-2.77269900	4.99020300
C	3.52352700	-2.70649800	3.30160900
H	3.34055400	-3.69880900	3.72427400
H	4.22001600	-2.20759000	3.98143100
H	4.01085300	-2.82709800	2.32885200
C	-2.96595100	-2.36775700	-1.12567900
C	-2.50152100	-3.36732900	-1.99964800
H	-1.43612500	-3.53237000	-2.12274000
C	-3.39834100	-4.15723100	-2.71881800
H	-3.01932100	-4.92972300	-3.38243200
C	-4.77331800	-3.94768900	-2.59593800
H	-5.47148300	-4.55928400	-3.16099700
C	-5.24415700	-2.94331000	-1.75025200
H	-6.31174700	-2.76537400	-1.65445000
C	-4.34958300	-2.15961200	-1.01891300
H	-4.73352500	-1.38382700	-0.36607600
C	-2.81179200	-0.64862200	1.19049900
C	-3.09537800	-1.49244700	2.27929300

H	-2.69369800	-2.50022500	2.30986200
C	-3.89236800	-1.04449900	3.33136900
H	-4.10196900	-1.70791700	4.16650800
C	-4.41725600	0.25046200	3.31215900
H	-5.03569600	0.59990300	4.13497200
C	-4.14470400	1.08959800	2.23294700
H	-4.54213000	2.10003300	2.20893400
C	-3.34697200	0.64536600	1.17432300
H	-3.15633800	1.29540700	0.32880300
C	1.42913500	3.33894100	-0.77183200
C	1.18927100	4.71904100	-0.73640800
H	0.37235300	5.11611800	-0.14390400
C	2.00048600	5.59786800	-1.46054300
H	1.79787800	6.66502600	-1.42420300
C	3.06432100	5.11186600	-2.21954400
H	3.69428400	5.79714500	-2.78026300
C	3.31367000	3.73725100	-2.25736600
H	4.13872000	3.34812100	-2.84810800
C	2.50133900	2.85672200	-1.54538800
H	2.69755300	1.78811100	-1.58872900
C	-0.70335500	3.13233800	1.19306300
C	-1.79159900	3.79130800	0.59267600
H	-1.99911200	3.64683100	-0.46335900
C	-2.62813900	4.60824300	1.35510800
H	-3.46003400	5.11678500	0.87498200

C	-2.40517300	4.76245200	2.72565000
H	-3.06056000	5.39553000	3.31821700
C	-1.33881200	4.09614100	3.33119900
H	-1.16084600	4.20615800	4.39778400
C	-0.48949900	3.29011700	2.57064800
H	0.34187900	2.78718500	3.05314000
O	0.32591300	-0.51233100	1.52513700
P	-1.78524200	-1.27133500	-0.22201200
P	0.38997700	2.09152800	0.12892100
C	-0.67277200	-2.45174400	0.66987200
H	-1.54641700	-4.33030900	0.06777400
C	0.11539400	-4.62725900	1.39469200
C	-0.77388000	-3.84679000	0.65301400
H	0.03021300	-5.70984100	1.37113100
C	1.10190300	-4.02534900	2.17408200
H	1.77427700	-4.65430400	2.74778400
C	1.22098000	-2.63143000	2.24484600
C	0.31943000	-1.88378000	1.48359700
C	2.12368300	-1.25010400	-1.62204700
Cl	-2.75864300	1.66700600	-2.09591900
C	3.34625200	-1.98886700	-1.57611000
C	3.39623800	-3.27074700	-0.98763200
C	4.53698000	-1.45841300	-2.11504800
C	4.59140600	-3.98597700	-0.93833800
H	2.48628500	-3.69449400	-0.57455600

C	5.72835200	-2.17863700	-2.06219400
H	4.51200700	-0.47575000	-2.57701000
C	5.76391500	-3.44502200	-1.47298700
H	4.60650800	-4.97371600	-0.48374100
H	6.63373200	-1.74965900	-2.48447300
H	6.69410000	-4.00566600	-1.43433300
C	1.06922000	-0.62721400	-1.62866400
C	-1.20320200	-0.79691700	-3.40477200
H	-0.65419800	-1.73520900	-3.34905000
H	-2.27875600	-0.92658600	-3.31287200
H	-0.97256300	-0.29429000	-4.34565500

INT4-1t

Cartesian coordinates

ATOM	X	Y	Z
Rh	-0.59299600	1.25973100	-0.40033700
C	-0.61275500	3.07344700	-1.50419900
C	-0.52384800	0.39173200	-2.04951700
O	-0.54730700	-0.11686300	-3.07950000
C	-1.04766600	-3.80767000	-0.42524300
C	2.46166100	-0.90648200	-1.61464300
C	2.94225900	-1.73710600	-2.63046500
C	3.62433100	-1.11787700	-3.68433000
H	4.00473200	-1.71288600	-4.50768900
C	3.82317300	0.26335800	-3.69656200

C	3.37750000	1.05488200	-2.63651900
C	2.69729600	0.47595300	-1.55713600
H	-1.20719800	-5.66970800	-1.48913300
H	0.90234500	-5.36827200	-2.72553700
H	-1.99443200	-3.93928700	0.08512800
H	4.34144100	0.72583800	-4.53145100
H	3.55200500	2.12465300	-2.65466400
O	1.69772500	-1.42783100	-0.59076200
C	2.75391600	-3.25069800	-2.46728400
C	2.87120800	-3.99000400	-3.81147000
H	2.76946500	-5.07033600	-3.67248900
H	2.10888200	-3.65952500	-4.52479300
H	3.85712900	-3.82732600	-4.25752800
C	3.86291700	-3.77615000	-1.50993100
H	3.81667900	-3.27747400	-0.53663100
H	3.74359900	-4.85344500	-1.34705300
H	4.85493200	-3.59665100	-1.94050900
P	-0.92017500	-1.22411300	0.83121400
P	1.95319300	1.38311600	-0.13084800
C	-2.69296900	-1.71083800	1.05478600
C	-3.21589000	-2.15272600	2.27919800
C	-3.54936300	-1.63998500	-0.05731300
C	-4.56200700	-2.51040400	2.38828900
H	-2.57879000	-2.22027300	3.15332300
C	-4.89033200	-2.00475800	0.05234300

H	-3.17385100	-1.28889000	-1.01116000
C	-5.40203000	-2.43779500	1.27742400
H	-4.94935100	-2.84741700	3.34626800
H	-5.53802200	-1.92913200	-0.81628800
H	-6.45002500	-2.71183800	1.36490300
C	-0.20773700	-1.52287300	2.50457600
C	-0.51200500	-0.59996200	3.52114000
C	0.56664600	-2.64954800	2.81477600
C	-0.07443300	-0.82459200	4.82667200
H	-1.08292500	0.29388600	3.28806400
C	1.02040400	-2.85722200	4.11888100
H	0.80822200	-3.37412100	2.04363100
C	0.69298200	-1.95190300	5.12916500
H	-0.32828700	-0.11057400	5.60581500
H	1.62101200	-3.73450300	4.34487200
H	1.03618200	-2.12245200	6.14632300
C	2.83551700	0.68570900	1.34098900
C	2.38875900	1.06918000	2.61409200
C	3.99441700	-0.09640600	1.22834200
C	3.09687000	0.67797600	3.75140400
H	1.48810700	1.66810100	2.71125900
C	4.69397600	-0.48981900	2.37002400
H	4.36325900	-0.39570600	0.25240900
C	4.24851400	-0.10125700	3.63434500
H	2.73938000	0.97954200	4.73164800

H	5.59037900	-1.09633100	2.26805500
H	4.79638800	-0.40419700	4.52279300
C	2.74193700	3.05940800	-0.15589600
C	1.98804500	4.18836200	0.19520400
C	4.12286700	3.20880300	-0.37936900
C	2.59455500	5.44491600	0.27648000
H	0.93833300	4.08031500	0.44017100
C	4.72181700	4.46522100	-0.30647900
H	4.73868600	2.34129400	-0.59507400
C	3.95610100	5.58936800	0.01437600
H	1.99548900	6.30854300	0.55192500
H	5.78877800	4.56379400	-0.48841100
H	4.42428900	6.56829400	0.07471200
C	-0.60761100	-4.77960000	-1.32197900
Cl	-0.85099700	2.49772800	1.69337200
C	0.58993200	-4.60907400	-2.01675600
C	1.39030600	-3.48280100	-1.80208700
C	0.93004800	-2.53907300	-0.87496500
C	-0.29554800	-2.64455000	-0.19759000
C	-5.16474700	1.69415200	-1.06684800
C	-5.77269500	1.36055000	-2.29531400
C	-5.98315900	2.18529200	-0.02804700
C	-7.14607100	1.51293600	-2.47530100
H	-5.15161400	0.98593900	-3.10417300
C	-7.35501500	2.33651700	-0.21543300

H	-5.52298000	2.44435600	0.92052200
C	-7.94439600	2.00128800	-1.43773200
H	-7.59492800	1.25200500	-3.43070500
H	-7.96824800	2.71825000	0.59716900
H	-9.01515100	2.12106500	-1.58077300
C	-2.56700400	1.37775900	-0.65882700
C	-3.75939500	1.53408200	-0.87450100
H	-0.93968400	3.82606400	-0.78936500
H	0.38899900	3.28012300	-1.88377000
H	-1.32772800	2.99317500	-2.32194500

TS3-1t

Cartesian coordinates

ATOM	X	Y	Z
Rh	-0.66740300	1.15957700	-0.07502800
C	-2.55108000	1.73983600	-0.44891900
C	-3.76593200	1.71053500	-0.65570200
C	-1.45570300	3.31340200	-0.57929600
C	-1.05242900	-3.65727400	-1.08032600
C	2.61349400	-0.74295500	-1.57118900
C	3.20061300	-1.46032800	-2.61882700
C	4.07029800	-0.75769600	-3.46033700
H	4.54787100	-1.26596800	-4.29095300
C	4.34898800	0.59177400	-3.24078100
C	3.75543600	1.27270900	-2.17772600

C	2.85658100	0.61703100	-1.32560400
H	-1.20250300	-5.29741200	-2.46363000
H	1.01015300	-4.90881000	-3.47036500
H	-2.03094600	-3.83651900	-0.65025700
H	5.03717100	1.11445000	-3.89889600
H	3.98139100	2.32084500	-2.01699800
O	1.74721400	-1.36882300	-0.70097800
C	2.92129800	-2.96581100	-2.71694400
C	3.13215000	-3.49338200	-4.14743200
H	2.96406100	-4.57339200	-4.19287900
H	2.45913300	-3.00746000	-4.86158300
H	4.16284300	-3.32752300	-4.47469200
C	3.90319400	-3.70453500	-1.76165300
H	3.79220900	-3.35409900	-0.73065400
H	3.71078100	-4.78352700	-1.77904200
H	4.94011100	-3.52988400	-2.07131000
P	-0.86778800	-1.37876400	0.66436000
P	1.94900300	1.41296400	0.08034400
C	-2.62422200	-1.89939300	0.88430400
C	-3.07628800	-2.64656200	1.98358700
C	-3.54585200	-1.53233500	-0.11086700
C	-4.41867400	-3.01669900	2.08191700
H	-2.38370200	-2.94273300	2.76338800
C	-4.88548500	-1.90980400	-0.01264200
H	-3.21969400	-0.94107900	-0.95841900

C	-5.32555400	-2.65016800	1.08584400
H	-4.75301000	-3.59512400	2.93932300
H	-5.58493400	-1.60514100	-0.78553100
H	-6.37099500	-2.93585600	1.16738300
C	-0.05032400	-1.95154200	2.21700100
C	-0.37729700	-1.30019200	3.42020800
C	0.86719300	-3.01221100	2.24771400
C	0.18907500	-1.71919900	4.62423600
H	-1.06251100	-0.45931700	3.40914900
C	1.44225800	-3.41713300	3.45382500
H	1.12764500	-3.53605000	1.33418700
C	1.10183800	-2.77616100	4.64548800
H	-0.08135200	-1.21146900	5.54645800
H	2.15086600	-4.24133600	3.45963100
H	1.54485600	-3.09789600	5.58445500
C	2.95637500	0.88326300	1.53754000
C	2.38823800	0.94285500	2.81920300
C	4.29187600	0.46901100	1.39936200
C	3.14900400	0.60002400	3.93943200
H	1.35584100	1.25698200	2.93609900
C	5.04370700	0.12217700	2.52181300
H	4.74907300	0.41821500	0.41634300
C	4.47430400	0.18884300	3.79540500
H	2.69558900	0.64748800	4.92547900
H	6.07533500	-0.19803200	2.39980500

H	5.06146500	-0.08080200	4.66957700
C	2.39390200	3.19600800	-0.11858100
C	1.95182000	3.88238000	-1.26473800
C	3.08984100	3.91309400	0.86556700
C	2.22312300	5.24029800	-1.43310800
H	1.40314800	3.34981900	-2.03758100
C	3.34716800	5.27613000	0.70162900
H	3.43300900	3.40918300	1.76245900
C	2.92025700	5.94244500	-0.44735600
H	1.88139900	5.74989400	-2.33005300
H	3.88632100	5.81518400	1.47604600
H	3.12391100	7.00238500	-0.57284100
C	-0.58565700	-4.47802200	-2.10593400
Cl	-0.96557300	1.92312900	2.22854500
C	0.66916000	-4.25597700	-2.67398400
C	1.49009900	-3.21707400	-2.22217900
C	0.98609200	-2.40728700	-1.19760200
C	-0.27646200	-2.58752800	-0.61123100
C	-5.16813100	1.70073200	-0.86149100
C	-5.71576100	1.48763400	-2.14826900
C	-6.05974300	1.89892700	0.21871600
C	-7.09450400	1.47374400	-2.34141300
H	-5.04223900	1.33550900	-2.98698800
C	-7.43605800	1.88786000	0.01391500
H	-5.64963900	2.05835400	1.21120500

C	-7.96281800	1.67453100	-1.26403100
H	-7.49473200	1.30864600	-3.33879300
H	-8.10400500	2.04424000	0.85733600
H	-9.03839400	1.66544200	-1.41878900
C	-0.51418100	0.67062200	-1.85650600
O	-0.45519000	0.37813000	-2.97078900
H	-1.72873000	3.55679500	-1.60349800
H	-2.05711700	3.83136200	0.16100100
H	-0.40758900	3.56341700	-0.40322800

INT5-1t

Cartesian coordinates

ATOM	X	Y	Z
Rh	-0.72592600	1.17622900	-0.24145400
C	-1.30199200	3.14038100	-0.91738300
C	-2.37784100	2.47950900	-0.91989400
O	-0.27816900	0.31679200	-3.07887700
C	-1.61697000	-3.44049500	-1.31954000
C	2.52135500	-1.19336700	-1.25036200
C	3.14526200	-2.04411700	-2.17231800
C	4.27013800	-1.54819000	-2.83847300
H	4.78652900	-2.16860700	-3.56279100
C	4.75472400	-0.26732900	-2.57311200
C	4.10461100	0.55523100	-1.65478300
C	2.94988500	0.11786300	-0.98797800

H	-1.84526500	-5.03876100	-2.74229800
H	0.52643000	-5.05860100	-3.40029200
H	-2.66152400	-3.45238100	-1.03005200
H	5.64285100	0.09168800	-3.08532000
H	4.48822900	1.55107200	-1.46626500
O	1.43239800	-1.63501800	-0.53300800
C	2.61514500	-3.47553600	-2.32935300
C	2.95611300	-4.06168200	-3.71159200
H	2.59506500	-5.09094000	-3.79703300
H	2.51447100	-3.47129500	-4.52124000
H	4.03896000	-4.10148700	-3.86267300
C	3.27543700	-4.36177200	-1.23333300
H	3.05958900	-3.97825100	-0.23112500
H	2.89703300	-5.38875400	-1.29527500
H	4.36362500	-4.38222500	-1.36416900
P	-1.24902500	-1.23843300	0.51588400
P	1.95646400	1.17372000	0.17795500
C	-3.05998500	-1.58716800	0.66894900
C	-3.60448400	-2.34963900	1.71338500
C	-3.91825200	-1.09367100	-0.32579400
C	-4.97558700	-2.61217600	1.75711700
H	-2.96168100	-2.74034000	2.49445000
C	-5.28511900	-1.36888900	-0.28876400
H	-3.51905400	-0.48377800	-1.12962600
C	-5.81799200	-2.12708800	0.75584800

H	-5.38240500	-3.19963400	2.57622600
H	-5.93146000	-0.96951600	-1.06460100
H	-6.88465400	-2.33283400	0.79250200
C	-0.56860700	-1.94000500	2.08963500
C	-0.64047000	-1.16297300	3.25746500
C	-0.03420600	-3.23591600	2.16906600
C	-0.19387700	-1.67906900	4.47509700
H	-1.03532200	-0.15336100	3.20682500
C	0.42173600	-3.74205900	3.38747400
H	0.02087600	-3.86019500	1.28351900
C	0.34067600	-2.96631000	4.54489000
H	-0.25813500	-1.06530500	5.36965200
H	0.83434600	-4.74692800	3.43018500
H	0.69243000	-3.36295900	5.49386000
C	2.70798800	0.76295300	1.81924000
C	2.05223800	1.19986700	2.98218800
C	3.92237100	0.06854300	1.94195200
C	2.61532300	0.96258900	4.23803500
H	1.09826700	1.71257000	2.90265900
C	4.47179100	-0.17848700	3.20065800
H	4.44570000	-0.27748800	1.05666100
C	3.82308100	0.27279200	4.35173900
H	2.09913700	1.31137500	5.12863700
H	5.41103300	-0.72012400	3.27957600
H	4.25534600	0.08370800	5.33105000

C	2.65906100	2.85329800	-0.18181000
C	2.58709900	3.35779800	-1.49294400
C	3.21101500	3.66748700	0.81727000
C	3.07723900	4.62649200	-1.79896400
H	2.15401000	2.75125800	-2.28432600
C	3.68209300	4.94792800	0.51316200
H	3.27842200	3.30499400	1.83726700
C	3.62337200	5.42975900	-0.79401400
H	3.02460200	4.99108200	-2.82168900
H	4.10304700	5.56370900	1.30363000
H	3.99743600	6.42234700	-1.02996400
C	-1.15500600	-4.33553600	-2.28513700
Cl	-1.25176200	2.05157400	2.01952900
C	0.18926200	-4.34323300	-2.65769600
C	1.10235000	-3.45505500	-2.07754600
C	0.59759800	-2.55159300	-1.13708300
C	-0.74396500	-2.51740700	-0.72974400
C	-3.81170100	2.33370400	-1.00928000
C	-4.45066200	2.19160500	-2.25579500
C	-4.59669000	2.38330300	0.15975200
H	-3.84873700	2.15174400	-3.15946200
C	-5.98524100	2.31624700	0.07418200
H	-4.09501400	2.47348100	1.11820000
C	-6.61337500	2.18565200	-1.16833300
H	-6.58080300	2.36419300	0.98206700

H	-7.69747800	2.13629500	-1.23001100
C	-0.44759000	0.63049500	-1.97856200
C	-0.61666300	4.44538800	-1.01160300
H	0.09743400	4.47582700	-1.83981400
H	-1.35456000	5.24417300	-1.16196400
H	-0.06057400	4.65269400	-0.09148100
C	-5.84137400	2.11857700	-2.33150300
H	-6.32287900	2.01876900	-3.30106000

5. Coordinates of radical structures in figure 4

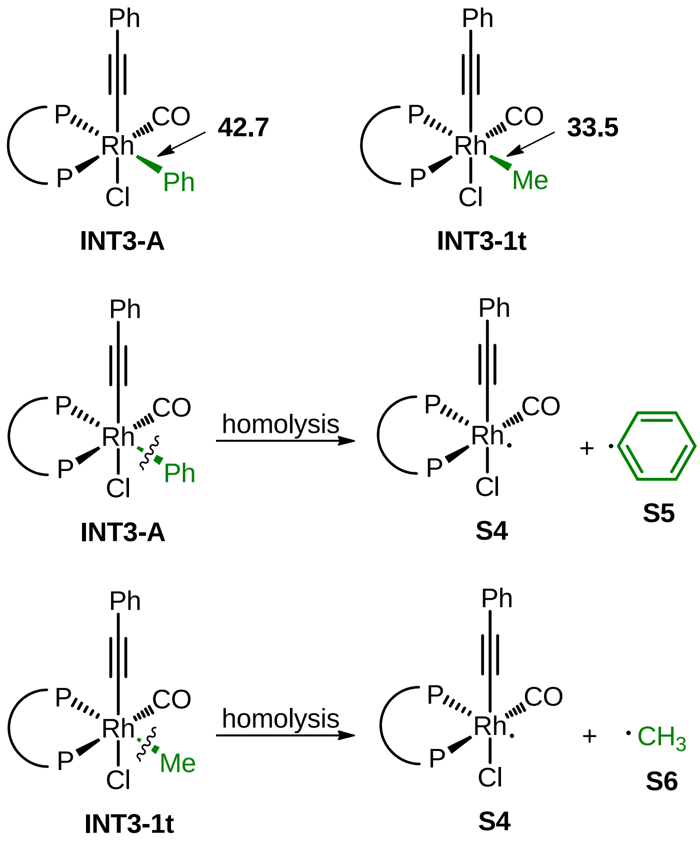

Figure S5

S4

Zero-point correction= 0.709621 (Hartree/Particle)

Thermal correction to Energy= 0.758919

Thermal correction to Enthalpy= 0.759863

Thermal correction to Gibbs Free Energy= 0.620727

Sum of electronic and zero-point Energies= -3253.548765

Sum of electronic and thermal Energies= -3253.499467

Sum of electronic and thermal Enthalpies= -3253.498523

Sum of electronic and thermal Free Energies= -3253.637660

Cartesian coordinates

ATOM	X	Y	Z
Rh	-0.69180400	0.00063200	-1.64704200
C	-0.63167500	1.15226200	-3.18703200
O	-0.59482600	1.75916500	-4.15742800
C	2.52510100	-0.10152400	3.49394400
C	1.98277700	1.17295400	2.83011200
C	0.89313900	1.05691600	1.95852600
C	0.29473700	2.14885300	1.31554400
C	0.79298200	3.42524800	1.61743600
H	0.35254300	4.29887500	1.15095900
C	1.86748700	3.57807000	2.49150000
H	2.24831500	4.57163500	2.70931100
C	2.46449100	2.46172300	3.07883100
H	3.31001800	2.60535000	3.74286200
C	1.66079700	-0.40568000	4.75149700
H	0.60601900	-0.52986000	4.48720300
H	1.73848200	0.41452500	5.47462100
H	2.00337500	-1.32861000	5.23341500
C	3.99355400	0.06058100	3.92474800
H	4.35315400	-0.84616500	4.42065100
H	4.10026500	0.87206700	4.65071600
H	4.64356600	0.27055200	3.06895400
C	-0.45087000	-3.41118900	-1.51746400
C	0.55161400	-3.32482200	-2.50096300

H	1.24321400	-2.48610800	-2.49646400
C	0.65858500	-4.30896500	-3.48262000
H	1.43857400	-4.23376300	-4.23545700
C	-0.24022000	-5.37886100	-3.50575700
H	-0.16172500	-6.13981400	-4.27763800
C	-1.24305000	-5.46326500	-2.53954000
H	-1.94895400	-6.28931800	-2.55604500
C	-1.34951600	-4.48591700	-1.54721800
H	-2.13551900	-4.55876400	-0.80297700
C	-1.91001200	-2.56118400	0.88511800
C	-1.64516700	-3.47182500	1.92325700
H	-0.64623100	-3.87508600	2.05469700
C	-2.66011700	-3.86217900	2.79642900
H	-2.44075400	-4.56636200	3.59480700
C	-3.95002300	-3.34702100	2.64766900
H	-4.73907600	-3.64863600	3.33177100
C	-4.21956800	-2.44453900	1.61890000
H	-5.21884600	-2.03605900	1.49690600
C	-3.20808200	-2.05098500	0.73984400
H	-3.42671000	-1.36237900	-0.06819300
C	-1.08615800	3.47885100	-0.81088500
C	-2.18578900	4.34725500	-0.77675900
H	-3.06031900	4.09533300	-0.18680600
C	-2.16384500	5.54176100	-1.50185800
H	-3.02535300	6.20337000	-1.46910800

C	-1.04465700	5.88563000	-2.25981500
H	-1.03077700	6.81501500	-2.82267600
C	0.05799100	5.02725300	-2.29531000
H	0.93306200	5.28612400	-2.88523900
C	0.03714300	3.82997700	-1.58267500
H	0.89568100	3.16379300	-1.62043300
C	-2.59361700	1.84384600	1.08054700
C	-3.81059700	1.56008200	0.43609200
H	-3.82056100	1.31074400	-0.62098500
C	-5.00520800	1.56712500	1.15748600
H	-5.93924900	1.35255000	0.64505900
C	-5.00008100	1.83490500	2.52828800
H	-5.93123600	1.83262500	3.08886400
C	-3.79305500	2.10077600	3.17695300
H	-3.77996500	2.30702200	4.24397900
C	-2.59616000	2.11009700	2.45889000
H	-1.66640700	2.33021100	2.97291600
O	0.35280500	-0.18293200	1.70268400
P	-0.57592500	-2.04833200	-0.28489300
P	-1.04808200	1.86061400	0.07736400
C	0.93277600	-2.25952500	0.75021000
H	1.59774800	-4.16553700	-0.00435000
C	2.92383600	-3.40893700	1.51543300
C	1.79681800	-3.35862900	0.69269800
H	3.59198700	-4.26377700	1.46294400

C	3.19876900	-2.36828000	2.40491700
H	4.08257200	-2.43195800	3.03107400
C	2.35211900	-1.25722200	2.49973600
C	1.23499400	-1.24144100	1.66111700
C	2.50779700	0.32244800	-1.45246900
Cl	-3.02199900	-0.47665500	-2.36010600
C	3.92706100	0.47612600	-1.40789400
C	4.73736400	-0.44179700	-0.70574700
C	4.55826600	1.55317300	-2.06572600
C	6.12083100	-0.28204700	-0.66352800
H	4.26598600	-1.27908600	-0.20100000
C	5.94174600	1.70729100	-2.01814000
H	3.94664500	2.26297000	-2.61505500
C	6.73120500	0.79208800	-1.31689200
H	6.72728000	-1.00242900	-0.11959200
H	6.40665500	2.54455500	-2.53282700
H	7.81072000	0.91297700	-1.28260600
C	1.28969700	0.17955300	-1.47654200

S5

Zero-point correction= 0.087653 (Hartree/Particle)

Thermal correction to Energy= 0.092021

Thermal correction to Enthalpy= 0.092966

Thermal correction to Gibbs Free Energy= 0.059609

Sum of electronic and zero-point Energies= -231.468665

Sum of electronic and thermal Energies= -231.464296

Sum of electronic and thermal Enthalpies= -231.463352

Sum of electronic and thermal Free Energies= -231.496708

Cartesian coordinates

ATOM	X	Y	Z
C	0.00017000	-1.40023300	0.00000000
C	1.22677300	-0.77197600	0.00000000
C	-1.22669700	-0.77201100	0.00000000
C	1.21445800	0.63289000	0.00000000
H	2.16330900	-1.32340500	0.00000000
C	-1.21465100	0.63256600	0.00000000
H	-2.16292000	-1.32398500	0.00000000
C	-0.00005100	1.32494700	0.00000000
H	2.15485100	1.17949100	0.00000000
H	-2.15496300	1.17930700	0.00000000
H	-0.00029200	2.41150100	0.00000000

S6

Zero-point correction= 0.029838 (Hartree/Particle)

Thermal correction to Energy= 0.032943

Thermal correction to Enthalpy= 0.033887

Thermal correction to Gibbs Free Energy= 0.011049

Sum of electronic and zero-point Energies= -39.807680

Sum of electronic and thermal Energies= -39.804574

Sum of electronic and thermal Enthalpies= -39.803630

Sum of electronic and thermal Free Energies= -39.826469

Cartesian coordinates

ATOM	X	Y	Z
C	0.00000000	0.00000000	-0.00015200
H	0.00000000	1.08286100	0.00030300
H	-0.93778600	-0.54143100	0.00030300
H	0.93778600	-0.54143100	0.00030300

6. Coordinates of radical structures in figure S1

S1

Cartesian coordinates

ATOM	X	Y	Z
C	1.72245300	-5.17396500	0.53738600
C	2.87493000	-4.91662200	-0.21420300
C	3.10002900	-3.64761000	-0.75435600
C	2.11621300	-2.68096900	-0.52108300
C	1.03829200	-2.84956300	0.34921200
C	0.82701400	-4.14737300	0.84418100
C	3.50287400	-0.89861300	-0.97856900
C	4.61602600	-1.73131500	-1.13792600
C	5.86839700	-1.14007700	-0.95665500
H	6.77408900	-1.72974000	-1.05824500
C	5.96908900	0.22073200	-0.64621900
C	4.82950100	1.01631900	-0.51483800
C	3.54942600	0.46106900	-0.67231200
H	1.54800500	-6.17341000	0.92640400
H	3.59530900	-5.71360700	-0.37024100
H	-0.01724900	-4.34502700	1.49694300
H	6.95036400	0.66677400	-0.50934100
H	4.93815200	2.06803200	-0.27332900
O	2.25054100	-1.45001100	-1.11373700
C	4.34224500	-3.19448600	-1.54187000
C	4.02297800	-3.23623000	-3.06331400

H	3.79461100	-4.26255800	-3.37358300
H	3.16387600	-2.60502300	-3.30653900
H	4.88346800	-2.88128200	-3.64173900
C	5.55157700	-4.10061500	-1.26169600
H	5.81498000	-4.11026400	-0.19853300
H	5.34637400	-5.12905200	-1.57570800
H	6.42450400	-3.76506600	-1.83062100
P	0.20775700	-1.31132800	1.00703200
P	1.93928600	1.42423800	-0.71140400
C	-1.36945200	-2.05873600	1.63404100
C	-1.75493100	-2.00340900	2.98001500
C	-2.23368800	-2.66895700	0.70847800
C	-2.97327800	-2.55099800	3.39307000
H	-1.10527500	-1.53657900	3.71295800
C	-3.44459800	-3.22077500	1.12284700
H	-1.95939900	-2.70678100	-0.34197100
C	-3.81906900	-3.16283400	2.46847500
H	-3.25576800	-2.49950900	4.44143700
H	-4.09977400	-3.68949900	0.39333900
H	-4.76486000	-3.59020600	2.79113600
C	1.17910200	-1.04616400	2.57674400
C	1.19671200	0.25374100	3.10726300
C	1.84874400	-2.06384000	3.27471000
C	1.85161900	0.52729300	4.31036100
H	0.70243400	1.04981900	2.55633300

C	2.51148700	-1.78808300	4.47200400
H	1.86056800	-3.07609900	2.88562300
C	2.51209500	-0.49346600	4.99564800
H	1.85226900	1.54091700	4.70267500
H	3.02924000	-2.58799000	4.99527500
H	3.02861000	-0.28192800	5.92839700
C	2.41602300	2.95492600	0.20885300
C	2.06596100	4.23118700	-0.25828700
C	3.04728900	2.84320800	1.45956500
C	2.35951700	5.36589100	0.50031500
H	1.54391800	4.33480400	-1.20175900
C	3.33877900	3.97994100	2.21513200
H	3.31649500	1.86555800	1.84808800
C	2.99720100	5.24636900	1.73610000
H	2.08038600	6.34614600	0.12299500
H	3.83442400	3.87328500	3.17696400
H	3.22397700	6.13269100	2.32319000
C	2.01735200	1.87336000	-2.51086700
C	3.03637800	2.68611900	-3.03030000
C	1.08444000	1.30812200	-3.39065700
C	3.11163400	2.93941400	-4.40024500
H	3.77277300	3.13111300	-2.36768400
C	1.16999100	1.55075500	-4.76268700
H	0.28273200	0.69690800	-2.98858800
C	2.17974100	2.36924300	-5.27045000

H	3.89987600	3.58058200	-4.78681200
H	0.43637500	1.10913200	-5.43208100
H	2.24020000	2.56502600	-6.33807900
Rh	-0.17465600	0.66040800	-0.15460900
Cl	-0.90536600	2.80036800	-1.07450000
C	-3.38632700	0.69198400	0.26724000
O	-2.29753100	0.23819900	-0.12452000
C	-4.56191300	0.27119700	-0.45187800
C	-5.51144300	-0.09781400	-1.11766100
C	-6.60838500	-0.53647400	-1.91171700
C	-7.93150500	-0.20052600	-1.56022400
C	-6.37918700	-1.31300400	-3.06618600
C	-8.99608200	-0.63363100	-2.34541900
H	-8.10855100	0.39995100	-0.67335200
C	-7.45036300	-1.73998200	-3.84569000
H	-5.35946400	-1.56669900	-3.33805200
C	-8.75906000	-1.40306300	-3.48806700
H	-10.01261300	-0.36948500	-2.06778500
H	-7.26507500	-2.33559300	-4.73511700
H	-9.59241700	-1.73804900	-4.09948400
C	-3.56200400	1.59862600	1.42543500
C	-4.85202200	1.89717500	1.89935900
C	-2.44502800	2.15248300	2.07332600
C	-5.02230000	2.72515600	3.00509300
H	-5.71616300	1.47268700	1.39854800

C	-2.62079400	2.98229400	3.17685100
H	-1.45256400	1.94724100	1.68300600
C	-3.90599400	3.26951000	3.64580600
H	-6.02261500	2.94828100	3.36558300
H	-1.75335900	3.41753300	3.66509300
H	-4.03797400	3.92164100	4.50540900

S2

Cartesian coordinates

ATOM	X	Y	Z
C	-2.32565600	2.28167900	-0.40676100
C	-2.90389800	3.46593200	-0.88907600
H	-2.27623100	4.33430600	-1.05462400
C	-4.26227900	3.54181100	-1.18462200
H	-4.68240000	4.46704900	-1.56890200
C	-5.08200600	2.43168300	-0.98882000
H	-6.13893700	2.50631600	-1.22082400
C	-4.55818000	1.23590900	-0.49143300
C	-3.18208600	1.17885800	-0.22127600
C	-5.40084000	0.00008700	-0.15636300
C	-4.55824500	-1.23577500	-0.49143600
C	-3.18214000	-1.17879200	-0.22131000
C	-2.32575700	-2.28163800	-0.40686100
C	-2.90407800	-3.46586200	-0.88916200
H	-2.27647100	-4.33426700	-1.05475600

C	-4.26247700	-3.54168100	-1.18464500
H	-4.68265300	-4.46690100	-1.56890800
C	-5.08214500	-2.43151700	-0.98881200
H	-6.13908900	-2.50609300	-1.22078200
C	-5.68507600	0.00009700	1.37425200
H	-4.75454300	0.00008100	1.94985400
H	-6.26086000	-0.88989000	1.65415200
H	-6.26082800	0.89010900	1.65414200
C	-6.74681300	0.00010700	-0.90049000
H	-7.34185000	0.87659200	-0.62623700
H	-7.34187000	-0.87636800	-0.62624500
H	-6.61041400	0.00011100	-1.98710100
C	0.15611100	3.64765900	-1.01413000
C	0.30302500	4.93007600	-0.46093500
H	0.01077400	5.11472600	0.56810000
C	0.82949800	5.97610400	-1.22289200
H	0.94090900	6.96214300	-0.77848900
C	1.20859900	5.75718700	-2.54818900
H	1.61845800	6.57166000	-3.14016600
C	1.05968800	4.48626700	-3.10894400
H	1.35105900	4.30839100	-4.14100000
C	0.54311900	3.43635700	-2.34878500
H	0.42544600	2.44846600	-2.78570900
C	-0.36604500	2.74651900	1.68440600
C	0.90929100	3.02273100	2.20806300

H	1.78101300	2.96097200	1.56224800
C	1.07056200	3.39116100	3.54281200
H	2.06480900	3.60672700	3.92537700
C	-0.04084500	3.47589600	4.38625000
H	0.08357100	3.75927200	5.42819800
C	-1.31053300	3.19699600	3.88044900
H	-2.18200900	3.26372300	4.52691200
C	-1.47304000	2.83811100	2.53970000
H	-2.46873300	2.63477200	2.15920000
C	0.15594000	-3.64765700	-1.01448600
C	0.30274400	-4.93013100	-0.46137300
H	0.01049500	-5.11482000	0.56765600
C	0.82911300	-5.97615900	-1.22339300
H	0.94041800	-6.96224000	-0.77905800
C	1.20823800	-5.75719200	-2.54868000
H	1.61801900	-6.57166600	-3.14070800
C	1.05945400	-4.48622200	-3.10934600
H	1.35085700	-4.30829900	-4.14138400
C	0.54297800	-3.43631100	-2.34911900
H	0.42539200	-2.44838600	-2.78598700
C	-0.36598400	-2.74661900	1.68414100
C	0.90935500	-3.02318600	2.20761000
H	1.78099700	-2.96168900	1.56166000
C	1.07071500	-3.39162100	3.54234600
H	2.06495400	-3.60747500	3.92477300

C	-0.04059200	-3.47601000	4.38595000
H	0.08389700	-3.75940000	5.42788600
C	-1.31027500	-3.19674700	3.88033600
H	-2.18167000	-3.26319600	4.52693500
C	-1.47287600	-2.83785700	2.53960100
H	-2.46856200	-2.63422900	2.15923600
O	-2.65268200	0.00001300	0.26608300
P	-0.49862300	2.18595700	-0.07402000
P	-0.49869300	-2.18599500	-0.07425900
Cl	-0.66506700	0.00010200	-2.64403400
Rh	0.24574000	0.00000800	-0.39878200
C	2.68326200	-0.00011700	1.31713500
O	1.42855600	-0.00015500	1.36116800
C	3.34418900	0.00001600	0.05397600
C	3.80321600	0.00012000	-1.07591200
C	4.22669600	0.00019700	-2.43174700
C	5.59533200	0.00018200	-2.76934300
C	3.25095500	0.00030100	-3.45304800
C	5.98096000	0.00027100	-4.10606300
H	6.33963800	0.00010000	-1.97878500
C	3.65274800	0.00039100	-4.78589500
H	2.19653900	0.00030900	-3.18713500
C	5.01182800	0.00037800	-5.11453100
H	7.03624300	0.00025900	-4.36404300
H	2.90108400	0.00047400	-5.57003500

H	5.31754700	0.00044800	-6.15741900
C	3.46927900	-0.00023400	2.56840600
C	4.87399800	-0.00014400	2.53882800
C	2.80036300	-0.00044100	3.80578800
C	5.59992800	-0.00025200	3.72750600
H	5.38725600	0.00001500	1.58198400
C	3.53154200	-0.00055000	4.98962000
H	1.71654800	-0.00051600	3.81641800
C	4.93032800	-0.00045500	4.95407900
H	6.68585800	-0.00017800	3.69813700
H	3.01184700	-0.00071000	5.94363800
H	5.49693800	-0.00053900	5.88145800

S3

Cartesian coordinates

ATOM	X	Y	Z
C	-1.65893700	4.32602500	-2.76679500
C	-2.81362400	3.60352900	-3.08199800
C	-2.97657300	2.29072500	-2.63182700
C	-1.93265700	1.74574000	-1.87539200
C	-0.81590200	2.46787600	-1.44787100
C	-0.67903600	3.77592900	-1.93987800
C	-3.25973300	0.08078600	-0.96045300
C	-4.40983500	0.56977500	-1.59151600
C	-5.63582100	0.20824200	-1.02875100

H	-6.56382100	0.56164200	-1.46665700
C	-5.68502800	-0.61995200	0.09822800
C	-4.51468400	-1.10982900	0.67711800
C	-3.26034600	-0.75579700	0.15598500
H	-1.53997700	5.33938200	-3.13972000
H	-3.58787400	4.07553500	-3.67820100
H	0.17800600	4.37401300	-1.65138800
H	-6.64789300	-0.89334500	0.52066300
H	-4.57599900	-1.75750100	1.54540700
O	-2.02692500	0.44152700	-1.45435900
C	-4.20725800	1.40147600	-2.87098000
C	-3.90583900	0.42963100	-4.04765600
H	-3.72736700	0.99588700	-4.96897800
H	-3.02035900	-0.17905600	-3.84404100
H	-4.75516600	-0.24345300	-4.21092600
C	-5.45288200	2.23186600	-3.21857000
H	-5.70832400	2.93728800	-2.42042200
H	-5.29595800	2.79757500	-4.14237100
H	-6.31450800	1.57963100	-3.39212500
P	0.22831200	1.77141800	-0.07520800
P	-1.62736700	-1.46927600	0.71129800
C	1.71325100	2.87396300	-0.20733700
C	2.10171300	3.73917100	0.82431300
C	2.47326200	2.83992200	-1.38986400
C	3.22429500	4.55896200	0.67718800

H	1.53155600	3.78306700	1.74580400
C	3.58966400	3.66390900	-1.53390000
H	2.19308000	2.16817500	-2.19645900
C	3.96898300	4.52642500	-0.50114600
H	3.51060900	5.22435900	1.48736300
H	4.16375000	3.62998900	-2.45629300
H	4.83901900	5.16771900	-0.61630400
C	-0.64066400	2.42564200	1.42847700
C	-0.25159700	1.90338600	2.67453400
C	-1.63957700	3.41011900	1.39079300
C	-0.83547700	2.36711600	3.85421000
H	0.51027400	1.12893900	2.71483600
C	-2.23280100	3.86184000	2.57197800
H	-1.95723300	3.82923100	0.44215700
C	-1.83023900	3.34621600	3.80527800
H	-0.52004300	1.95275100	4.80765600
H	-3.00902000	4.62130000	2.52566300
H	-2.29125100	3.70312400	4.72257300
C	-1.93636900	-1.81303300	2.49624500
C	-1.43352500	-2.97070600	3.11301600
C	-2.58256700	-0.84911800	3.28879700
C	-1.60124300	-3.16749500	4.48474800
H	-0.89579800	-3.70461100	2.52466400
C	-2.74582400	-1.05054300	4.66015000
H	-2.96340300	0.06213800	2.83819500

C	-2.25953100	-2.21264700	5.26178200
H	-1.20979700	-4.07064100	4.94532800
H	-3.25365800	-0.29627900	5.25562600
H	-2.38896600	-2.37081200	6.32933700
C	-1.77559500	-3.08998900	-0.17049100
C	-2.65120300	-4.09966300	0.25404500
C	-1.06456500	-3.26308900	-1.36639200
C	-2.80033400	-5.26666800	-0.49654300
H	-3.21223000	-3.98615800	1.17659800
C	-1.22636100	-4.42444000	-2.12252500
H	-0.37185300	-2.49416400	-1.69427900
C	-2.09035500	-5.43071300	-1.68751100
H	-3.47299500	-6.04683400	-0.14938800
H	-0.66438900	-4.54634100	-3.04453400
H	-2.20824800	-6.33981200	-2.27147800
Rh	0.67324700	-0.49787700	0.29123100
Cl	1.32700000	-2.74540900	1.05103700
C	2.70115000	-0.61669800	-2.21958900
O	2.10114800	0.03489700	-3.07495100
C	2.47958800	-0.38368400	-0.79369400
C	2.78843600	-0.16020200	0.41048900
C	3.64665400	0.17029700	1.52047100
C	3.38907900	-0.32756800	2.81189500
C	4.76504300	1.00218200	1.31406900
C	4.23839600	-0.00207900	3.86753200

H	2.54200700	-0.99024900	2.95542300
C	5.60379200	1.32504500	2.37817200
H	4.95550200	1.40015900	0.32248000
C	5.34455900	0.82555700	3.65723600
H	4.03717000	-0.40037400	4.85855900
H	6.46175000	1.97024800	2.20792600
H	6.00217600	1.07891000	4.48469200
C	3.70044800	-1.66217200	-2.59649600
C	4.17994600	-2.60350500	-1.67270300
C	4.15000600	-1.69864200	-3.92699300
C	5.10677500	-3.56396400	-2.07942400
H	3.80192500	-2.60029100	-0.65516300
C	5.08175700	-2.65214600	-4.32414500
H	3.75622700	-0.96900700	-4.62722400
C	5.56246500	-3.58619000	-3.39917800
H	5.46803700	-4.29833100	-1.36488100
H	5.43424200	-2.67242300	-5.35206000
H	6.28804300	-4.33341700	-3.71071200

7. Coordinates of radical structures in figure S2

INT1-A’

Cartesian coordinates

ATOM	X	Y	Z
C	1.05625000	-3.00267400	-0.34825300
C	1.22160700	-4.36328800	-0.64116300
H	0.37796700	-4.92941800	-1.02047100
C	2.45236300	-4.99247400	-0.48202500
H	2.56318400	-6.04364600	-0.73163200
C	3.53983800	-4.27157700	0.00556400
H	4.48804700	-4.77904600	0.14281000
C	3.42353700	-2.91516600	0.32481900
C	2.18578700	-2.29296100	0.10766700
C	4.54296300	-2.10070600	0.97963200
C	4.44973500	-0.66320700	0.45989500
C	3.17935200	-0.11617200	0.22782200
C	2.99518700	1.23582600	-0.12780700
C	4.14386200	2.02450500	-0.28473400
H	4.03670100	3.06101900	-0.58488400
C	5.41686700	1.49578000	-0.09693400
H	6.29301500	2.12134400	-0.24036800
C	5.56333900	0.16208800	0.27601200
H	6.56030000	-0.23505300	0.42967600
C	4.29124700	-2.07907900	2.51631500
H	3.31502300	-1.64458900	2.75330700

H	5.06088900	-1.48222800	3.01926100
H	4.31903200	-3.09752000	2.92080600
C	5.92998000	-2.71782700	0.72686900
H	5.98308400	-3.73157500	1.13423900
H	6.70905900	-2.14269300	1.23550300
H	6.16886300	-2.75908400	-0.34092300
C	-1.42727900	-3.07294200	-1.96498900
C	-2.05690500	-4.32003800	-1.80325400
H	-2.07209200	-4.80173600	-0.83030200
C	-2.68682600	-4.94057100	-2.88251400
H	-3.16893200	-5.90470000	-2.74277000
C	-2.70424400	-4.32008000	-4.13349400
H	-3.19892200	-4.80169200	-4.97306900
C	-2.09132200	-3.07720300	-4.29911300
H	-2.10531800	-2.58604400	-5.26826600
C	-1.45731000	-2.45203400	-3.22352200
H	-0.96604100	-1.49450100	-3.35773600
C	-1.56956000	-2.83018600	0.88936200
C	-2.95486600	-2.58985100	0.91603200
H	-3.43512700	-2.10155600	0.07258100
C	-3.72495500	-2.98739700	2.00772500
H	-4.79556100	-2.79991200	2.00845100
C	-3.12430000	-3.62692900	3.09538100
H	-3.72428900	-3.93540500	3.94722100
C	-1.75079100	-3.86749300	3.08013600

H	-1.27529700	-4.36780600	3.91975000
C	-0.97749600	-3.47186700	1.98564200
H	0.08895800	-3.67288600	1.98606000
C	1.54471900	3.24498300	-1.74450600
C	2.05203800	4.52555500	-1.46085300
H	2.31230200	4.79709900	-0.44220200
C	2.20381400	5.46732600	-2.47882500
H	2.59749300	6.45333200	-2.24574800
C	1.83996800	5.14580900	-3.78817900
H	1.95292400	5.88137500	-4.58059500
C	1.32104500	3.88211800	-4.07275700
H	1.02497400	3.62926600	-5.08719700
C	1.16882100	2.93423700	-3.05942200
H	0.77120100	1.95150700	-3.28265700
C	1.02456300	3.04253800	1.06811400
C	-0.10637300	3.87871100	1.05592200
H	-0.74222500	3.91566900	0.17545900
C	-0.41265100	4.66605300	2.16430900
H	-1.28719900	5.31086100	2.13997300
C	0.39845400	4.62650100	3.30187100
H	0.15670700	5.23974300	4.16592800
C	1.51799000	3.79528200	3.32369600
H	2.15425200	3.75909800	4.20419100
C	1.83026500	3.00599700	2.21366300
H	2.70730100	2.36711000	2.24189300

O	2.05984800	-0.93228900	0.37705700
P	-0.60728200	-2.20256700	-0.55612100
P	1.31856900	1.98661700	-0.41321200
Cl	1.30493200	-0.47212000	-2.67904000
Rh	-0.02100100	0.09748700	-0.61474800
C	-2.71057700	1.86874200	-0.99329200
O	-2.25613500	2.61948300	-1.85650300
C	-1.83383000	1.00880000	-0.20586300
C	-1.34383300	0.62999000	0.92165200
C	-1.41332500	0.59092900	2.36368000
C	-2.50089600	1.16778000	3.05415200
C	-0.38602200	-0.01112700	3.11391600
C	-2.55406300	1.13648600	4.44432500
H	-3.29591300	1.64334200	2.48844600
C	-0.44585100	-0.04306800	4.50533200
H	0.45332600	-0.45015900	2.58414000
C	-1.52856000	0.52966600	5.17632900
H	-3.39737100	1.58864900	4.96014800
H	0.35596800	-0.51375900	5.06855300
H	-1.57336900	0.50659500	6.26205100
C	-4.18625200	1.83882500	-0.71193000
C	-4.77446500	0.89591100	0.14343300
C	-4.99913600	2.78812500	-1.35089100
C	-6.15272600	0.90846800	0.36286200
H	-4.15161500	0.15248700	0.63044600

C	-6.37348500	2.80045500	-1.12980400
H	-4.52693800	3.50217700	-2.01760900
C	-6.95362300	1.86047400	-0.27141700
H	-6.60160900	0.17359400	1.02599600
H	-6.99592500	3.54011800	-1.62645700
H	-8.02705700	1.86917400	-0.10061900

TS1-A'

Cartesian coordinates

ATOM	X	Y	Z
Rh	0.08748300	0.00295700	-0.27767300
C	1.81268500	-0.91037200	-0.09043500
C	0.96746900	-1.15743200	1.46649800
O	1.13938500	-0.43391200	2.43398300
C	2.97483700	-1.28711300	-0.29697600
C	-2.18186100	5.19582400	-0.13824800
H	-2.95338600	5.95688700	-0.16687900
C	-2.53419300	3.85986600	0.07282600
C	-1.51146500	2.89942100	0.07747000
C	-3.96245400	3.39561700	0.35952400
C	-4.14115000	2.04126600	-0.32835600
C	-3.07091500	1.13682300	-0.29519800
C	-3.18883400	-0.18409400	-0.75717400
C	-4.40498400	-0.55734700	-1.34306100
H	-4.52007100	-1.55845500	-1.74050300

C	-5.46100000	0.34192500	-1.45103000
H	-6.38819100	0.03666000	-1.92720300
C	-5.33203400	1.62727700	-0.93157000
H	-6.17133000	2.31050000	-0.99500400
C	-4.11509200	3.19350600	1.89589300
H	-3.38189600	2.47777100	2.27998000
H	-5.11726100	2.81724700	2.13127300
H	-3.96800900	4.14529000	2.41939600
C	-5.01243500	4.42252100	-0.09679900
H	-4.88315000	5.37053900	0.43378000
H	-6.02137500	4.07174800	0.13932300
H	-4.95497700	4.61549500	-1.17320100
C	2.51994900	2.55548900	-1.12584200
C	3.44212800	3.55601400	-0.77070800
H	3.39838500	4.01502100	0.21181300
C	4.43611600	3.95239000	-1.66653100
H	5.14108200	4.72800700	-1.37894100
C	4.52783600	3.34753800	-2.92177400
H	5.30465100	3.65313200	-3.61803500
C	3.62248600	2.34584800	-3.27611500
H	3.69053100	1.86778700	-4.24949900
C	2.62290300	1.94761100	-2.38640900
H	1.90759100	1.18437600	-2.67022600
C	1.96117600	2.33956600	1.68972800
C	3.20735600	1.76518300	1.99185100

H	3.71133000	1.14617000	1.25700900
C	3.79632700	1.96913500	3.23710400
H	4.76009200	1.51710800	3.45542400
C	3.14696900	2.74067100	4.20417400
H	3.60669900	2.89735900	5.17646400
C	1.90464900	3.30519300	3.91711500
H	1.39116900	3.90544500	4.66368200
C	1.31341800	3.10621400	2.66742600
H	0.34829800	3.55553200	2.45874500
C	-1.96437700	-2.62145700	-1.91550100
C	-2.99654100	-3.56581200	-2.06135300
H	-3.77905100	-3.64410000	-1.31335800
C	-3.01861400	-4.42698000	-3.15929700
H	-3.82228300	-5.15200800	-3.25756200
C	-2.01189000	-4.35803300	-4.12472600
H	-2.03058300	-5.02982500	-4.97892000
C	-0.98358700	-3.42465800	-3.98839700
H	-0.19955500	-3.36146700	-4.73788800
C	-0.95585400	-2.56215700	-2.89121900
H	-0.17030700	-1.81962000	-2.79882700
C	-2.49874400	-2.30936600	0.98135500
C	-2.55071100	-3.70430800	1.11136500
H	-2.20531000	-4.34101800	0.30613400
C	-3.03120900	-4.29244000	2.28363000
H	-3.06401900	-5.37577200	2.36152600

C	-3.45614200	-3.49915400	3.34838100
H	-3.83060000	-3.95889600	4.25910500
C	-3.38484100	-2.10857000	3.24018400
H	-3.69970200	-1.47842000	4.06773500
C	-2.90949500	-1.52007700	2.06996000
H	-2.85553800	-0.43763900	2.00852000
O	-1.84967500	1.55400000	0.24546900
P	1.20138300	2.01004100	0.04292700
P	-1.83349400	-1.43699100	-0.51319600
Cl	-0.62285200	0.92336400	-2.57457200
C	-0.15865400	3.25454200	-0.06134300
H	1.18433100	4.90277600	-0.38932100
C	-0.85158200	5.57246300	-0.30947300
C	0.14901200	4.60835600	-0.25691900
H	-0.59674800	6.61451300	-0.47853300
C	4.29580500	-1.77187800	-0.48516600
C	5.36117200	-0.88317900	-0.75604400
C	4.57544600	-3.15510700	-0.40522700
C	6.65409000	-1.36504800	-0.93913700
H	5.15407300	0.17959700	-0.83731500
C	5.87364900	-3.62467900	-0.58047500
H	3.76386900	-3.84429000	-0.19347300
C	6.91802900	-2.73492300	-0.84959100
H	7.46084900	-0.66861000	-1.15262100
H	6.07255600	-4.69094000	-0.50952000

H	7.92945000	-3.10656300	-0.99017200
C	0.79786500	-2.65094400	1.66333300
C	0.80446100	-3.58951300	0.62124800
C	0.69798100	-3.10724100	2.98535400
C	0.73230500	-4.95446000	0.89813700
H	0.88707000	-3.25058400	-0.40558600
C	0.61777000	-4.47205700	3.26144400
H	0.70429100	-2.37446100	3.78494400
C	0.64213900	-5.40135900	2.21988000
H	0.74975100	-5.67010500	0.07982900
H	0.54610800	-4.80992300	4.29218500
H	0.59268000	-6.46597700	2.43443100

INT2-A'

Cartesian coordinates

ATOM	X	Y	Z
Rh	0.02165800	-0.00255000	-0.15864100
C	1.92863100	-0.29284500	-0.41828600
C	-0.07991700	-0.04466800	1.88809500
O	-1.17583300	0.32369100	2.27959900
C	3.10776000	-0.43525400	-0.71786300
C	-4.24023500	3.36107600	-1.27181100
H	-5.26327500	3.62414500	-1.51492900
C	-3.93965600	2.05644100	-0.86702500
C	-2.60031000	1.74902300	-0.59315600

C	-5.00295900	0.98654300	-0.61683300
C	-4.40863800	-0.36714400	-1.00772100
C	-3.05787900	-0.61117900	-0.72814800
C	-2.48229100	-1.88647100	-0.87408600
C	-3.27940400	-2.90992500	-1.40238200
H	-2.84794100	-3.89391600	-1.55221800
C	-4.60337900	-2.67447400	-1.75799600
H	-5.20390900	-3.47186400	-2.18550800
C	-5.16172200	-1.41579500	-1.54578700
H	-6.20354400	-1.25379900	-1.79747900
C	-5.30070500	0.95653100	0.91250500
H	-4.39407700	0.75481500	1.49146600
H	-6.03571800	0.17544700	1.13935800
H	-5.70653400	1.92170800	1.23714400
C	-6.31517700	1.28233400	-1.36313800
H	-6.73710900	2.23804200	-1.03918100
H	-7.06835000	0.52265000	-1.13513200
H	-6.17088100	1.31506500	-2.44821300
C	1.25885500	3.31229400	-1.24593400
C	1.56794600	4.65104500	-0.94566100
H	1.16584200	5.11945300	-0.05259300
C	2.40996200	5.38441200	-1.78185100
H	2.63948700	6.41897700	-1.54066300
C	2.96245000	4.78607500	-2.91631300
H	3.62329300	5.35600900	-3.56443800

C	2.66951000	3.45348500	-3.21073300
H	3.10101400	2.97991100	-4.08818000
C	1.82225600	2.71358000	-2.38330000
H	1.58424800	1.68348300	-2.62106200
C	0.40638500	3.13800500	1.49148500
C	1.66511900	2.96912400	2.09453100
H	2.43245400	2.38427300	1.59406900
C	1.93088900	3.53644700	3.33928100
H	2.90697300	3.39700000	3.79545700
C	0.94263300	4.26930400	4.00220700
H	1.15006900	4.70816100	4.97454700
C	-0.31280600	4.42881000	3.41553900
H	-1.08822700	4.99127000	3.92888300
C	-0.58261400	3.86572700	2.16536600
H	-1.56332600	3.99517500	1.72021300
C	-0.02468400	-3.44869500	-1.50635100
C	-0.31437500	-4.82061600	-1.39324800
H	-0.95796700	-5.18022500	-0.59628900
C	0.24099000	-5.73426000	-2.28893900
H	0.00785000	-6.79144300	-2.19321700
C	1.09955600	-5.29033500	-3.29731100
H	1.53619900	-6.00321200	-3.99208600
C	1.39996800	-3.93195600	-3.40589800
H	2.07145600	-3.58107000	-4.18449100
C	0.84349400	-3.00928000	-2.51761000

H	1.06473000	-1.95327500	-2.61498400
C	-0.95160900	-3.19748900	1.21674200
C	0.16343500	-3.87305800	1.74294200
H	1.10796200	-3.86385500	1.20676500
C	0.06813700	-4.56394300	2.94937600
H	0.93876800	-5.08238500	3.34131100
C	-1.13757900	-4.58266600	3.65480900
H	-1.21005300	-5.11973400	4.59668100
C	-2.24562300	-3.90458800	3.14705400
H	-3.18637100	-3.91037700	3.69099600
C	-2.15513800	-3.21472100	1.93613600
H	-3.02582400	-2.69296700	1.55378200
O	-2.25891800	0.43497700	-0.25799700
P	0.15048600	2.33894600	-0.14743400
P	-0.74734000	-2.22748700	-0.33807100
Cl	-0.41852300	0.15972500	-2.74113300
C	-1.58855100	2.72744400	-0.62535300
H	-1.16855800	4.78400100	-1.09060500
C	-3.25183000	4.33816200	-1.36479800
C	-1.93993700	4.02353500	-1.02602200
H	-3.50633500	5.34225800	-1.69069300
C	4.48246400	-0.61156100	-1.06207100
C	5.31481800	0.49903500	-1.31806700
C	5.04701600	-1.90155300	-1.15678900
C	6.65628000	0.32270100	-1.64944500

H	4.88959700	1.49663500	-1.25977400
C	6.38947400	-2.07042700	-1.48822400
H	4.41422600	-2.76490000	-0.97220600
C	7.20242700	-0.96087900	-1.73473900
H	7.27944000	1.19243600	-1.84338400
H	6.80344500	-3.07366200	-1.55602600
H	8.24954000	-1.09517400	-1.99309400
C	0.95194300	-0.44370400	2.91167400
C	2.15781200	-1.08514600	2.60676000
C	0.65000500	-0.16263900	4.25805300
C	3.04497500	-1.43901700	3.62578000
H	2.40748900	-1.28975700	1.57361300
C	1.53755800	-0.50976000	5.27106000
H	-0.28850300	0.33255400	4.48216800
C	2.73997100	-1.15242300	4.95682700
H	3.97889100	-1.93512300	3.37458200
H	1.29520300	-0.28157500	6.30601200
H	3.43518400	-1.42497500	5.74724300

TS1-B’

Cartesian coordinates

ATOM	X	Y	Z
Rh	0.33043300	-0.32426900	0.47538300
C	-0.49473900	-2.23614200	0.93262200
C	-0.73407900	-1.75368000	-0.77404200

O	-0.00452300	-2.22595700	-1.65099400
C	-3.29947000	-1.89533300	-1.45250200
C	0.40207500	5.30821400	-0.58518000
H	0.77463800	6.25590000	-0.95730900
C	0.98734400	4.11435400	-1.01246000
C	0.49680200	2.90817300	-0.48796500
C	2.09618000	4.03772300	-2.06218400
C	3.05268800	2.93325500	-1.61157600
C	2.49641800	1.76610200	-1.06417800
C	3.28039700	0.64728800	-0.73712400
C	4.66655300	0.75441500	-0.91921100
H	5.30310300	-0.08095900	-0.65507000
C	5.24713700	1.91958300	-1.40703000
H	6.32533800	1.98382100	-1.52097000
C	4.43971700	2.99790900	-1.76118000
H	4.89861400	3.89449500	-2.16258300
C	1.46379600	3.62283600	-3.42328200
H	0.94383700	2.66329700	-3.34715800
H	2.24200300	3.52996200	-4.18962000
H	0.74123900	4.37930900	-3.75096300
C	2.80991500	5.38555000	-2.25605600
H	2.10478900	6.14928700	-2.59795000
H	3.58020300	5.30622700	-3.02902700
H	3.28036400	5.73633300	-1.33146500
C	-1.95577200	1.52628400	2.67481200

C	-3.04325400	2.35338800	3.01160700
H	-3.60888700	2.85884600	2.23588700
C	-3.43506400	2.51045700	4.34140200
H	-4.27563000	3.15656300	4.58078300
C	-2.75520400	1.83448100	5.35641700
H	-3.06150300	1.95549600	6.39228800
C	-1.68651700	0.99849200	5.03240800
H	-1.15283400	0.46531400	5.81446400
C	-1.28880700	0.84281000	3.70320900
H	-0.44315000	0.21191500	3.45991700
C	-2.98136100	1.36732700	-0.02297100
C	-4.18037500	0.84656300	0.49035800
H	-4.20006200	0.39601700	1.47762300
C	-5.36470100	0.92379300	-0.24330800
H	-6.28282500	0.52177900	0.17581200
C	-5.36953600	1.50405500	-1.51225700
H	-6.29186000	1.56087300	-2.08352000
C	-4.17904400	1.99951300	-2.04540600
H	-4.16847300	2.44805300	-3.03535100
C	-2.99669600	1.93201300	-1.30834800
H	-2.08450300	2.33558400	-1.73570600
C	3.71088000	-1.66785700	1.08033600
C	4.96385100	-2.20414900	0.73015000
H	5.27417900	-2.23724500	-0.30906500
C	5.81123500	-2.73221600	1.70473000

H	6.77648900	-3.13869400	1.41419500
C	5.41561300	-2.74575000	3.04357300
H	6.07479600	-3.15879900	3.80281300
C	4.16755100	-2.23379600	3.39923000
H	3.84847200	-2.24413000	4.43791000
C	3.31910900	-1.70045900	2.42734000
H	2.36221800	-1.28195500	2.71256000
C	2.87451300	-2.01958500	-1.66515900
C	3.11085200	-3.39833800	-1.54938800
H	3.17503400	-3.86127500	-0.56983200
C	3.28791100	-4.19139900	-2.68391900
H	3.47283000	-5.25632100	-2.56973700
C	3.22987000	-3.62094800	-3.95547800
H	3.36800700	-4.23787400	-4.83938000
C	2.99429000	-2.25097800	-4.08315500
H	2.94709000	-1.79479000	-5.06851900
C	2.81683300	-1.45883700	-2.94993400
H	2.63062300	-0.39687900	-3.07160400
O	1.11640700	1.71629700	-0.86299500
P	-1.39089300	1.27088800	0.92852000
P	2.54497000	-0.96439500	-0.17566200
Cl	1.63392900	1.16414600	2.13201300
C	-0.60652800	2.86599300	0.38102900
H	-2.00567000	4.09540500	1.45896900
C	-0.66277700	5.30024300	0.31258900

C	-1.16960100	4.08942500	0.77065800
H	-1.10432200	6.23531000	0.64473200
C	-4.63345200	-2.14777900	-1.88575600
C	-5.60209700	-2.62444000	-0.97951200
C	-5.00067500	-1.94776900	-3.23135500
C	-6.90004500	-2.88892200	-1.40944500
H	-5.31981700	-2.78770800	0.05602200
C	-6.30259600	-2.20861300	-3.65153700
H	-4.25505700	-1.58721100	-3.93288800
C	-7.25632100	-2.67898500	-2.74452800
H	-7.63596500	-3.26118000	-0.70158300
H	-6.57354300	-2.04927600	-4.69189400
H	-8.27004700	-2.88524200	-3.07723900
C	-2.16068900	-1.70056800	-1.06941600
C	0.36248100	-3.35449500	1.04363900
C	-1.61134200	-2.19005500	1.80031300
C	0.17297200	-4.31973600	2.02696700
H	1.14483800	-3.48256000	0.30583100
C	-1.80296700	-3.15502700	2.78625400
H	-2.33659300	-1.39163300	1.69452700
C	-0.90198200	-4.21566700	2.91542400
H	0.85438000	-5.16416300	2.09159100
H	-2.65779300	-3.07995100	3.45362500
H	-1.05481400	-4.97274700	3.68008800

INT2-B’

Cartesian coordinates

ATOM	X	Y	Z
Rh	-0.14184000	0.03273700	0.47555900
C	1.43956800	0.20666800	1.73671400
C	0.89670100	0.06058600	-1.26068000
O	0.23872500	0.00946600	-2.29561200
C	3.54905900	0.12739400	-1.45469500
C	-4.65033500	-2.75892800	-1.39782700
H	-5.64383400	-2.88471000	-1.81286600
C	-4.04270200	-1.50031200	-1.42921800
C	-2.76780100	-1.37298200	-0.85771000
C	-4.64628000	-0.29407000	-2.14879600
C	-4.23004700	0.96768400	-1.39198300
C	-2.94808800	1.02138200	-0.82652300
C	-2.41538200	2.21188100	-0.29753400
C	-3.24326600	3.34125200	-0.26264300
H	-2.86624400	4.26189100	0.16828600
C	-4.54548300	3.29273800	-0.74807900
H	-5.18091600	4.17177500	-0.69516100
C	-5.02354300	2.11687700	-1.32137600
H	-6.02836000	2.10087200	-1.72761400
C	-4.01679900	-0.22620600	-3.57279100
H	-2.92591100	-0.15183300	-3.52176800
H	-4.39743600	0.64851300	-4.11294900

H	-4.27176100	-1.12698900	-4.14322300
C	-6.17400300	-0.40630200	-2.29376700
H	-6.44537400	-1.29785500	-2.86623100
H	-6.57347200	0.44654400	-2.85012700
H	-6.67427300	-0.45185400	-1.32059300
C	-0.32886300	-3.55081800	1.71139800
C	0.48064700	-4.69783900	1.69896300
H	1.12298800	-4.90701500	0.85152700
C	0.46323800	-5.58738300	2.77583200
H	1.09556900	-6.47098900	2.74889500
C	-0.36066600	-5.34549800	3.87493000
H	-0.37251400	-6.03912000	4.71173000
C	-1.16932800	-4.20682900	3.89329500
H	-1.81304700	-4.00690100	4.74569600
C	-1.15734900	-3.31210600	2.82343800
H	-1.77275600	-2.41837100	2.85391300
C	0.71410300	-3.16809000	-0.96201700
C	2.10527900	-3.10987100	-0.77050800
H	2.51180800	-2.57567600	0.08362100
C	2.97033100	-3.72466700	-1.67531700
H	4.04329800	-3.66996800	-1.51397200
C	2.45898400	-4.38713500	-2.79338400
H	3.13338000	-4.85830700	-3.50349100
C	1.07991400	-4.43236700	-3.00105000
H	0.67570900	-4.93712900	-3.87451300

C	0.20996800	-3.82953500	-2.09000600
H	-0.85955900	-3.87319300	-2.26399800
C	-0.63019500	3.50001200	1.69670700
C	-0.70149500	4.89049400	1.49130800
H	-0.74850400	5.29320300	0.48443200
C	-0.68781700	5.76770900	2.57553900
H	-0.74576400	6.83894700	2.40143400
C	-0.59056700	5.26923200	3.87654900
H	-0.57521900	5.95304500	4.72137300
C	-0.50675700	3.89264600	4.08652900
H	-0.42582600	3.49735500	5.09522000
C	-0.52560900	3.00769800	3.00656900
H	-0.48224700	1.93933800	3.17883300
C	0.16812000	3.27397800	-1.07308700
C	1.36711300	3.96052300	-0.81168600
H	1.75867300	4.00501200	0.19983300
C	2.05327900	4.60759600	-1.83918600
H	2.97549300	5.13794500	-1.61748600
C	1.55679800	4.57552700	-3.14379700
H	2.09127900	5.08042800	-3.94402000
C	0.37154200	3.89074500	-3.41434300
H	-0.02050000	3.85648200	-4.42719100
C	-0.31750400	3.24180600	-2.38888000
H	-1.23205100	2.70644200	-2.61822200
O	-2.15780700	-0.12486800	-0.80845500

P	-0.34636900	-2.34817300	0.30374100
P	-0.65969900	2.32507000	0.27789600
Cl	-1.94328700	-0.00977100	2.35939800
C	-2.06825500	-2.48101300	-0.34534300
H	-2.19931200	-4.58703500	0.06684000
C	-4.00234800	-3.86537600	-0.85224700
C	-2.71446400	-3.72611200	-0.34687800
H	-4.49579600	-4.83256000	-0.83353500
C	4.97115600	0.13397900	-1.54226000
C	5.75662800	0.10024500	-0.37126700
C	5.61134700	0.16744500	-2.79797100
C	7.14636500	0.09954400	-0.46166800
H	5.26146200	0.08096400	0.59471600
C	7.00144600	0.16781300	-2.87721000
H	5.00471900	0.19325700	-3.69791400
C	7.77193400	0.13318400	-1.71127800
H	7.74416500	0.07332100	0.44543000
H	7.48600500	0.19448600	-3.84950000
H	8.85673700	0.13280500	-1.77667700
C	2.33648500	0.11156500	-1.34820600
C	2.44384400	1.17410700	1.58180900
C	1.49030700	-0.60857600	2.87894300
C	3.46467700	1.31999000	2.52864400
H	2.45122700	1.82020500	0.71125200
C	2.51576100	-0.47027800	3.82100900

H	0.71232600	-1.33990800	3.06190800
C	3.51033500	0.49386400	3.65260100
H	4.22162300	2.08845000	2.38274300
H	2.52453100	-1.11841700	4.69483900
H	4.30214100	0.60605300	4.38921200

8. Coordinates of radical structures in figure S3

INT1-C

Cartesian coordinates

ATOM	X	Y	Z
P	2.33284000	1.02438000	-0.46869800
P	0.07854900	-1.32734500	-1.26122000
Rh	0.24305400	0.24883200	0.37910600
Cl	0.61861200	1.63032500	2.37985500
C	-2.38267900	1.56506000	-0.87430500
O	-1.81454100	1.75342200	-1.95386200
C	-1.79122900	0.73150500	0.15857500
C	-1.74592500	-0.04018200	1.16571200
C	-2.31552000	-0.80439900	2.24593600
C	-1.55733300	-1.12179300	3.38913100
C	-3.65095900	-1.25140200	2.16156000
C	-2.12811200	-1.85962900	4.42344000
H	-0.54123700	-0.74782000	3.45737700
C	-4.20698900	-2.00155400	3.19499400
H	-4.23554500	-1.01221700	1.27864800
C	-3.44925800	-2.30639400	4.32925200
H	-1.53884100	-2.08729300	5.30771000
H	-5.23512500	-2.34540100	3.11668900
H	-3.88745500	-2.88739900	5.13651100
C	-3.72270700	2.17597700	-0.59692800
C	-4.29801600	2.18695500	0.68251800

C	-4.40798500	2.77454600	-1.66632500
C	-5.54470100	2.77919000	0.88450300
H	-3.76465800	1.74790200	1.51892700
C	-5.65426600	3.35991200	-1.46299500
H	-3.94091000	2.76868900	-2.64561900
C	-6.22576600	3.36238100	-0.18631100
H	-5.98094200	2.78996000	1.87952800
H	-6.18123100	3.81732400	-2.29623600
H	-7.19753000	3.82247500	-0.02666400
C	2.23360200	-0.49077500	-2.95153900
H	2.82359500	-1.29029600	-2.48737100
H	2.55441700	-0.44946800	-4.00099400
C	0.73970400	-0.86136000	-2.93879900
H	0.55760800	-1.69494200	-3.62693200
H	0.14364800	-0.00865500	-3.28308600
C	2.55809300	0.86970800	-2.31409700
H	3.57794400	1.18193600	-2.56875800
H	1.89276600	1.63526100	-2.73145800
C	2.58423600	2.84191800	-0.29614300
C	3.85023900	3.40306300	-0.08143200
C	1.47643500	3.68708000	-0.46176900
C	4.00590900	4.79009300	-0.03392400
H	4.71528300	2.76159900	0.05750400
C	1.63812200	5.07155900	-0.42366400
H	0.48944600	3.25834200	-0.60772200

C	2.90178000	5.62579400	-0.20647700
H	4.99086300	5.21509300	0.14145200
H	0.77272700	5.71666800	-0.54888900
H	3.02397500	6.70507600	-0.16619600
C	3.83774900	0.26598300	0.28162300
C	4.89652800	-0.26574100	-0.46988300
C	3.90175700	0.20050400	1.68619400
C	5.99568700	-0.85029400	0.16505800
H	4.87836700	-0.23091600	-1.55443800
C	5.00495600	-0.37607100	2.31496600
H	3.08432000	0.60726900	2.27694100
C	6.05330000	-0.90577400	1.55757800
H	6.80582700	-1.26004900	-0.43258500
H	5.04344400	-0.41202400	3.40054200
H	6.90977400	-1.35852200	2.05033100
C	1.05311600	-2.84562900	-0.85269000
C	1.11420600	-3.93858000	-1.73445900
C	1.76061300	-2.91172300	0.35589200
C	1.87132400	-5.06511000	-1.41598800
H	0.55668900	-3.91837600	-2.66707800
C	2.51821700	-4.04158700	0.67561500
H	1.71779900	-2.07391600	1.04658500
C	2.57632400	-5.11770900	-0.20974400
H	1.90870700	-5.90343500	-2.10669300
H	3.06349000	-4.07418500	1.61467200

H	3.16594300	-5.99658100	0.03743500
C	-1.58932300	-2.01376600	-1.66150800
C	-2.38865000	-1.44932200	-2.66792400
C	-2.09887300	-3.08976500	-0.91704400
C	-3.65854200	-1.96454200	-2.93537200
H	-2.03831200	-0.59129900	-3.23121800
C	-3.36942500	-3.60057400	-1.18487100
H	-1.50021700	-3.53635300	-0.12921700
C	-4.15128800	-3.04238000	-2.19806200
H	-4.26329800	-1.51767500	-3.71999000
H	-3.74607500	-4.43568600	-0.60036800
H	-5.13971200	-3.44201000	-2.40873500

INT2-C

Cartesian coordinates

ATOM	X	Y	Z
P	-2.58213100	-0.15930500	0.27269900
P	0.54795100	-1.26690200	1.34568500
Rh	-0.22782000	0.22531700	-0.27201900
Cl	-1.05025000	1.14006800	-2.37487400
C	-0.18738900	1.77875400	0.99599000
O	-0.61204900	1.61387000	2.12781000
C	1.66949200	0.37670100	-0.89765900
C	2.81510500	0.41232700	-1.33612600
C	4.13370200	0.46437000	-1.88118300

C	4.33267800	0.68042900	-3.26162000
C	5.26874100	0.30407400	-1.05850500
C	5.61812200	0.73282500	-3.79529500
H	3.46407100	0.80709800	-3.90100800
C	6.55142900	0.35931500	-1.59933000
H	5.12599900	0.13839500	0.00507700
C	6.73356700	0.57308900	-2.96834000
H	5.75084300	0.90088400	-4.86122800
H	7.41436000	0.23589000	-0.94930700
H	7.73571300	0.61602700	-3.38700100
C	0.31255600	3.12468800	0.56425400
C	0.80927000	3.42599800	-0.71147900
C	0.26298800	4.14157000	1.54138600
C	1.24781200	4.71906500	-1.00012600
H	0.84508900	2.66026900	-1.47310400
C	0.70349400	5.42623400	1.24714400
H	-0.12422400	3.90074000	2.52526100
C	1.19827700	5.71833700	-0.02843000
H	1.62882200	4.94109700	-1.99278600
H	0.66200000	6.20038800	2.00888400
H	1.54286200	6.72282500	-0.26112000
C	-2.03925700	-1.88348600	2.49820100
H	-2.09713900	-2.71015000	1.77912500
H	-2.48073500	-2.26549100	3.42798200
C	-0.57297700	-1.52279700	2.80546900

H	-0.11611300	-2.28579700	3.44559100
H	-0.53382100	-0.58082100	3.36032400
C	-2.90510400	-0.70164400	2.02605900
H	-3.96592200	-0.95751800	2.12013700
H	-2.72126900	0.16297500	2.66944400
C	2.16114500	-0.95205800	2.17902800
C	3.26233200	-1.79880900	1.99046000
C	2.29261300	0.16017300	3.02789600
C	4.46975300	-1.54348100	2.64444800
H	3.18403000	-2.65601400	1.33073200
C	3.49981600	0.40940700	3.67994700
H	1.45966700	0.84060000	3.17093100
C	4.59155900	-0.44111700	3.49107800
H	5.31446200	-2.20908100	2.48814300
H	3.58779700	1.27403900	4.33218900
H	5.53177400	-0.24333900	3.99879200
C	0.72239700	-2.92874800	0.56754300
C	0.43181200	-4.11565800	1.26144100
C	1.16339200	-3.01503800	-0.76493900
C	0.56859600	-5.35569300	0.63629100
H	0.10180400	-4.08614800	2.29463900
C	1.30568100	-4.25845600	-1.38374400
H	1.41568200	-2.10820000	-1.30750000
C	1.00379000	-5.43017500	-0.68819800
H	0.33594400	-6.26311600	1.18708000

H	1.65340700	-4.30625100	-2.41198600
H	1.10951100	-6.39682900	-1.17325500
C	-3.72685000	1.26943300	0.07526500
C	-4.46258500	1.43054900	-1.10904800
C	-3.82344100	2.25340800	1.07321500
C	-5.29446800	2.53729700	-1.27926500
H	-4.37994900	0.69627100	-1.90237500
C	-4.65810000	3.35799400	0.89870100
H	-3.23550300	2.17676800	1.98190500
C	-5.39860500	3.50148800	-0.27567300
H	-5.85619300	2.64721000	-2.20292300
H	-4.72400100	4.10872500	1.68177700
H	-6.04671800	4.36330600	-0.41068900
C	-3.36207300	-1.50791800	-0.71332700
C	-4.66576000	-1.95521300	-0.43611000
C	-2.64501100	-2.11618900	-1.75337200
C	-5.23042100	-2.99602100	-1.17144800
H	-5.25215500	-1.47945900	0.34519600
C	-3.21216500	-3.15941200	-2.48982000
H	-1.65385200	-1.75461200	-2.00643300
C	-4.50158500	-3.60343000	-2.19807700
H	-6.23991600	-3.33005300	-0.94672400
H	-2.64443400	-3.61768600	-3.29478700
H	-4.94261500	-4.41432300	-2.77176600

INT3-C

Cartesian coordinates

ATOM	X	Y	Z
P	-1.93049900	-1.09718200	0.52637900
P	1.40233100	-0.65885300	1.35784800
Rh	0.23277600	-0.79921700	-0.79878300
Cl	0.62712000	-3.28211200	-0.65666100
C	-0.59152900	-0.92820200	-2.52152900
O	-1.06305800	-0.96649200	-3.56279100
C	-0.09980700	1.16027700	-0.95810500
C	-0.30596800	2.34498100	-1.17993600
C	-0.49829000	3.73980000	-1.42632000
C	-1.16250100	4.56434500	-0.49490800
C	-0.00958400	4.32893700	-2.61169200
C	-1.32663600	5.92600300	-0.74133300
H	-1.55212900	4.11817200	0.41420400
C	-0.17925800	5.69048200	-2.85216100
H	0.50330900	3.70201000	-3.33515400
C	-0.83658500	6.49678200	-1.91892700
H	-1.84311000	6.54529800	-0.01166700
H	0.20452500	6.12484300	-3.77198000
H	-0.96801900	7.55874200	-2.10884000
C	2.03100800	-0.55154200	-1.90041000
C	2.78708200	0.62630000	-1.84138900
C	2.47778800	-1.58285600	-2.73814600

C	3.97692100	0.75502000	-2.56486000
H	2.45105500	1.46152200	-1.23874600
C	3.66339000	-1.44918600	-3.47018700
H	1.92083400	-2.51039800	-2.80795600
C	4.42281000	-0.28285300	-3.38322300
H	4.55088800	1.67607300	-2.48723800
H	3.98791300	-2.26716300	-4.11011600
H	5.34496800	-0.18110800	-3.95002200
C	-0.53137400	-2.57951100	2.52213300
H	-0.66195000	-3.37765200	1.78700600
H	-0.67946500	-3.03740500	3.50846300
C	0.93899700	-2.09923800	2.45540400
H	1.55005600	-2.91889100	2.07008500
H	1.32821700	-1.86909800	3.45350400
C	-1.63150200	-1.51972200	2.32383700
H	-2.57978200	-1.88565000	2.73198000
H	-1.38655400	-0.60104800	2.86208300
C	-3.02881000	0.38517700	0.60974600
C	-2.89186700	1.36031700	1.60930000
C	-3.98666400	0.58870400	-0.39791400
C	-3.71637100	2.48749900	1.62054200
H	-2.13828700	1.26286500	2.38343200
C	-4.80099900	1.72034600	-0.39142400
H	-4.10844800	-0.14634400	-1.18794400
C	-4.67425600	2.67105800	0.62308500

H	-3.60410600	3.22344700	2.41253800
H	-5.53588400	1.85678200	-1.18003100
H	-5.31243900	3.55038100	0.63178100
C	-3.11231200	-2.42545200	0.01227400
C	-4.35869500	-2.53791400	0.65690900
C	-2.79548200	-3.33114800	-1.00853400
C	-5.26320400	-3.53074000	0.28662500
H	-4.63432300	-1.83448700	1.43789500
C	-3.70632400	-4.32534600	-1.37915700
H	-1.82519200	-3.28757700	-1.48864500
C	-4.93842400	-4.42735800	-0.73589400
H	-6.22261800	-3.60164500	0.79215900
H	-3.44218200	-5.02193200	-2.16999900
H	-5.64456500	-5.20083500	-1.02614600
C	1.11779600	0.85566900	2.37848200
C	1.34425700	2.11504000	1.79686100
C	0.71629100	0.80512900	3.72414800
C	1.16950900	3.28493700	2.53688200
H	1.64400500	2.18477600	0.75931100
C	0.53330700	1.97760200	4.46176600
H	0.54766900	-0.14576700	4.21729700
C	0.75859900	3.22059600	3.86972000
H	1.34462300	4.24634400	2.06281500
H	0.22136800	1.91389400	5.50085200
H	0.61797500	4.13269200	4.44354300

C	3.23837000	-0.83595400	1.34195400
C	4.08066600	0.01193300	2.07506800
C	3.79962200	-1.89263200	0.60438300
C	5.46219200	-0.19334300	2.07109800
H	3.66755100	0.83333600	2.65013900
C	5.17910300	-2.09338000	0.60867900
H	3.15908000	-2.54638800	0.02088300
C	6.01398400	-1.24477300	1.33943600
H	6.10408400	0.47216500	2.64237600
H	5.60096500	-2.91015600	0.02968400
H	7.08947100	-1.40135900	1.33614400

INT4-C

Cartesian coordinates

ATOM	X	Y	Z
P	2.37906700	-0.38249200	0.43937200
P	-0.89273700	-1.68608800	0.96494200
Rh	0.07257300	0.45985000	-0.01048100
Cl	-0.07164000	1.26914900	2.31897700
C	0.04935900	-0.05863200	-1.80620800
O	-0.01886100	-0.33240700	-2.91761900
C	-1.78332000	1.13568000	-0.39651700
C	-2.89616800	1.55009400	-0.68476000
C	-4.20348600	2.05167200	-0.96652400
C	-4.94134900	2.73546300	0.02207700

C	-4.78993700	1.87945500	-2.23718300
C	-6.21598100	3.22528700	-0.25273300
H	-4.49612300	2.87948700	1.00197900
C	-6.06531000	2.37143900	-2.50540400
H	-4.22895800	1.35919900	-3.00814000
C	-6.78553400	3.04594500	-1.51613700
H	-6.76722600	3.75184300	0.52254400
H	-6.49869800	2.22942900	-3.49238900
H	-7.77986800	3.42993100	-1.72834700
C	0.79293000	2.32761600	-0.68440000
C	0.27140900	3.49979600	-0.12011900
C	1.77375000	2.44500800	-1.67784400
C	0.75018200	4.75218800	-0.51594700
H	-0.50106400	3.43929400	0.63665000
C	2.24985800	3.70124700	-2.07530100
H	2.19405800	1.56526200	-2.15666700
C	1.74311100	4.86064300	-1.49235000
H	0.33535600	5.64799900	-0.05881900
H	3.01617500	3.76297300	-2.84514900
H	2.10912600	5.83713500	-1.79958400
C	1.48354800	-1.91223400	2.67750500
H	1.50547100	-0.90444000	3.10163400
H	1.90600500	-2.57888000	3.44008500
C	0.01162800	-2.32586900	2.47597800
H	-0.57198000	-1.94036500	3.31694100

H	-0.10100800	-3.41653500	2.49909800
C	2.38671100	-1.96872000	1.42946000
H	3.42334300	-2.16185300	1.72223400
H	2.09732500	-2.78714000	0.76306400
C	3.29773000	-0.88331100	-1.08645100
C	2.85822800	-1.99603500	-1.82559900
C	4.38242900	-0.13827400	-1.57535600
C	3.50182100	-2.36513100	-3.00678300
H	2.00378300	-2.57943200	-1.49262600
C	5.02078700	-0.50714800	-2.76151000
H	4.73114200	0.73230500	-1.03059700
C	4.58663300	-1.62263900	-3.47801400
H	3.14917200	-3.23080200	-3.56073300
H	5.85909800	0.08204700	-3.12333100
H	5.08530500	-1.90901000	-4.39988200
C	3.57864300	0.66819300	1.36367500
C	4.84832800	0.15951300	1.69647200
C	3.24867500	1.97538600	1.74603200
C	5.76312100	0.93992300	2.40040400
H	5.13791200	-0.84194900	1.38983600
C	4.16964600	2.75413000	2.45233100
H	2.27380500	2.37828300	1.50634600
C	5.42292300	2.24098700	2.78213600
H	6.74008200	0.53369000	2.64849000
H	3.89704600	3.76410000	2.74483600

H	6.13540100	2.84953100	3.33286300
C	-0.89836100	-3.06931200	-0.26130500
C	-1.68773500	-2.89656900	-1.41460300
C	-0.14287400	-4.24539800	-0.14125900
C	-1.71944900	-3.87021300	-2.41074000
H	-2.28878200	-1.99750600	-1.52597200
C	-0.16862100	-5.21825600	-1.14639300
H	0.46756300	-4.42369000	0.73829700
C	-0.95570700	-5.03416400	-2.28222700
H	-2.33961600	-3.71821100	-3.28977000
H	0.42378900	-6.12226300	-1.03219700
H	-0.97756200	-5.79132800	-3.06112900
C	-2.63537500	-1.69172800	1.57337400
C	-3.41392300	-2.86019200	1.53593100
C	-3.17125800	-0.52878800	2.14860600
C	-4.70618100	-2.86500200	2.06275400
H	-3.01917700	-3.76830500	1.09100500
C	-4.46373200	-0.54223200	2.67564000
H	-2.57886300	0.37782600	2.18128200
C	-5.23419700	-1.70515800	2.63236500
H	-5.29869500	-3.77540000	2.02458900
H	-4.87044500	0.36559500	3.11253700
H	-6.24258500	-1.70760400	3.03787400

INT5-C

Cartesian coordinates

ATOM	X	Y	Z
C	0.63319800	2.29717700	-0.24350300
C	-0.63320300	2.29714700	-0.24347700
C	1.92602900	2.94082400	-0.24161900
O	0.00003300	0.04132100	-3.09870800
Cl	0.00008400	0.41600100	2.36620700
C	-1.92606000	2.94074700	-0.24153800
C	-2.27842700	3.83623100	-1.27093200
C	-2.85012600	2.69468500	0.79267400
C	-3.52004400	4.47001400	-1.26173500
H	-1.56982400	4.02943300	-2.07138300
C	-4.08486300	3.33927800	0.79807600
H	-2.57584700	2.00611000	1.58523400
C	-4.42711900	4.22571600	-0.22769700
H	-3.77753800	5.15916900	-2.06208100
H	-4.78425300	3.14399300	1.60659600
H	-5.39370900	4.72265100	-0.22126800
C	0.00003300	0.08072000	-1.94392200
C	2.27833900	3.83627200	-1.27106500
C	2.85012100	2.69486000	0.79259200
C	3.51992400	4.47011700	-1.26191700
H	1.56971500	4.02939900	-2.07151600
C	4.08482500	3.33951800	0.79794500
H	2.57588700	2.00631400	1.58519300

C	4.42702500	4.22592000	-0.22787700
H	3.77737300	5.15924400	-2.06230300
H	4.78423400	3.14431200	1.60646700
H	5.39358800	4.72290600	-0.22148500
P	1.88907300	-1.40837900	0.15463400
P	-1.88908000	-1.40832100	0.15460000
Rh	0.00003100	0.19786900	-0.09958800
C	-0.00004500	-3.24501000	1.38736900
H	-0.00005500	-2.50471600	2.19295400
H	-0.00007000	-4.23129100	1.86945600
C	-1.30066300	-3.14329800	0.56195300
H	-2.11017600	-3.66069000	1.08888200
H	-1.15893600	-3.66057400	-0.39462600
C	1.30062400	-3.14333300	0.56202800
H	2.11010500	-3.66070600	1.08902600
H	1.15895300	-3.66064900	-0.39453800
C	-3.16931800	-1.10920400	1.45865000
C	-4.43061000	-0.58575900	1.13250000
C	-2.88110700	-1.37243400	2.80891600
C	-5.38196600	-0.34793200	2.12615100
H	-4.68105600	-0.36799400	0.10003200
C	-3.83588600	-1.13767400	3.79861900
H	-1.90504900	-1.74194300	3.09877200
C	-5.08990000	-0.62553000	3.46205300
H	-6.35480700	0.05042200	1.84979100

H	-3.59224400	-1.34867300	4.83645500
H	-5.83163400	-0.44192000	4.23486200
C	-2.93839000	-1.67496300	-1.34623600
C	-3.21051700	-0.57934700	-2.18199800
C	-3.50968000	-2.91811900	-1.66489000
C	-4.02408700	-0.72386000	-3.30754900
H	-2.79309800	0.39547100	-1.94621400
C	-4.31121400	-3.06516800	-2.79778900
H	-3.34229600	-3.77987800	-1.02654200
C	-4.57003600	-1.96877900	-3.62267900
H	-4.22321600	0.13705700	-3.93979800
H	-4.73868700	-4.03676800	-3.03142300
H	-5.19518500	-2.08435900	-4.50397900
C	3.16928400	-1.10921200	1.45869200
C	4.43046100	-0.58548100	1.13256200
C	2.88116600	-1.37265700	2.80893400
C	5.38180200	-0.34758400	2.12621000
H	4.68082900	-0.36754700	0.10011100
C	3.83592900	-1.13782600	3.79863600
H	1.90519500	-1.74241000	3.09877800
C	5.08983100	-0.62539400	3.46208900
H	6.35455700	0.05099100	1.84986700
H	3.59236300	-1.34899500	4.83645500
H	5.83155300	-0.44172900	4.23489700
C	2.93841900	-1.67508100	-1.34616800

C	3.21053400	-0.57951000	-2.18199000
C	3.50974900	-2.91824100	-1.66473500
C	4.02413000	-0.72406800	-3.30751800
H	2.79308600	0.39531200	-1.94627400
C	4.31130900	-3.06533700	-2.79760900
H	3.34237600	-3.77996600	-1.02633800
C	4.57011700	-1.96899100	-3.62256200
H	4.22324600	0.13681500	-3.93981600
H	4.73881300	-4.03694000	-3.03117500
H	5.19528700	-2.08460900	-4.50384300

CAT-2a-C

Cartesian coordinates

ATOM	X	Y	Z
Cl	-1.01379300	1.38181200	2.50381100
C	1.78943300	0.06611900	1.59751200
C	1.90259300	1.19921300	1.08093500
C	2.50864700	2.47950200	0.82612700
C	3.85153700	2.69632200	1.19828500
C	1.79946700	3.52764000	0.21305200
C	4.45868000	3.92702000	0.96499500
H	4.40493900	1.89144300	1.67246500
C	2.41449000	4.75652300	-0.02023300
H	0.76460600	3.35897100	-0.06521200
C	3.74468800	4.96205600	0.35313600

H	5.49299800	4.07990800	1.26218800
H	1.85099100	5.55858500	-0.49002800
H	4.22145300	5.92179200	0.17263200
C	2.00985500	-1.14464300	2.33911200
C	1.02320100	-1.65421800	3.20757100
C	3.23559200	-1.83020600	2.21461900
C	1.26630600	-2.81901000	3.93153200
H	0.09075500	-1.10825200	3.31008900
C	3.46527100	-2.99623200	2.94228700
H	3.99403800	-1.44199400	1.54178900
C	2.48288500	-3.49570700	3.80106300
H	0.50295000	-3.19873600	4.60570900
H	4.41435500	-3.51602000	2.83871100
H	2.66538900	-4.40507000	4.36763800
P	-2.25937700	0.37037300	-0.47515600
P	0.76879800	-0.47491800	-1.46846400
Rh	-0.07044700	0.46438500	0.43435300
C	-1.23808300	0.91848700	-3.03148800
H	-1.16908700	1.90275200	-2.55247200
H	-1.55798100	1.10726900	-4.06417600
C	0.15718900	0.27026800	-3.08326800
H	0.89273400	1.02034100	-3.38908700
H	0.17441700	-0.51433500	-3.84804900
C	-2.30877300	0.06747800	-2.31919400
H	-3.30807500	0.31626600	-2.69117000

H	-2.13797200	-0.99636300	-2.51725200
C	2.60118200	-0.47109900	-1.74190700
C	3.37912600	-1.62284100	-1.54978600
C	3.24963100	0.72527500	-2.09013400
C	4.76468700	-1.58360200	-1.72498900
H	2.90625600	-2.55645200	-1.26476100
C	4.63239900	0.76283600	-2.26806400
H	2.68178900	1.64409500	-2.20472900
C	5.39491300	-0.39342000	-2.08983100
H	5.34894300	-2.48829600	-1.57771000
H	5.11286000	1.69957700	-2.53672700
H	6.47232900	-0.36464500	-2.22885800
C	0.33595300	-2.26978700	-1.65442300
C	0.56655500	-2.98135200	-2.84522800
C	-0.22360700	-2.94919000	-0.56249600
C	0.22944500	-4.33068900	-2.94464400
H	1.02934500	-2.48872400	-3.69619400
C	-0.56132500	-4.30184600	-0.66197100
H	-0.39175500	-2.41066500	0.36583000
C	-0.33980500	-4.99303100	-1.85281300
H	0.41405900	-4.86623600	-3.87228900
H	-0.99812500	-4.80979200	0.19324000
H	-0.60397300	-6.04431000	-1.93197100
C	-3.25707800	1.92246300	-0.36946000
C	-2.65379700	3.12811500	0.01085600

C	-4.61754700	1.91960600	-0.71893200
C	-3.39383500	4.31322700	0.02361200
H	-1.61893400	3.12565500	0.33383800
C	-5.35506600	3.10256500	-0.70122000
H	-5.10850200	0.98844800	-0.99044300
C	-4.74207900	4.30363300	-0.33331600
H	-2.91575400	5.24044200	0.32788400
H	-6.40871200	3.08598700	-0.96765100
H	-5.31804300	5.22524700	-0.31613800
C	-3.37717400	-0.92040100	0.22767000
C	-4.07245700	-1.84153900	-0.57171700
C	-3.53500600	-0.97714500	1.62418200
C	-4.90771900	-2.79875300	0.01021500
H	-3.97373900	-1.82467200	-1.65194400
C	-4.37432200	-1.93136200	2.19842300
H	-2.99104600	-0.27627800	2.25176700
C	-5.06146400	-2.84560300	1.39562300
H	-5.43713700	-3.50534900	-0.62381500
H	-4.48835600	-1.96086600	3.27886100
H	-5.71226700	-3.58970700	1.84752700

TS1-C

Cartesian coordinates

ATOM	X	Y	Z
C	-1.28968800	1.33342300	-0.94307100

O	-1.52001200	0.81559900	-2.02509800
Rh	0.10461100	0.24933400	0.28243200
C	-1.89733100	0.44841500	0.56296900
C	-3.07415100	0.46427200	0.93513600
C	-4.42885500	0.51961400	1.35688100
C	-4.80718600	1.34347900	2.43979000
C	-5.42022800	-0.24233700	0.70073700
C	-6.13541300	1.39974500	2.85105600
H	-4.04487600	1.92652000	2.94704900
C	-6.74682700	-0.17490100	1.11647800
H	-5.13107500	-0.87762300	-0.13009300
C	-7.10856500	0.64322200	2.19101200
H	-6.41442800	2.03537600	3.68706400
H	-7.50283700	-0.76269700	0.60254800
H	-8.14537000	0.69105600	2.51333500
C	-1.34322400	2.83032200	-0.78545000
C	-1.50013600	3.49637500	0.43653000
C	-1.25289900	3.58078100	-1.97148600
C	-1.56077900	4.88920900	0.46957300
H	-1.55234700	2.92603800	1.35421500
C	-1.30226900	4.97244300	-1.93194200
H	-1.15302300	3.05623400	-2.91559600
C	-1.45716700	5.63139000	-0.70930000
H	-1.68197000	5.39487700	1.42358600
H	-1.22724200	5.54159200	-2.85493000

H	-1.50035600	6.71715100	-0.67622800
P	2.41336700	0.33888300	-0.33422800
P	-0.19865900	-1.67585800	-0.98572900
Cl	0.62560300	1.68934900	2.20325400
C	2.19555400	-1.55416400	-2.54965900
H	2.59693400	-2.34977900	-1.91142800
H	2.59330400	-1.74441700	-3.55558200
C	0.66398700	-1.67826200	-2.63228500
H	0.39794500	-2.59618700	-3.16898000
H	0.24263800	-0.83754400	-3.19411900
C	2.72763400	-0.18230900	-2.09990200
H	3.80499700	-0.11676500	-2.29330700
H	2.26349800	0.60800400	-2.70318300
C	0.52623000	-3.13421400	-0.11377700
C	0.69153100	-4.37179300	-0.75966000
C	0.89799300	-3.01949200	1.23278900
C	1.22998000	-5.46130900	-0.07694400
H	0.38815800	-4.49258200	-1.79622100
C	1.43284800	-4.11337600	1.91858300
H	0.76800900	-2.07086100	1.74763200
C	1.60271600	-5.33316700	1.26444000
H	1.35436400	-6.41158500	-0.58954500
H	1.72069800	-4.00488900	2.96027700
H	2.02136700	-6.18370200	1.79584400
C	-1.89088200	-2.29260300	-1.39006700

C	-2.51627700	-1.99969100	-2.61111200
C	-2.57616900	-3.07933400	-0.45055000
C	-3.78847300	-2.50097700	-2.89266400
H	-2.02615500	-1.36485700	-3.33901400
C	-3.84892600	-3.57561800	-0.73220700
H	-2.11347900	-3.31333300	0.50304500
C	-4.45751000	-3.29219400	-1.95730000
H	-4.25639800	-2.26777700	-3.84540900
H	-4.36247300	-4.18585600	0.00588700
H	-5.44619400	-3.68463800	-2.18060000
C	3.58503000	-0.65936400	0.68574800
C	4.55618100	-1.51483300	0.14378000
C	3.49109900	-0.53834300	2.08409800
C	5.41426900	-2.23587000	0.97852200
H	4.65713100	-1.62908400	-0.93086500
C	4.35497500	-1.25384100	2.91270600
H	2.74014900	0.12047400	2.51357800
C	5.31672900	-2.10616900	2.36383600
H	6.15863900	-2.89671800	0.54191400
H	4.27472300	-1.14403700	3.99103500
H	5.98624600	-2.66530500	3.01227900
C	3.19900000	2.00966100	-0.37324800
C	2.40052100	3.15795300	-0.44701300
C	4.59626100	2.14383000	-0.40304200
C	2.98948700	4.41868100	-0.56259300

H	1.32274200	3.06898000	-0.39315000
C	5.18170800	3.40522700	-0.51288900
H	5.23106300	1.26562700	-0.32677400
C	4.37861100	4.54526900	-0.59513700
H	2.35544700	5.29935000	-0.61443600
H	6.26470800	3.49689900	-0.52801400
H	4.83582800	5.52804900	-0.67663900

TS2-C

Cartesian coordinates

ATOM	X	Y	Z
C	0.26147000	0.79279300	2.61481600
O	0.61330700	1.39436000	3.54648300
C	-1.40867000	-0.17331000	2.63755400
C	-1.51648200	2.33294700	-0.49285900
Cl	1.45643700	-1.89007800	1.92868800
C	-2.23536500	3.38123100	-1.15029500
C	-3.50420700	3.14188700	-1.71816000
C	-1.69018500	4.67809500	-1.25130400
C	-4.19939900	4.16427100	-2.36034500
H	-3.93281100	2.14613600	-1.64834400
C	-2.39147900	5.69542700	-1.89472400
H	-0.71209200	4.87249700	-0.82077200
C	-3.64838200	5.44512400	-2.45162300
H	-5.17626700	3.96030300	-2.79157100

H	-1.95474400	6.68867200	-1.96187900
H	-4.19330400	6.24080200	-2.95246700
C	-0.90153700	1.42583600	0.05559200
C	-1.47067700	-1.35985900	3.38287500
C	-2.49034300	0.72168500	2.67337200
C	-2.61952100	-1.66288600	4.11793600
H	-0.62315000	-2.03617100	3.38635700
C	-3.62999400	0.41865800	3.41883300
H	-2.43920100	1.65236000	2.11784700
C	-3.70006900	-0.77865000	4.13597800
H	-2.66579100	-2.59146300	4.68170300
H	-4.46135600	1.11910600	3.44124400
H	-4.58925400	-1.01598500	4.71459500
P	1.96416000	0.71120000	-0.39678800
P	-0.77300200	-1.50510300	-0.93328100
Rh	0.12262200	-0.06034900	0.92565300
C	0.92733000	-0.28158300	-2.93604000
H	1.58256700	-1.15216300	-2.82523900
H	0.93980100	-0.03043000	-4.00543500
C	-0.52221900	-0.66011800	-2.57592300
H	-0.94875400	-1.29492900	-3.36118600
H	-1.14290900	0.24052400	-2.52450000
C	1.51560100	0.93217100	-2.19009300
H	2.41223200	1.28949800	-2.71015300
H	0.79522200	1.75621400	-2.19451800

C	-2.59954700	-1.80902600	-0.98740000
C	-3.13366600	-2.90698600	-1.68241200
C	-3.47830700	-0.90904400	-0.36642200
C	-4.51416600	-3.09481400	-1.75865900
H	-2.47245800	-3.62412200	-2.15868300
C	-4.85950800	-1.10112100	-0.44354000
H	-3.08318100	-0.05892900	0.17515000
C	-5.38122800	-2.19257900	-1.13907100
H	-4.91056200	-3.95097200	-2.29839200
H	-5.52518200	-0.39895000	0.05142500
H	-6.45620700	-2.34334500	-1.19411200
C	-0.08982800	-3.20162000	-1.16400900
C	0.38105600	-3.68573700	-2.39497400
C	-0.08149600	-4.06350900	-0.05432300
C	0.85422700	-4.99486500	-2.51252800
H	0.37507800	-3.05474200	-3.27738300
C	0.38187100	-5.37267700	-0.17641800
H	-0.42283200	-3.70320400	0.90906000
C	0.85546700	-5.84110200	-1.40370000
H	1.21660900	-5.35068300	-3.47350600
H	0.38344000	-6.02315200	0.69377000
H	1.22393600	-6.85944400	-1.49480500
C	2.57126100	2.41103100	0.02511800
C	3.82880100	2.83545700	-0.43828000
C	1.78928800	3.30506700	0.76989400

C	4.28874400	4.12357400	-0.16561100
H	4.45946400	2.15491600	-1.00208500
C	2.25489400	4.59288900	1.04568400
H	0.81200100	3.00240700	1.12312500
C	3.50346800	5.00545400	0.57981300
H	5.26474600	4.43377400	-0.52929400
H	1.63934000	5.26919800	1.63269000
H	3.86587800	6.00610400	0.79994300
C	3.53409700	-0.25187400	-0.39613100
C	4.14057700	-0.72809400	-1.56801300
C	4.17666200	-0.47045400	0.83455000
C	5.35929300	-1.40981600	-1.51203800
H	3.68086100	-0.56676400	-2.53665100
C	5.39632400	-1.14149300	0.88603000
H	3.71743000	-0.12522600	1.75379600
C	5.98947400	-1.61719700	-0.28593500
H	5.81325000	-1.77288300	-2.43027000
H	5.87785200	-1.30240100	1.84642600
H	6.93731700	-2.14696100	-0.24207700

TS3-C

Cartesian coordinates

ATOM	X	Y	Z
C	0.27823400	2.40695100	-0.36326400
C	-1.38274900	1.41670000	-0.30562100

C	-2.54023200	1.82394800	-0.40976100
Rh	0.22428700	0.21344200	-0.07327300
Cl	0.12945300	0.40754700	2.37978000
C	-3.86137500	2.33333800	-0.49344300
C	-4.46671400	2.59600300	-1.74353800
C	-4.60970300	2.58943500	0.67911800
C	-5.76474500	3.09373800	-1.81418300
H	-3.90078700	2.40781000	-2.65142100
C	-5.90602200	3.08897400	0.59706500
H	-4.15431400	2.38815500	1.64379200
C	-6.49233400	3.34331300	-0.64690400
H	-6.21133700	3.29075400	-2.78569300
H	-6.46396400	3.28245100	1.50996500
H	-7.50507300	3.73289100	-0.70577100
C	0.33394000	-0.12289100	-1.89775000
C	0.58930000	2.92770300	-1.63719900
C	0.45804500	3.24471500	0.75491800
C	1.08412500	4.22435900	-1.78134200
H	0.41856300	2.33583600	-2.53051600
C	0.94928700	4.54080200	0.60011700
H	0.19525300	2.88014900	1.74063900
C	1.27378200	5.04020600	-0.66399100
H	1.31452700	4.59727800	-2.77694000
H	1.07377400	5.16732300	1.48068700
H	1.65555800	6.05119900	-0.77753300

P	2.56431200	-0.64595600	0.18828400
P	-0.93540100	-1.99587700	0.40243300
O	0.39999900	-0.35643500	-3.02335600
C	1.51881000	-2.89213100	1.66269600
H	1.35014400	-2.09604600	2.39517300
H	1.92731100	-3.74809700	2.21453500
C	0.18503100	-3.35300000	1.03916300
H	-0.37751700	-3.95672300	1.75967300
H	0.40281000	-4.00450000	0.18493800
C	2.57555500	-2.46100400	0.61915500
H	3.57613800	-2.73108100	0.97208400
H	2.41143100	-3.01315300	-0.31421900
C	-1.82085900	-2.78657700	-1.01705100
C	-2.40181600	-1.96180000	-1.99449000
C	-1.97713400	-4.17791900	-1.13323800
C	-3.11090200	-2.51418100	-3.06310900
H	-2.31436900	-0.88228500	-1.91183400
C	-2.67637500	-4.72858300	-2.20786700
H	-1.56629800	-4.84314500	-0.37999000
C	-3.24361800	-3.89840100	-3.17675400
H	-3.55489400	-1.85934100	-3.80785600
H	-2.78351700	-5.80753200	-2.28299300
H	-3.78889900	-4.32855300	-4.01245400
C	-2.27602600	-1.86711400	1.65903400
C	-2.05331800	-2.18315600	3.00788600

C	-3.53564600	-1.37853100	1.27799000
C	-3.07496000	-2.04093400	3.94715900
H	-1.07898200	-2.52420700	3.34098700
C	-4.55210500	-1.22879000	2.22116800
H	-3.73189400	-1.11425600	0.24483500
C	-4.32710900	-1.56404600	3.55742700
H	-2.88511200	-2.29356000	4.98693500
H	-5.51933200	-0.84663800	1.90652700
H	-5.12058500	-1.44941000	4.29112900
C	3.71962900	0.11033100	1.41943800
C	3.39227800	1.31306000	2.05808700
C	4.97215100	-0.47574000	1.66976700
C	4.29480500	1.90671800	2.94472900
H	2.43237800	1.77735500	1.87391700
C	5.86748500	0.11566600	2.55893300
H	5.26744300	-1.38642700	1.15542600
C	5.52770000	1.30898400	3.20218700
H	4.02502500	2.83728400	3.43647900
H	6.83173400	-0.35057800	2.74357700
H	6.22544800	1.77105300	3.89566900
C	3.58758800	-0.55616400	-1.35081800
C	3.67544700	0.69233700	-1.99192300
C	4.28541300	-1.64370500	-1.89724300
C	4.44475200	0.84657100	-3.14416800
H	3.13520800	1.54663300	-1.59189900

C	5.04945000	-1.48791400	-3.05679100
H	4.24700700	-2.62084500	-1.42644100
C	5.13251300	-0.24402900	-3.68200500
H	4.50165200	1.81941800	-3.62489200
H	5.58113800	-2.34224000	-3.46760500
H	5.72761100	-0.12464200	-4.58329200
